# Transesterification of cellulose with unactivated esters in superbase–acid conjugate ionic liquids†

**DOI:** 10.1039/d2ra08186e

**Published:** 2023-02-17

**Authors:** Aleksandar R. Todorov, Alistair W. T. King, Ilkka Kilpeläinen

**Affiliations:** a Materials Chemistry Division, Department of Chemistry, University of Helsinki FI-00560 Helsinki Finland aleksandar.todorov@helsinki.fi; b VTT Technical Research Centre of Finland Ltd Tietotie 4e 02150 Espoo Finland

## Abstract

A sustainable homogeneous transesterification protocol utilizing the superbase ionic liquid [mTBNH][OAc] and unactivated methyl esters has been developed for the preparation of cellulose esters with controllable degree of substitution. [mTBNH][OAc] shows excellent recyclability with a high recovery of sufficient purity for repeated use. This reaction media allows for cellulose transesterification reactions not only using activated or cyclic esters, but also with unactivated methyl esters, which extends the substrate and application scope. Furthermore, the solubility properties of the prepared cellulose materials were tested and some intrinsic trends were observed at low degrees of substitution.

## Abstract 2

Controllable homogeneous cellulose transesterification with unactivated methyl esters.

## Graphical abstract

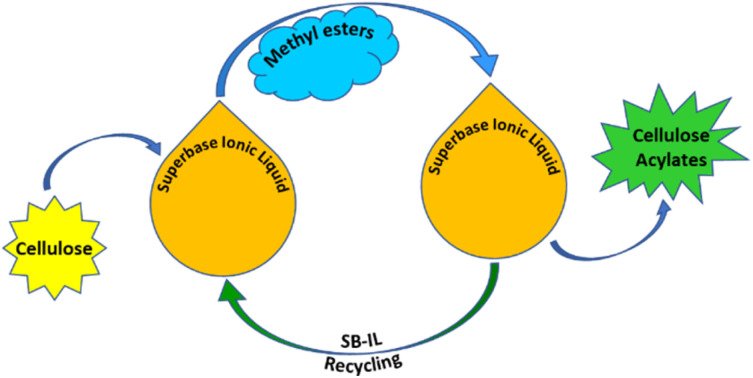

## Introduction

Before the formal isolation of cellulose (elemental composition determination) in 1838, technical or native cellulosics have been mainly used as an energy source, building materials, and for paper, and textiles, on a primitive level.^1^ The first key modification of cellulose dates back to 1870, where the first industrial polymer material (celluloid) was obtained by nitration using nitric acid.^2^ The earliest and most important examples of cellulose derivates were the production of inorganic esters (nitrates, sulphates, xanthogenates) and later organic esters (mainly acetate). While the former esters have been mostly used as protective coatings, films, explosives and in textile fibre manufacture,^3^ the ‘organic’ (note: both types are actually organic by definition) cellulose esters have gained popularity for their easy processability (solvent and melt) combined with their chemical stability. Their application ranges from manufacturing of textiles,^4^ transparent films,^5^ membranes,^6^ and cigarette filter tow^7^ to development of novel drug delivery systems.^8,9^ Furthermore, cellulose acetates (CA) with degrees of substitution (DS) between 0.5 and 1.1 represent good water solubility properties,^10^ which has been beneficial for certain applications *e.g.* film coating for oral tablets.^11^ Although these key cellulose esters have been commercially applied for decades, to date, there still are a few challenges with the production of main bulk commercial ‘organic’ esters. With acetylation as the prime example, its production and product specifications rely on heterogeneous acid-catalysed esterification – this proceeds to cellulose triacetate (CTA), with lower DS values only achievable by deacetylation to, *e.g.* the solvent processible cellulose diacetate (CDA, with a DS value of ∼2.4). Also, these heterogeneous production conditions are not so flexible in the range of substituents possible (acetate, propanoate, butyrate, and mixtures thereof, as the predominant bulk products).^12–14^ Economical access to lower DS values, with wider application scope has long been suggested to be possible with direct-dissolution solvents. However, to date no one has demonstrated high recyclability potential *via* this method, due to current cellulose solvent complexities.

The presence of strong intra- and inter-molecular hydrogen-bonds in the cellulose crystal structure is prime factor in preventing the solubility of cellulose in common molecular solvents. However, it is not only the breakage of hydrogen-bonds that is necessary to achieve solubility as also hydrophobic interactions play a role in the interaction of cellulose chains. Therefore, amphiphilic solvents are necessary,^15^ thus increasing the complexity of the solvent media. Solvents such as *N*,*N*-dimethylacetamide-lithium chloride (DMA-LiCl), dimethyl sulfoxide-tetrabutyl ammonium fluoride (DMSO-TBAF) and aqueous *N*-methylmorpholine-*N*-oxide (*N*MMO) are now classical systems for cellulose dissolution.^16^ However, their hazard potential, high cost (in some cases), and instability make them unpractical or, if not properly controlled, even hazardous in industrial usage.^17–19^

**Scheme 1:**
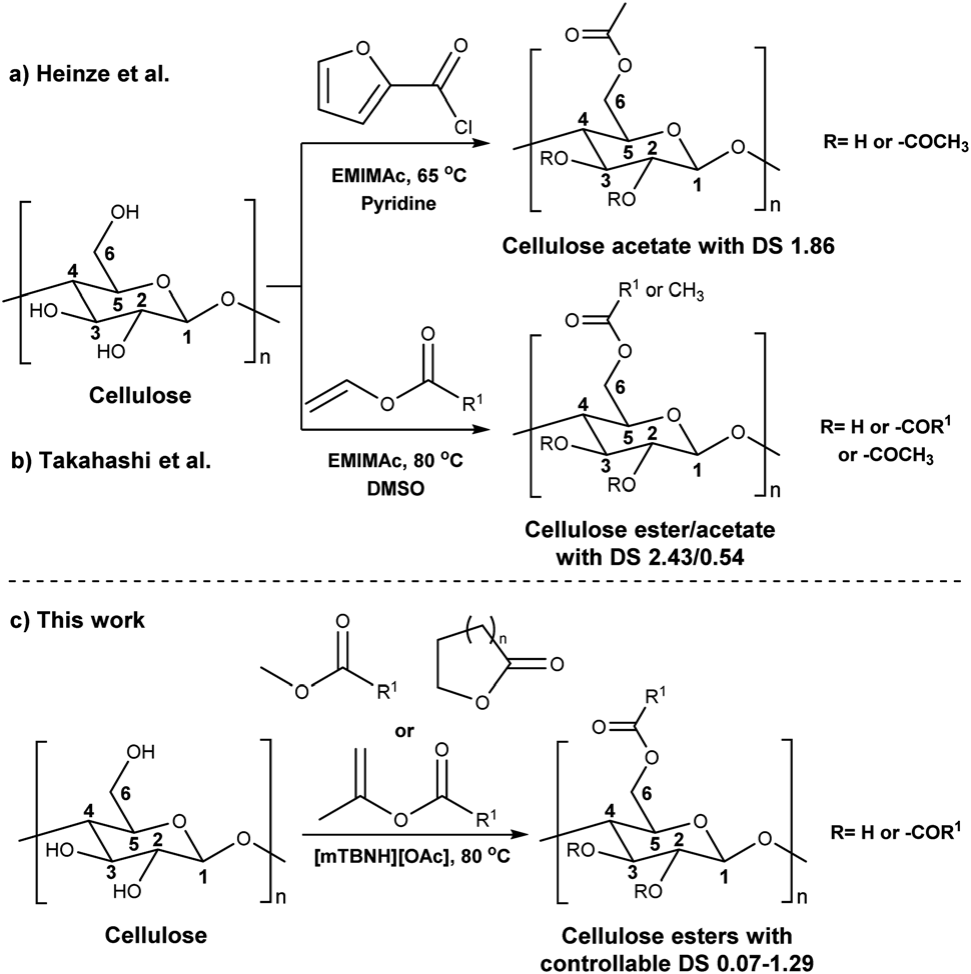
Cellulose modification.

In addition to the common utilization of simple cellulose derivatives, they are often considered as potential alternatives to fossil-based polymer products. However, often cellulosic materials do not have the property of melt processability, which keeps fossil-based polymers very far ahead of the game. Thus, cost reduction, linked to recyclability, in homogeneous processing is necessary. Moreover, to be able to preserve the sustainable properties of cellulosics, their design and synthesis should be compatible with the principles of green chemistry.^20^ The syntheses should be atom- and energy efficient, preferentially catalytic in nature, utilizing non-hazardous chemicals, *etc.* To address some of these points the cellulose solvent selection is critical. An ideal cellulose solvent should have low vapor pressure, to avoid possible VOCs (volatile organic compounds) emissions, since cellulose dissolution usually requires elevated temperatures, and should be non-toxic. Considering these criteria, a reasonable reaction media for cellulose modifications could be a class of molten salts, *i.e.* ionic liquids (ILs), which virtually can be tuned to perfection.^21^ The growing number of publications utilizing ILs for cellulose modifications clearly show their capability to replace the classical archetypical cellulose solvents.^22^ Recently, ILs composed from the 1-ethyl-3-methylimidazolium cation and different carboxylate anions *e.g.*, acetate, benzoate, pivalate, *etc.*, have shown promising potential as they have not only served as solvents, but also as catalysts in the synthesis of cellulose esters.^23–25^ However, the catalytical activity of the imidazolium-based ILs could originate from carbene formation during the reaction and/or from the mixed anhydride formation between the anions and the acyl donors.^26–29^ Even though these ILs can produce cellulose esters with high DS, the mixed anhydride formation (with the carboxylate anion of the IL) often leads to mixed esters – which can complicate recycling of the IL. Seemingly the side product formation depends on the acyl donor and in some cases, it can override the originally desired synthesis (Scheme 1a and b).^27,30,31^ Another class of ILs, namely superbase ionic liquids (SB-ILs), could potentially be used as an alternative to the archetypical cellulose solvents and the imidazolium ILs. These ILs are composed from equimolar amounts of organic superbase (a compound which has very high affinity to protons) *e.g.*, amidine or guanidine and carboxylic acid *e.g.*, formic, acetic, propionic acid, *etc.* SB-ILs based on the bicyclic guanidine core are particularly attractive, because they have showed excellent capabilities for cellulose dissolution and they are already used in the manufacturing of regenerated cellulosic materials, like textile fibres.^32–35^ Notably, their neutral superbase cores have been successfully used as organic base catalysts in many different transformations.^36–38^ Noteworthy, it is possible (to some extent) to alter the composition of the SB-IL by using an excess of the acid or the base component, while retaining its cellulose dissolution capability. This can enable utilization of different reaction pathways for chemical modification reactions.

Consequently, these SB-ILs could potentially not only serve as reaction media but may also enhance the reactivity of cellulose. This may provide means for utilization of less reactive, less toxic, and less harmful acyl donors (*e.g.*, unactivated esters) for cellulose derivatization (Scheme 1c). Moreover, the usage of SB-ILs together with unactivated acyl donors offers better chances for easy recovery of by-products, that do not get ‘trapped’ in the basic solvent.

**Chart 1:**
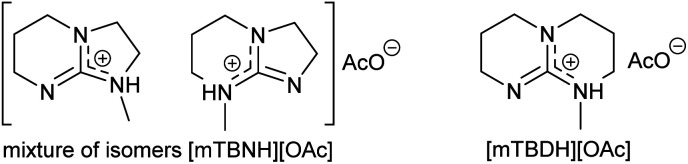
Structural representation of the used SB-ILs in this study.

Herein, we report a sustainable procedure for synthesising materials with various DS values through transesterification of cellulose with unactivated esters, moderately activated (isopropenyl), and cyclic esters (lactones) using a novel cellulose direct-dissolution SB-IL (Chart 1), developed in our research group.^39^

## Results and discussion

### Reaction optimization

To assess the preliminary reactivity for transesterification, reactions of cellulose in the SB-IL 5/7-methyl-1,5,7-triazabicyclo[4.3.0]non-5-enium acetate [mTBNH][OAc] (mixture of isomers), using unactivated esters—we selected microcrystalline cellulose (MCC) Avicel® PH-101 purchased from Sigma Aldrich as a model compound. To evaluate how the reaction conditions affect the achievable DS values, we varied several reaction parameters, *i.e.*, type and amount of reagent, reaction time and temperature, composition of the SB-IL (Tables 1 and 2), and as well as the SB-IL unconjugated species themselves (Table 3 entry 1 and 2).

**Table 1:** Reaction parameters variations^a^

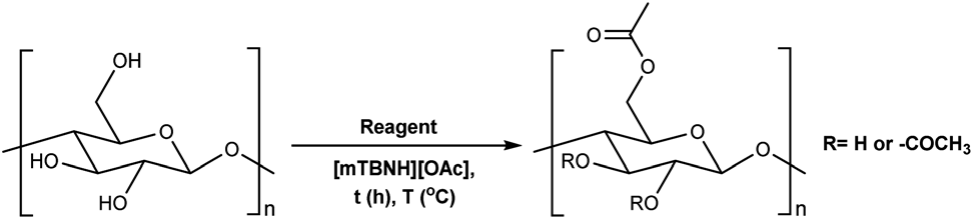					
Entry	Reagent	Equivalents	*T* (°C)	*t* (hours)	DS^b^
**Reagent** c					
1	MeOAc	3	80	20	0.08
2	EtOAc	”	”	”	0.05
3	i-PrOAc	”	”	”	0.05
4	*t*-BuOAc	”	”	”	0.04
5	No reagent	”	”	”	—

**Reagent amount (equivalents to AGU)**					
6	MeOAc	1	80	20	0.05
7	”	2	”	”	0.07
8	”	3	”	”	0.08
9	”	4	”	”	0.09
10	”	5	”	”	0.10
11	”	6	”	”	0.12
12	”	9	”	”	0.15
13	”	12	”	”	0.19
14	”	15	”	”	0.21

**Reaction time (hours)**					
15	MeOAc	3	80	24	0.09
16	”	”	”	48	0.15
17	”	”	”	72	0.19

**Reaction temperature (°C)**					
18	MeOAc	3	65	20	0.05
19	”	”	80	”	0.08
20	”	”	100	”	0.18

a Reactions were performed in sealed 8 ml vials, where the desired amount of reagent was added to 50 mg of MCC dissolved in 1 ml of [mTBNH][OAc], then the reaction was stirred at the specified conditions.

b DS values are an average of two reactions and were determined by diffusion-edited ^1^H NMR at 65 °C with a standard calibration curve against DS ranges, determined under quantitative conditions. (—) Under the detection limit of the method.

c Methyl acetate (MeOAc), ethyl acetate (EtOAc), iso-propyl acetate (i-PrOAc) and *tert*-butyl acetate (*t*-BuOAc).

**Table 2:** Variation of the ionic liquid unconjugated acid–base ratio^a^

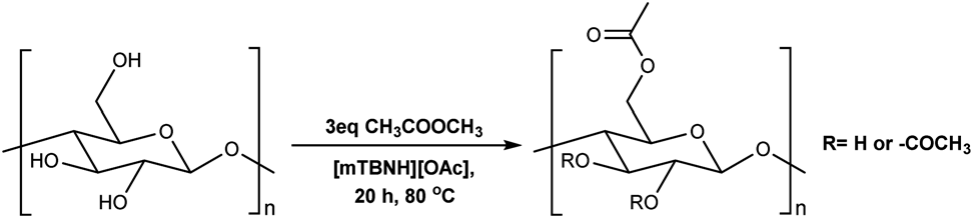		
Entry	Varied ionic liquid composition	DS (MeOAc/blank/MeOPr)^b^^,^^c^^,^^d^
1a, b, c	[mTBNH][OAc] 1 : 1.5	0.06/—/—
2a, b	[mTBNH][OAc] 1 : 1.25	0.07/—
3a, b, c	[mTBNH][OAc] 1 : 1	0.08/—/—
4a, b	[mTBNH][OAc] 1.25 : 1	0.09/—
5a, b, c	[mTBNH][OAc] 1.5 : 1	0.10/—/—
6a, b	[mTBNH][OAc] 1.75 : 1	0.12/—
7a, b, c	[mTBNH][OAc] 2 : 1	0.11/—/—
8a, b	[mTBNH][OAc] 3 : 1	0.14/—
9a, b	[mTBNH][OAc] 4 : 1	0.13/—
10a, b, c	[mTBNH][OAc] 5 : 1	0.14/—/—
11a, b, c	[mTBNH][OAc] 10 : 1	0.16/—/—

a Reactions were performed in sealed 8 ml vials, where the reagent was added to 50 mg of MCC dissolved in 1 ml of the corresponding [mTBNH][OAc] mixture, then the reaction was stirred at 80 °C for 20 h.

b DS values are an average of two reactions and were determined by diffusion-edited ^1^H NMR at 65 °C with a standard calibration curve against DS ranges determined under quantitative conditions.

c DS for acetylation determined from the same reaction conditions, but without added reagent.

d DS for acetylation determined from the same reaction conditions, but with MeOPr as reagent instead of MeOAc. (—) Under the detection limit of the method.

**Table 3:** Substrate scope of unactivated esters^a^

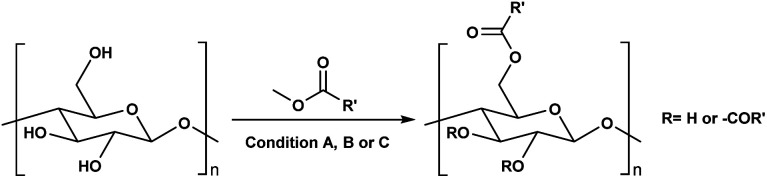		
Entry	R′	DS (condition A, B or C)^b^
1	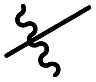	0.07/0.13/0.13
2^c^	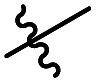	0.09/0.17/0.22
3^d^	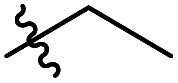	0.06/0.10/0.08
4^d^	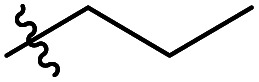	0.04/0.05/0.05
5^d^	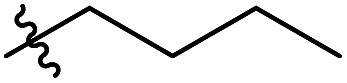	0.04/0.05/0.06
6	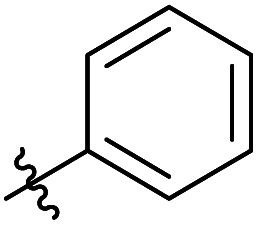	0.02/0.05/0.02
7	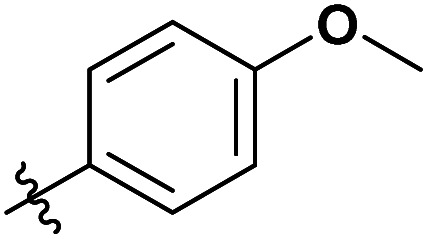	0.005/0.01/0.01
8	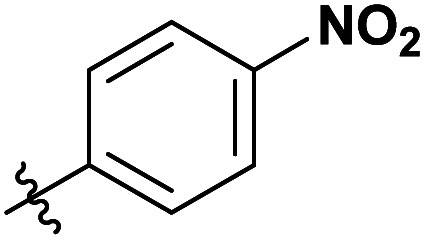	0.25/0.75/0.03
9	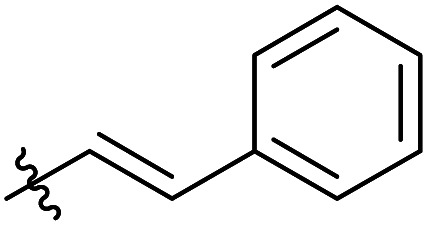	0.03/0.07/0.07

a Reactions were performed in 50 ml round bottom flask, where the corresponding reagent was added to 250 mg of MCC dissolved in 5 ml of [mTBNH][OAc], then the reaction was stirred at 80 °C. Condition A: 3 equivalents of reagent, 20 h reaction time. Condition B: 9 equivalents of reagent, 20 h reaction time. Condition C: 3 equivalents of reagent, 50 h reaction time.

b DS of isolated sample determined by ^1^H NMR at 65 °C in 20 wt% tetrabutylphosphonium acetate in DMSO-d_6_.

c [mTBDH][OAc] was used instead of [mTBNH][OAc], temperature used was 85 °C instead of 80 °C.

d DS of isolated sample determined by diffusion edited ^1^H NMR at 65 °C with a standard calibration curve against DS ranges determined under quantitative conditions.

To determine our next steps, we begin with the evaluation of the suitability of the unactivated esters (Table 1 entry 1–4), specifically focusing on the achievable DS values. The choice of acetate esters was obvious, firstly because of the availability of the reagents and secondly because of the relative simplicity of the expected products and non-acidic by-products. Evidently, from the results the reactivity of the esters follows a predictable homologous reactivity order (*t*-Bu < i-Pr < Et < Me), expectedly due to sterics. The bulky *tert*-butyl group in the *tert*-butyl acetate (*t*-BuOAc) restricts the access of the reagent to the cellulose hydroxyl groups, whereas the methyl group in the methyl acetate (MeOAc) is much less hindered, reaching a DS value two times higher. Omitting the ester from the reaction (Table 1 entry 5) leads to no acetylation, which directly suggests that the IL alone does not contribute to the acetylation of the cellulose. A detailed study of the amount of MeOAc added to the reaction mixture compared to the anhydroglucose unit (AGU) of cellulose, revealed that the achievable DS values are strongly dependent on stoichiometry (Table 1 entry 6–14). DS values as high as 0.21 could be achieved by adjusting the amount of reagent, allowing for more flexibility in the cellulose ester compositions, at these mild temperatures. Further increasing the amount of the reagent was not possible, as the cellulose precipitates out of the reaction mixture and the reaction progresses from homogeneous to heterogeneous. As expected, increasing both the reaction time and the temperature increases the DS of the obtained cellulose materials (Table 1 entry 15–20). Interestingly, the increase in the time or the temperature gives comparable results, to the increase in the amount of the reagent (Table 1 entry 13, 17 and 20), thus, providing us with more flexibility in reaction optimisation.

### Mechanistic insights

**Scheme 2:**
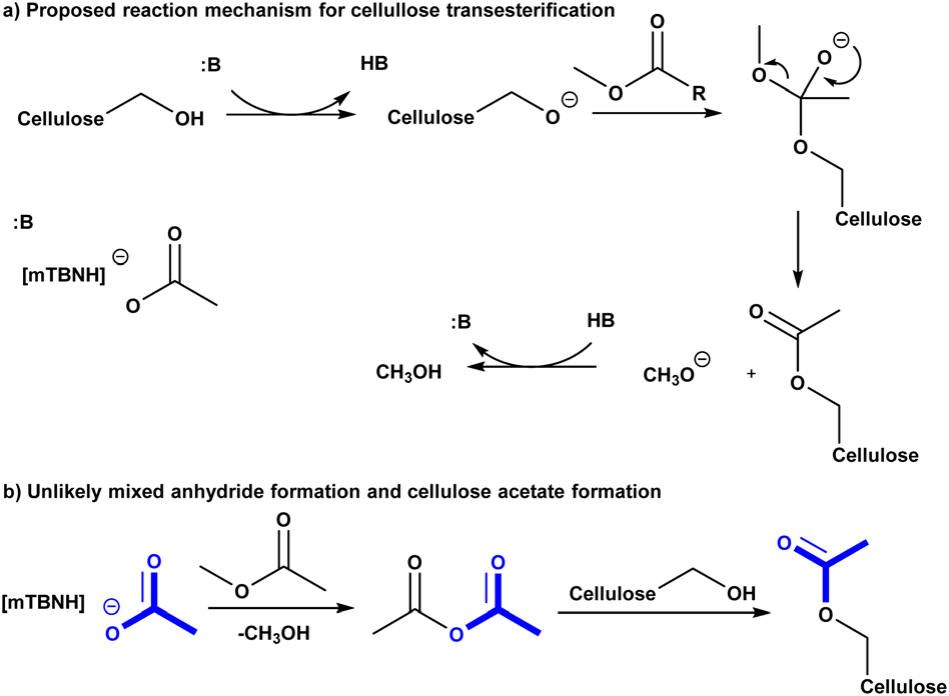
(a) Proposed reaction mechanism for cellulose transesterification. (b) unlikely mixed anhydride formation and cellulose acetylation.

To get deeper understanding of the reaction mechanism we performed a series of experiments with variable IL unconjugated acid-base ratio compositions (Table 2). However, it was not clear at this stage if excess acid or base would prevent the cellulose dissolution or IL recycling, at some stoichiometry. Having in mind these mechanistic and scope-limiting questions, we have decided to enrich the SB-IL with its own SB. To our delight, the increase of the base component in the IL leads to increase in DS of the obtained cellulose materials (Table 2 entry 1a–11a). Comparing the results, we have noticed that the DS of the materials has doubled when the maximum amount of added base has been used (Table 2 entry 3a and 11a). Increase of the SB component beyond this composition was not possible because the cellulose could no longer be dissolved – transition from homogeneous to heterogeneous conditions. The gradual increase in the DS of the obtained materials, with the increase of the SB component in the IL, strongly suggests that the transesterification reaction of the cellulose is highly dependent on base catalysis. An ionic mechanism is proposed (Scheme 2a).

In order to assess if the SB-IL can contribute to acetylation of the cellulose itself, we have performed control experiments where the acylating reagent was omitted. To our delight, the experiments show with no doubt that SB-IL does not contribute to the acetylation of cellulose (Table 2 entry 1b–11b). To elaborate further this finding, we performed series of experiments, where the MeOAc was replaced by MeOPr (Table 2 entry 1c, 3c, 5c, 7c, 10c and 11c). The experimental data clearly show that there was no mixed acylation observed, *via* formation of a possible mixed anhydride intermediate (Scheme 2b), clearly indicating that the SB-IL is not implicated in the chemical transformation itself. This is a very positive result regarding the scope of functionalities that can be incorporated, maintaining recyclability of the SB-IL.

**Fig. 1:**
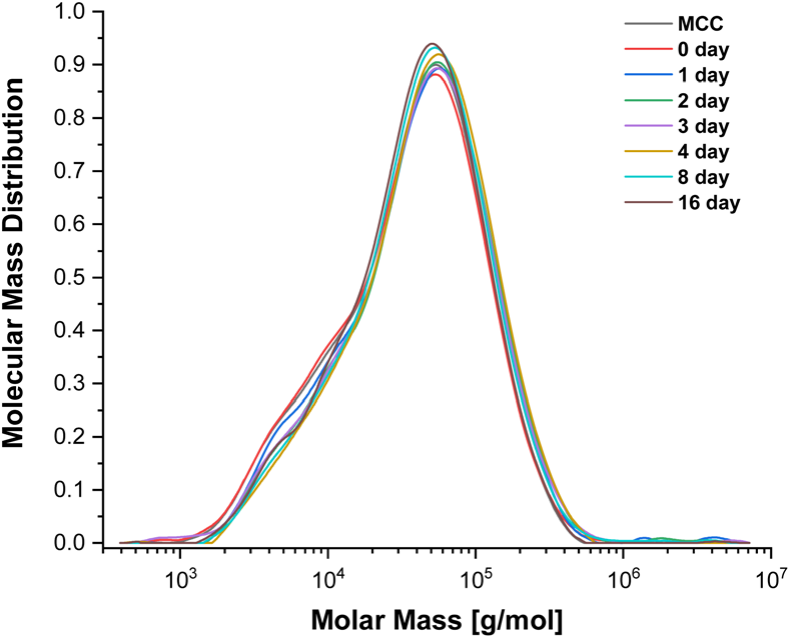
Molecular weight distribution of MCC treated in [mTBNH][OAc] at 80 °C.

To evaluate the potential for cellulose depolymerisation, under the tested reaction conditions, we have performed a prolonged (16 day) experiment in the SB-IL at 80 °C. The obtained results from gel permeation chromatography (GPC), yielding the molar mass distributions (Fig. 1), suggested to us that there was no major degradation of the cellulose chains during the 16 days of the experiment. The number average molecular weights (DP_*n*_) of the obtained samples remained very similar to the initial untreated and treated cellulose, within expected error ranges (Table S2†).

### Substrate scope – unactivated esters

Next, we studied the scope of the reaction (Table 3), where we chose three different reaction conditions to produce different DS values. Condition A was the standard reaction conditions used in the initial optimisation: 3 eq. of methyl esters – 20 hours – 80 °C – MCC. Conditions B and C were chosen to produce materials with higher DS values, by increasing reagent stoichiometry or reaction time. Namely, condition B include 9 eq. of methyl ester, while condition C used the standard amount of reagent, but the time was increased from 20 hours to 50 hours. We studied the scope of the reaction with aliphatic methyl esters (Table 3 entry 1–5) with increasing carbon chain length, from 2 carbon atoms to 5. The reactivity of the aromatic methyl esters (Table 3 entry 6–8) was modulated with different substituents, which alter the electronic properties of the reagents (electron neutral, rich, or deficient). Additionally, methyl cinnamate was used to illustrate the reactivity of unactivated conjugated esters, to further reactive sites (Table 3 entry 9).

Upon increasing the carbon chain length of the aliphatic methyl esters (Table 3 entry 1–5) we noticed a slight decrease in the reactivity. This is not unexpected and is potentially related to increasing aggregation of the more hydrophobic chains or simply increase in molecular weight of the reagent, reducing molecular collisions. The change of the SB-IL from [mTBNH][OAc] to [mTBDH][OAc] allowed for a slight increase in DS values (Table 3 entry 1 and 2). This is simply explained by the higher reaction temperature needed to perform the reaction. Reaction temperature for this set of experiments was set at 85 °C, because the SB-IL [mTBDH][OAc] has a melting point of 82–83 °C. Expectedly, changes in the reaction conditions enabled us to produce cellulosic materials with different DS values. Evidently, the increased amount of the reagent or the reaction time, leads to higher DS values. While the reactivity of the unactivated aliphatic esters was similar in all cases, the reactivity with the unactivated aromatic esters was quite different (Table 3 entry 6–8). Expectedly, from the obtained results the electron rich unactivated aromatic ester (Table 3 entry 7, *p*-methoxy) showed the lowest reactivity, while the electron neutral unactivated aromatic ester (Table 3 entry 6, *p*-H) show similar reactivity to the unactivated aliphatic esters. The electron deficient unactivated aromatic ester (Table 3 entry 8, *p*-nitro) showed excellent reactivity compared to the other unactivated esters used in this study. The obtained DS value of 0.25, under the standard reaction conditions, was tripled upon increase of the reagent to 0.75, while it plummets to 0.03 upon increasing of the reaction time – this is rather unusual reactivity that should be investigated in future studies. Other aromatic ester yields were too low to draw similar conclusions. Similar DS values, like the one obtained from the reactions with the unactivated aliphatic esters, were obtained when the unactivated conjugated ester (Table 3 entry 9) was used. Importantly, the mild reaction conditions allow us to embed such fragments as activated double bonds into the prepared cellulosic materials, giving us the opportunity for further modification or cross-linking of the isolated materials.^40^ Furthermore, we found out that we could prepare cellulosic materials with higher DS by simply reprocessing the recovered cellulosic materials under the same reaction conditions. Utilizing this enrichment strategy, we were able to prepare CAs samples with linearly progressing DS values from 0, 0.07, 0.12 to 0.21 after three consecutive reaction cycles. Plausibly the same enrichment strategy could be applied for the preparation of mixed cellulose esters with variable substitution patterns and DS, thus providing us with virtually unlimited possibilities for product scope.

### Substrate scope – activated esters, and recyclability tests

To evaluate further the potential of the SB-IL [mTBNH][OAc] as a reaction media for cellulose transesterification, we performed series of experiments with more activated esters. As model activated esters, we choose isopropenyl acetate and vinyl acetate. To our delight, both activated esters performed well under the developed reaction conditions, producing materials with DS values of 1.75 and 1.44 (Table 4 entry 2 and 3). Naturally, the activated esters outperformed the unactivated esters (Table 4 entry 2, 3 and 1).

**Table 4:** Reactivity differences between unactivated and activated esters^a^

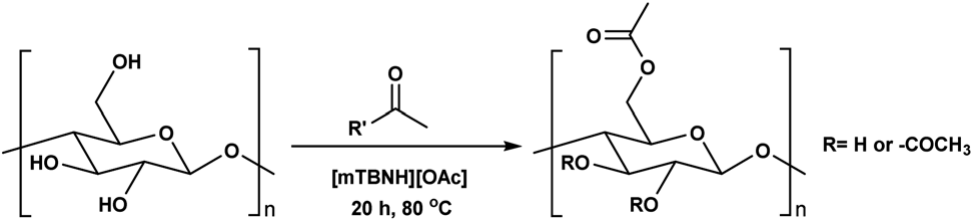		
Entry	R′	DS^b^
1	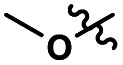	0.07
2	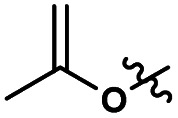	1.75
3	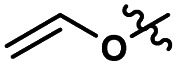	1.44

a Reactions were performed in 50 ml round bottom flask, where 3 equivalents of the corresponding reagent were added to 250 mg of MCC dissolved in 5 ml of [mTBNH][OAc], then the reaction was stirred at 80 °C for 20 h.

b DS of isolated sample determined by ^1^H NMR at 65 °C in 20 wt.% tetrabutylphosphonium acetate in DMSO-d_6_.

While, obtaining cellulosic materials with various degree of substitution was important for us, their solubility properties are a key test point for application interest (see subsection Solubility tests). To get materials with lower DS, which should allow for water processibility, we deliberately lowered the reaction temperature from 80 °C to 65 °C, with isopropenyl acetate as reagent. Further, simple variation in the amounts of added reagent gave us the opportunity to prepare materials with adjustable and consistent DS values, varying from 0.11 to 1.29 (Table 5 entry 1–6). While we decreased the temperature of the reaction from 80 °C to 65 °C, the observed DS values dropped from 1.75 to 1.29 (Table 4 entry 2 and Table 5 entry 6), thus, suggest that the reaction is strongly dependent on the reaction temperature.

**Table 5:** Transesterification with isopropenyl acetate^a^

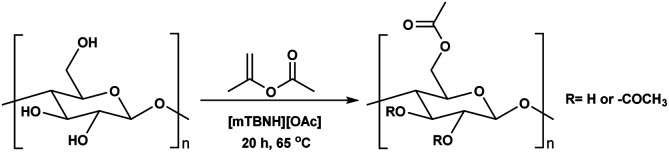		
Entry	Equivalents of reagent to AGU	DS^b^
1	0.5	0.11
2	1	0.32
3	1.5	0.59
4	2	0.72
5	2.5	1.07
6	3	1.29

a Reactions were performed in 100 ml round bottom flask, where *x* equivalents of the isopropenyl acetate were added to 500 mg of MCC dissolved in 10 ml of [mTBNH][OAc], then the reaction was stirred at 65 °C for 20 h.

b DS of isolated sample determined by ^1^H NMR at 65 °C in 20 wt% tetrabutylphosphonium acetate in DMSO-d_6_.

**Fig. 2:**
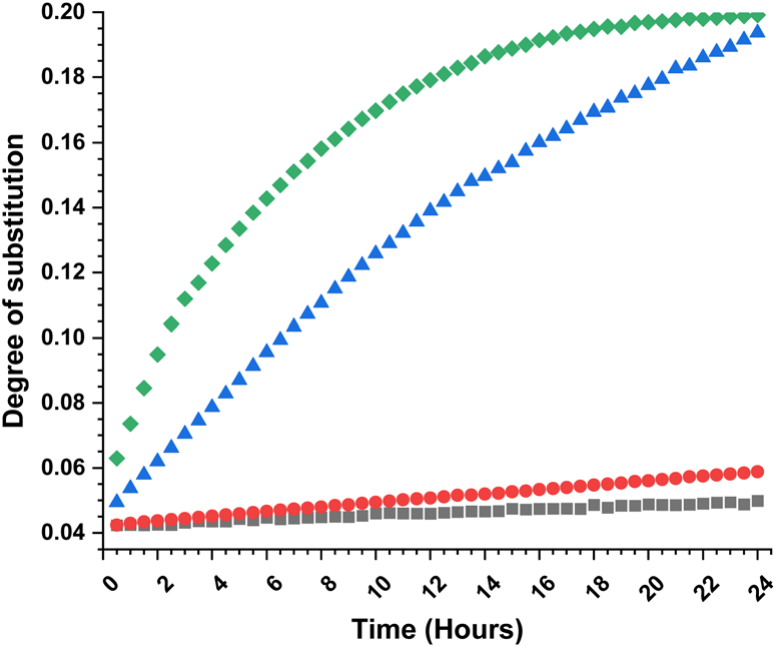
Combined kinetic plot (degree of substitution *vs.* time) for cellulose acetylation. Black squares and red balls – transesterification with MeOAc at 65 °C and 80 °C, blue triangles and green diamonds – transesterification with i-propenylOAc at 65 °C and 80 °C. The DS values were determined from the diffusion edited ^1^H NMR with standard calibration curve.

To obtain further insights of the reaction efficiency, we carried out a set of kinetic experiments, where we compared the cellulose transesterification with unactivated and activated esters at 65 °C and 80 °C – the DS values were followed in the NMR tube using a calibration-corrected diffusion-edited ^1^H experiment (details could be found in the ESI†).^41,42^ The experiments were performed as closely as possible to the original reaction conditions. However, there were a few deviations from the original conditions *e.g.*, presence of DMSO-d_6_, which was used to reduce the viscosity of the sample and allow for the correct diffusion coefficient ranges; no mechanical stirring of the sample, resulting in limited diffusion of the reagent into the reaction mixture; decreased amount of reagent (for the iso-propenyl acetate reactions). Despite these differences, the gained information from the experiments had great value (Fig. 2). Evidently, the reaction temperature plays a critical role in the transesterification of cellulose. The kinetic experiments performed with the methyl ester showed clear trend of increasing DS, with increasing reaction temperature. The DS values for the 80 °C reaction were similar (0.06 *vs.* 0.07) compared to the values obtained from the isolated samples. The kinetic experiments performed with the isopropenyl esters showed an intriguing trend. The transesterification performed at 65 °C steadily progressed with time, while the reaction performed at 80 °C showed an initial rapid reaction rate. Another interesting feature is the fact that at the end of both reactions, the DS values are almost similar – 0.2. Plausibly, due to the same reaction differences the transesterification at 65 °C has been positively impacted and proceeds to higher DS values than the isolated samples (0.19 *vs.* 0.11).

**Fig. 3:**
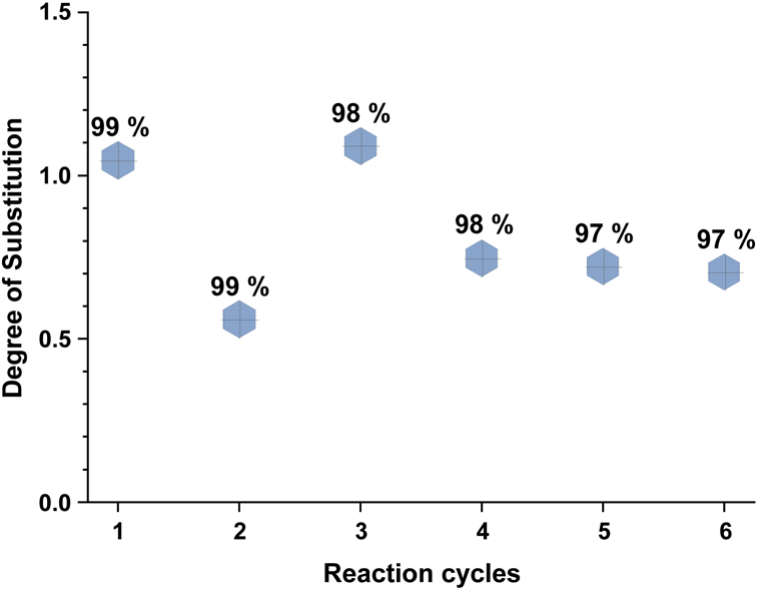
Recyclability of the reaction media over six reaction cycles. Reactions were performed in 100 ml round bottom flask, where 2 equivalents of isopropenyl acetate were added to 500 mg of MCC dissolved in 10 ml of [mTBNH][OAc], then the reaction was stirred at 65 °C for 20 h. DS of isolated sample determined by ^1^H NMR at 65 °C in 20 wt% tetrabutylphosphonium acetate in DMSO-d_6_. Percentage denotes the recovery rate of the IL.

**Fig. 4:**
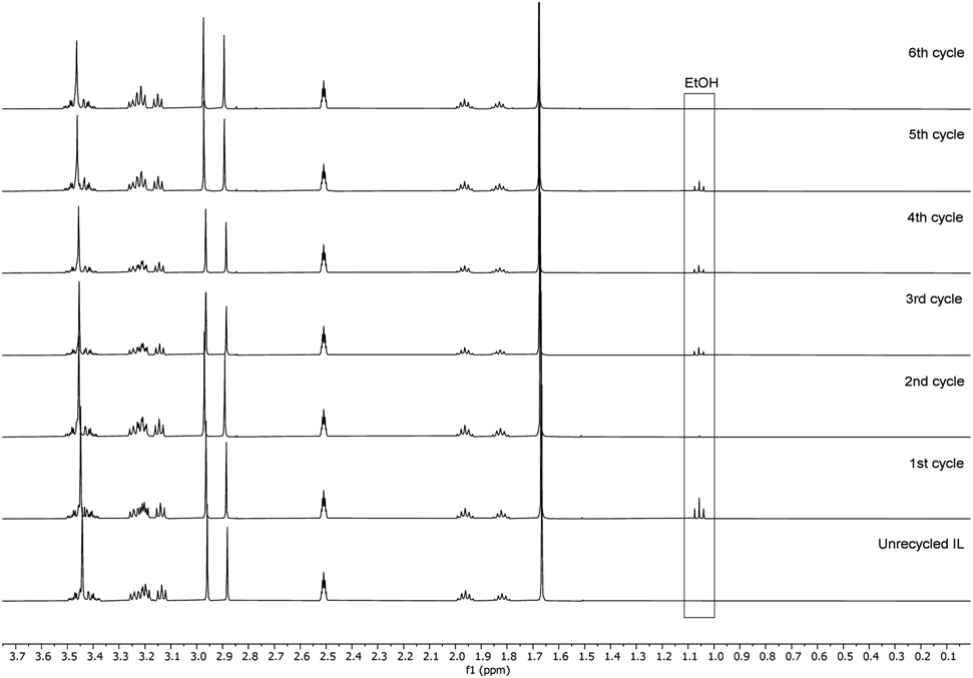
^1^H NMR of the recycled IL in DMSO-d_6_.

Recyclability of the reaction media, the unreacted reagents, and the cellulose regeneration solvents has always been a major criticism in the development of homogeneous esterification methods, utilising direct-dissolution solvents. To verify the recyclability of our reaction media, a series of experiments was performed with recovered IL – reaction conditions were the same as in the Table 5 entry 4. We have performed six successive cycles of reaction and recovery of the used IL – the results have been summarized in Fig. 3. After each reaction cycle the reaction mixture was diluted with ethanol in order to recover the formed cellulose material. After filtration and further washing of the isolated cellulose material, the combined ethanol washings were evaporated, and the IL was recovered (detailed procedure could be found in the ESI†). NMR analysis of the recovered IL, after each cycle, demonstrated convincingly that there is no degradation of the IL (Fig. 4). Some traces of ethanol (the anti-solvent used for recovery of the cellulose material) could be seen in the shown NMR, but not in the final product. The overall recovery of the IL after the sixth reaction cycle was 97% (0.5% loss per cycle), with no degradation in its cellulose dissolution capabilities. Each cycle proceeded consistently, producing the DS values with some initial variability but convergence at later cycles – this variability seems to be linked with some fluctuations of the reaction temperature and/or the amount of residual ethanol in the recovered ILs, which may function to consume some of the acetylating agent, resulting in lower DS values. To verify the plausible deactivation effect of the residual ethanol, a series of control experiments were performed (ESI† for details). We replicated the same reaction conditions as in the recycling experiments replacing the recycled IL with ethanol containing IL (1, 5, and 10 v/v%). Remarkably, even 10 v/v% of ethanol did not deteriorate the IL cellulose dissolution capability. These control experiments showed a clear trend of deactivation upon increasing of the added amount of ethanol and we registered a drop in the DS up to 0.24 (*vs.* DS 0.72 Table 5 entry 4). To confirm further the sustainability of the reaction conditions, we recovered the anti-solvent used for the cellulose recovery. The rate of recovery was found to be between 96 and 99%.

### Substrate scope – cyclic esters

Encouraged from the excellent recyclability results and the already broad scope of substrates *e.g.*, unactivated and activated esters, we decided to investigate further expansion of the plausible substrate scope. A series of experiments performed under the same reaction conditions, as for cellulose transesterification with unactivated esters, revealed that some lactones were indeed highly reactive. As model substrates we chose cyclic esters with different ring sizes, from 4 to 7, and different substitution pattern. We also choose methyl glycolate as an open chain analogue. The results of these trials are shown in Table 6. Clearly, the reaction of cellulose with methyl glycolate (Table 6 entry 1) shares similarities to the reactions using other unactivated aliphatic esters (Table 3 entry 1–5). The obtained material has a similar DS. However, also grafting was detected (degree of grafting (DG) 1.57). From the tested lactones, those with ring size 4 and 5 were inactive under these reaction conditions (Table 6 entry 5–7), except for α-angelica lactone (Table 6 entry 4), which produces material with only low DS of 0.01 and no detectable grafting. The material prepared with the δ-valerolactone (Table 6 entry 3) has a similar DS to the one prepared from the methyl glycolate but has a slightly longer graft length of 2.01 *vs.* 1.57. ε-Caprolactone, the 7 membered cyclic ester, showed the greatest reactivity under the employed reaction conditions. The DS of the newly obtained cellulosic material has been determined to be 0.24, six times higher than the δ-valerolactone, and the average degree of grafting was determined to be 2.29 (Table 6 entry 2). The similarly obtained average degrees of grafting, between the different lactones and the methyl glycolate, suggests that most likely the grafting takes place after the attachment of the monomer substituent to the cellulose *i.e.*, ‘grafting from’ rather than ‘grafting to’. Also, additional experiments replicating the reaction conditions used for this transformation, where the cellulose was omitted, showed no self-polymerization of the ε-caprolactone (see ESI†). Speculatively, the DG might be limited by the hydrophilicity of the used SB-IL and/or the absence of reactive radical initiators.^43^

**Table 6:** Substrate scope of cyclic esters^a^

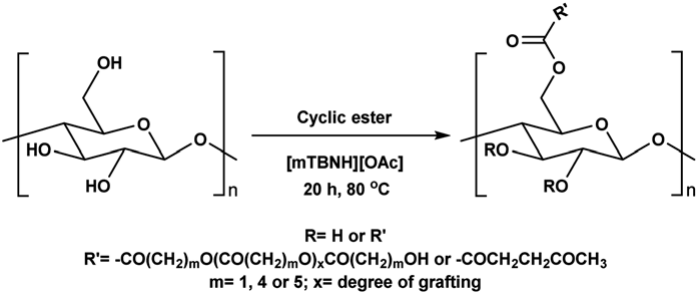		
Entry	Cyclic ester	DS/DG^b^
1^c^	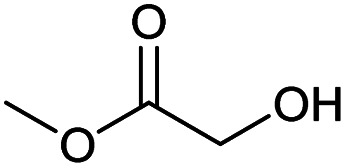	0.05/1.57
2	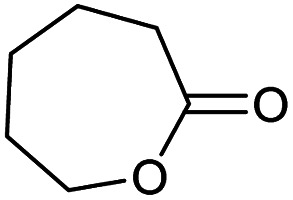	0.24/2.29
3	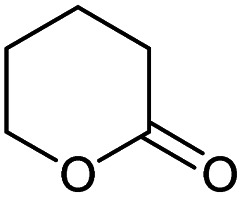	0.04/2.01
4	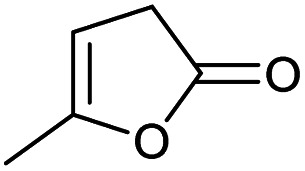	0.01/—
5	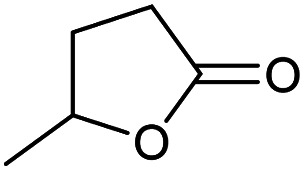	Unreactive
6	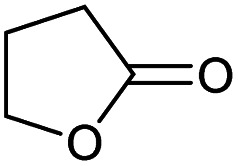	Unreactive
7	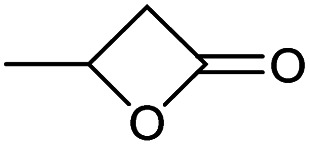	Unreactive

a Reactions were performed in 50 ml round bottom flask, where 3 equivalents of the cyclic ester were added to 250 mg of MCC dissolved in 5 ml of [mTBNH][OAc], then the reaction was stirred at 80 °C for 20 h.

b DS and DG of isolated sample determined by ^1^H and q^13^C NMR at 65 °C in 20 wt% tetrabutylphosphonium acetate in DMSO-d_6_.

c Methyl glycolate has been used for comparison of the reactivity and the grafting plausibility of open chain esters and cyclic esters.

To better understand cellulose modification with the ε-caprolactone (Table 6 entry 2), we performed series of experiments with decreased temperature (65 °C) and increasing stoichiometric increments of the reagent (Table 7). Expectedly, the DS of the isolated materials increased with increasing reagent (Table 7 entry 1–7). We detected a tenfold increase in the DS, however, observed only slight variations in the average degree of grafting. The observed variation in the DG supports our initial idea that the grafting happens after the cellulose modification, and not before. Upon careful review of the obtained data, a clear trend of decrease in the DG can be seen (Table 7 entry 1–4), after which the DG starts to increase again (Table 7 entry 5–6). Additionally, as expected the decrease of the temperature influences the DS and the DG for this reaction. A quick comparison between the reactions performed at 80 °C and 65 °C reveals that the DS has dropped to 0.10 from 0.24 (Table 7 entry 6 *vs.*Table 6 entry 2). The observed average DG decreased similarly from 2.29 to 1.76, as the reaction temperature was decreased.

**Table 7:** Transesterification with ε-caprolactone^a^

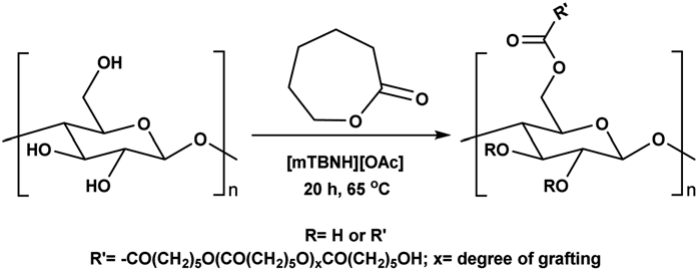		
Entry	Equivalents of reagent to AGU	DS/DG^b^
1	0.5	0.01/1.84
2	1	0.03/1.64
3	1.5	0.05/1.56
4	2	0.07/1.53
5	2.5	0.07/1.58
6	3	0.10/1.76

a Reactions were performed in 50 ml round bottom flask, where *x* equivalents of the ε-caprolactone were added to 250 mg of MCC dissolved in 5 ml of [mTBNH][OAc], then the reaction was stirred at 65 °C for 20 h.

b DS and DG of isolated sample determined by ^1^H and q^13^C NMR at 65 °C in 20 w.% tetrabutylphosphonium acetate in DMSO-d_6_.

## Solubility tests

Lead by our interest in the properties of the prepared cellulosic materials, we studied their dissolution in five different solvents. We chose water (H_2_O), dimethyl sulfoxide (DMSO), dimethyl acetamide (DMA), gamma-valerolactone (γ-VL) and cyrene™ as the model solvents for the dissolution tests (Table 8). γ-VL and cyrene™ were selected as promising green alternatives to the classical dipolar aprotic solvents.^44^ Cellulose acetates (Table 8 entry 1) with DS values lower than 0.09 did not dissolve in any of the solvents, while CAs with DS values between 0.09 and 0.32 dissolved in DMSO. In the DS range of 0.59 to 1.07 the CAs we prepared were soluble in all of the used solvents except for γ-VL and cyrene™. Upon further increase in the DS (1.29–1.75), the prepared CAs become insoluble in water, but they remained soluble in DMSO and DMA. Also, the CAs samples at this DS range were soluble in γ-VL and cyrene™. The other aliphatic cellulose esters *e.g.*, propionate, butyrate, valerate (Table 8 entry 2–4) were insoluble in any of the used solvents. Seemingly, the low DS of these esters combined with their growing hydrophobicity (alkyl chain growth) does not permit their solubility into the used solvents. The cellulosic materials prepared from the aromatic and conjugated unactivated esters were not soluble in any of the used solvents (Table 8 entry 5, 6 and 9). Surprisingly, the exception from this was cellulose *p*-nitrobenzoate (Table 8 entry 8), which was soluble in DMSO, even at low DS (0.03 and 0.25). The sample with DS of 0.75 was soluble not only in DMSO, but also in DMA. Speculatively, the enhanced solubility of this material could originate from the mesomeric effect of the *p*-nitrobenzoate substituent, which could enhance the solvent interactions. Cellulose glycolate and 5-hydroxypentanoate were soluble in DMSO at DS 0.05 and 0.04 respectively, but they were not soluble in either H_2_O, DMA, γ-VL or cyrene™ (Table 8 entry 10 and 11). The cellulosic material obtained from the reaction with the α-angelica lactone did not dissolve at all in any of the used solvents (Table 8 entry 12). The cellulose 6-hydroxyhexanoate (Table 8 entry 7) with the lowest DS of 0.01 and DG of 1.84 was not soluble in any of the solvents in use. The materials from the same series with DS between 0.03 and 0.10 were soluble in DMSO, but not in H_2_O, DMA, γ-VL or cyrene™. The highest substituted material from this series, with DS 0.24, was not soluble in H_2_O, γ-VL or cyrene™ but was soluble in both DMSO and DMA. Evidently, the samples possessing minimal DG (Table 8 entry 7,10–11) present better solubility properties, than their short chain analogues (Table 8 entry 2–4, 12). The differences might be originating from the increased solvent interactions with the grafted aliphatic chains in these materials.

**Table 8:** Dissolution test of prepared cellulosic materials^a^^,^^b^

Entry	Cellulose derivate	DS/DG	H_2_O/DMSO/DMA/γ-VL/Cyrene™	Entry	Cellulose derivate	DS/DG	H_2_O/DMSO/DMA/γ-VL/Cyrene™
1	Acetate	0.07	−/−/−/−/−	7	6-Hydroxyhexanoate		
		0.09	−/+^c^/−/−/−				
		0.11	−/+^c^/−/−/−				
		0.13	−/+^c^/−/−/−			0.01/1.84	−/−/−/−/−
		0.17	−/+^d^/−/−/−			0.03/1.64	−/+^c^/−/−/−
		0.22	−/+/−/−/−			0.05/1.56	−/+^d^/−/−/−
		0.32	−/+/−/−/−			0.07/1.53	−/+/−/−/−
		0.59	+/+/+/−/−			0.07/1.58	−/+/−/−/−
		0.72	+/+/+/−/−			0.10/1.76	−/+/−/−/−
		1.07	+/+/+/−/−			0.24/2.29	−/+/+/−/−
		1.29	−/+/+/+^c^/+^c^				
		1.44	−/+/+/+/+				
		1.75	−/+/+/+/+				
2	Propionate	0.06	−/−/−/−/−	8	4-Nitrobenzoate	0.03	−/+^d^/−/−/−
		0.08	−/−/−/−/−			0.25	−/+/−/−/−
		0.10	−/−/−/−/−			0.75	−/+/+/−/−
3	Butyrate	0.04	−/−/−/−/−	9	Cinnamate	0.03	−/−/−/−/−
		0.05	−/−/−/−/−			0.07	−/−/−/−/−
4	Valerate	0.04	−/−/−/−/−	10	Glycolate	0.05/1.57	−/+^d^/−/−/−
		0.05	−/−/−/−/−				
		0.06	−/−/−/−/−				
5	Benzoate	0.02	−/−/−/−/−	11	5-Hydroxypentanoate	0.04/2.01	−/+^d^/−/−/−
		0.05	−/−/−/−/−				
6	4-Methoxybenzoate	0.005	−/−/−/−/−	12	4-Oxopentanoate	0.01	−/−/−/−/−
		0.01	−/−/−/−/−				

a 10 mg of each cellulosic material was suspended in 1 ml of the corresponding solvent. After the suspensions were stirred at 100 °C for 20 h, the dissolution was confirmed.

b A chart depicting the structures of the prepared cellulosic materials could be found in the ESI.

c Solutions clarify at 100 °C, but partially precipitate upon cooling.

d Optical microscope images of the dissolved samples could be found in the ESI associated with this article.

## Experimental

Description of the materials, the analytical methods, and the devices used as well as the general and the detailed synthetic procedures are provided in the ESI† associated with this article along with the accompanying spectroscopic data.

## Conclusions

In summary, we have developed a sustainable cellulose transesterification protocol, which employs the superbase ionic liquid [mTBNH][OAc] not only as the reaction media, but also shows significant catalytic activity. This dual role of the SB-IL in the developed protocol allowed us to use unactivated methyl esters as the acyl donors for this transformation. The excellent recyclability and activation properties of the used SB-IL makes it an attractive choice for cellulose transformations. The developed conditions allowed us also to expand the already broad scope of the acyl donors with activated and cyclic esters. Careful reaction screening provided us with multiple possibilities for preparation of cellulosic materials with different substitution and degree of substitution. Additionally, we observed clear trends in the dissolution behaviour of these cellulose materials according to the attached substituent to them, with even very low DS values.

## Author contributions

A. R. T. planned the work, performed the experiments, characterized the prepared materials, and wrote the manuscript. A. W. T. K. contributed to the planning of the work, and the characterization of some of the prepared materials. I. K. conceived the project, and provided overall project guidance, and edited the manuscript together with A. R. T. and A. W. T. K.

## Conflicts of interest

There are no conflicts of interest to declare.

## Supplementary Material

RA-013-D2RA08186E-s001
