# Supplementary material for: Transesterification of cellulose with unactivated esters in superbase–acid conjugate ionic liquids
Source: RSC Adv. 2023 Feb 17;13(9):5983–92. doi: 10.1039/d2ra08186e (PMC9936960; doi:10.1039/d2ra08186e)
Supplement: RA-013-D2RA08186E-s001 [file RA-013-D2RA08186E-s001.pdf]

Electronic Supplementary Material (ESI) for RSC Advances.
This journal is © The Royal Society of Chemistry 2023

Electronic Supplementary Information

Transesterification of cellulose with unactivated esters in superbase ionic liquids

Aleksandar R. Todorov,*[a] Alistair W. T. King[a,b] and Ilkka Kilpeläinen[a]

[a] Materials Chemistry Division, Department of Chemistry, University of Helsinki, FI-00560 Helsinki, Finland. [b] VTT Technical Research Centre of Finland Ltd, Tietotie 4e, 02150 Espoo, Finland.

Corresponding author: aleksandar.todorov@helsinki.fi

Contents

General information 3
Determination of the degree of substitution – isolated samples 3
Determination of the degree of substitution – reaction optimization samples 4
Optimization studies 4
Reagent 5
Reagent amount 5
Reaction time 6
Reaction temperature 6
Ionic liquid composition 6
Miscellaneous 7
Reaction efficiency studies 8
Recyclability studies 9
Enhancement experiments 9
Miscellaneous experiments 10
Gel permission chromatography (GPC) measurements 13
Optical microscopy images 13
Cellulose materials – schematic representation 16
General procedure 17
Synthesis of cellulose materials 17
Cellulose acetate 17
Cellulose propionate 18
Cellulose butyrate 18
Cellulose valerate 19
Cellulose benzoate 19
Cellulose 4-methoxybenzoate 19
Cellulose 4-nitrobenzoate 19
Cellulose cinnamate 20
Cellulose glycolate 20
Cellulose 4-oxopentanoate 20
Cellulose 5-hydroxypentanoate 20
Cellulose 6-hydroxyhexanoate 20
References 21
Copy of spectral data 22

General information

The general experimental procedures, the specific details for representative reactions, the spectroscopic information for the prepared cellulosic materials, and as well the equipment used for their characterisation have been presented below. All commercial chemicals were used as received. The model microcrystalline cellulose (MCC) Avicel® PH-101 was purchased from Sigma Aldrich. The methyl esters used in this study were acquired either from Sigma Aldrich or TCI Europe. The used ionic liquids were obtained from Liuotin group Oy (https://liuotin.com/). Liquid state ^{1}H, diffusion-edited ^{1}H, and ^{13}C{^{1}H} NMR spectra were recorded at 25 or 65 °C using Bruker Avance Neo 400 [400.15 MHz], Bruker Avance Neo 500 [499.83 MHz], and Bruker Avance Neo 600 [599.69 MHz] spectrometers. ^{1}H and ^{13}C{^{1}H} spectra were referenced to the residual solvent signals of DMSO-d_6 2.50 and 39.5 ppm, respectively with no special notation for the equivalent carbons.

Determination of the degree of substitution – isolated samples

The degree of substitution (DS) of the prepared and isolated cellulosic materials was determined from the liquid state quantitative ^{1}H NMR or ^{13}C NMR spectra at 65 °C in 20 w.% tetrabutylphosphonium acetate DMSO-d_6 electrolyte solution following scheme S1.[1,2]

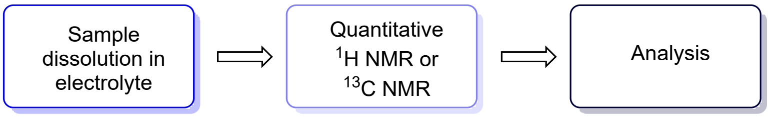

Scheme S1. DS determination of isolated samples

The NMR data was analysed following scheme S2, where the crude NMR data was firstly processed in MestReNova (phase corrected only) and then it was exported as .txt format in order to be analysed in Fityk.

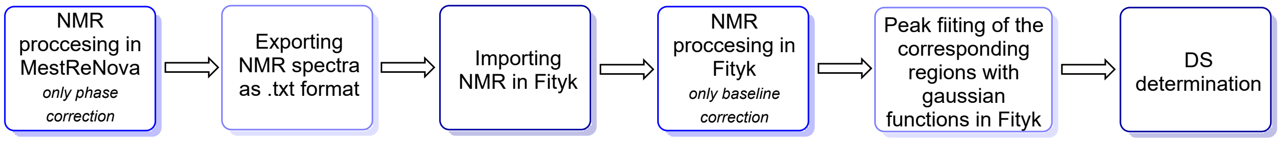

Scheme S2. Analysis of the NMR data

Once the NMR data have been imported in Fityk, it was further processed (spline baseline corrected only). After that, the cellulose region, and the corresponding substituent region were peak fitted with gaussian functions. Examples of spline baseline correction and gaussian peak fitting in Fityk could be seen in figure S1.

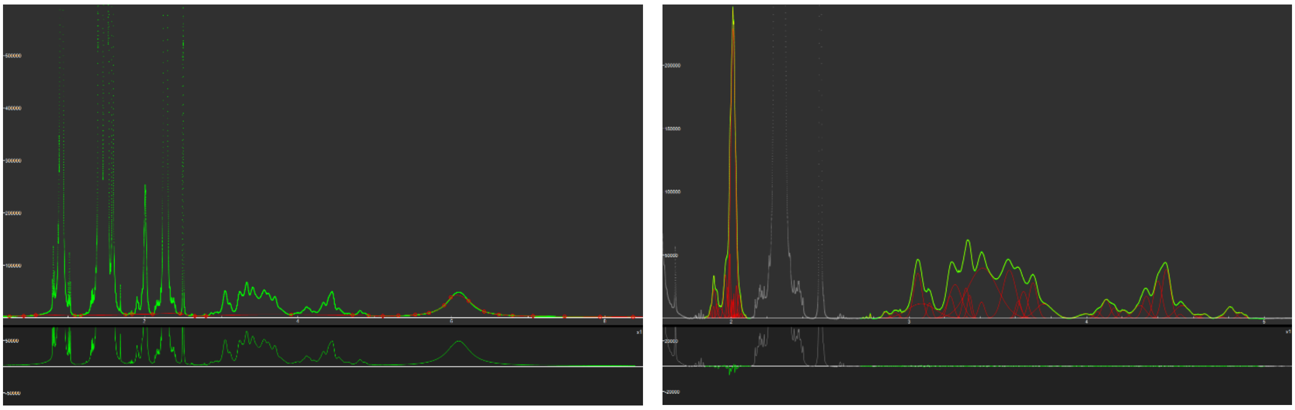

Figure S1 Example of spline baseline correction and gaussian peak fitting in Fityk for cellulose acetate with DS 0.72

After peak fitting the corresponding NMR regions, the DS was determined following equation 1

$$DS = \frac{\frac{I_{(substituent)}}{x}}{\frac{I_{(cellulose)}}{7}}$$ Eq. 1

where, $I_{(substituent)}$ is the peak area of the substituent signal, $I_{(cellulose)}$ is the peak area of the combined cellulose backbone signals, ***X*** (dependable from the substituent e.g., 3 for acetate) and ***7*** are the total number of protons for the above-mentioned fragments.

Determination of the degree of substitution – reaction optimization samples

The DS of the reaction optimization samples was determined by the diffusion-edited ^{1}H NMR at 65 °C with a standard calibration curve. The standard calibration curve was prepared from two sets of cellulose acetate samples with known DS. The diffusion-edited ^{1}H NMR of these samples were recorded in as similar as possible environmental conditions (e.g., concentration, solvents, dilutions etc.,) as the analysed samples, in order to avoid substantial errors.

Table S1. Acetylated samples with known DS used to build standard calibration curve

Number of sample	Average integral ratio (Acetyl/Cellulose)	DS
1	0.624608436	0.112
2	1.234308122	0.322
3	1.576160965	0.589
4	1.74030033	0.722
5	1.970603139	1.069
6	2.136792299	1.293

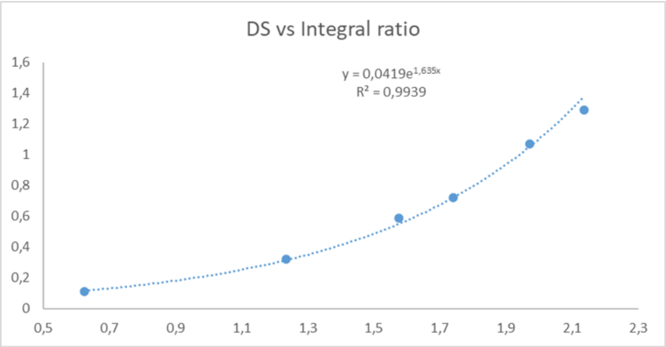

Figure S2. The standard calibration curve with exponential fit

The integral ratio between the acetyl signal (1.87-2.20 ppm) and the cellulose signal (4.00-5.50 ppm) from the diffusion-edited ^{1}H spectra was plotted against the know DS (table S1 and figure S2), thus allowing us to obtain the standard calibration curve. The curve was exponentially fitted and equation 2 was obtained, which was used further to determine the DS of the samples in the reaction optimizations series.

$$DS = 0.0419e^{1.635x}$$ Eq.2

Here in equation 2, ***X*** is the integral ratio between the acetyl signal (1.87-2.20 ppm) and the cellulose signal (4.00-5.00 ppm) from the diffusion-edited ^{1}H spectra. Upon solving equation 2, the reported DS values for the reaction optimization were obtained. The reported DS values in table 1 and 2 are average of two independent reactions performed in the same conditions. The shown method for determination of the DS works well for cellulose acetate samples and in the calibrated region. The lower detection limit of the described method was found to be DS of 0.043 or integral ratio of 0.01. Upon cross validation, comparable results were obtained when the DS was determined from the isolated cellulose acetate samples through liquid state quantitative ^{1}H NMR.

Optimization studies

Microcrystalline cellulose (0.0500 g, 0.3084 mmol) was placed into 8 mL vial. Then a cylindrical stirrer bar (10 x 6 mm) was added followed by 1 mL [mTBNH][OAc] and the vial was sealed. The viscous reaction mixture was suspended with vortex genie 2 (scientific industries) and then it was immersed into a preheated to the desired reaction temperature (typical 80 °C) silicon bath. The suspended MCC was stirred at this temperature until complete dissolution occurs (typically 30 minutes). After that the vial was taken out from the silicon bath and the desired amount of the used reagent was added to the dissolved MCC. Then the vial was resealed, and the reaction mixture was immersed again into the silicon bath, and it was stirred for the corresponding time (typically 20 h). After that the reaction mixture was diluted with 1 mL of DMSO-d_6 and was analysed by diffusion-edited ^{1}H NMR. Then the DS was determined from the crude reaction mixtures using the standard calibration curve and the results can be found in table 1 and 2.

Reagent

The titled parameter was varied following the optimization studies procedure. Results are average of two independent reactions performed in the same conditions.

Methyl acetate: Starting from MCC (0.0500 g, 0.3084 mmol) and MeOAc (0.0737 mL, 0.9252 mmol). Reaction time was 20 h and the temperature was set at 80 °C. **Obtained DS: 0.08**

Ethyl acetate: Starting from MCC (0.0500 g, 0.3084 mmol) and EtOAc (0.0904 mL, 0.9252 mmol). Reaction time was 20 h and the temperature was set at 80 °C. **Obtained DS: 0.05**

***i*-Propyl acetate:** Starting from MCC (0.0500 g, 0.3084 mmol) and *i*-PrOAc (0.1086 mL, 0.9252 mmol). Reaction time was 20 h and the temperature was set at 80 °C. **Obtained DS: 0.05**

***t*-Butyl acetate:** Starting from MCC (0.0500 g, 0.3084 mmol) and *t*-BuOAc (0.1251 mL, 0.9252 mmol). Reaction time was 20 h and the temperature was set at 80 °C. **Obtained DS: 0.04**

Reagent amount

The titled parameter was varied following the optimization studies procedure. Results are average of two independent reactions performed in the same conditions.

Methyl acetate – 1 equivalent to AGU: Starting from MCC (0.0500 g, 0.3084 mmol) and MeOAc (0.0246 mL, 0.3084 mmol). Reaction time was 20 h and the temperature was set at 80 °C. **Obtained DS: 0.05**

Methyl acetate – 2 equivalents to AGU: Starting from MCC (0.0500 g, 0.3084 mmol) and MeOAc (0.0491 mL, 0.6168 mmol). Reaction time was 20 h and the temperature was set at 80 °C. **Obtained DS: 0.07**

Methyl acetate – 3 equivalents to AGU: Starting from MCC (0.0500 g, 0.3084 mmol) and MeOAc (0.0737 mL, 0.9252 mmol). Reaction time was 20 h and the temperature was set at 80 °C. **Obtained DS: 0.08**

Methyl acetate – 4 equivalents to AGU: Starting from MCC (0.0500 g, 0.3084 mmol) and MeOAc (0.0983 mL, 1.2336 mmol). Reaction time was 20 h and the temperature was set at 80 °C. **Obtained DS: 0.09**

Methyl acetate – 5 equivalents to AGU: Starting from MCC (0.0500 g, 0.3084 mmol) and MeOAc (0.1228 mL, 1.5420 mmol). Reaction time was 20 h and the temperature was set at 80 °C. **Obtained DS: 0.10**

Methyl acetate – 6 equivalents to AGU: Starting from MCC (0.0500 g, 0.3084 mmol) and MeOAc (0.1474 mL, 1.8504 mmol). Reaction time was 20 h and the temperature was set at 80 °C. **Obtained DS: 0.12**

Methyl acetate – 9 equivalents to AGU: Starting from MCC (0.0500 g, 0.3084 mmol) and MeOAc (0.2211 mL, 2.7756 mmol). Reaction time was 20 h and the temperature was set at 80 °C. **Obtained DS: 0.15**

Methyl acetate – 12 equivalents to AGU: Starting from MCC (0.0500 g, 0.3084 mmol) and MeOAc (0.2948 mL, 3.7008 mmol). Reaction time was 20 h and the temperature was set at 80 °C. **Obtained DS: 0.19**

Methyl acetate – 15 equivalents to AGU: Starting from MCC (0.0500 g, 0.3084 mmol) and MeOAc (0.3685 mL, 4.6260 mmol). Reaction time was 20 h and the temperature was set at 80 °C. **Obtained DS: 0.21**

Reaction time

The titled parameter was varied following the optimization studies procedure. Results are average of two independent reactions performed in the same conditions.

Methyl acetate – 24 h: Starting from MCC (0.0500 g, 0.3084 mmol) and MeOAc (0.0737 mL, 0.9252 mmol). Reaction time was 24 h and the temperature was set at 80 °C. **Obtained DS: 0.09**

Methyl acetate – 48 h: Starting from MCC (0.0500 g, 0.3084 mmol) and MeOAc (0.0737 mL, 0.9252 mmol). Reaction time was 48 h and the temperature was set at 80 °C. **Obtained DS: 0.15**

Methyl acetate – 72 h: Starting from MCC (0.0500 g, 0.3084 mmol) and MeOAc (0.0737 mL, 0.9252 mmol). Reaction time was 72 h and the temperature was set at 80 °C. **Obtained DS: 0.19**

Reaction temperature

The titled parameter was varied following the optimization studies procedure. Results are average of two independent reactions performed in the same conditions.

Methyl acetate – 65 °C: Starting from MCC (0.0500 g, 0.3084 mmol) and MeOAc (0.0737 mL, 0.9252 mmol). Reaction time was 20 h and the temperature was set at 65 °C. **Obtained DS: 0.05**

Methyl acetate – 80 °C: Starting from MCC (0.0500 g, 0.3084 mmol) and MeOAc (0.0737 mL, 0.9252 mmol). Reaction time was 20 h and the temperature was set at 80 °C. **Obtained DS: 0.08**

Methyl acetate – 100 °C: Starting from MCC (0.0500 g, 0.3084 mmol) and MeOAc (0.0737 mL, 0.9252 mmol). Reaction time was 20 h and the temperature was set at 100 °C. **Obtained DS: 0.18**

Ionic liquid composition

The titled parameter was varied following the optimization studies procedure. Results are average of two independent reactions performed in the same conditions.

Methyl acetate – [mTBNH][OAc] [1:1.5]: Starting from MCC (0.0500 g, 0.3084 mmol) and MeOAc (0.0737 mL, 0.9252 mmol). Reaction time was 20 h and the temperature was set at 80 °C. **Obtained DS: 0.06**

Methyl acetate – [mTBNH][OAc] [1:1.25]: Starting from MCC (0.0500 g, 0.3084 mmol) and MeOAc (0.0737 mL, 0.9252 mmol). Reaction time was 20 h and the temperature was set at 80 °C. **Obtained DS: 0.07**

Methyl acetate – [mTBNH][OAc] [1:1]: Starting from MCC (0.0500 g, 0.3084 mmol) and MeOAc (0.0737 mL, 0.9252 mmol). Reaction time was 20 h and the temperature was set at 80 °C. **Obtained DS: 0.08**

Methyl acetate – [mTBNH][OAc] [1.25:1]: Starting from MCC (0.0500 g, 0.3084 mmol) and MeOAc (0.0737 mL, 0.9252 mmol). Reaction time was 20 h and the temperature was set at 80 °C. **Obtained DS: 0.09**

Methyl acetate – [mTBNH][OAc] [1.5:1]: Starting from MCC (0.0500 g, 0.3084 mmol) and MeOAc (0.0737 mL, 0.9252 mmol). Reaction time was 20 h and the temperature was set at 80 °C. **Obtained DS: 0.10**

Methyl acetate – [mTBNH][OAc] [1.75:1]: Starting from MCC (0.0500 g, 0.3084 mmol) and MeOAc (0.0737 mL, 0.9252 mmol). Reaction time was 20 h and the temperature was set at 80 °C. **Obtained DS: 0.12**

Methyl acetate – [mTBNH][OAc] [2:1]: Starting from MCC (0.0500 g, 0.3084 mmol) and MeOAc (0.0737 mL, 0.9252 mmol). Reaction time was 20 h and the temperature was set at 80 °C. **Obtained DS: 0.11**

Methyl acetate – [mTBNH][OAc] [3:1]: Starting from MCC (0.0500 g, 0.3084 mmol) and MeOAc (0.0737 mL, 0.9252 mmol). Reaction time was 20 h and the temperature was set at 80 °C. **Obtained DS: 0.14**

Methyl acetate – [mTBNH][OAc] [4:1]: Starting from MCC (0.0500 g, 0.3084 mmol) and MeOAc (0.0737 mL, 0.9252 mmol). Reaction time was 20 h and the temperature was set at 80 °C. **Obtained DS: 0.13**

Methyl acetate – [mTBNH][OAc] [5:1]: Starting from MCC (0.0500 g, 0.3084 mmol) and MeOAc (0.0737 mL, 0.9252 mmol). Reaction time was 20 h and the temperature was set at 80 °C. **Obtained DS: 0.14**

Methyl acetate – [mTBNH][OAc] [10:1]: Starting from MCC (0.0500 g, 0.3084 mmol) and MeOAc (0.0737 mL, 0.9252 mmol). Reaction time was 20 h and the temperature was set at 80 °C. **Obtained DS: 0.16**

Miscellaneous

Blank reactions in various ionic liquid compositions were performed following the optimization studies procedure. Results are average of two independent reactions performed in the same conditions.

[mTBNH][OAc] [1:1.5]: Starting from MCC (0.0500 g, 0.3084 mmol). Reaction time was 20 h and the temperature was set at 80 °C. **Obtained DS of acetylation: under the method detection limit**

[mTBNH][OAc] [1:1.25]: Starting from MCC (0.0500 g, 0.3084 mmol). Reaction time was 20 h and the temperature was set at 80 °C. **Obtained DS of acetylation: under the method detection limit**

[mTBNH][OAc] [1:1]: Starting from MCC (0.0500 g, 0.3084 mmol). Reaction time was 20 h and the temperature was set at 80 °C. **Obtained DS of acetylation: under the method detection limit**

[mTBNH][OAc] [1.25:1]: Starting from MCC (0.0500 g, 0.3084 mmol). Reaction time was 20 h and the temperature was set at 80 °C. **Obtained DS of acetylation: under the method detection limit**

[mTBNH][OAc] [1.5:1]: Starting from MCC (0.0500 g, 0.3084 mmol). Reaction time was 20 h and the temperature was set at 80 °C. **Obtained DS of acetylation: under the method detection limit**

[mTBNH][OAc] [1.75:1]: Starting from MCC (0.0500 g, 0.3084 mmol). Reaction time was 20 h and the temperature was set at 80 °C. **Obtained DS of acetylation: under the method detection limit**

[mTBNH][OAc] [2:1]: Starting from MCC (0.0500 g, 0.3084 mmol). Reaction time was 20 h and the temperature was set at 80 °C. **Obtained DS of acetylation: under the method detection limit**

[mTBNH][OAc] [3:1]: Starting from MCC (0.0500 g, 0.3084 mmol). Reaction time was 20 h and the temperature was set at 80 °C. **Obtained DS of acetylation: under the method detection limit**

[mTBNH][OAc] [4:1]: Starting from MCC (0.0500 g, 0.3084 mmol). Reaction time was 20 h and the temperature was set at 80 °C. **Obtained DS of acetylation: under the method detection limit**

[mTBNH][OAc] [5:1]: Starting from MCC (0.0500 g, 0.3084 mmol). Reaction time was 20 h and the temperature was set at 80 °C. **Obtained DS of acetylation: under the method detection limit**

[mTBNH][OAc] [10:1]: Starting from MCC (0.0500 g, 0.3084 mmol). Reaction time was 20 h and the temperature was set at 80 °C. **Obtained DS of acetylation: under the method detection limit**

Reactions with methyl propionate in various ionic liquid compositions were performed following the optimization studies procedure. Results are average of two independent reactions performed in the same conditions.

Methyl propionate – [mTBNH][OAc] [1:1.5]: Starting from MCC (0.0500 g, 0.3084 mmol) and MeOPr (0.0891 mL, 0.9252 mmol). Reaction time was 20 h and the temperature was set at 80 °C. **Obtained DS of acetylation: under the method detection limit**

Methyl propionate – [mTBNH][OAc] [1:1]: Starting from MCC (0.0500 g, 0.3084 mmol) and MeOPr (0.0891 mL, 0.9252 mmol). Reaction time was 20 h and the temperature was set at 80 °C. **Obtained DS of acetylation: under the method detection limit**

Methyl propionate – [mTBNH][OAc] [1.5:1]: Starting from MCC (0.0500 g, 0.3084 mmol) and MeOPr (0.0891 mL, 0.9252 mmol). Reaction time was 20 h and the temperature was set at 80 °C. **Obtained DS of acetylation: under the method detection limit**

Methyl propionate – [mTBNH][OAc] [2:1]: Starting from MCC (0.0500 g, 0.3084 mmol) and MeOPr (0.0891 mL, 0.9252 mmol). Reaction time was 20 h and the temperature was set at 80 °C. **Obtained DS of acetylation: under the method detection limit**

Methyl propionate – [mTBNH][OAc] [5:1]: Starting from MCC (0.0500 g, 0.3084 mmol) and MeOPr (0.0891 mL, 0.9252 mmol). Reaction time was 20 h and the temperature was set at 80 °C. **Obtained DS of acetylation: under the method detection limit**

Methyl propionate – [mTBNH][OAc] [10:1]: Starting from MCC (0.0500 g, 0.3084 mmol) and MeOPr (0.0891 mL, 0.9252 mmol). Reaction time was 20 h and the temperature was set at 80 °C. **Obtained DS of acetylation: under the method detection limit**

Reaction efficiency studies

Microcrystalline cellulose (0.0500 g, 0.3084 mmol) was placed into 8 mL vial. Then a cylindrical stirrer bar (10 x 6 mm) was added followed by 1 mL [mTBNH][OAc] and the vial was sealed. The viscous reaction mixture was suspended with vortex genie 2 (scientific industries) and then it was immersed into a preheated to 80 °C silicon bath. After 30 minutes of stirring the MCC was completely dissolved, and the vial was removed from the silicon bath. After cooling the reaction mixture, the desired amount of acetylating reagent was added followed by 1 mL of DMSO-d_6. After that, 1 mL from the well stirred reaction mixture was transferred to an NMR tube. Then the NMR tube was placed into the preheated to the desired temperature NMR spectrometer and was subject of NMR analysis. The diffusion-edited ^{1}H data was collected every 30 minutes for 24 h resulting in 48 individual points. The DS was determined using the standard calibration curve.

Methyl acetate – 3 equivalents to AGU at 65 °C: Starting from MCC (0.0500 g, 0.3084 mmol) and MeOAc (0.0737 mL, 0.9252 mmol), following the above-mentioned procedure. Reaction time was 24 h and the NMR temperature was set at 65 °C.

Methyl acetate – 3 equivalents to AGU at 80 °C: Starting from MCC (0.0500 g, 0.3084 mmol) and MeOAc (0.0737 mL, 0.9252 mmol), following the above-mentioned procedure. Reaction time was 24 h and the NMR temperature was set at 80 °C.

***i*-Propenyl acetate – 0.5 equivalents to AGU at 65 °C:** Starting from MCC (0.0500 g, 0.3084 mmol) and *i*-PropenylOAc (0.0170 mL, 0.1542 mmol), following the above-mentioned procedure. Reaction time was 24 h and the NMR temperature was set at 65 °C.

***i*-Propenyl acetate – 0.5 equivalents to AGU at 80 °C:** Starting from MCC (0.0500 g, 0.3084 mmol) and *i*-PropenylOAc (0.0170 mL, 0.1542 mmol), following the above-mentioned procedure. Reaction time was 24 h and the NMR temperature was set at 80 °C.

Recyclability studies

Ionic liquid recyclability: A 100 mL round bottom flask was charged with microcrystalline cellulose (0.5000 g, 3.0838 mmol), followed by an oval stirrer bar (30 x 15 mm) and 10 mL (11.5475 g) [mTBNH][OAc]. The viscous reaction mixture was suspended with vortex genie 2 (scientific industries) and then it was immersed into a preheated to 65 °C silicon bath. After 30 minutes of stirring the MCC was completely dissolved, and 2 equivalents of *i*-propenyl acetate (0.6793 mL, 6.1676 mmol) were added to the reaction mixture. Then the reaction was stirred for 20 h furthermore at 65 °C before it was quenched. The reaction mixture was diluted with 90 mL EtOH, while it is still hot. Upon cooling, the already diluted reaction mixture was further suspended into 510 mL EtOH, and it was allowed to stir overnight at room temperature. After that the formed cellulosic solids were filtrated off and were washed (x3 times) with EtOH. After the filtration the obtained cellulose material was freeze dried and was subject of data analysis (DS, dissolution, etc.,). The ethanol washings containing the used ionic liquid were combined and evaporated (rotary evaporator Buchi® R-210 with vacuum controller V-855 and vacuum pump V-710) after each reaction cycle. The dried [mTBNH][OAc] was reused in six consecutive cycles.

Cycle 1: **Obtained DS: 1.05; Recovered solvent: 11.4320 g, 99.0 %**

Cycle 2: **Obtained DS: 0.56; Recovered solvent: 11.4040 g, 98.8 %**

Cycle 3: **Obtained DS: 1.09; Recovered solvent: 11.3166 g, 98.0 %**

Cycle 4: **Obtained DS: 0.75; Recovered solvent: 11.2935 g, 97.8 %**

Cycle 5: **Obtained DS: 0.72; Recovered solvent: 11.2010 g, 97.0 %**

Cycle 6: **Obtained DS: 0.71; Recovered solvent: 11.1401 g, 96.5 %**

Cellulose recovery solvent retrieval: A 50 mL round bottom flask was charged with microcrystalline cellulose (0.2500 g, 1.5419 mmol), followed by an oval stirrer bar (20 x 10 mm) and 5 mL [mTBNH][OAc]. The viscous reaction mixture was suspended with vortex genie 2 (scientific industries) and then it was immersed into a preheated to 80 °C silicon bath. After 30 minutes of stirring the MCC was completely dissolved, and 3 equivalents of MeOAc (0.3685 mL, 4.6257 mmol) were added to the reaction mixture. Then the reaction mixture was stirred for 20 h furthermore at 80 °C before it was quenched. The reaction mixture was diluted with 50 mL (38.21 g) EtOH, while it was still hot. After that the formed cellulosic solids were filtrated off and were washed (x3 times) with 20 mL (14.84 g, 14.55 g, 15.66 g) EtOH. After the filtration the obtained cellulose material was freeze dried and was subject of data analysis (DS, dissolution, etc.,). The combined ethanol washings were fractionally distilled at atmospheric pressure with regular distillation apparatus. **Recovered ethanol: 82.43 g, 99.0 %**

Enhancement experiments

Experiment 1: A 50 mL round bottom flask was charged with microcrystalline cellulose (0.2500 g, 1.5419 mmol), followed by an oval stirrer bar (20 x 10 mm) and 5 mL [mTBNH][OAc]. The viscous reaction mixture was suspended with vortex genie 2 (scientific industries) and then it was immersed into a preheated to 80 °C silicon bath. After 30 minutes of stirring the MCC was completely dissolved, and 3 equivalents of MeOAc (0.3685 mL, 4.6257 mmol) were added to the reaction mixture. Then the reaction mixture was stirred for 20 h furthermore at 80 °C before it was quenched. The reaction mixture was diluted with 45 mL EtOH while it was still hot. Upon cooling, the already diluted reaction mixture was further suspended into 255 mL EtOH, and it was allowed to stir overnight at room temperature. After that the formed cellulosic solids were filtrated off and were washed (x3 times) with EtOH. After the filtration, the obtained cellulose material was freeze dried and was subject of data analysis (DS, dissolution, etc.,). **Obtained DS: 0.07**

Experiment 2: A 25 mL round bottom flask was charged with cellulose acetate with DS of 0.07 (0.1000 g, 0.6168 mmol), followed by an oval stirrer bar (20 x 10 mm) and 2 mL [mTBNH][OAc]. The viscous reaction mixture was suspended with vortex genie 2 (scientific industries) and then it was immersed into a preheated to 80 °C silicon bath. After 30 minutes of stirring the MCC was completely dissolved, and 3 equivalents of MeOAc (0.1474 mL, 1.8504 mmol) were added to the reaction mixture. Then the reaction mixture was stirred for 20 h furthermore at 80 °C before it was quenched. The reaction mixture was diluted with 20 mL EtOH while it was still hot. Upon cooling, the already diluted reaction mixture was further suspended into 130 mL EtOH, and it was allowed to stir overnight at room temperature. After that the formed cellulosic solids were filtrated off and were washed (x3 times) with EtOH. After the filtration, the obtained cellulose material was freeze dried and was subject of data analysis (DS, dissolution, etc.,). **Obtained DS: 0.12**

Experiment 3: A 25 mL round bottom flask was charged with cellulose acetate with DS of 0.12 (0.0500 g, 0.3084 mmol), followed by an oval stirrer bar (20 x 10 mm) and 1 mL [mTBNH][OAc]. The viscous reaction mixture was suspended with vortex genie 2 (scientific industries) and then it was immersed into a preheated to 80 °C silicon bath. After 30 minutes of stirring the MCC was completely dissolved, and 3 equivalents of MeOAc (0.0737 mL, 0.9252 mmol) were added to the reaction mixture. Then the reaction mixture was stirred for 20 h furthermore at 80 °C before it was quenched. The reaction mixture was diluted with 20 mL EtOH while it was still hot. Upon cooling, the already diluted reaction mixture was further suspended into 130 mL EtOH, and it was allowed to stir overnight at room temperature. After that the formed cellulosic solids were filtrated off and were washed (x3 times) with EtOH. After the filtration, the obtained cellulose material was freeze dried and was subject of data analysis (DS, dissolution, etc.,). **Obtained DS: 0.21**

Miscellaneous experiments

Competitive reactions with ethanol

Experiment 1 – 1 v.v. % added EtOH: A 100 mL round bottom flask was charged with microcrystalline cellulose (0.5000 g, 3.0838 mmol), followed by an oval stirrer bar (30 x 15 mm) and a mixture of 10 mL [mTBNH][OAc] and 0.1 mL of EtOH (1.7126 mmol). The viscous reaction mixture was suspended with vortex genie 2 (scientific industries) and then it was immersed into a preheated to 65 °C silicon bath. After 30 minutes of stirring the MCC was completely dissolved, and 2 equivalents of *i*-propenyl acetate (0.6793 mL, 6.1676 mmol) were added to the reaction mixture. Then the reaction was stirred for 20 h furthermore at 65 °C before it was quenched. The reaction mixture was diluted with 90 mL EtOH, while it is still hot. Upon cooling, the already diluted reaction mixture was further suspended into 510 mL EtOH, and it was allowed to stir overnight at room temperature. After that the formed cellulosic solids were filtrated off and were washed (x3 times) with EtOH. After the filtration the obtained cellulose material was freeze dried and was subject of data analysis (DS, dissolution, etc.,). **Obtained DS: 0.39**

Experiment 2 – 5 v.v. % added EtOH: A 100 mL round bottom flask was charged with microcrystalline cellulose (0.5000 g, 3.0838 mmol), followed by an oval stirrer bar (30 x 15 mm) and a mixture of 10 mL [mTBNH][OAc] and 0.5 mL of EtOH (8.5631 mmol). The viscous reaction mixture was suspended with vortex genie 2 (scientific industries) and then it was immersed into a preheated to 65 °C silicon bath. After 30 minutes of stirring the MCC was completely dissolved, and 2 equivalents of *i*-propenyl acetate (0.6793 mL, 6.1676 mmol) were added to the reaction mixture. Then the reaction was stirred for 20 h furthermore at 65 °C before it was quenched. The reaction mixture was diluted with 90 mL EtOH, while it is still hot. Upon cooling, the already diluted reaction mixture was further suspended into 510 mL EtOH, and it was allowed to stir overnight at room temperature. After that the formed cellulosic solids were filtrated off and were washed (x3 times) with EtOH. After the filtration the obtained cellulose material was freeze dried and was subject of data analysis (DS, dissolution, etc.,). **Obtained DS: 0.31**

Experiment 3 – 10 v.v. % added EtOH: A 100 mL round bottom flask was charged with microcrystalline cellulose (0.5000 g, 3.0838 mmol), followed by an oval stirrer bar (30 x 15 mm) and a mixture of 10 mL [mTBNH][OAc] and 1 mL of EtOH (17.1261 mmol). The viscous reaction mixture was suspended with vortex genie 2 (scientific industries) and then it was immersed into a preheated to 65 °C silicon bath. After 30 minutes of stirring the MCC was completely dissolved, and 2 equivalents of *i*-propenyl acetate (0.6793 mL, 6.1676 mmol) were added to the reaction mixture. Then the reaction was stirred for 20 h furthermore at 65 °C before it was quenched. The reaction mixture was diluted with 90 mL EtOH, while it is still hot. Upon cooling, the already diluted reaction mixture was further suspended into 510 mL EtOH, and it was allowed to stir overnight at room temperature. After that the formed cellulosic solids were filtrated off and were washed (x3 times) with EtOH. After the filtration the obtained cellulose material was freeze dried and was subject of data analysis (DS, dissolution, etc.,). **Obtained DS: 0.24**

***ε*-Caprolactone test reaction:** A 50 mL round bottom flask was charged with *ε*-caprolactone (0.4934 mL, 4.6257 mmol) followed by an oval stirrer bar (20 x 10 mm) and 5 mL [mTBNH][OAc]. The viscous reaction mixture was immersed into a preheated to 80 °C silicon bath and the reaction mixture was stirred for 24 h at this temperature. Aliquots of the reaction mixture were taken, and the decomposition/self-polymerization of the *ε*-caprolactone was monitored by ^{1}H NMR in DMSO-d_6. The aliquots were taken in the beginning of the reaction, then at 1 h, 3 h, 6 h and 24 h. Diffusion edited ^{1}H NMR of the first and the last samples together with the ^{1}H NMR was used to confirm the presence or the absence of the self-polymerized *ε*-caprolactone.

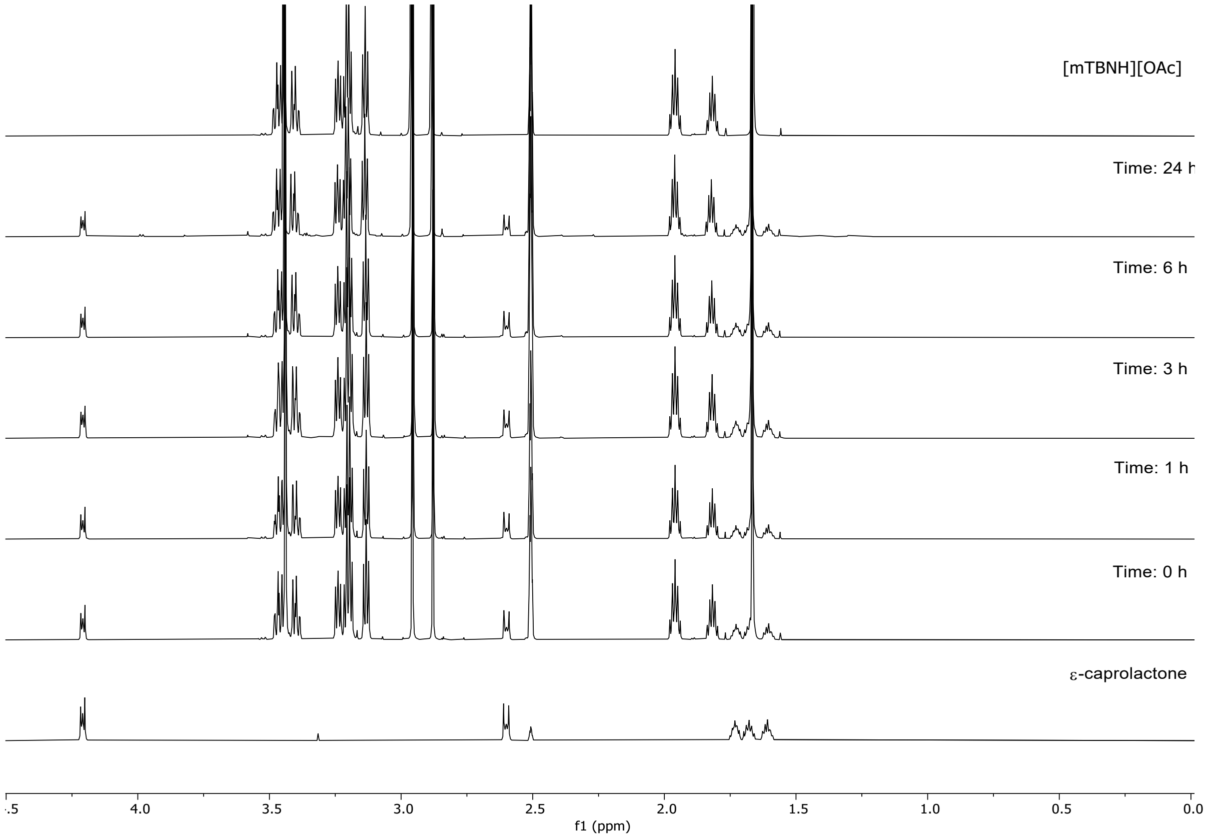

Figure S3 ^{1}H spectra of the ε-caprolactone reaction mixture

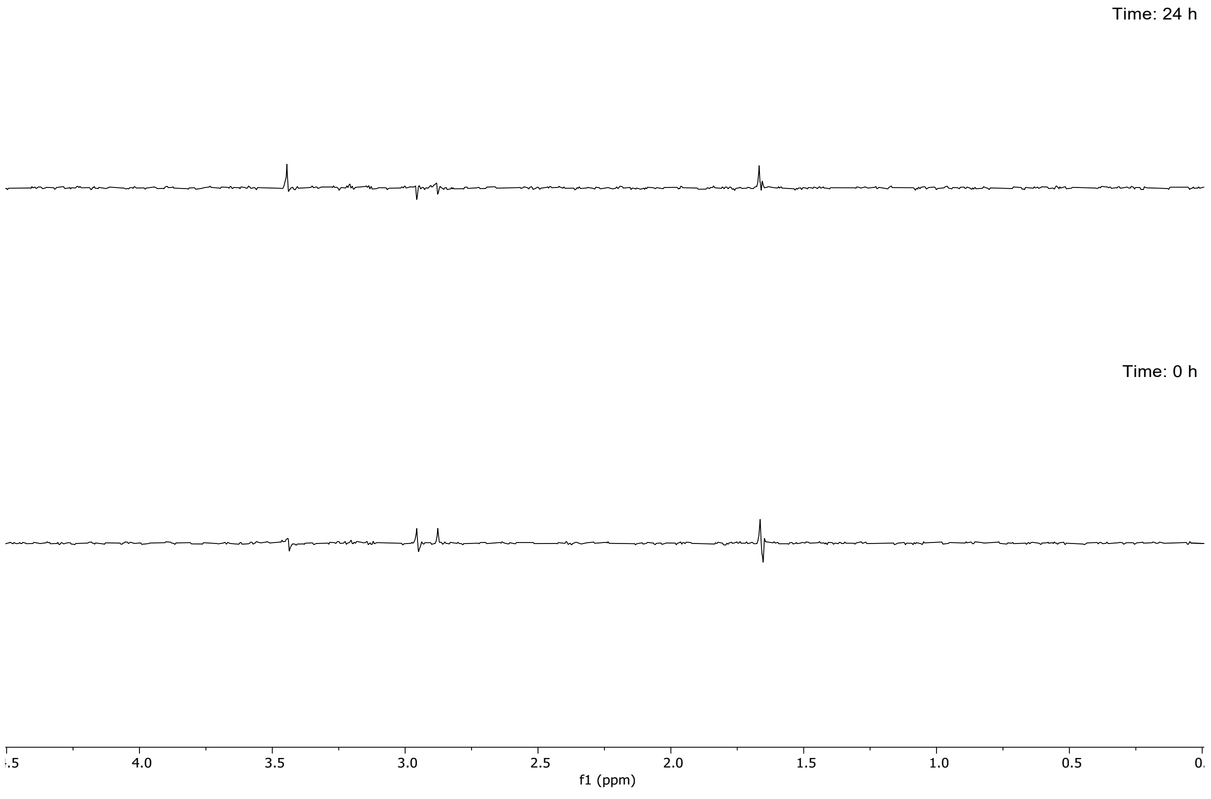

Figure S4 Diffusion edited ^{1}H spectra of the ε-caprolactone reaction mixture. N.B! The visible signals are artefacts from the NMR experiment

Gel permission chromatography (GPC) measurements

The GPC measurements and all the sample preparation were done following this literature procedures.[3,4]

Sample preparation: In a 20 mL glass vial equipped with a cylindrical stirrer bar (10 x 6 mm) was measured 17.5 mg of the desired cellulosic material. Then 1.25 mL anhydrous dimethylacetamide (DMAc) was added and the vial was sealed. Then the sealed vial was immersed into a preheated to 130 °C silicon bath and the suspension was stirred for 2 h. After the above-mentioned time, the mixture was allowed to cool to 90 °C and 87.5 mg of anhydrous lithium chloride (LiCl) was added in one portion to the hot suspension. Then the resulting mixture was stirred for 18 hours furthermore at ambient temperature. Next, the sample was diluted further with 16.25 mL anhydrous DMAc, in order that the final solution of LiCl to be 0.5 w/v %. All samples were filtered through Pall Acrodisc GHP 0.45µm filters before measurement.

Table S2 DP_n and DP_w values of treated cellulose

Time (days)	Number average molecular weight - DP_n	Weight average molecular weight - DP_w
Native MCC	107.438	373.936
0	101.764	379.980
1	121.068	468.854
2	128.469	472.493
3	113.914	446.096
4	137.104	432.959
8	132.231	446.096
16	123.905	390.958

Optical microscopy images

Selected dissolved samples were studied with the help of optical microscope (Olympus BX51TF microscope, equipped with DP70 colour camera and adjustable polarized lenses). The Cross-polarizer angle was optimized for better image contrast, producing differences in colours.

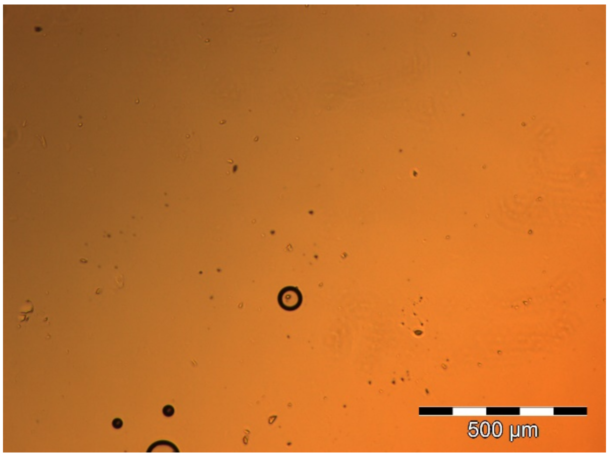

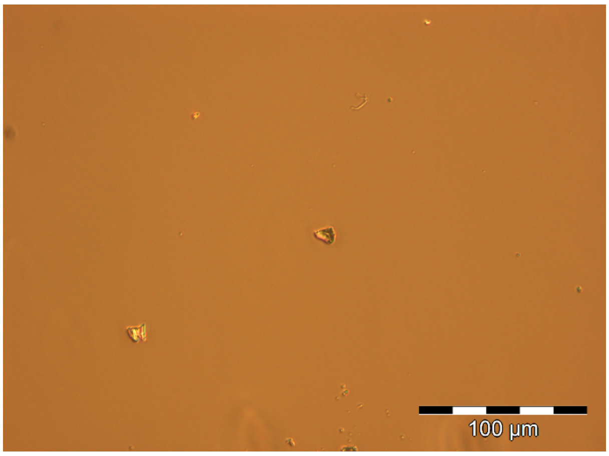

Figure S5 Microscopy images of cellulose 4-nitrobenzoate DS 0.03

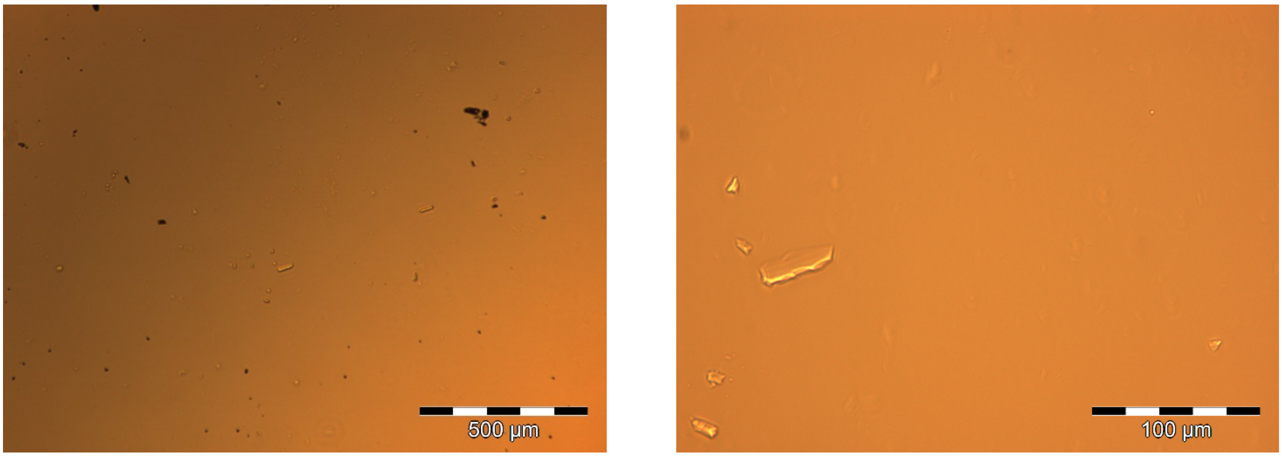

Figure S6 Microscopy images of cellulose acetate DS 0.17

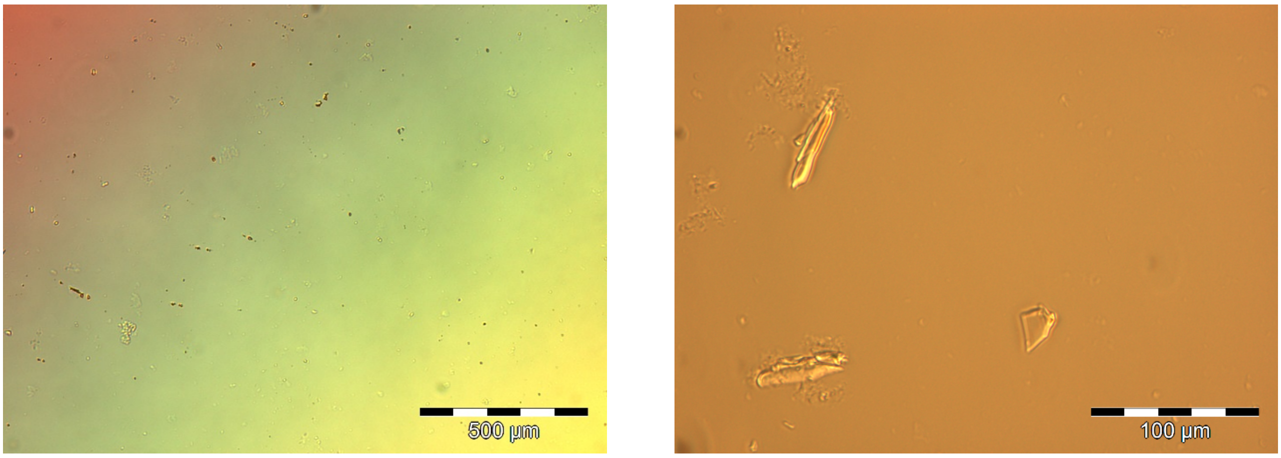

Figure S7 Microscopy images of cellulose 6-hydroxyhexanoate DS 0.05, DG 1.56

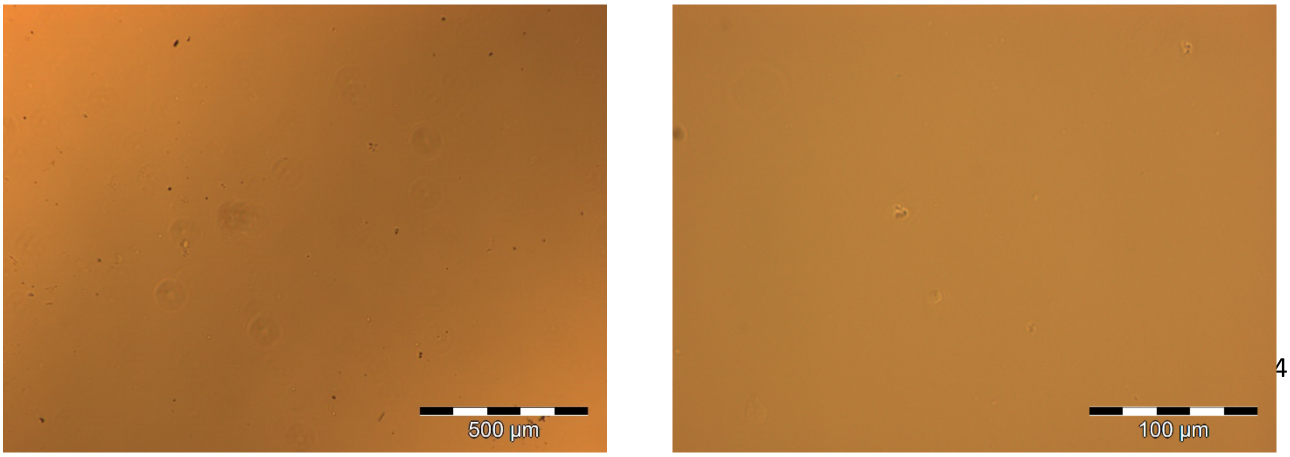

Figure S8 Microscopy images of cellulose glycolate DS 0.05, DG 1.57

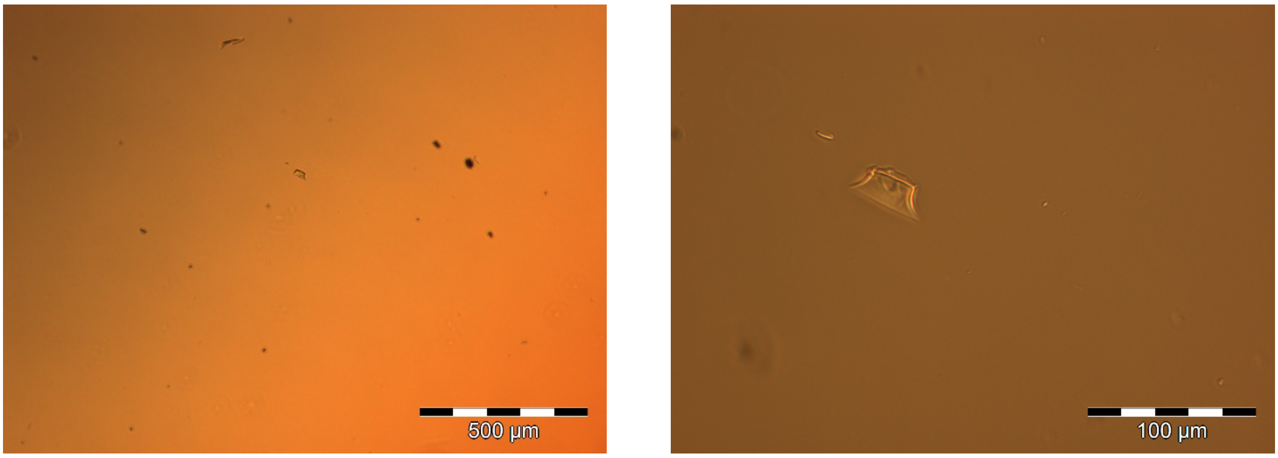

Figure S9 Microscopy images of cellulose 5-hydroxypentanoate DS 0.04, DG 2.01

Cellulose materials – schematic representation

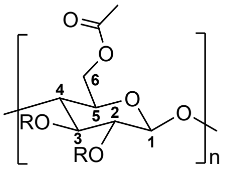

R: H or $-COCH_3$

Cellulose acetate

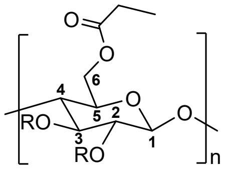

R: H or $-COCH_2CH_3$

Cellulose propionate

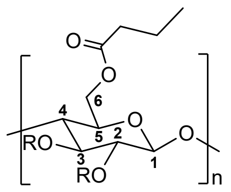

R: H or $-COCH_2CH_2CH_3$

Cellulose butyrate

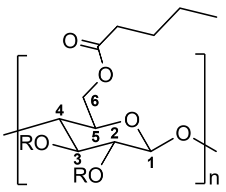

R: H or $-COCH_2(CH_2)_2CH_3$

Cellulose valerate

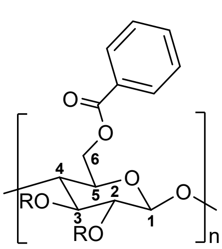

R: H or -COPh

Cellulose benzoate

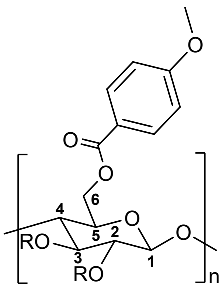

R: H or -CO-(4-MeO)-Ph

Cellulose 4-methoxybenzoate

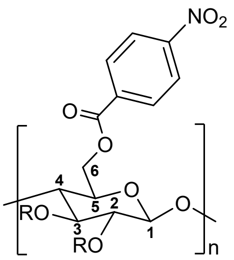

R: H or $-CO(4-NO_2)-Ph$

Cellulose 4-nitrobenzoate

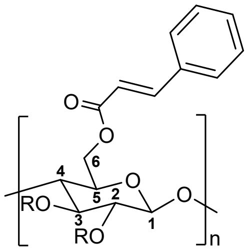

R: H or -COCH=CH-Ph

Cellulose cinnamate

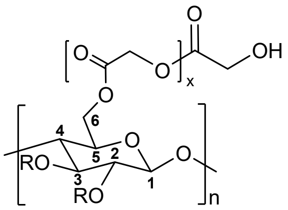

R: H or $-(COCH_2O)_xCOCH_2OH$

Cellulose glycolate

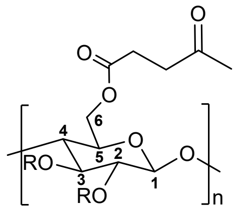

R: H or $-COCH_2CH_2COCH_3$

Cellulose 4-oxopentanoate

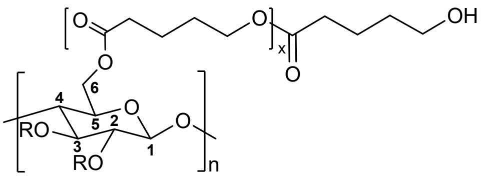

R: H or $-(COCH_2(CH_2)_2CH_2O)_xCOCH_2(CH_2)_2CH_2OH$

Cellulose 5-hydroxypentanoate

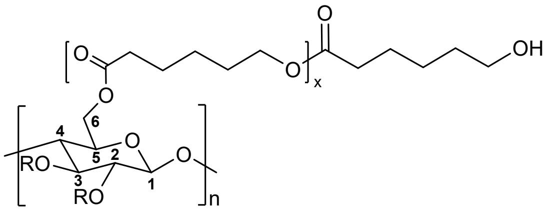

R: H or $-(COCH_2(CH_2)_3CH_2O)_xCOCH_2(CH_2)_3CH_2OH$

Cellulose 6-hydroxyhexanoate

Chart S1. Schematic representation of the prepared cellulose materials

General procedure

Conditions A: A 50 mL round bottom flask was charged with microcrystalline cellulose (0.2500 g, 1.5419 mmol), followed by an oval stirrer bar (20 x 10 mm) and 5 mL [mTBNH][OAc]. The viscous reaction mixture was suspended with vortex genie 2 (scientific industries) and then it was immersed into a preheated to 80 °C silicon bath. After 30 minutes of stirring the MCC was completely dissolved, and 3 equivalents of the desired reagent were added to the reaction mixture. Then the reaction was stirred for 20 h furthermore at 80 °C before it was quenched. After the desired time had passed, the reaction mixture, while hot, was diluted with 45 mL EtOH. Upon cooling, the already diluted reaction mixture was further suspended into 255 mL EtOH, and it was allowed to stir overnight at room temperature. After that the formed cellulosic solids were filtrated off and were washed (x3 times) with EtOH. After the filtration the obtained cellulose material was freeze dried and was subject of data analysis (DS, dissolution, etc.,).

Conditions B: Equivalent to Conditions A with an exception that 9 equivalents of the desired reagent were used.

Conditions C: Equivalent to Conditions A with an exception that 50 h of reaction time was used.

Synthesis of cellulose materials

Cellulose acetate

DS 0.07: The titled material was obtained by following the general procedure - conditions A. Starting from MCC (0.2500 g, 1.5419 mmol) and methyl acetate (0.3685 mL, 4.6257 mmol). Reaction time was 20 h and the temperature was set at 80 °C.

DS 0.13: The titled material was obtained by following the general procedure - conditions B. Starting from MCC (0.2500 g, 1.5419 mmol) and methyl acetate (1.1054 mL, 13.8771 mmol). Reaction time was 20 h and the temperature was set at 80 °C.

DS 0.13: The titled material was obtained by following the general procedure - conditions C. Starting from MCC (0.2500 g, 1.5419 mmol) and methyl acetate (0.3685 mL, 4.6257 mmol). Reaction time was 50 h and the temperature was set at 80 °C.

DS 0.09: The titled material was obtained by following the general procedure - conditions A with exception in the used ionic liquid (5 g [mTBDH][OAc]) and the reaction temperature. Starting from MCC (0.2500 g, 1.5419 mmol) and methyl acetate (0.3685 mL, 4.6257 mmol). Reaction time was 20 h and the temperature was set at 85 °C.

DS 0.17: The titled material was obtained by following the general procedure - conditions B with exception in the used ionic liquid (5 g [mTBDH][OAc]) and the reaction temperature. Starting from MCC (0.2500 g, 1.5419 mmol) and methyl acetate (1.1054 mL, 13.8771 mmol). Reaction time was 20 h and the temperature was set at 85 °C.

DS 0.22: The titled material was obtained by following the general procedure - conditions C with exception in the used ionic liquid (5 g [mTBDH][OAc]) and the reaction temperature. Starting from MCC (0.2500 g, 1.5419 mmol) and methyl acetate (0.3685 mL, 4.6257 mmol). Reaction time was 50 h and the temperature was set at 85 °C.

DS 1.75: The titled material was obtained by following the general procedure - conditions A. Starting from MCC (0.2500 g, 1.5419 mmol) and *i*-propenyl acetate (0.5095 mL, 4.6257 mmol). Reaction time was 20 h and the temperature was set at 80 °C.

DS 1.44: The titled material was obtained by following the general procedure - conditions A. Starting from MCC (0.2500 g, 1.5419 mmol) and vinyl acetate (0.4264 mL, 4.6257 mmol). Reaction time was 20 h and the temperature was set at 80 °C.

DS 0.11: The titled material was obtained by following the general procedure - conditions A with exceptions in the amount of the added reagent and the reaction temperature. Starting from MCC (0.5000 g, 3.0838 mmol) and *i*-propenyl acetate (0.1698 mL, 1.5419 mmol). Reaction time was 20 h and the temperature was set at 65 °C.

DS 0.32: The titled material was obtained by following the general procedure - conditions A with exceptions in the amount of the added reagent and the reaction temperature. Starting from MCC (0.5000 g, 3.0838 mmol) and *i*-propenyl acetate (0.3397 mL, 3.0838 mmol). Reaction time was 20 h and the temperature was set at 65 °C.

DS 0.59: The titled material was obtained by following the general procedure - conditions A with exceptions in the amount of the added reagent and the reaction temperature. Starting from MCC (0.5000 g, 3.0838 mmol) and *i*-propenyl acetate (0.5095 mL, 4.6257 mmol). Reaction time was 20 h and the temperature was set at 65 °C.

DS 0.72: The titled material was obtained by following the general procedure - conditions A with exceptions in the amount of the added reagent and the reaction temperature. Starting from MCC (0.5000 g, 3.0838 mmol) and *i*-propenyl acetate (0.6793 mL, 6.1676 mmol). Reaction time was 20 h and the temperature was set at 65 °C.

DS 1.07: The titled material was obtained by following the general procedure - conditions A with exceptions in the amount of the added reagent and the reaction temperature. Starting from MCC (0.5000 g, 3.0838 mmol) and *i*-propenyl acetate (0.8491 mL, 7.7095 mmol). Reaction time was 20 h and the temperature was set at 65 °C.

DS 1.29: The titled material was obtained by following the general procedure - conditions A with exceptions in the amount of the added reagent and the reaction temperature. Starting from MCC (0.5000 g, 3.0838 mmol) and *i*-propenyl acetate (1.0190 mL, 9.2514 mmol). Reaction time was 20 h and the temperature was set at 65 °C.

Cellulose propionate

DS 0.06: The titled material was obtained by following the general procedure - conditions A. Starting from MCC (0.2500 g, 1.5419 mmol) and methyl propionate (0.4454 mL, 4.6257 mmol). Reaction time was 20 h and the temperature was set at 80 °C.

DS 0.10: The titled material was obtained by following the general procedure - conditions B. Starting from MCC (0.2500 g, 1.5419 mmol) and methyl propionate (1.3363 mL, 13.8771 mmol). Reaction time was 20 h and the temperature was set at 80 °C.

DS 0.08: The titled material was obtained by following the general procedure - conditions C. Starting from MCC (0.2500 g, 1.5419 mmol) and methyl propionate (0.4454 mL, 4.6257 mmol). Reaction time was 50 h and the temperature was set at 80 °C.

Cellulose butyrate

DS 0.04: The titled material was obtained by following the general procedure - conditions A. Starting from MCC (0.2500 g, 1.5419 mmol) and methyl butyrate (0.5261 mL, 4.6257 mmol). Reaction time was 20 h and the temperature was set at 80 °C.

DS 0.05: The titled material was obtained by following the general procedure - conditions B. Starting from MCC (0.2500 g, 1.5419 mmol) and methyl butyrate (1.5782 mL, 13.8771 mmol). Reaction time was 20 h and the temperature was set at 80 °C.

DS 0.05: The titled material was obtained by following the general procedure - conditions C. Starting from MCC (0.2500 g, 1.5419 mmol) and methyl butyrate (0.5261 mL, 4.6257 mmol). Reaction time was 50 h and the temperature was set at 80 °C.

Cellulose valerate

DS 0.04: The titled material was obtained by following the general procedure - conditions A. Starting from MCC (0.2500 g, 1.5419 mmol) and methyl pentanoate (0.6141 mL, 4.6257 mmol). Reaction time was 20 h and the temperature was set at 80 °C.

DS 0.05: The titled material was obtained by following the general procedure - conditions B. Starting from MCC (0.2500 g, 1.5419 mmol) and methyl pentanoate (1.8422 mL, 13.8771 mmol). Reaction time was 20 h and the temperature was set at 80 °C.

DS 0.06: The titled material was obtained by following the general procedure - conditions C. Starting from MCC (0.2500 g, 1.5419 mmol) and methyl pentanoate (0.6141 mL, 4.6257 mmol). Reaction time was 50 h and the temperature was set at 80 °C.

Cellulose benzoate

DS 0.02: The titled material was obtained by following the general procedure - conditions A. Starting from MCC (0.2500 g, 1.5419 mmol) and methyl benzoate (0.5789 mL, 4.6257 mmol). Reaction time was 20 h and the temperature was set at 80 °C.

DS 0.05: The titled material was obtained by following the general procedure - conditions B. Starting from MCC (0.2500 g, 1.5419 mmol) and methyl benzoate (1.7366 mL, 13.8771 mmol). Reaction time was 20 h and the temperature was set at 80 °C.

DS 0.02: The titled material was obtained by following the general procedure - conditions C. Starting from MCC (0.2500 g, 1.5419 mmol) and methyl benzoate (0.5789 mL, 4.6257 mmol). Reaction time was 50 h and the temperature was set at 80 °C.

Cellulose 4-methoxybenzoate

DS 0.005: The titled material was obtained by following the general procedure - conditions A. Starting from MCC (0.2500 g, 1.5419 mmol) and methyl 4-methoxybenzoate (0.7687 g, 4.6257 mmol). Reaction time was 20 h and the temperature was set at 80 °C.

DS 0.01: The titled material was obtained by following the general procedure - conditions B. Starting from MCC (0.2500 g, 1.5419 mmol) and methyl 4-methoxybenzoate (2.3061 g, 13.8771 mmol). Reaction time was 20 h and the temperature was set at 80 °C.

DS 0.01: The titled material was obtained by following the general procedure - conditions C. Starting from MCC (0.2500 g, 1.5419 mmol) and methyl 4-methoxybenzoate (0.7687 g, 4.6257 mmol). Reaction time was 50 h and the temperature was set at 80 °C.

Cellulose 4-nitrobenzoate

DS 0.25: The titled material was obtained by following the general procedure - conditions A. Starting from MCC (0.2500 g, 1.5419 mmol) and methyl 4-nitrobenzoate (0.8379 g, 4.6257 mmol). Reaction time was 20 h and the temperature was set at 80 °C.

DS 0.75: The titled material was obtained by following the general procedure - conditions B. Starting from MCC (0.2500 g, 1.5419 mmol) and methyl 4-nitrobenzoate (2.5138 g, 13.8771 mmol). Reaction time was 20 h and the temperature was set at 80 °C.

DS 0.03: The titled material was obtained by following the general procedure - conditions C. Starting from MCC (0.2500 g, 1.5419 mmol) and methyl 4-nitrobenzoate (0.8379 g, 4.6257 mmol). Reaction time was 50 h and the temperature was set at 80 °C.

Cellulose cinnamate

DS 0.03: The titled material was obtained by following the general procedure - conditions A. Starting from MCC (0.2500 g, 1.5419 mmol) and methyl cinnamate (0.7502 g, 4.6257 mmol). Reaction time was 20 h and the temperature was set at 80 °C.

DS 0.07: The titled material was obtained by following the general procedure - conditions B. Starting from MCC (0.2500 g, 1.5419 mmol) and methyl cinnamate (2.2507 g, 13.8771 mmol). Reaction time was 20 h and the temperature was set at 80 °C.

DS 0.07: The titled material was obtained by following the general procedure - conditions C. Starting from MCC (0.2500 g, 1.5419 mmol) and methyl cinnamate (0.7502 g, 4.6257 mmol). Reaction time was 50 h and the temperature was set at 80 °C.

Cellulose glycolate

DS 0.05, DG 1.57: The titled material was obtained by following the general procedure - conditions A. Starting from MCC (0.2500 g, 1.5419 mmol) and methyl glycolate (0.3571 mL, 4.6257 mmol). Reaction time was 20 h and the temperature was set at 80 °C.

Cellulose 4-oxopentanoate

DS 0.01: The titled material was obtained by following the general procedure - conditions A. Starting from MCC (0.2500 g, 1.5419 mmol) and α-angelica lactone (0.4156 mL, 4.6257 mmol). Reaction time was 20 h and the temperature was set at 80 °C.

Cellulose 5-hydroxypentanoate

DS 0.04, DG 2.01: The titled material was obtained by following the general procedure - conditions A. Starting from MCC (0.2500 g, 1.5419 mmol) and δ-valerolactone (0.4292 mL, 4.6257 mmol). Reaction time was 20 h and the temperature was set at 80 °C.

Cellulose 6-hydroxyhexanoate

DS 0.24, DG 2.29: The titled material was obtained by following the general procedure - conditions A. Starting from MCC (0.2500 g, 1.5419 mmol) and ε-caprolactone (0.4934 mL, 4.6257 mmol). Reaction time was 20 h and the temperature was set at 80 °C.

DS 0.01, DG 1.84: The titled material was obtained by following the general procedure - conditions A with exceptions in the amount of the added reagent and the reaction temperature. Starting from MCC (0.2500 g, 1.5419 mmol) and ε-caprolactone (0.0822 mL, 0.7710 mmol). Reaction time was 20 h and the temperature was set at 65 °C.

DS 0.03, DG 1.64: The titled material was obtained by following the general procedure - conditions A with exceptions in the amount of the added reagent and the reaction temperature. Starting from MCC (0.2500 g, 1.5419 mmol) and ε-caprolactone (0.1645 mL, 1.5419 mmol). Reaction time was 20 h and the temperature was set at 65 °C.

DS 0.05, DG 1.56: The titled material was obtained by following the general procedure - conditions A with exceptions in the amount of the added reagent and the reaction temperature. Starting from MCC (0.2500 g, 1.5419 mmol) and *ε*-caprolactone (0.2467 mL, 2.3129 mmol). Reaction time was 20 h and the temperature was set at 65 °C.

DS 0.07, DG 1.53: The titled material was obtained by following the general procedure - conditions A with exceptions in the amount of the added reagent and the reaction temperature. Starting from MCC (0.2500 g, 1.5419 mmol) and *ε*-caprolactone (0.3290 mL, 3.0838 mmol). Reaction time was 20 h and the temperature was set at 65 °C.

DS 0.07, DG 1.58: The titled material was obtained by following the general procedure - conditions A with exceptions in the amount of the added reagent and the reaction temperature. Starting from MCC (0.2500 g, 1.5419 mmol) and *ε*-caprolactone (0.4112 mL, 3.8548 mmol). Reaction time was 20 h and the temperature was set at 65 °C.

DS 0.10, DG 1.76: The titled material was obtained by following the general procedure - conditions A with exceptions in the amount of the added reagent and the reaction temperature. Starting from MCC (0.2500 g, 1.5419 mmol) and *ε*-caprolactone (0.4934 mL, 4.6257 mmol). Reaction time was 20 h and the temperature was set at 65 °C.

References

1. A. W. T. King, V. Mäkelä, S. A. Kedzior, T. Laaksonen, G. J. Partl, S. Heikkinen, H. Koskela, H. A. Heikkinen, A. J. Holding, E. D. Cranston, and I. Kilpeläinen, *Biomacromolecules*, 2018, **19**, 2708–2720.
2. T. Koso, M. Beaumont, B. L. Tardy, D. R. del Cerro, S. Eyley, W. Thielemans, O. J. Rojas, I. Kilpeläinen and A. W. T. King, *Green Chem.*, 2022, **24**, 5604-5613.
3. T. Kakko, A. W. T. King and I. Kilpeläinen, *Cellulose*, 2017, **24**, 5341-5354.
4. D. R. del Cerro, T. V. Koso, T. Kakko, A. W. T. King and I. Kilpeläinen, *Cellulose*, 2020, **27**, 5545-5562.

Copy of spectral data

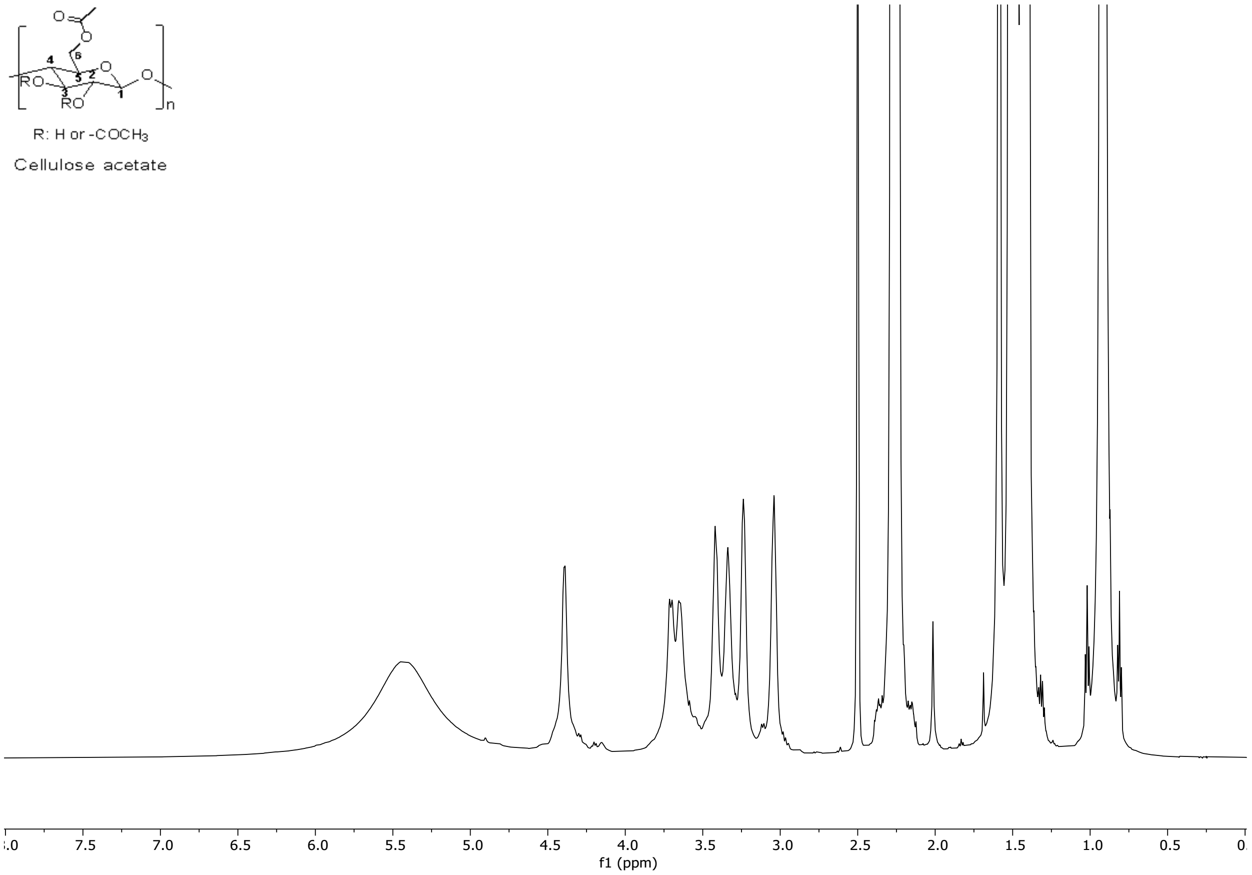

^{1}H and diffusion-edited ^{1}H spectra of **Cellulose acetate DS 0.07** in 20 w.% tetrabutylphosphonium acetate DMSO-d_6 electrolyte solution.

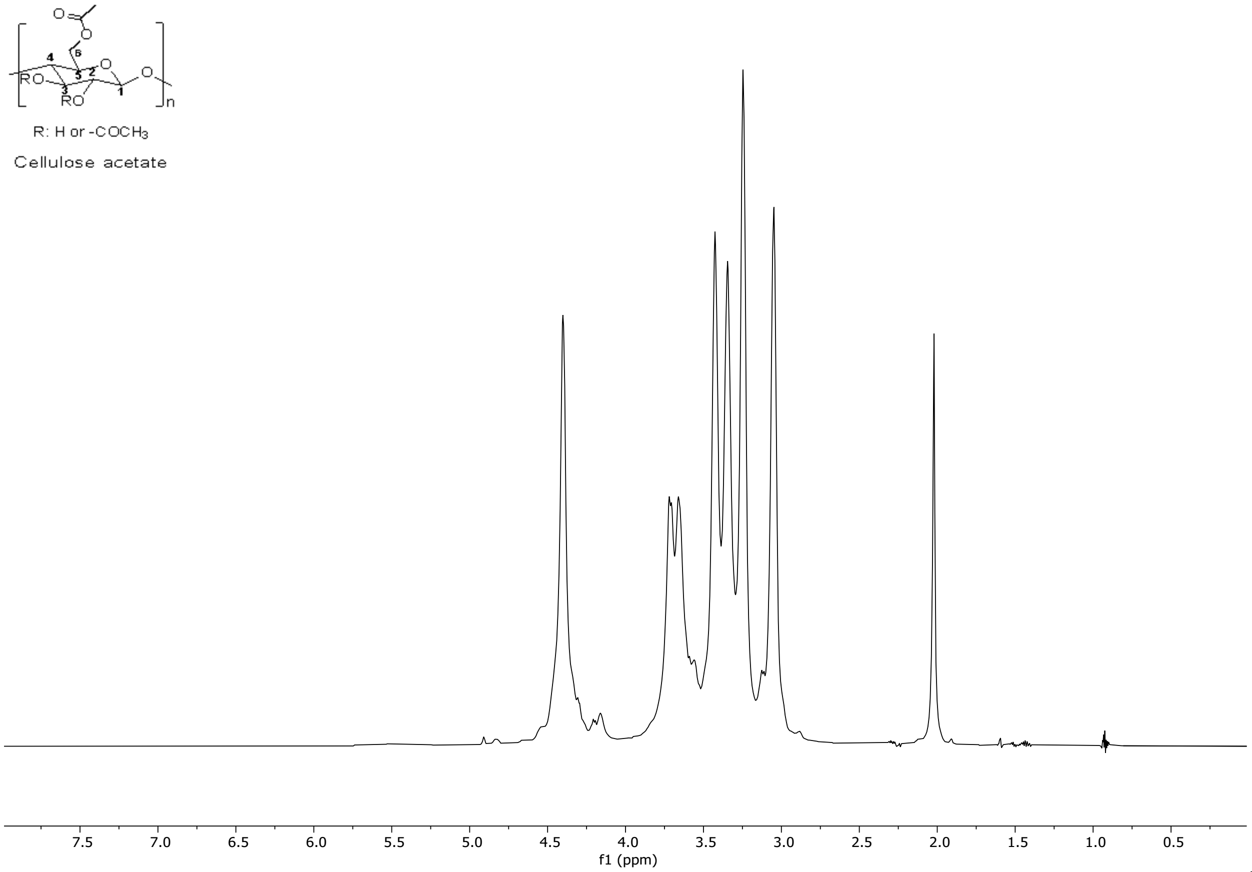

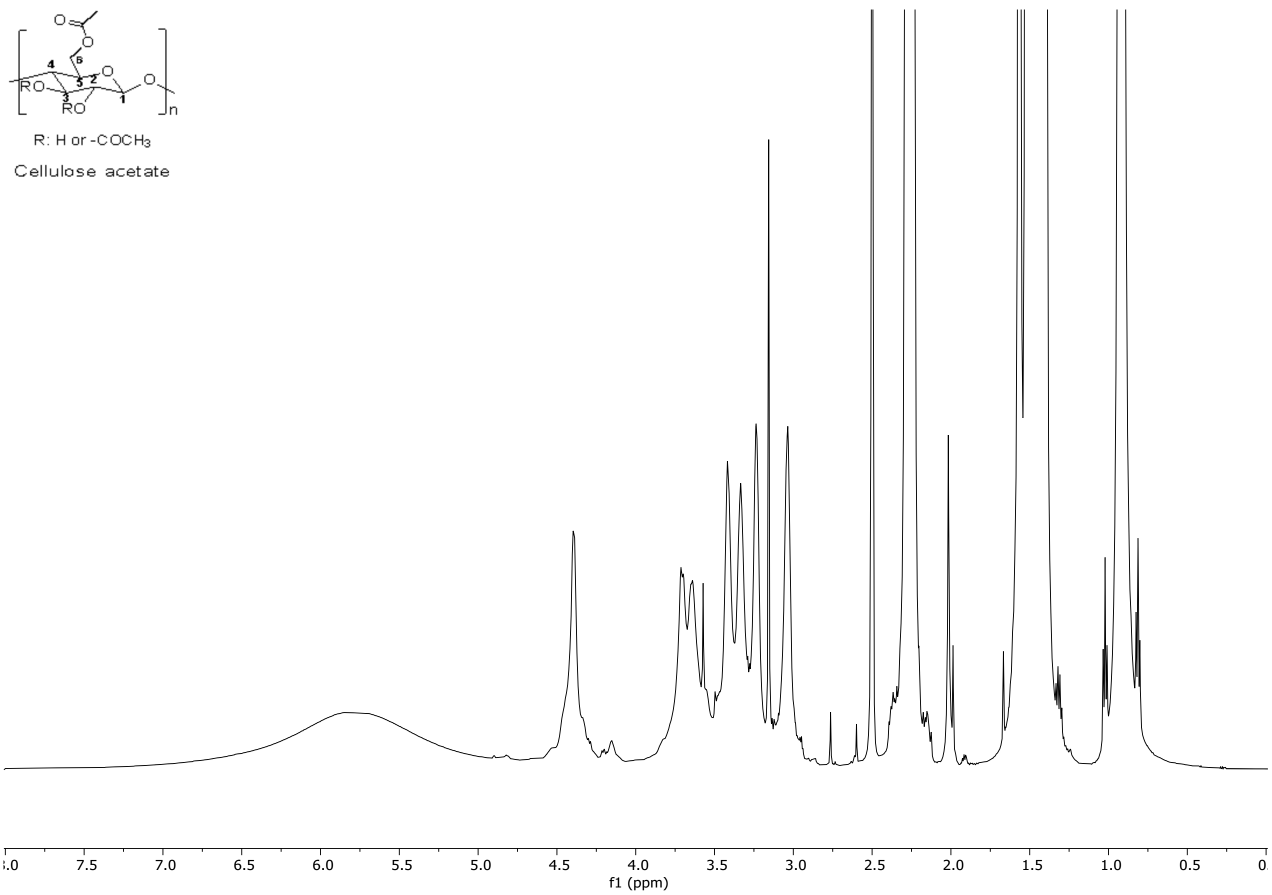

^{1}H and diffusion-edited ^{1}H spectra of **Cellulose acetate DS 0.13** in 20 w.% tetrabutylphosphonium acetate DMSO-d_6 electrolyte solution.

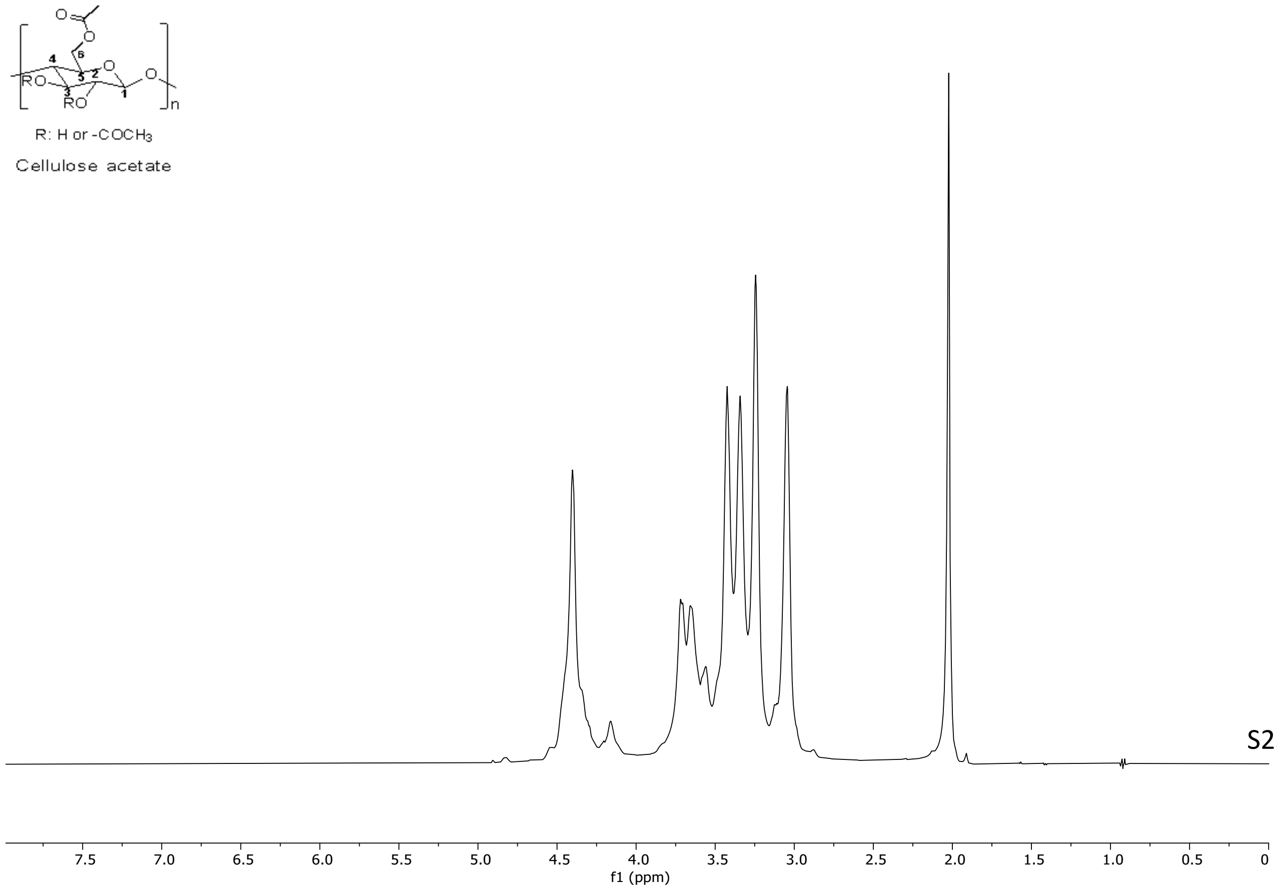

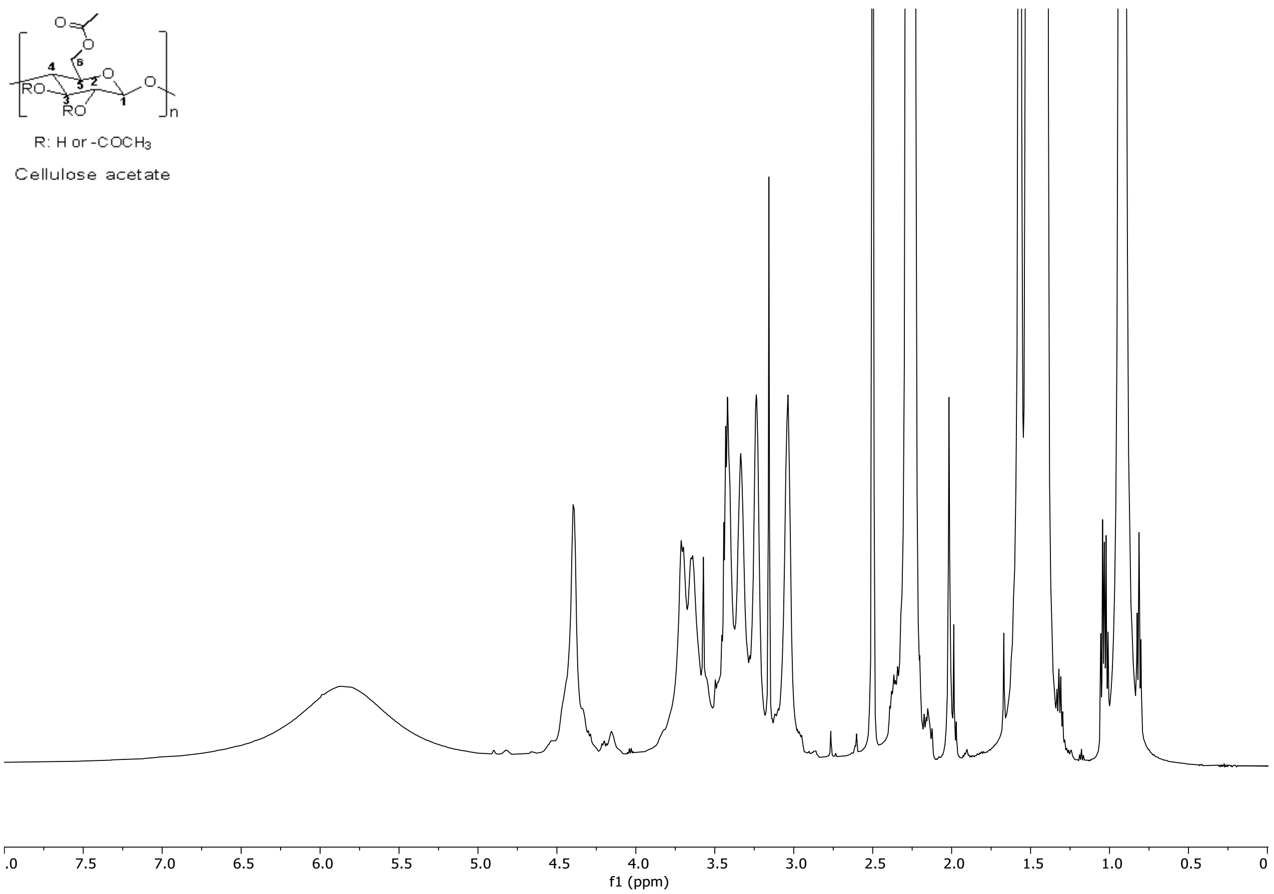

^{1}H and diffusion-edited ^{1}H spectra of **Cellulose acetate DS 0.13** in 20 w.% tetrabutylphosphonium acetate DMSO-d_6 electrolyte solution.

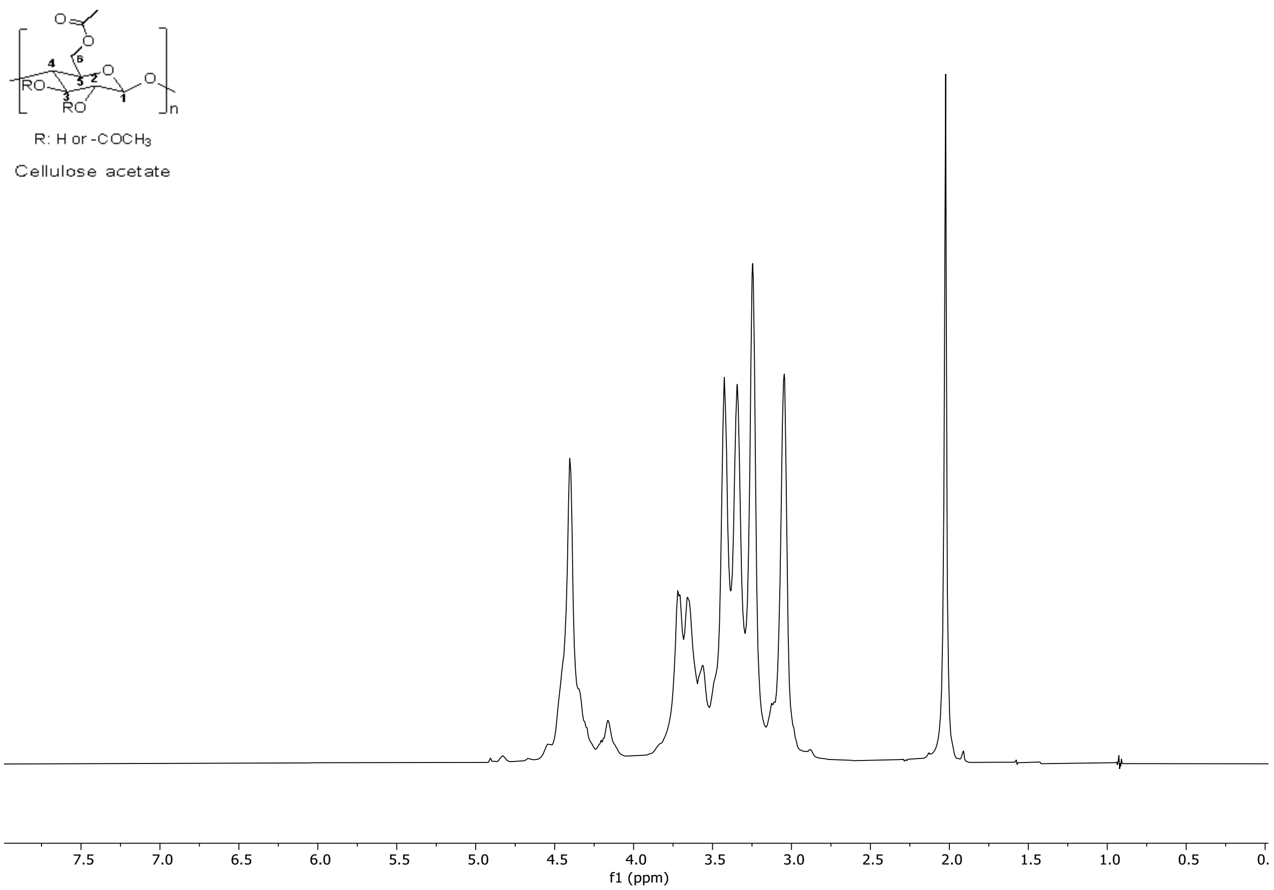

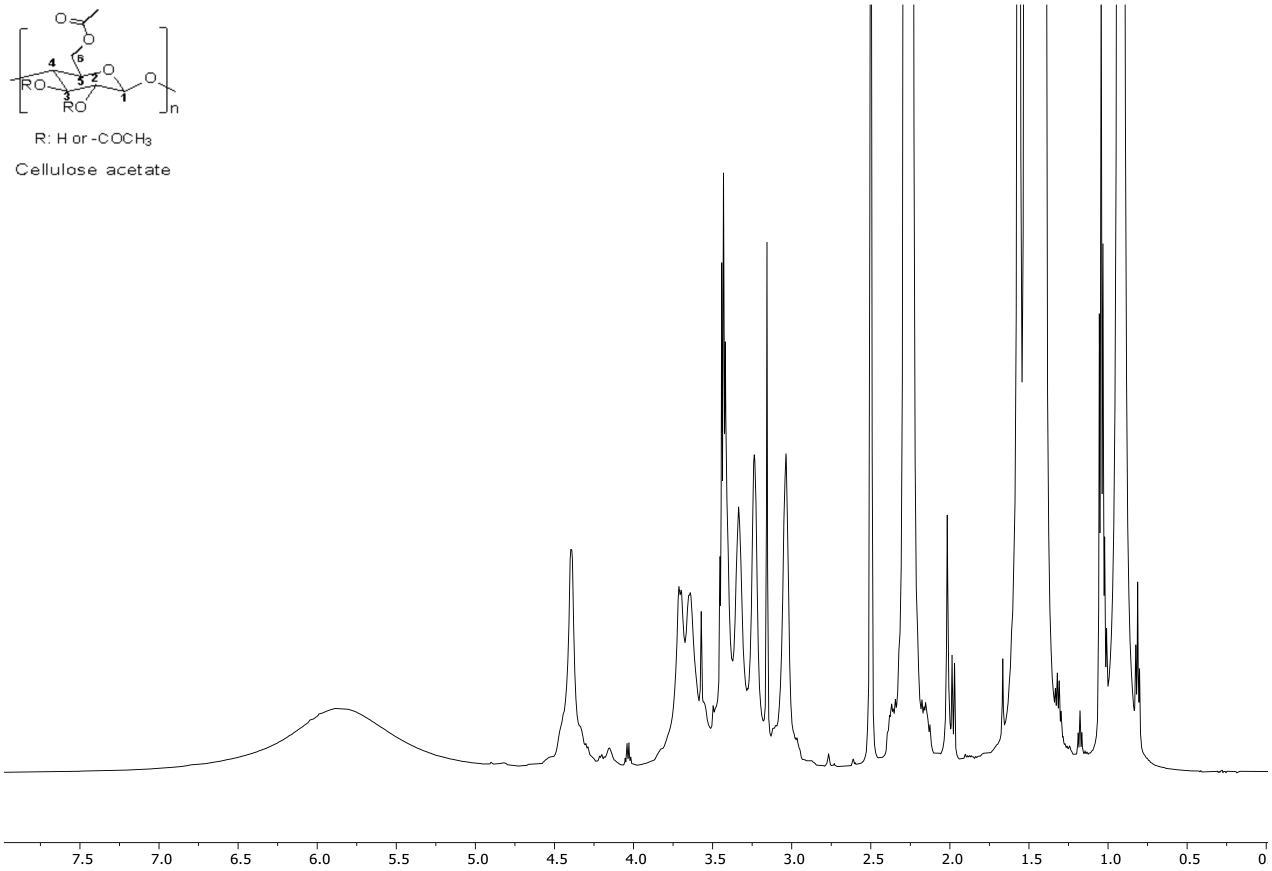

^{1}H and diffusion-edited ^{1}H spectra of **Cellulose acetate DS 0.09** in 20 w.% tetrabutylphosphonium acetate DMSO-d_6 electrolyte solution.

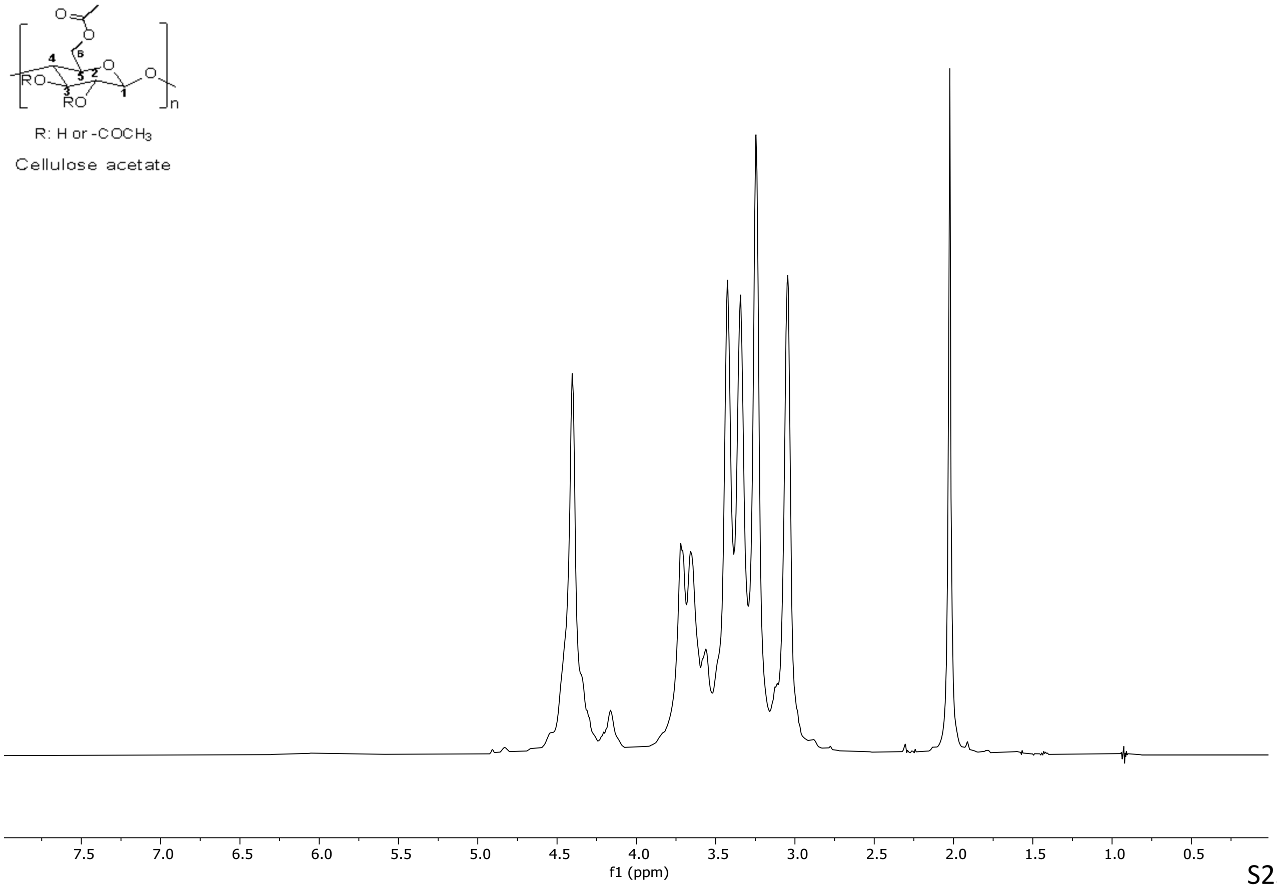

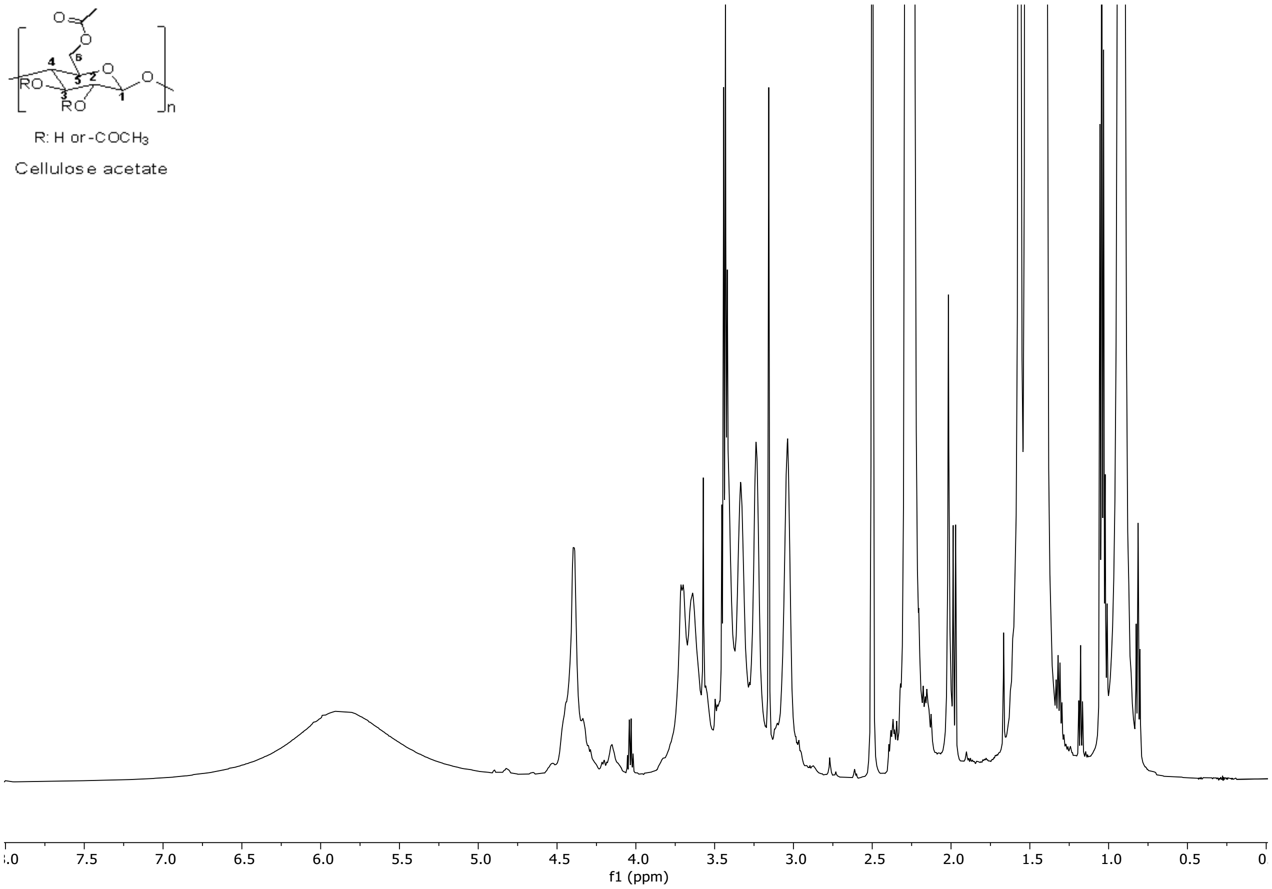

^{1}H and diffusion-edited ^{1}H spectra of **Cellulose acetate DS 0.17** in 20 w.% tetrabutylphosphonium acetate DMSO-d_6 electrolyte solution.

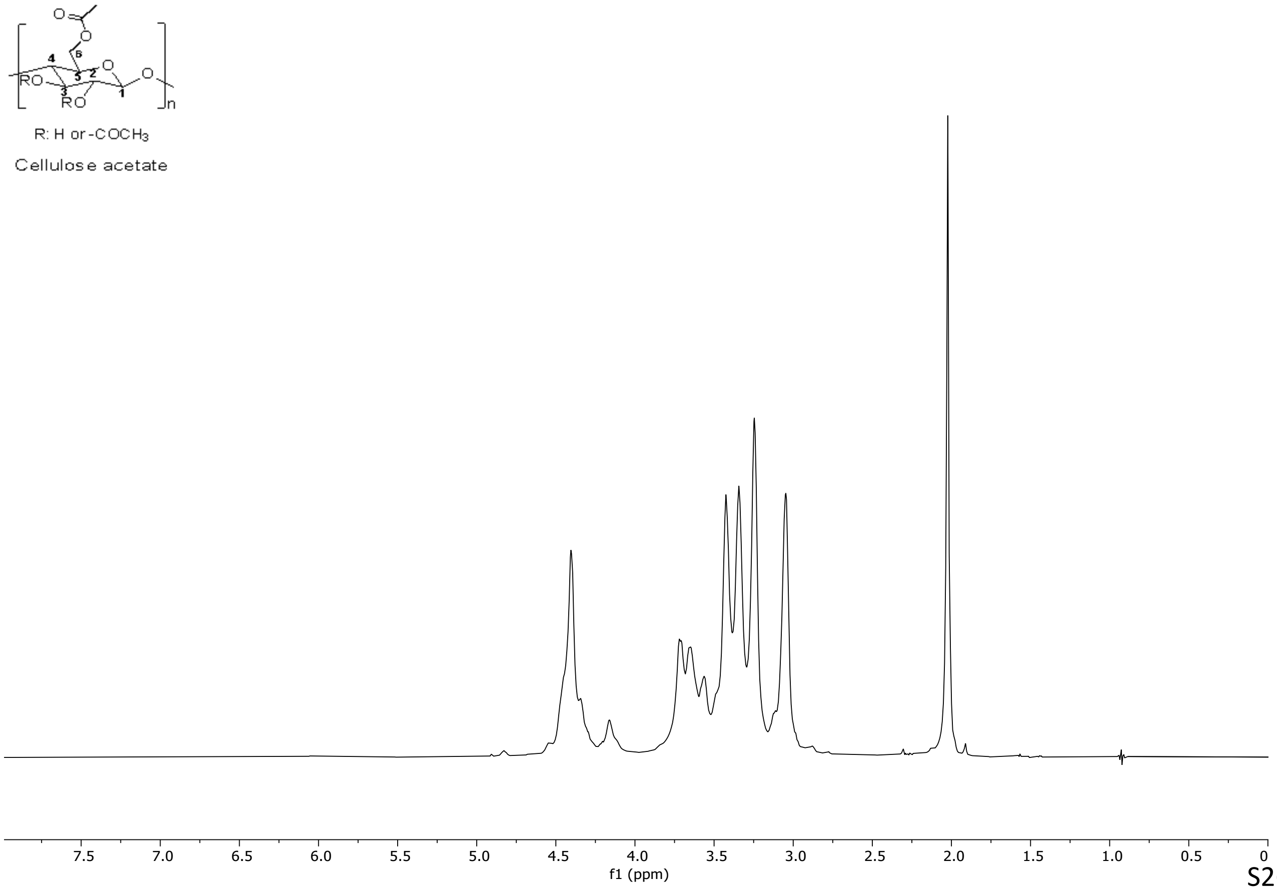

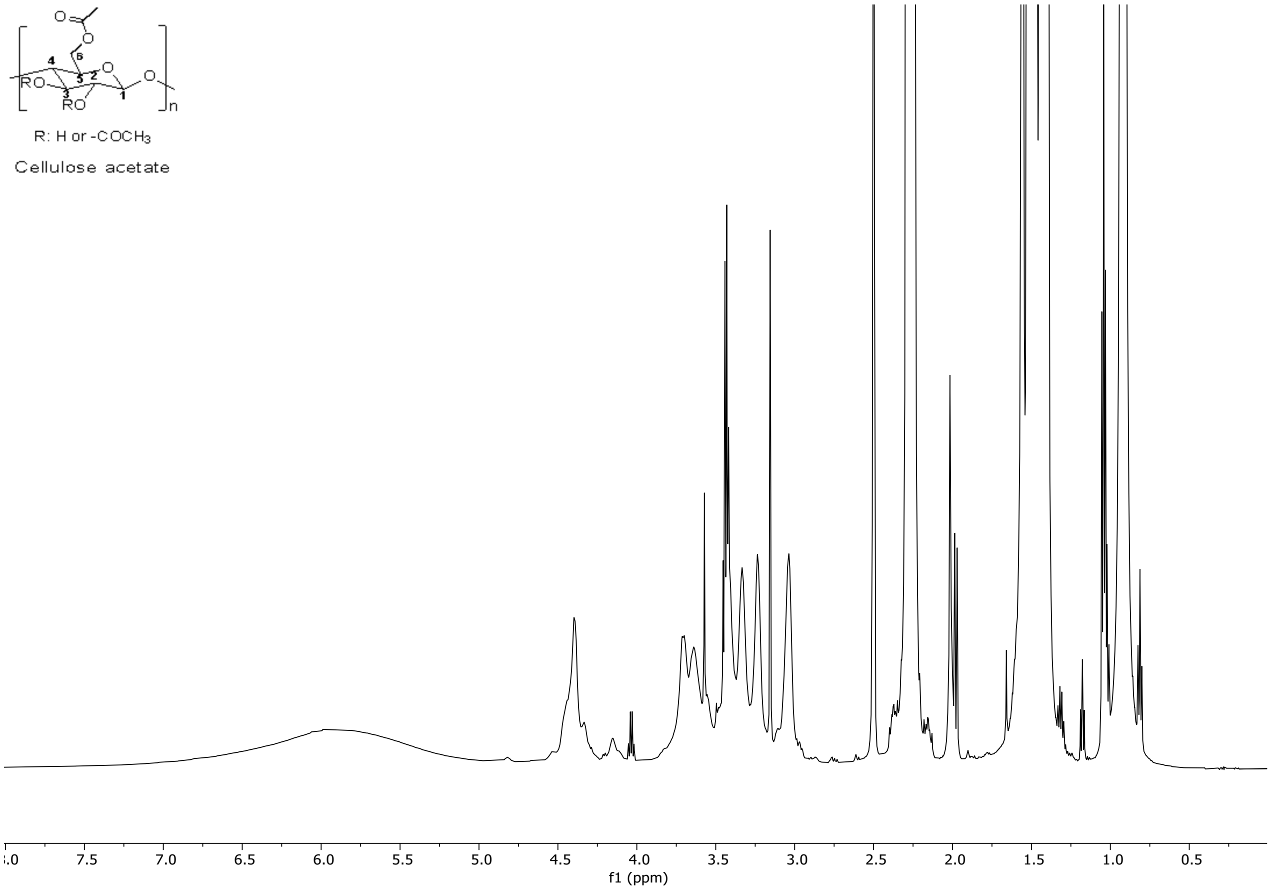

^{1}H and diffusion-edited ^{1}H spectra of **Cellulose acetate DS 0.22** in 20 w.% tetrabutylphosphonium acetate DMSO-d_6 electrolyte solution.

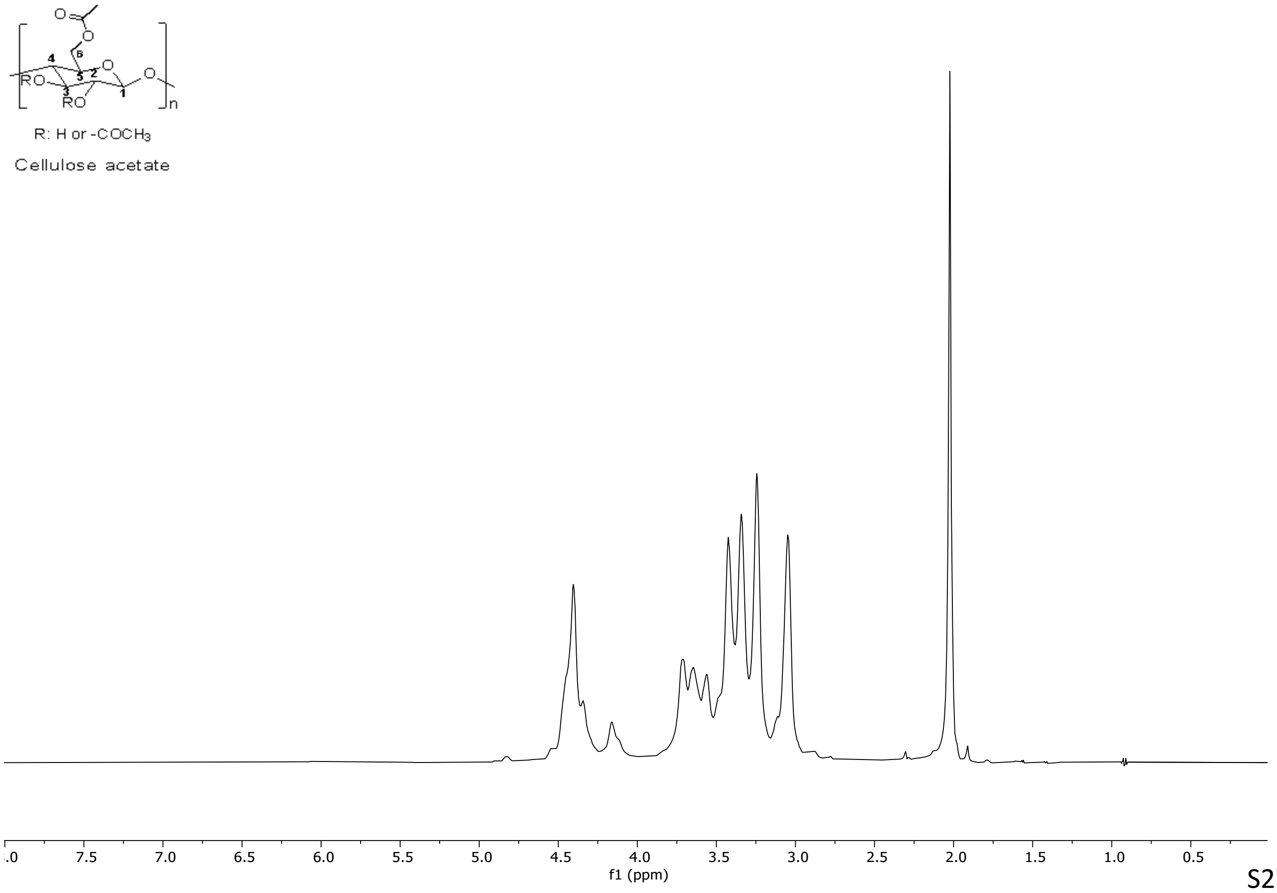

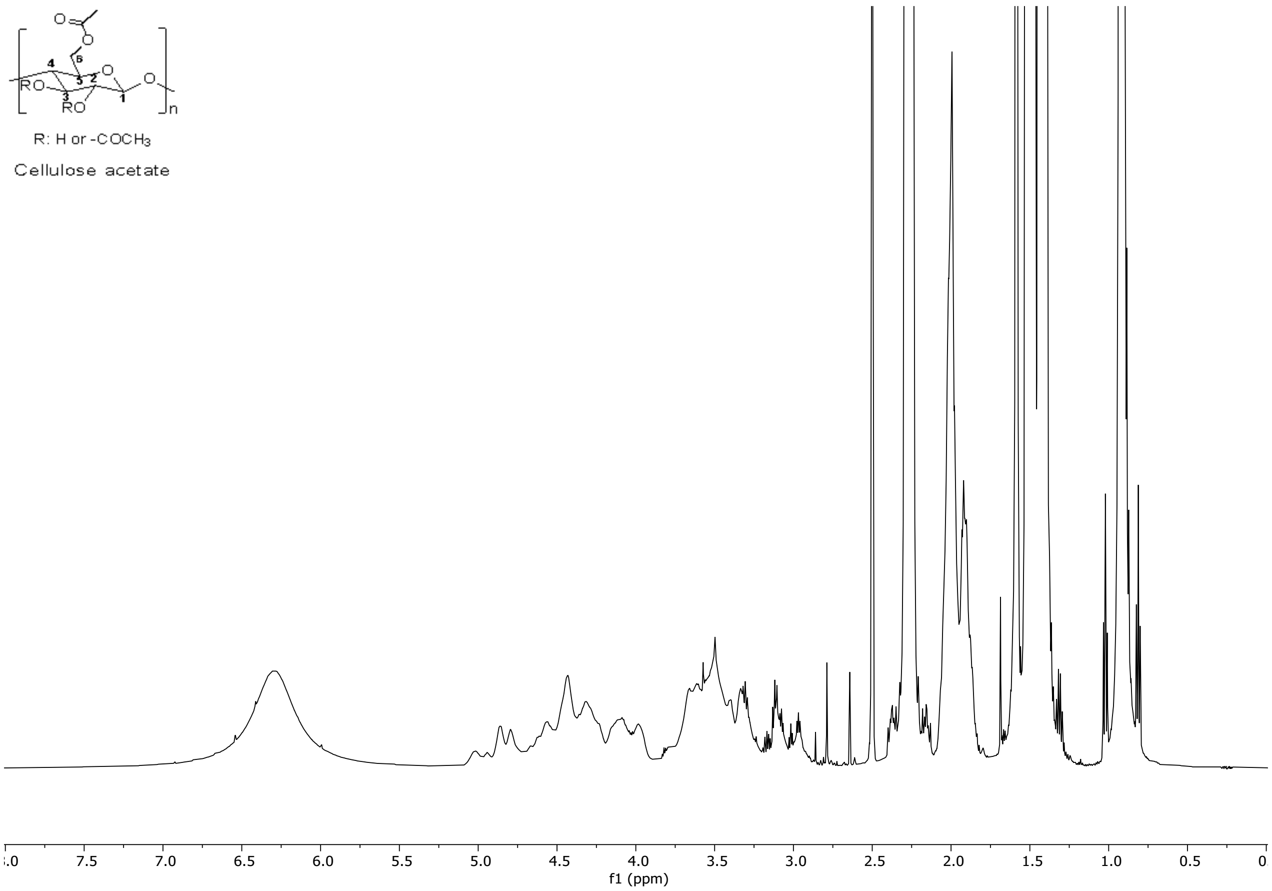

^{1}H and diffusion-edited ^{1}H spectra of **Cellulose acetate DS 1.75** in 20 w.% tetrabutylphosphonium acetate DMSO-d_6 electrolyte solution.

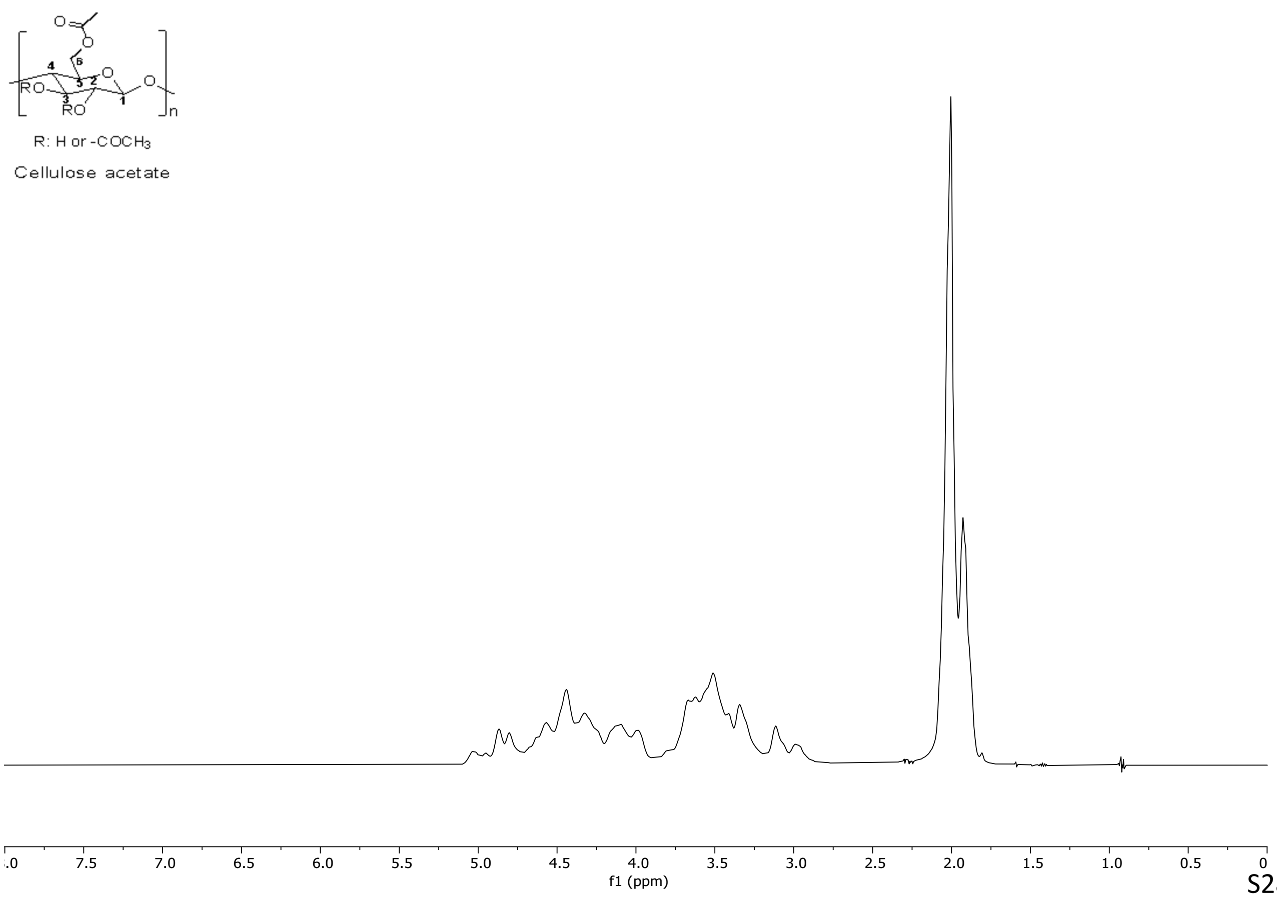

^1H and diffusion-edited ^1H spectra of **Cellulose acetate DS 1.44** in 20 w.% tetrabutylphosphonium acetate DMSO-d_6 electrolyte solution.

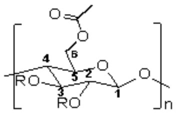

R: H or -$COCH_3$

Cellulose acetate

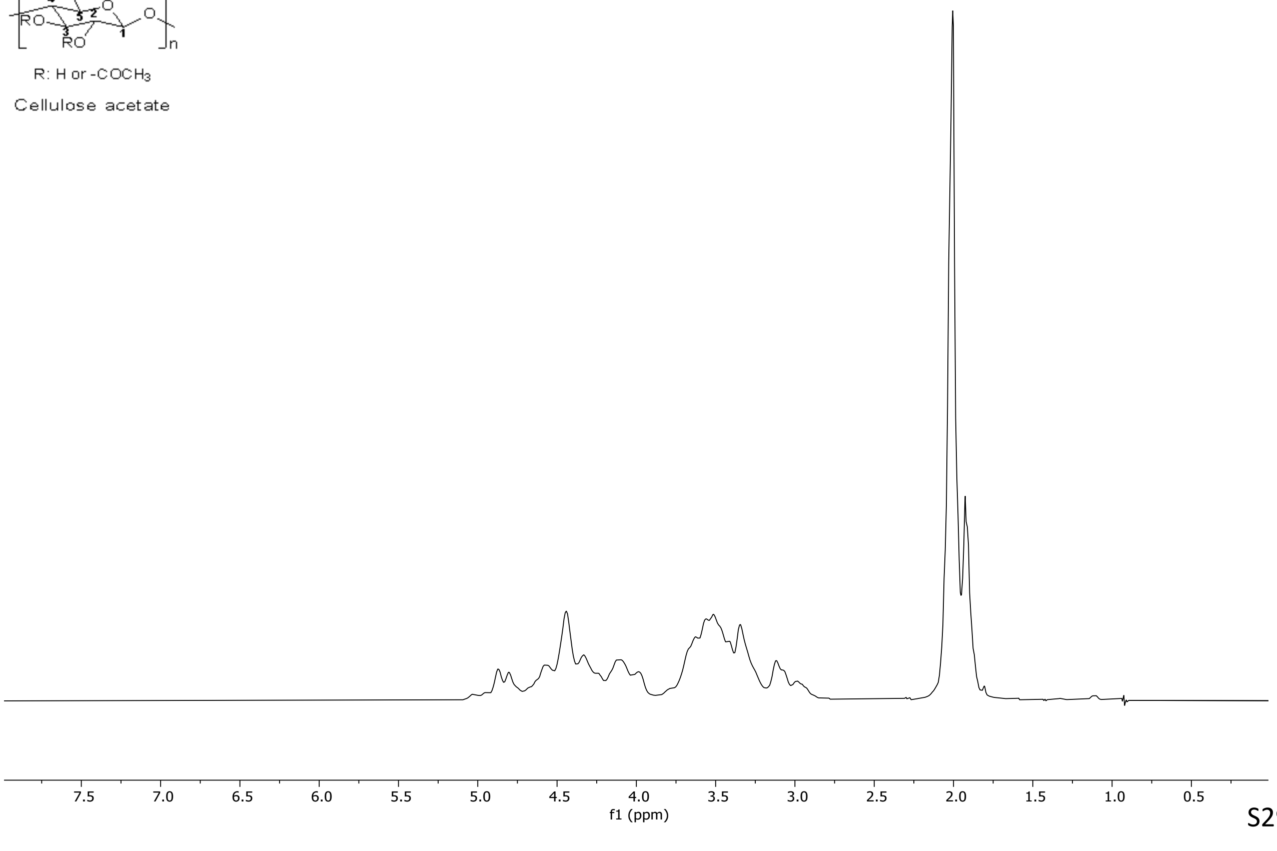

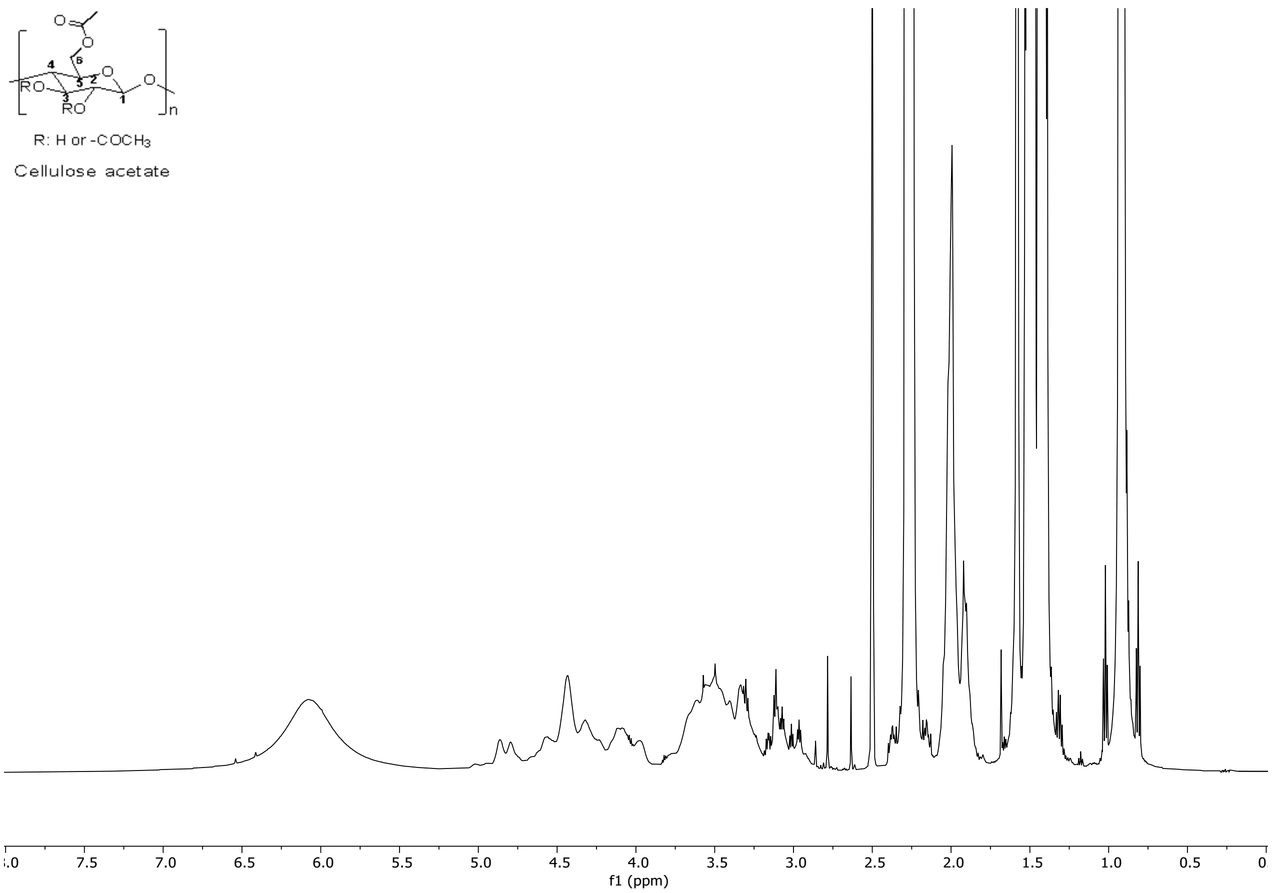
R: H or -COCH3
Cellulose acetate
f1 (ppm)

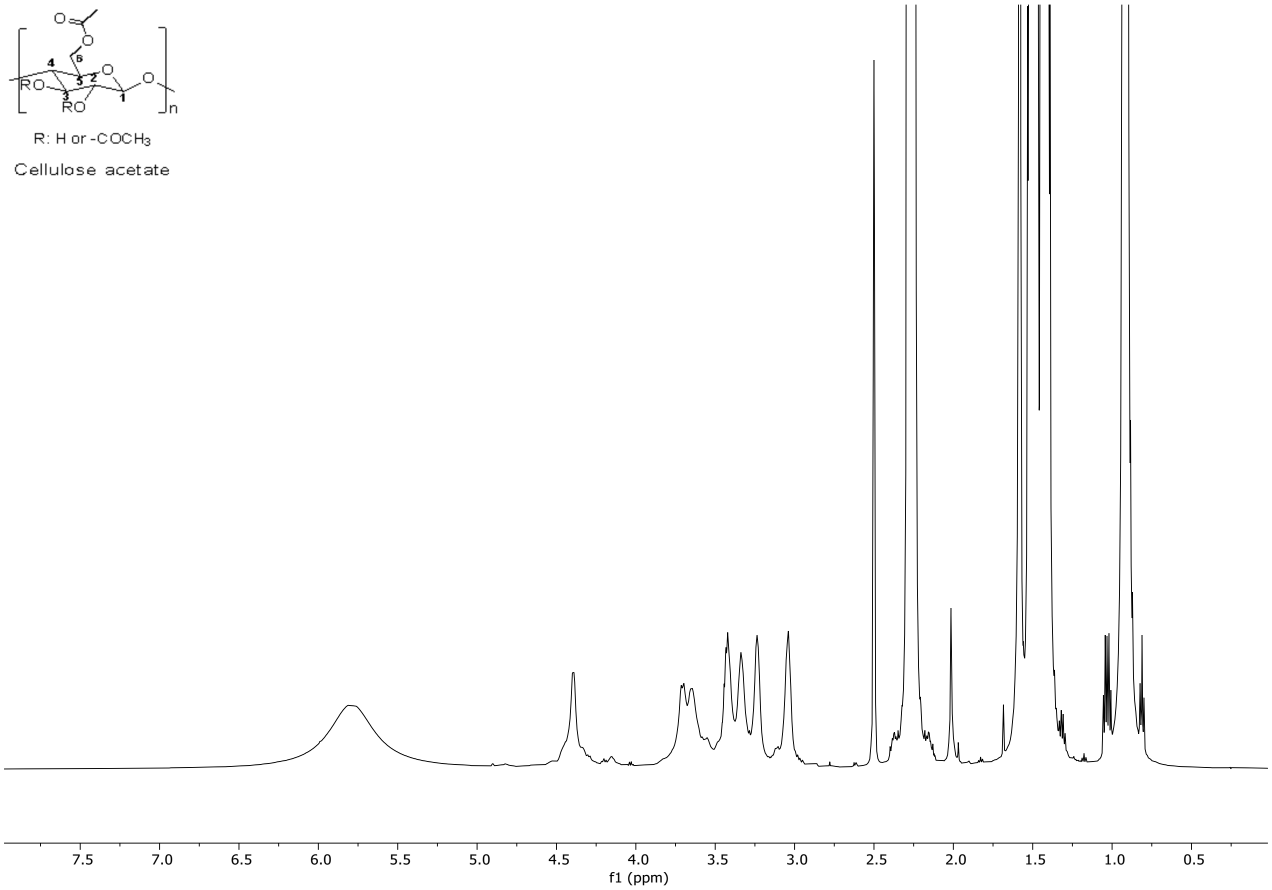

^{1}H and diffusion-edited ^{1}H spectra of **Cellulose acetate DS 0.11** in 20 w.% tetrabutylphosphonium acetate DMSO-d_6 electrolyte solution.

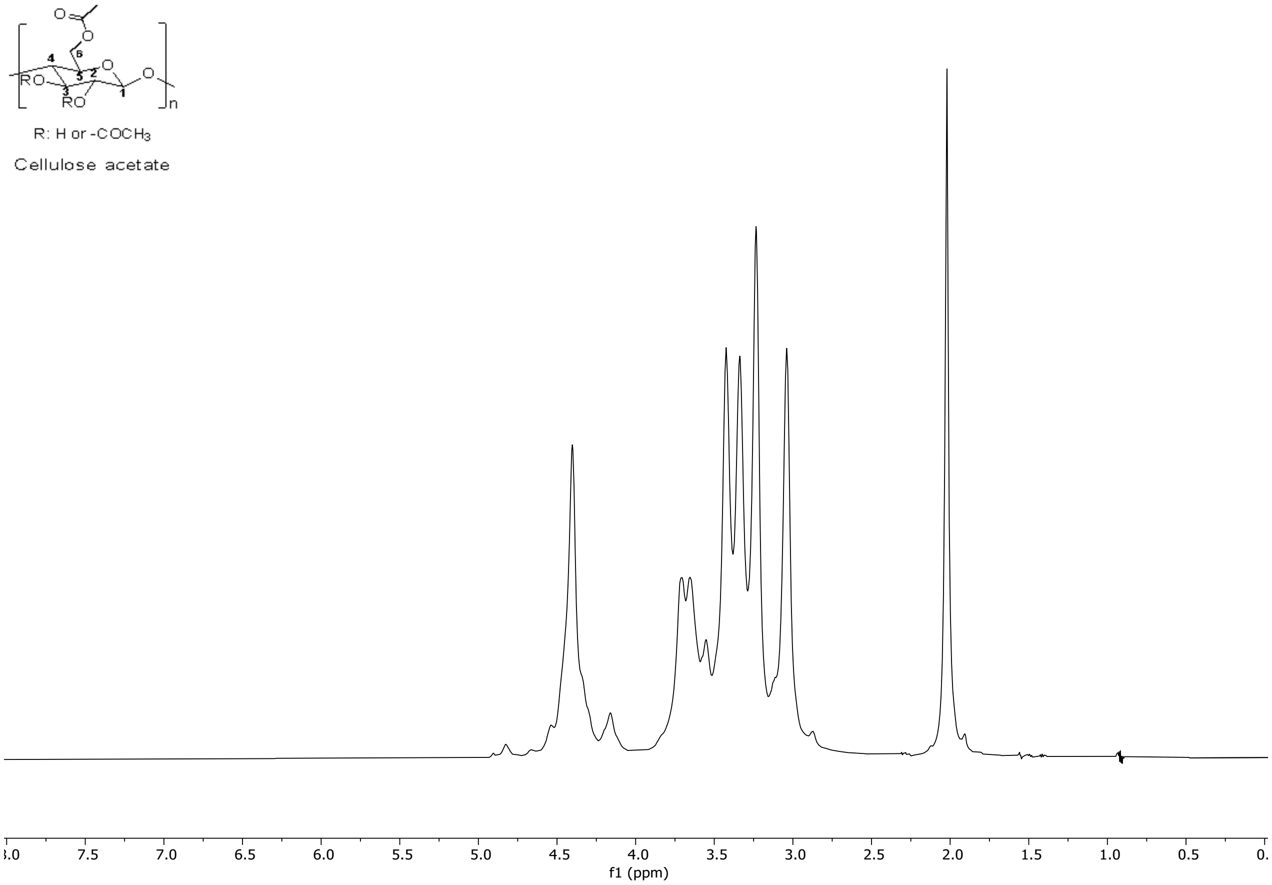

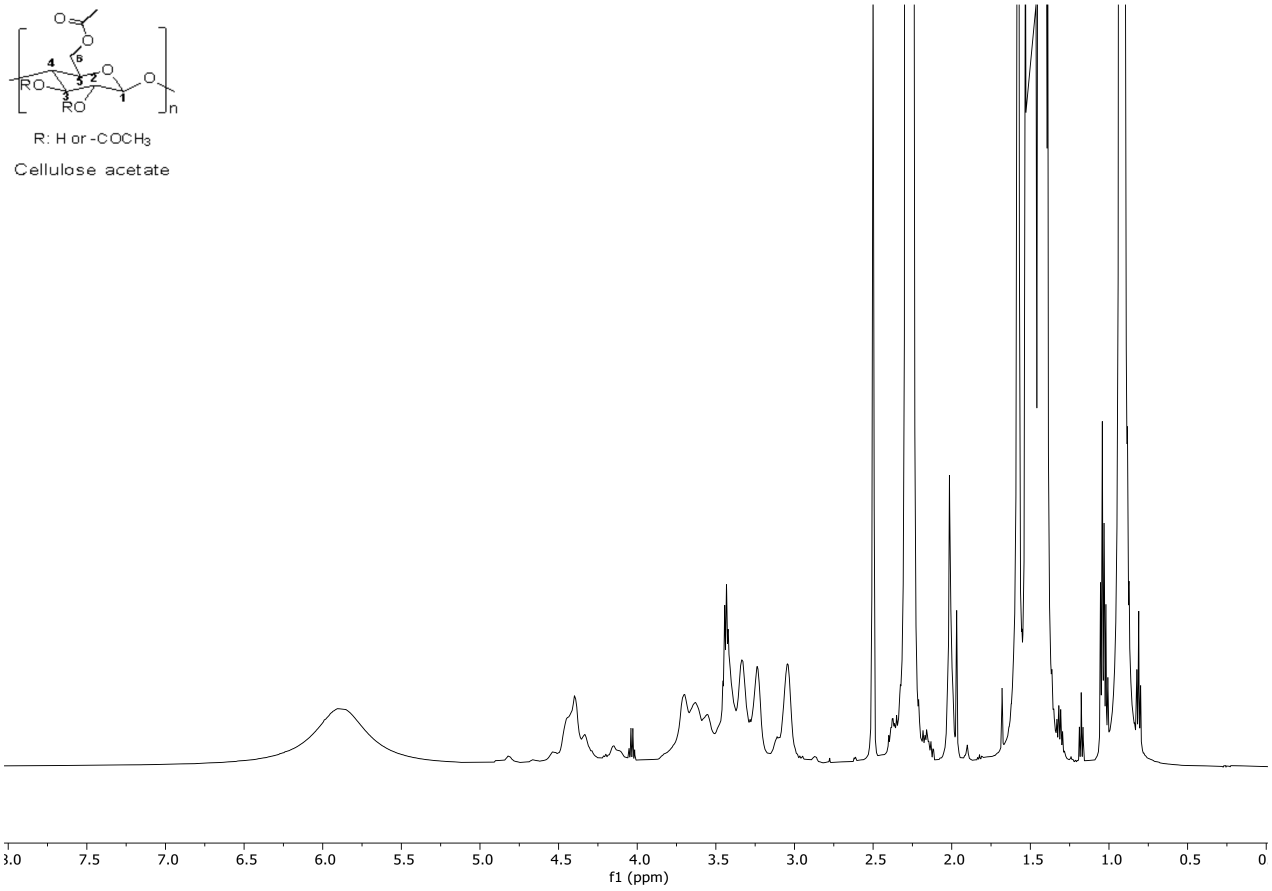

^{1}H and diffusion-edited ^{1}H spectra of **Cellulose acetate DS 0.32** in 20 w.% tetrabutylphosphonium acetate DMSO-d_6 electrolyte solution.

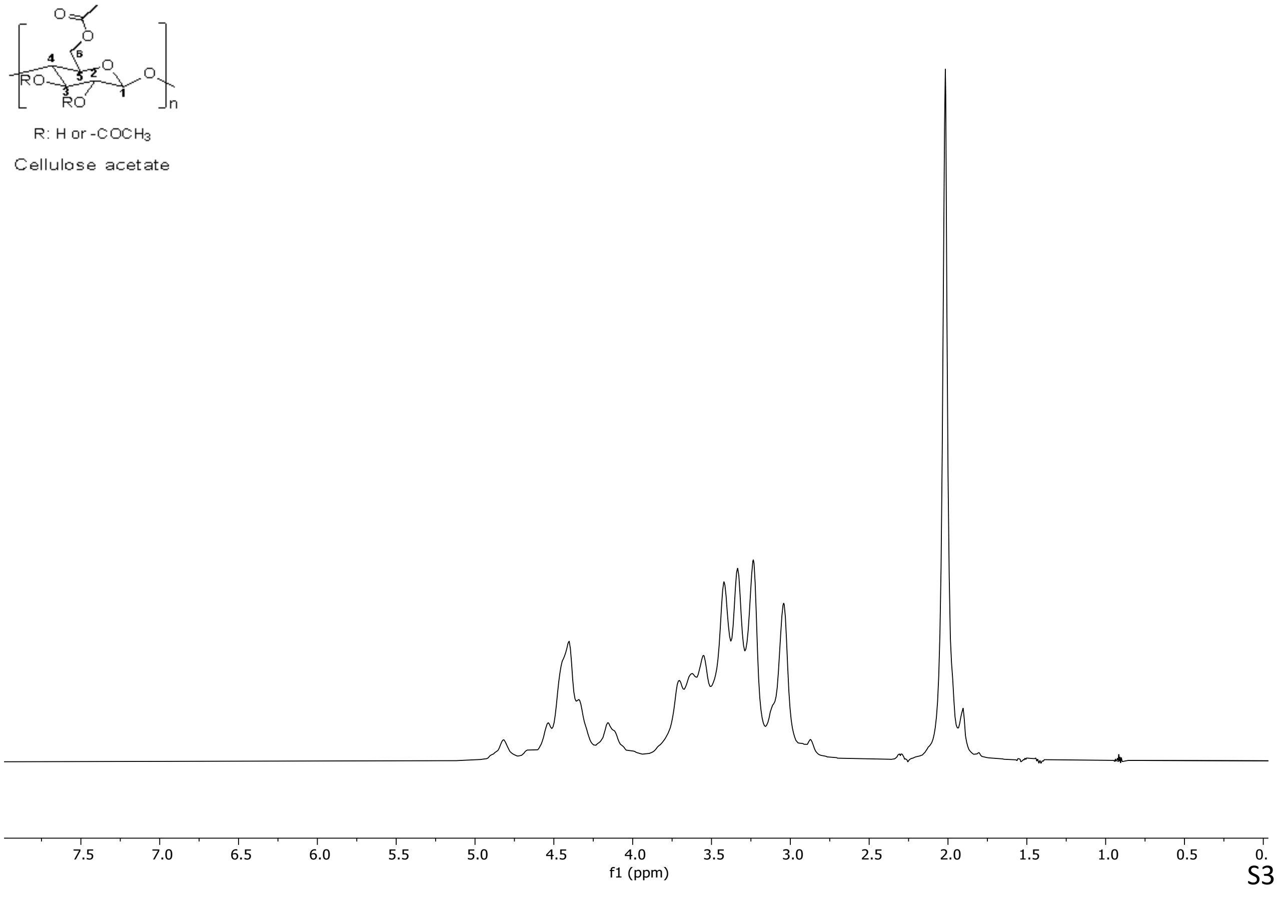

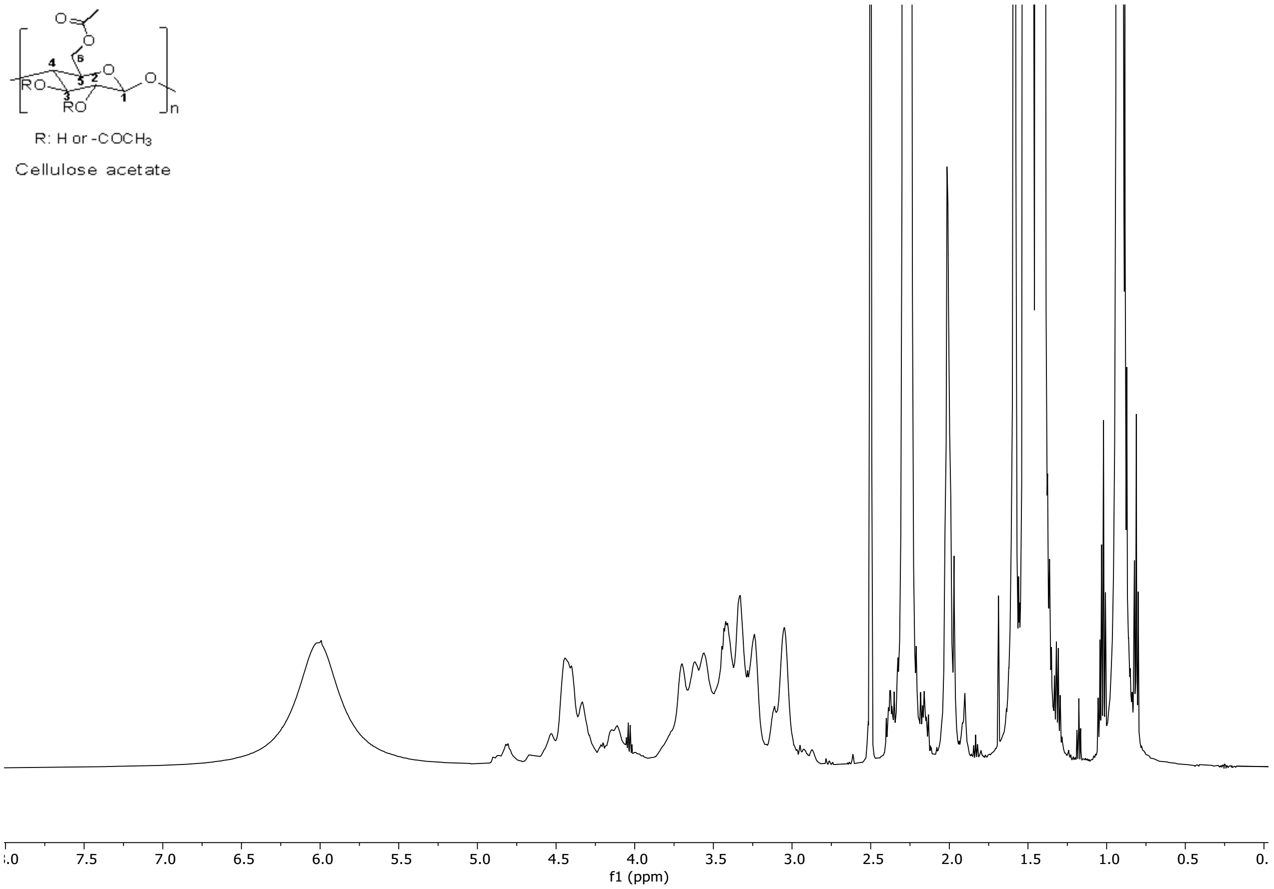

^{1}H and diffusion-edited ^{1}H spectra of **Cellulose acetate DS 0.59** in 20 w.% tetrabutylphosphonium acetate DMSO-d_6 electrolyte solution.

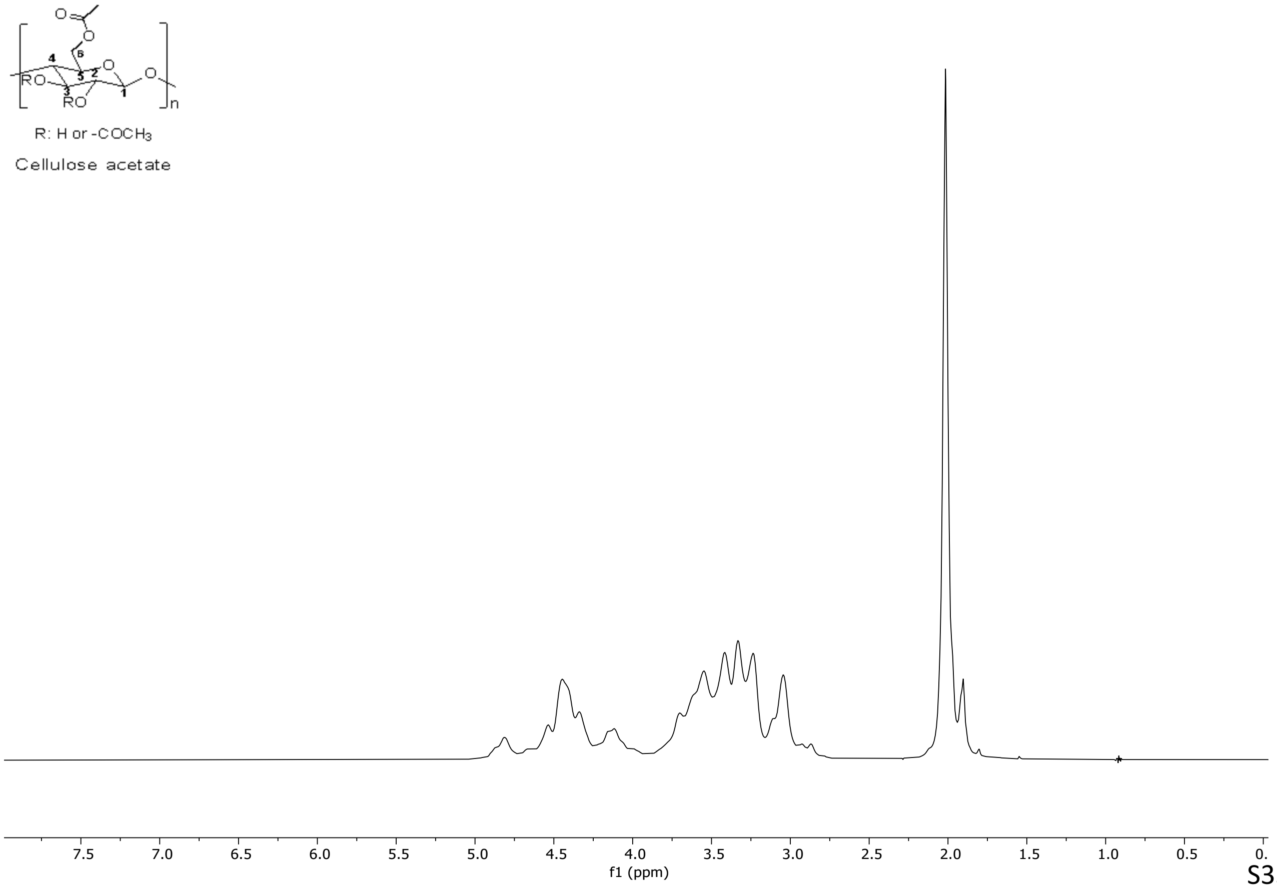

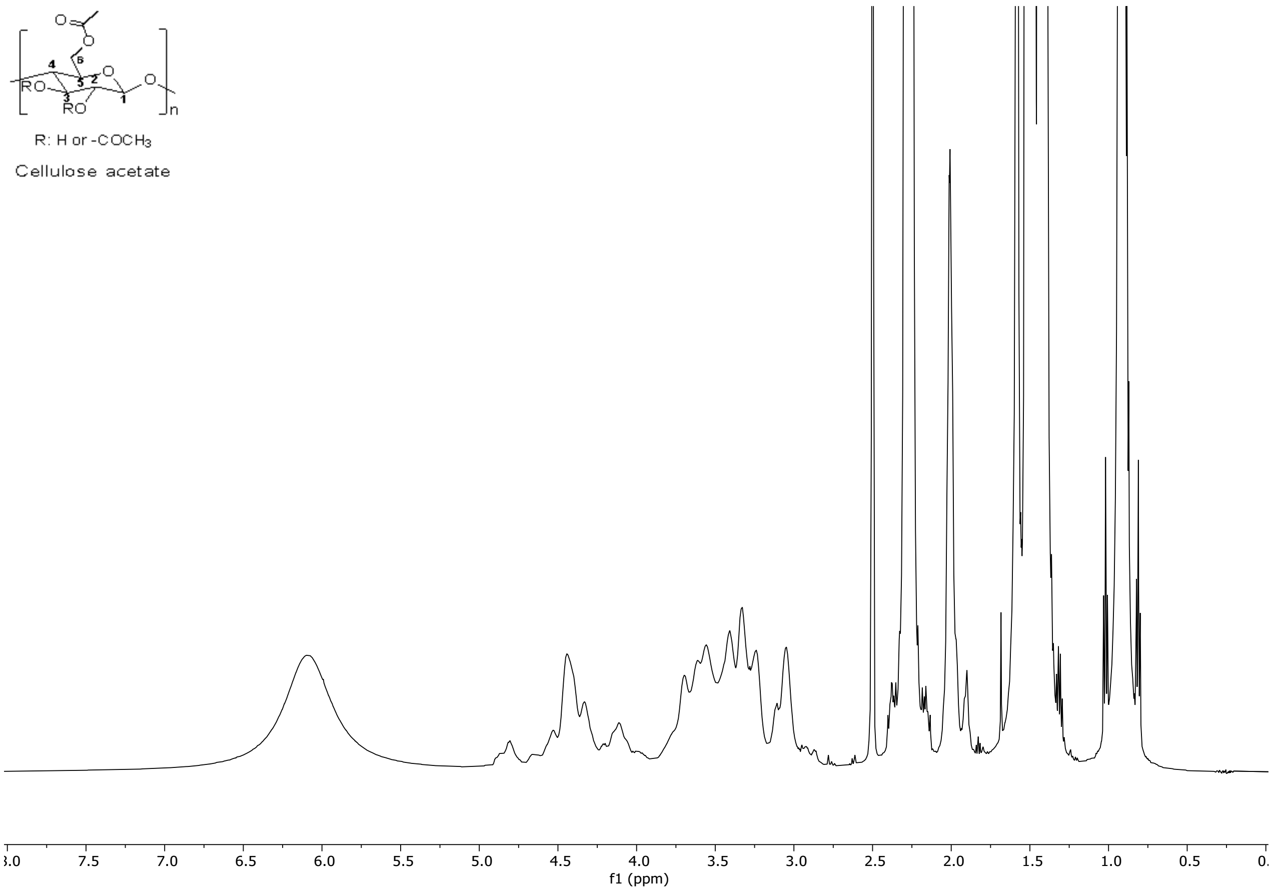

^{1}H and diffusion-edited ^{1}H spectra of **Cellulose acetate DS 0.72** in 20 w.% tetrabutylphosphonium acetate DMSO-d_6 electrolyte solution.

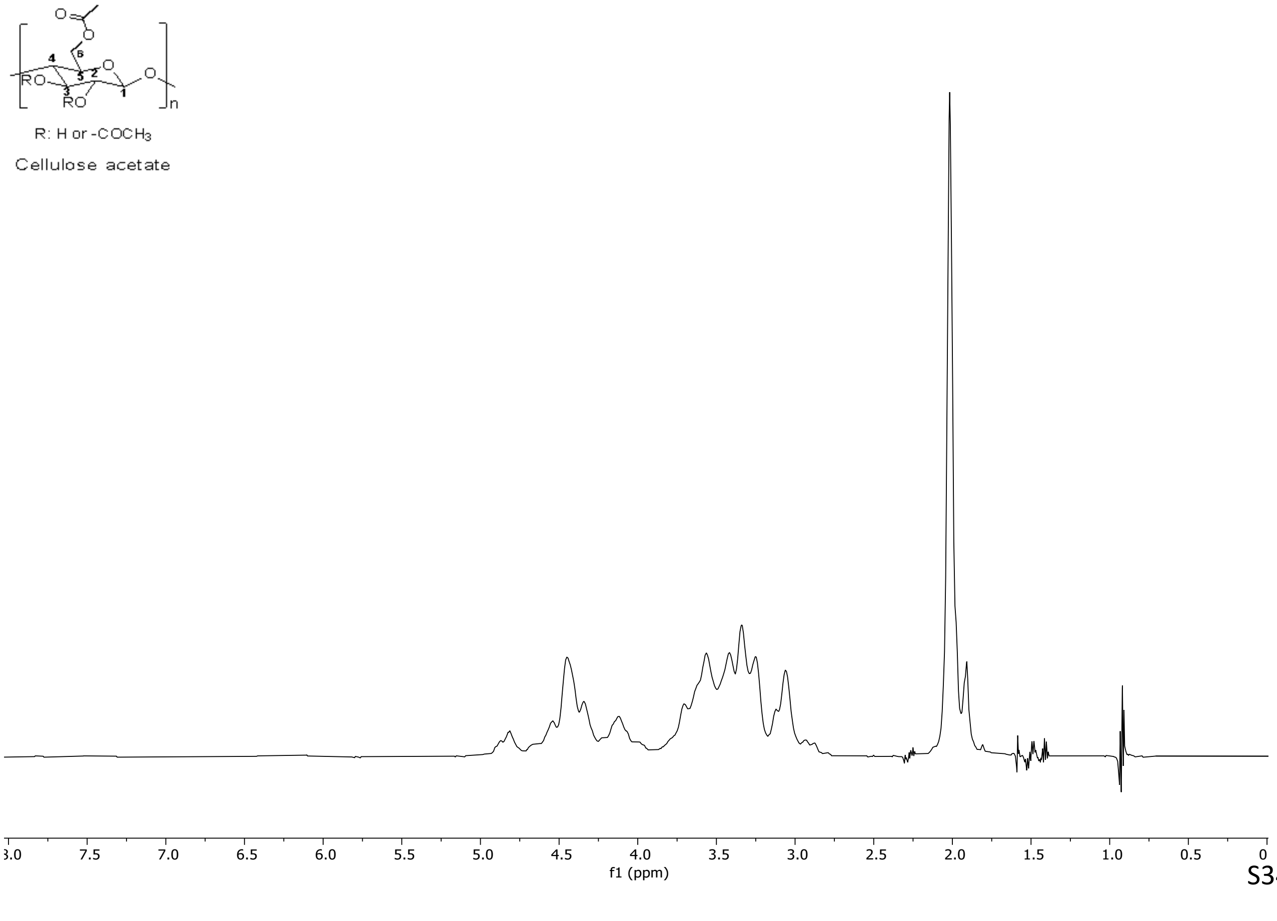

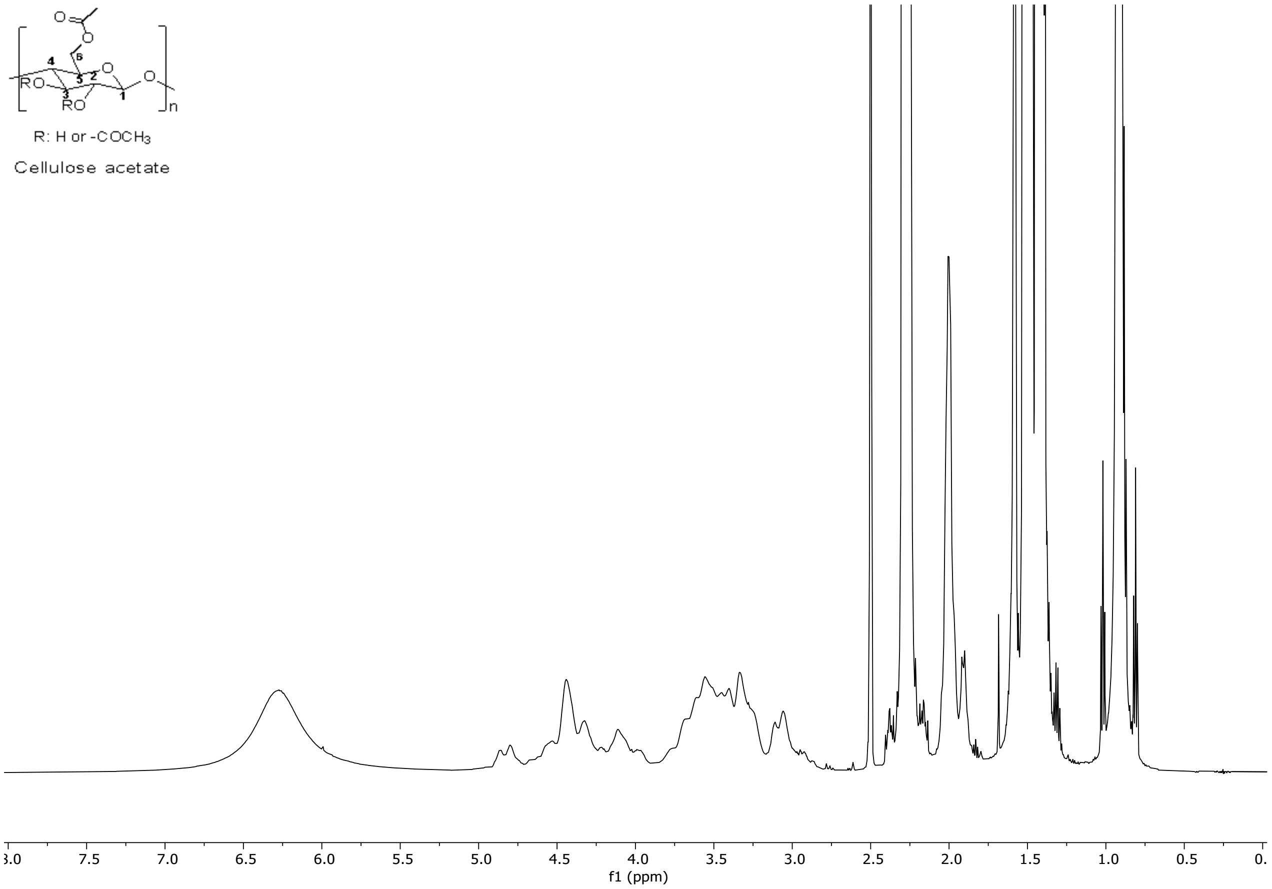

^{1}H and diffusion-edited ^{1}H spectra of **Cellulose acetate DS 1.07** in 20 w.% tetrabutylphosphonium acetate DMSO-d_6 electrolyte solution.

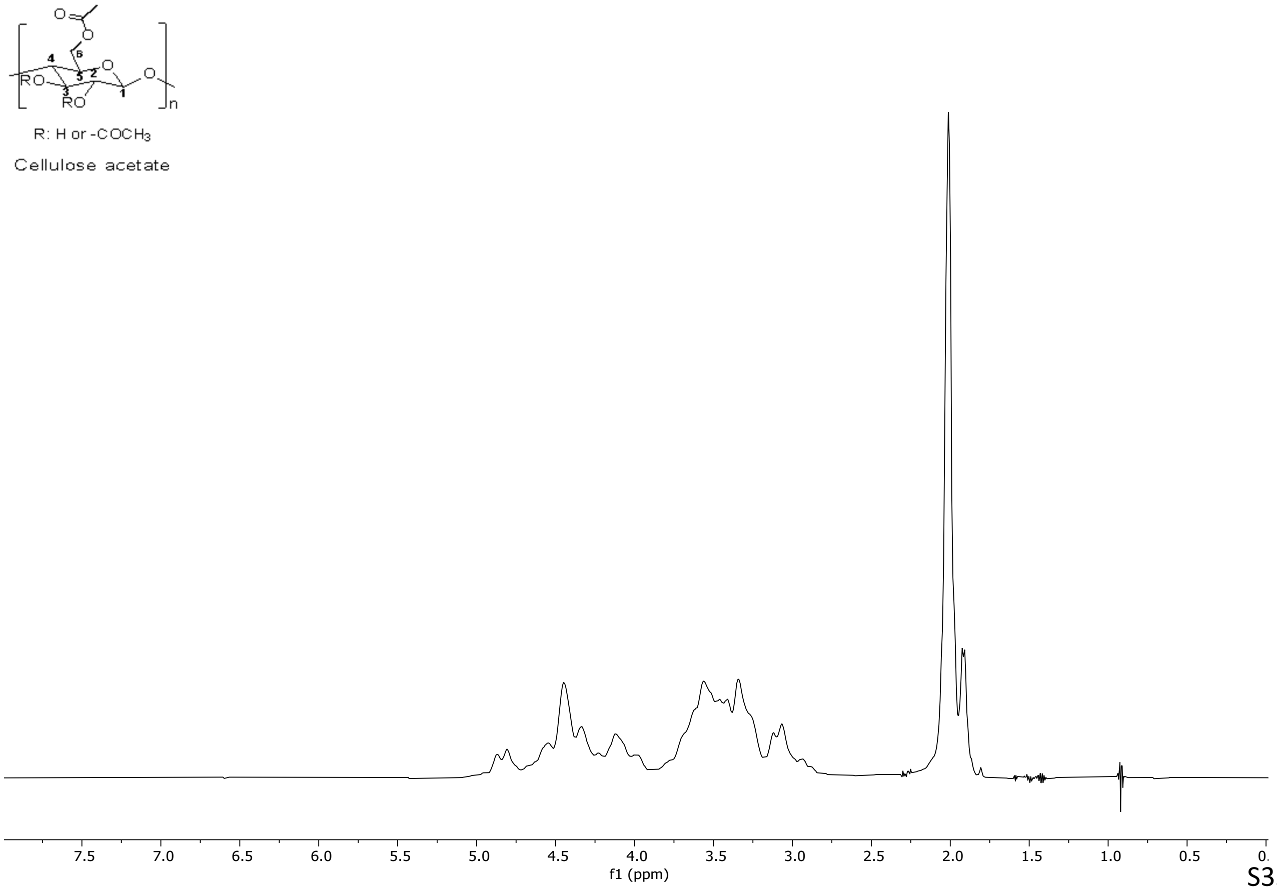

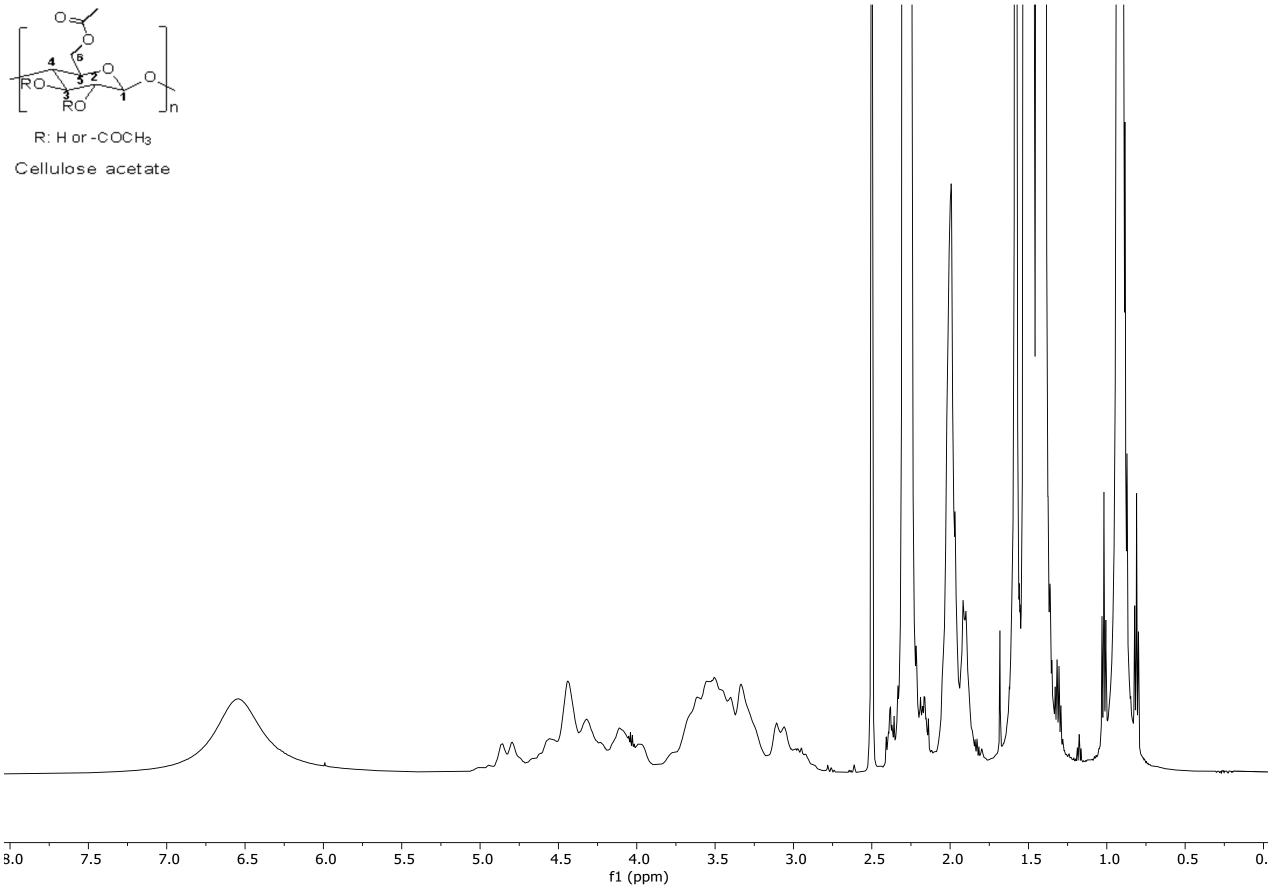

^{1}H and diffusion-edited ^{1}H spectra of **Cellulose acetate DS 1.29** in 20 w.% tetrabutylphosphonium acetate DMSO-d_6 electrolyte solution.

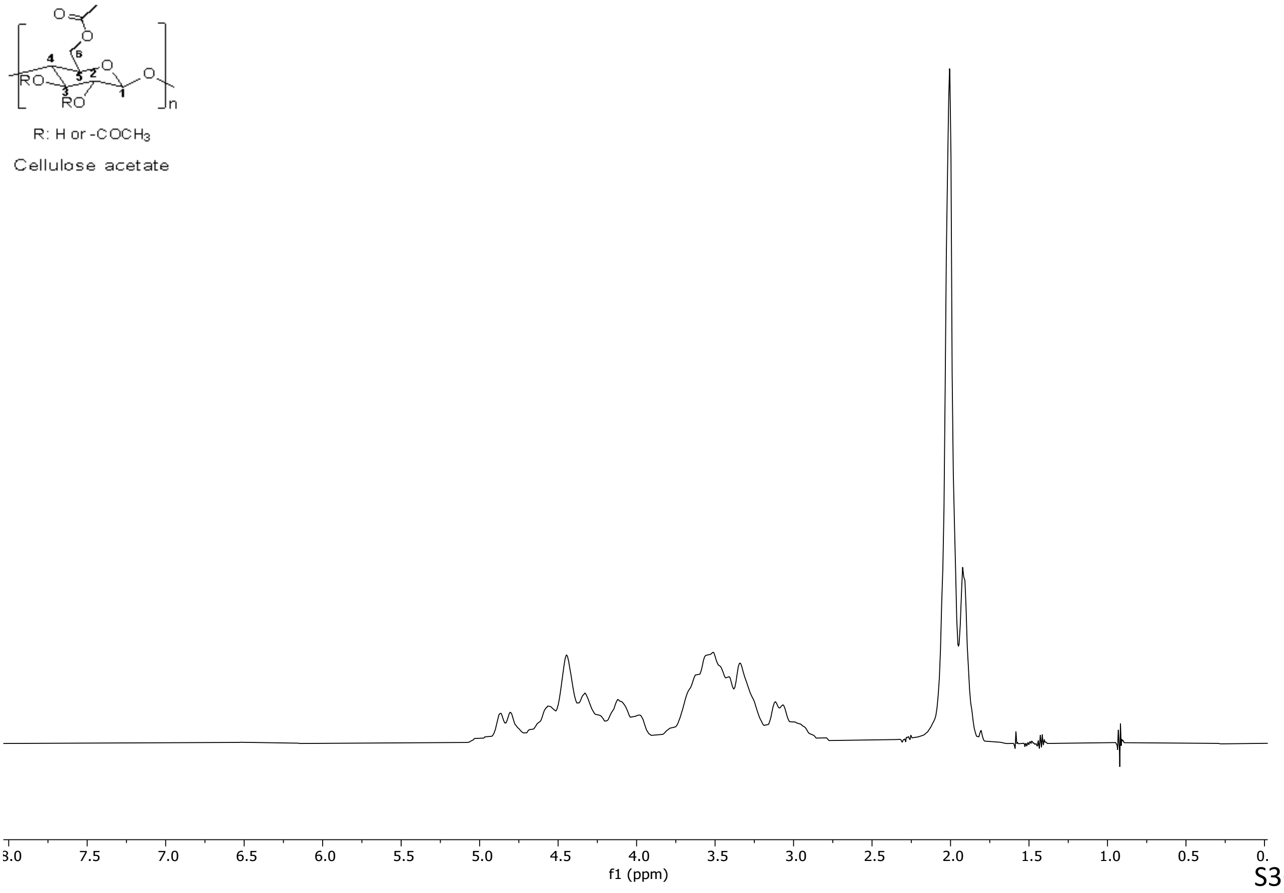

^{1}H and diffusion-edited ^{1}H spectra of **Cellulose acetate** samples from IL recyclability experiments in 20 w.% tetrabutylphosphonium acetate DMSO-

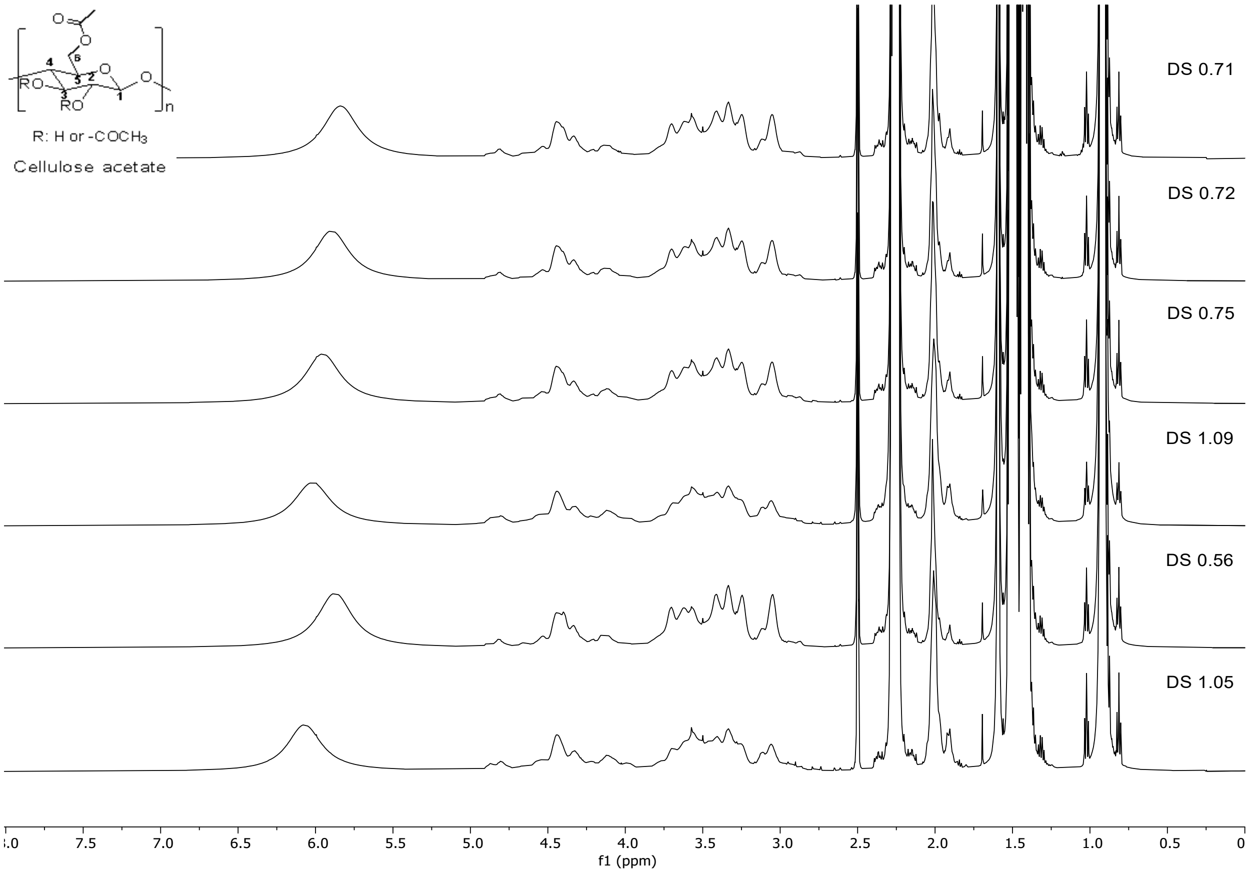

d_6 electrolyte solution.

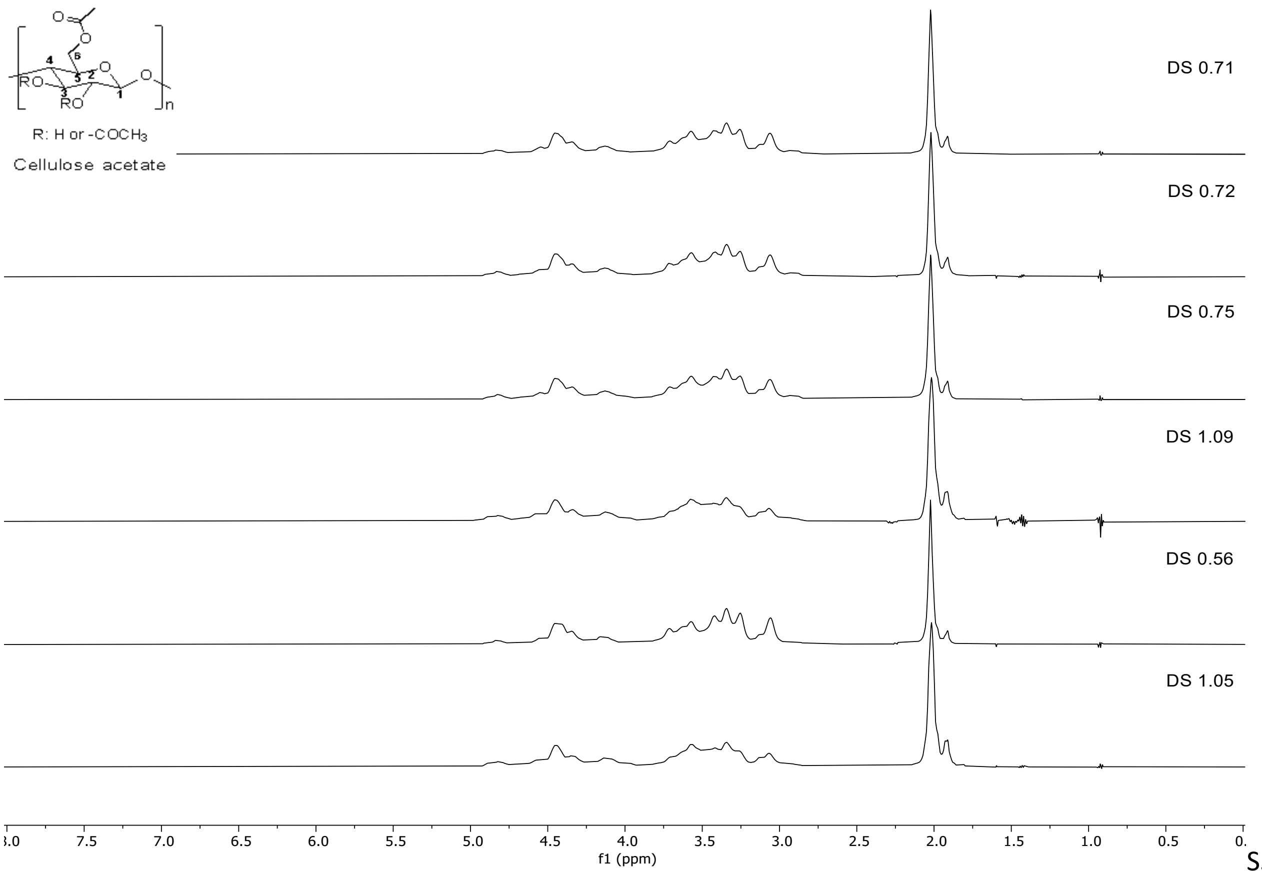

^{1}H and diffusion-edited ^{1}H spectra of **Cellulose acetate** samples from enhancement experiments in 20 w.% tetrabutylphosphonium acetate DMSO-

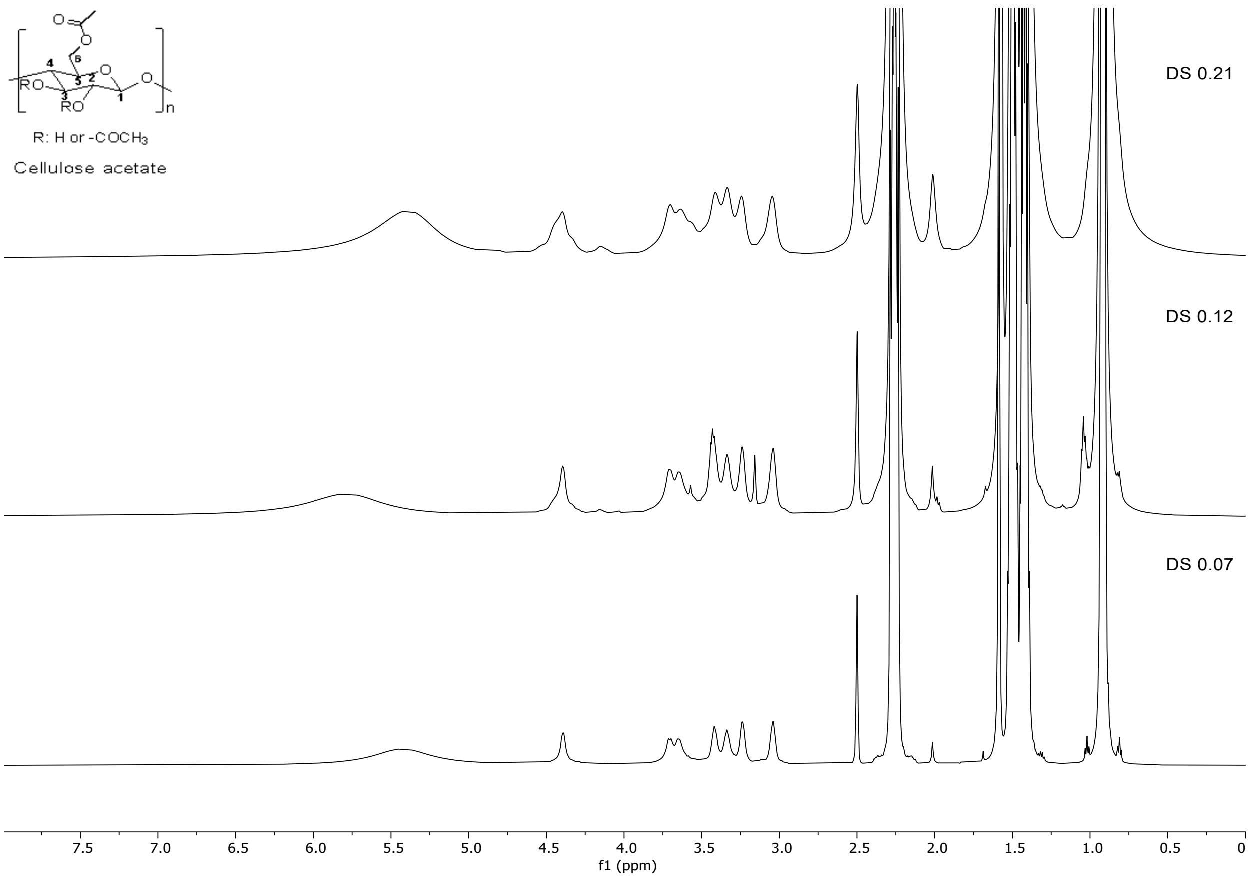

d_6 electrolyte solution.

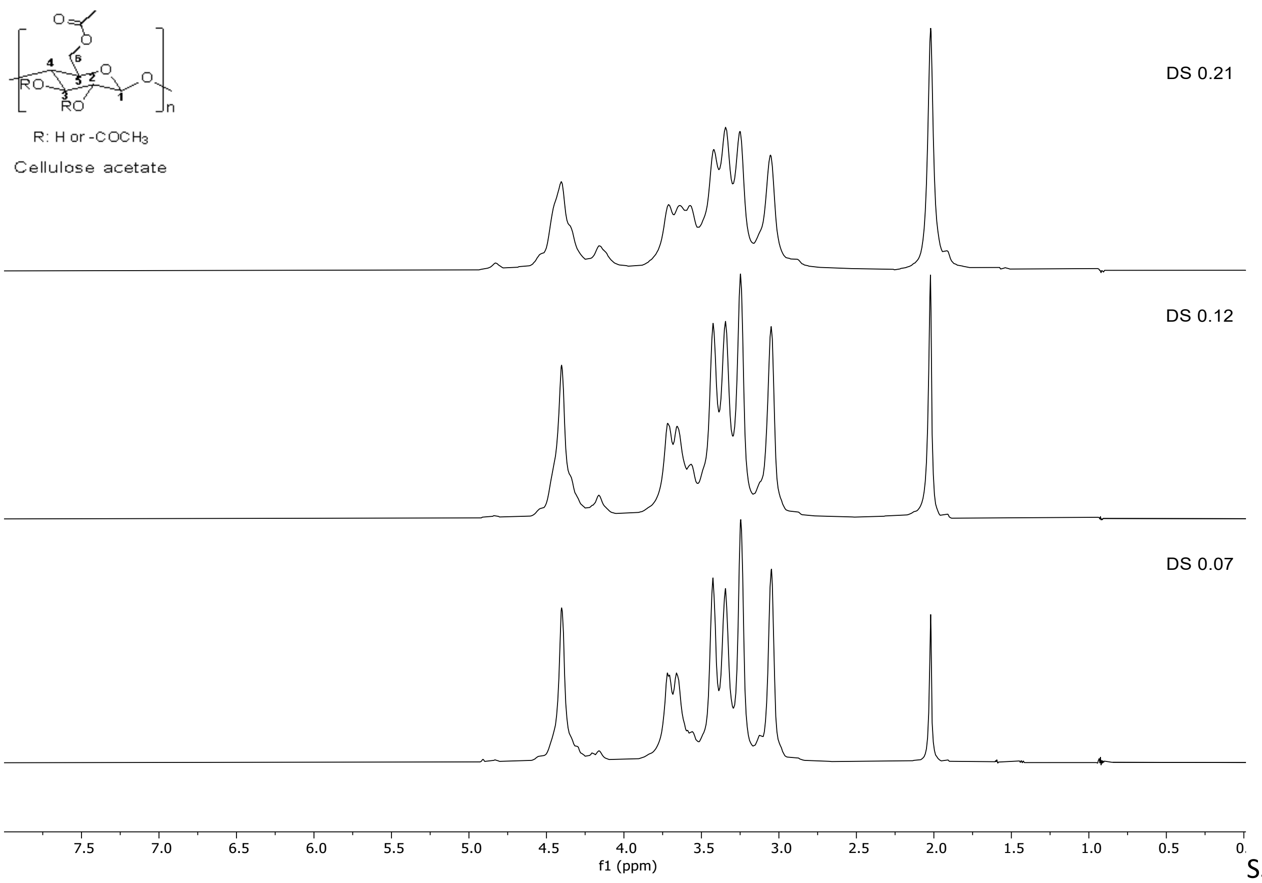

^1H and diffusion-edited ^1H spectra of **Cellulose acetate** samples from competitive reactions with ethanol in 20 w.% tetrabutylphosphonium acetate DMSO-d_6 electrolyte solution.

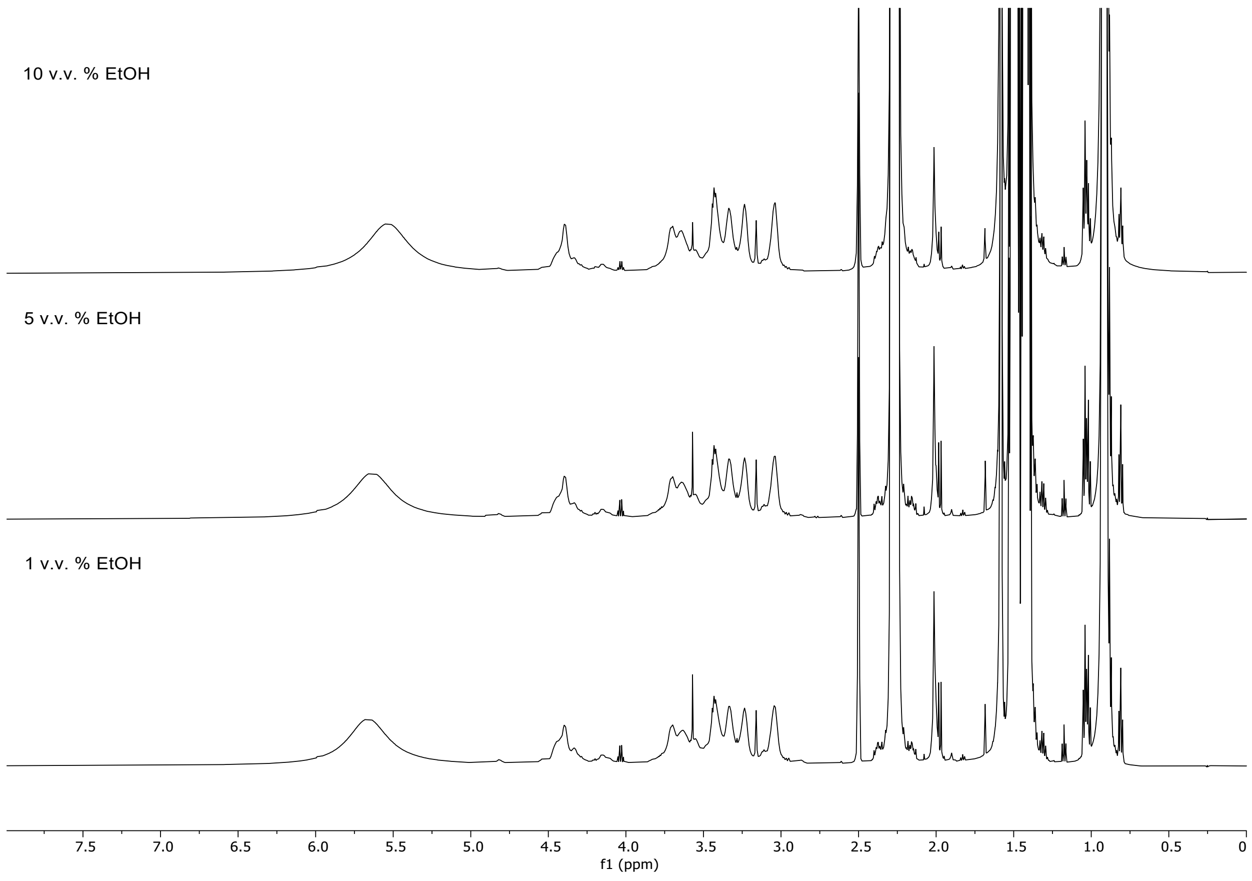
10 v.v. % EtOH
5 v.v. % EtOH
1 v.v. % EtOH
7.5 7.0 6.5 6.0 5.5 5.0 4.5 4.0 3.5 3.0 2.5 2.0 1.5 1.0 0.5 0
f1 (ppm)

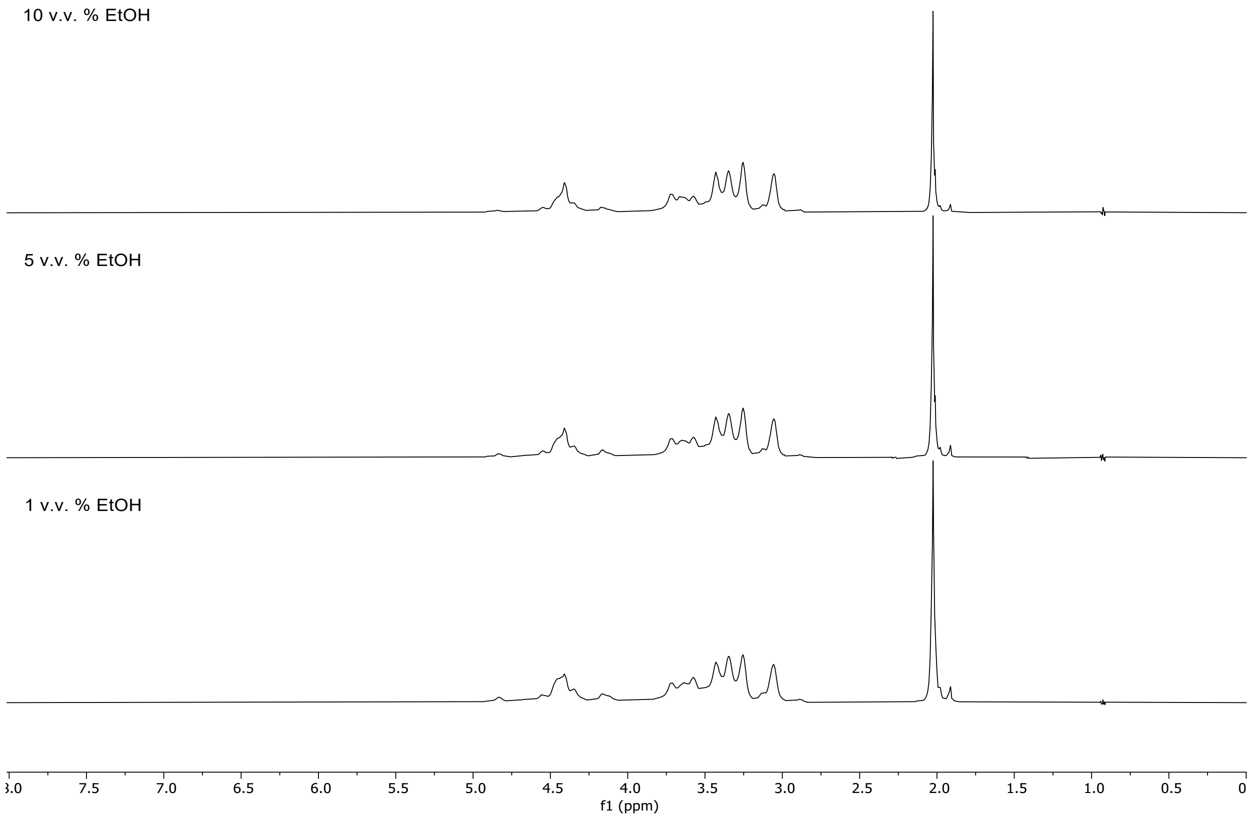
10 v.v. % EtOH
5 v.v. % EtOH
1 v.v. % EtOH
7.5 7.0 6.5 6.0 5.5 5.0 4.5 4.0 3.5 3.0 2.5 2.0 1.5 1.0 0.5 0
f1 (ppm)

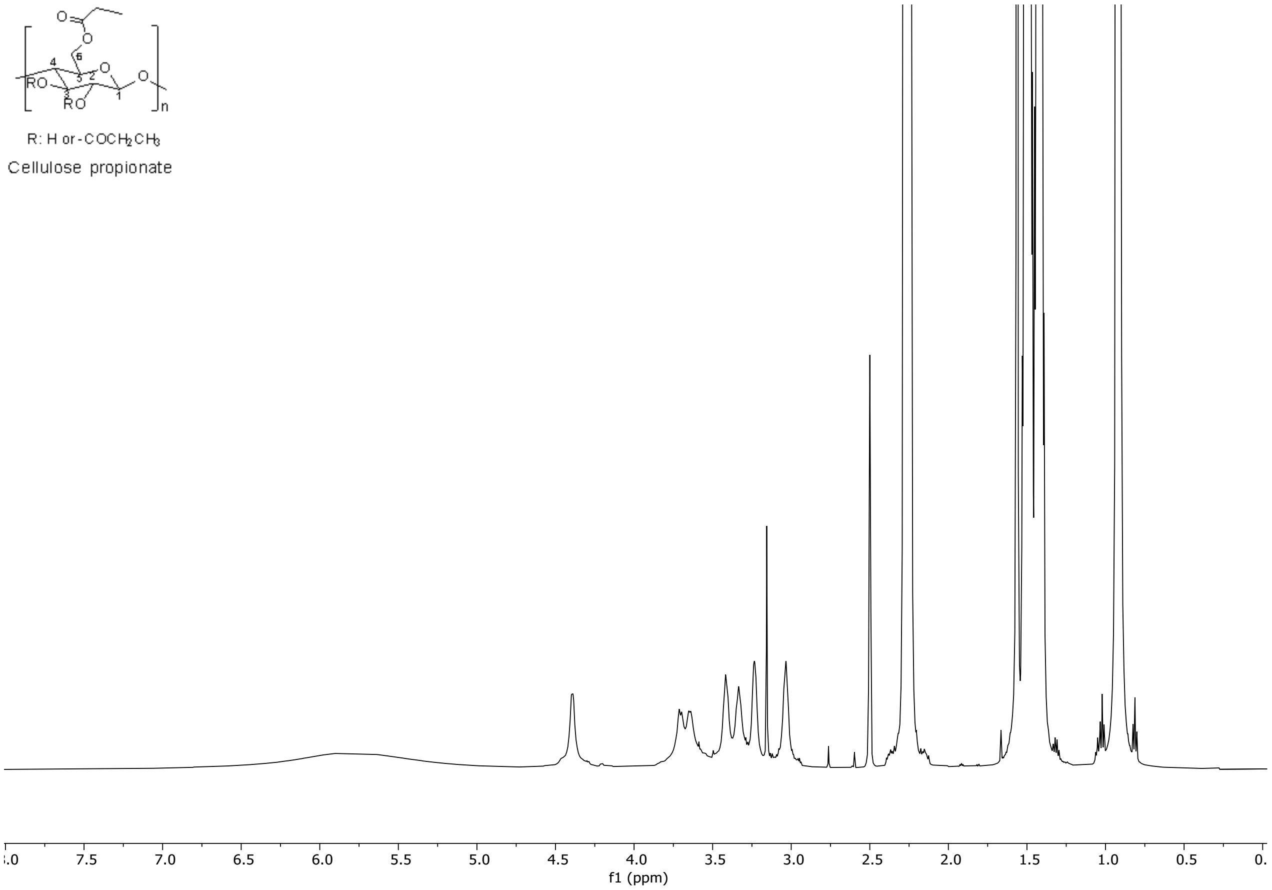

^{1}H and diffusion-edited ^{1}H spectra of **Cellulose propionate DS 0.06** in 20 w.% tetrabutylphosphonium acetate DMSO-d_6 electrolyte solution.

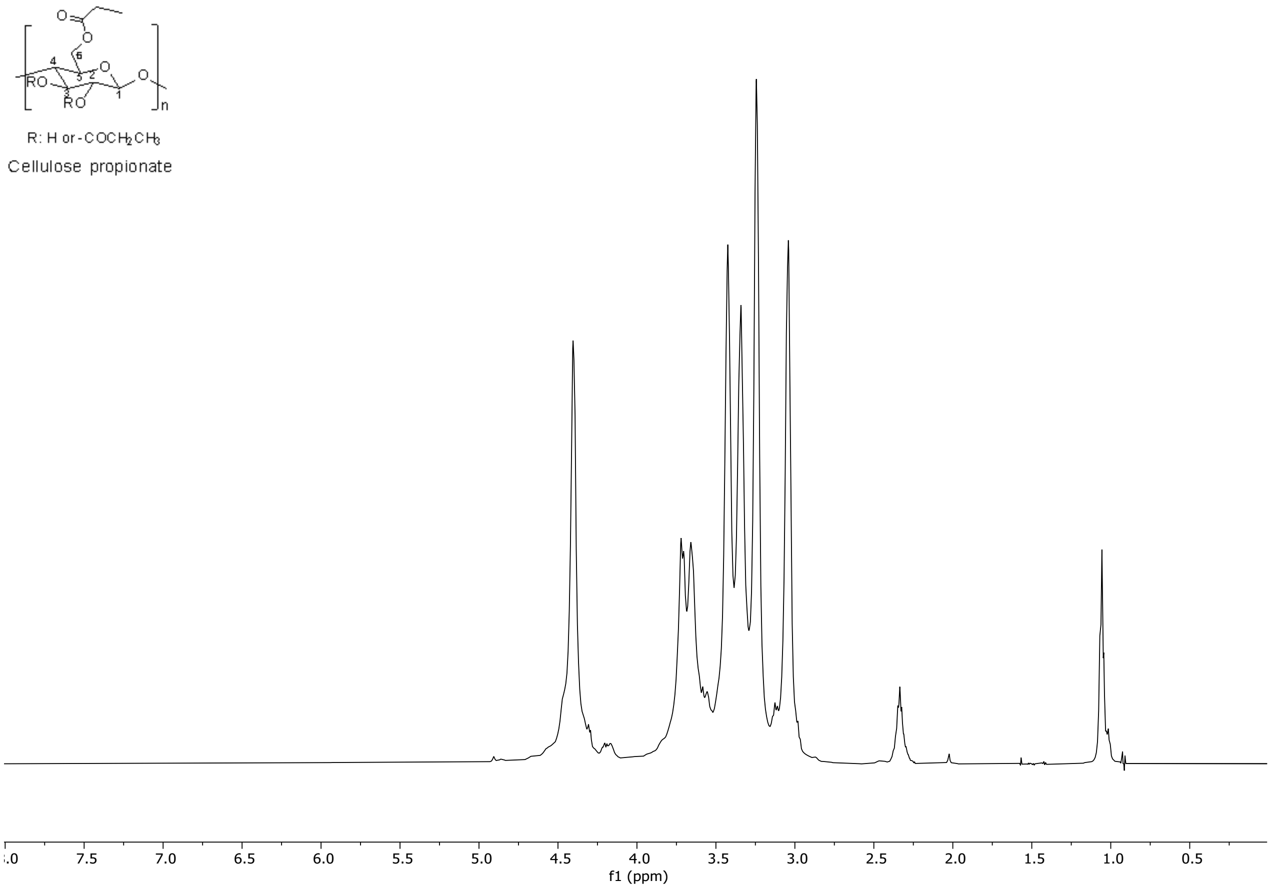

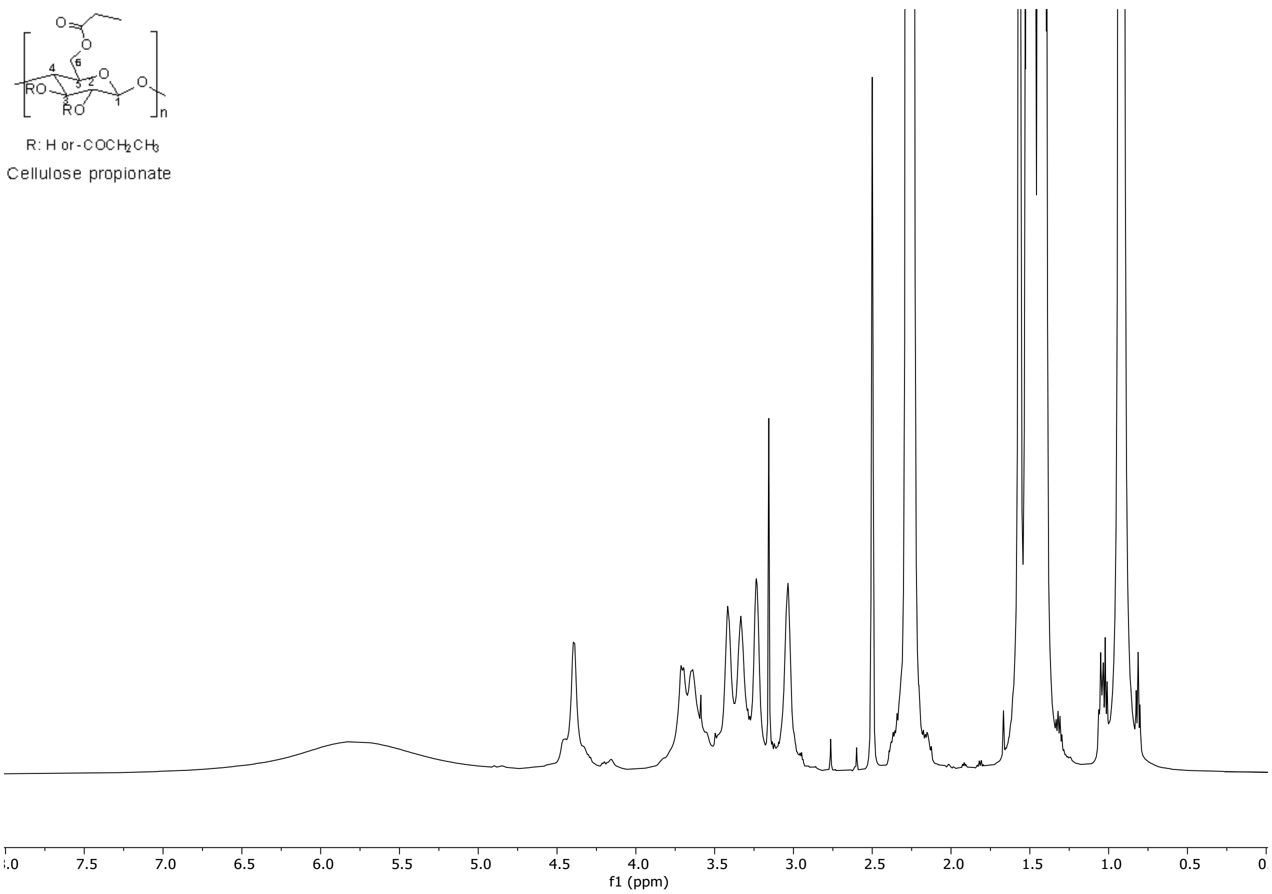

^{1}H and diffusion-edited ^{1}H spectra of **Cellulose propionate DS 0.10** in 20 w.% tetrabutylphosphonium acetate DMSO-d_6 electrolyte solution.

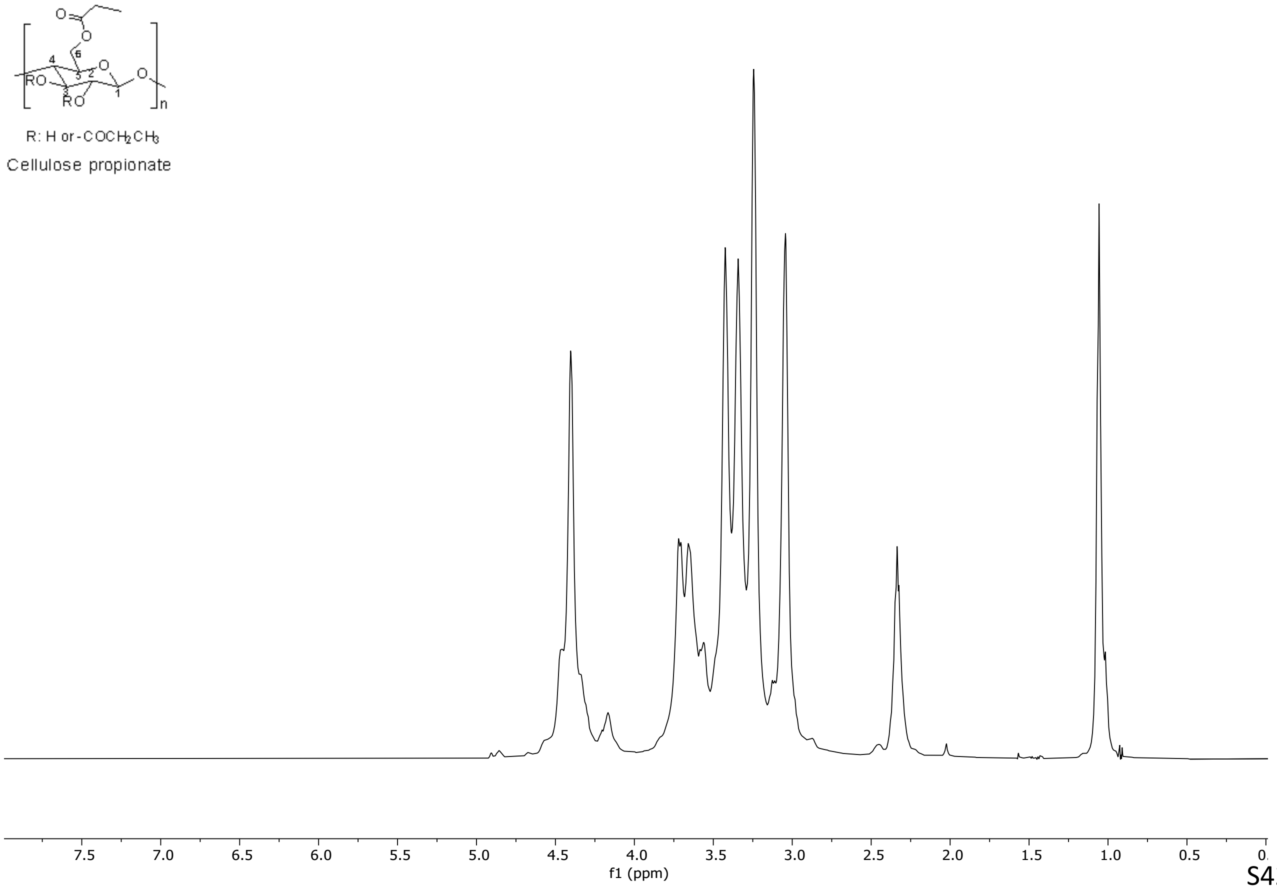

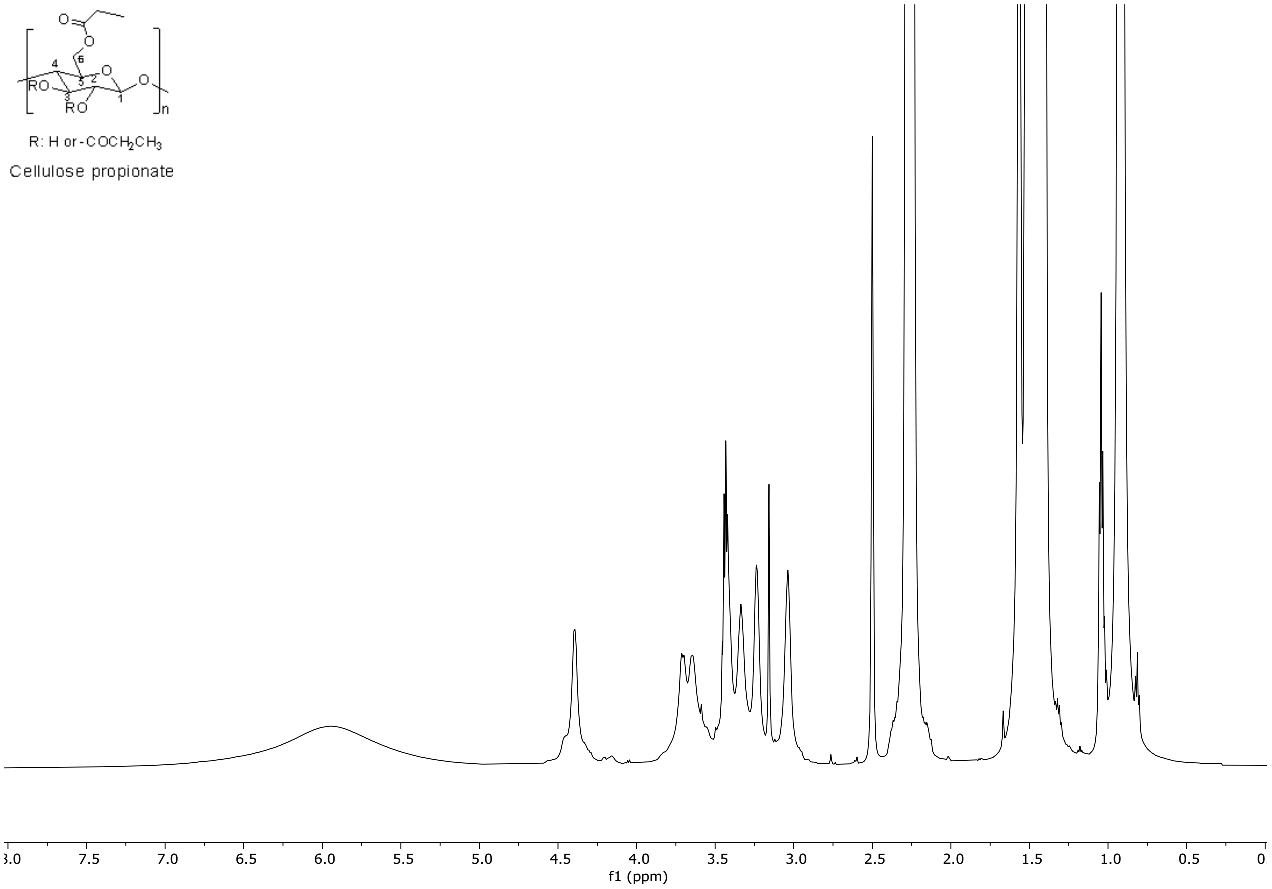

^{1}H and diffusion-edited ^{1}H spectra of **Cellulose propionate DS 0.08** in 20 w.% tetrabutylphosphonium acetate DMSO-d_6 electrolyte solution.

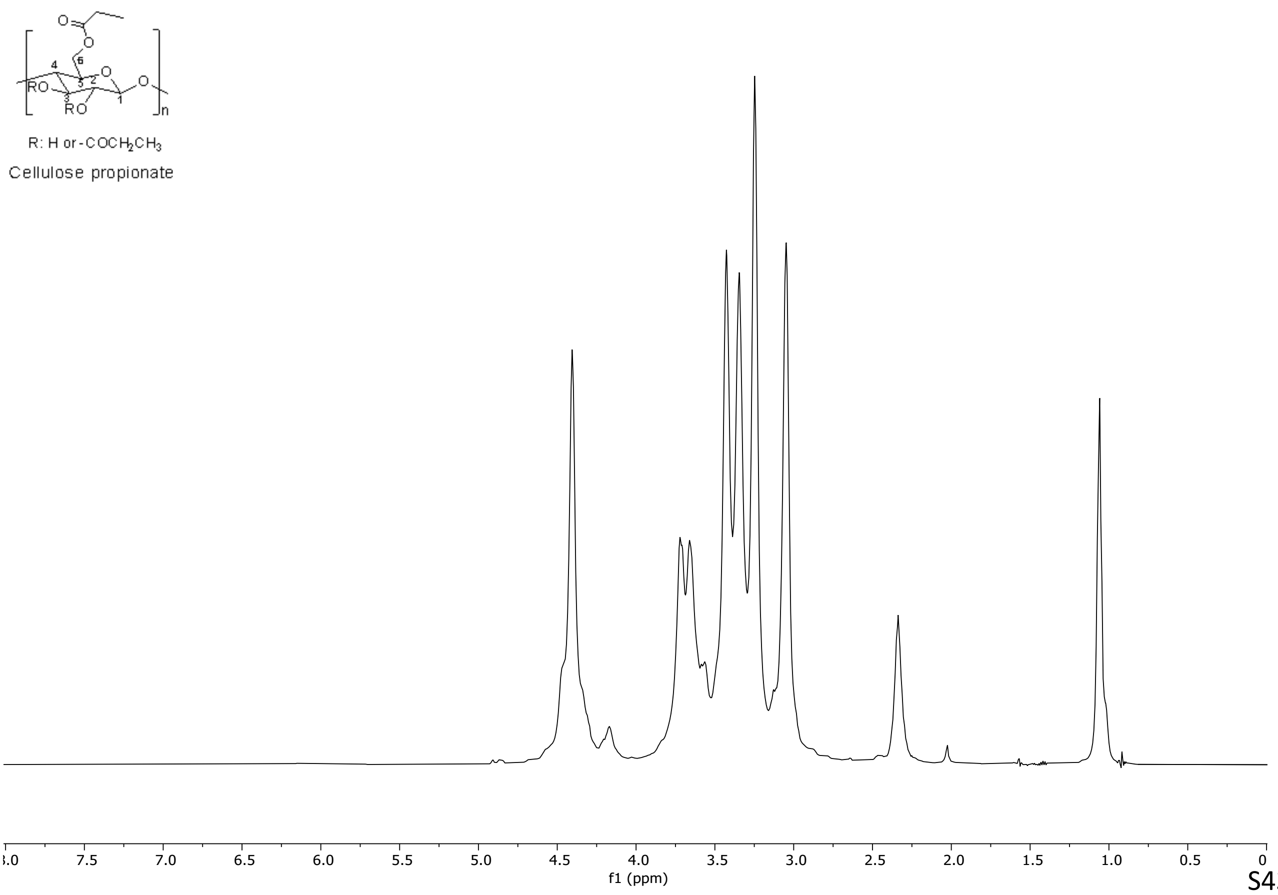

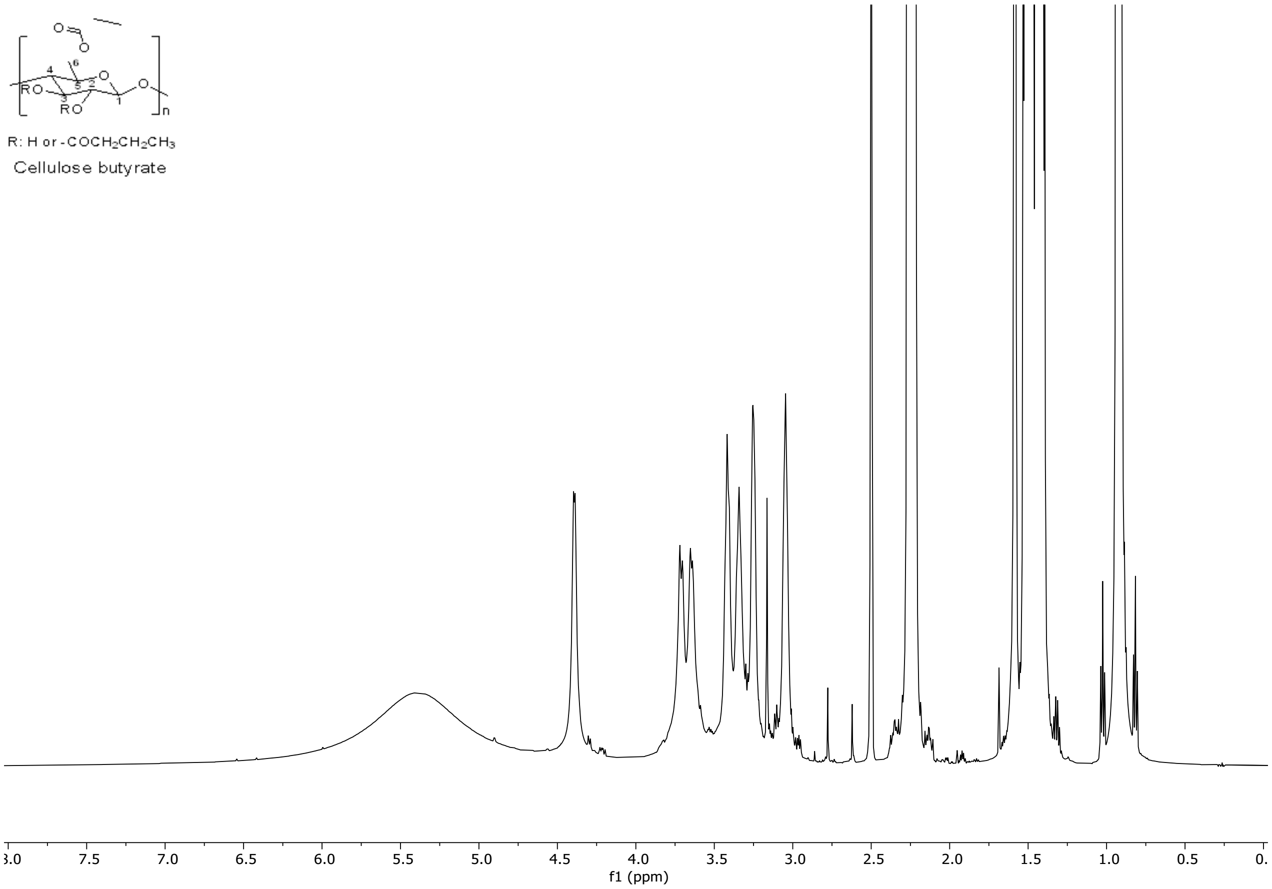

^{1}H and diffusion-edited ^{1}H spectra of **Cellulose butyrate DS 0.04** in 20 w.% tetrabutylphosphonium acetate DMSO-d_6 electrolyte solution.

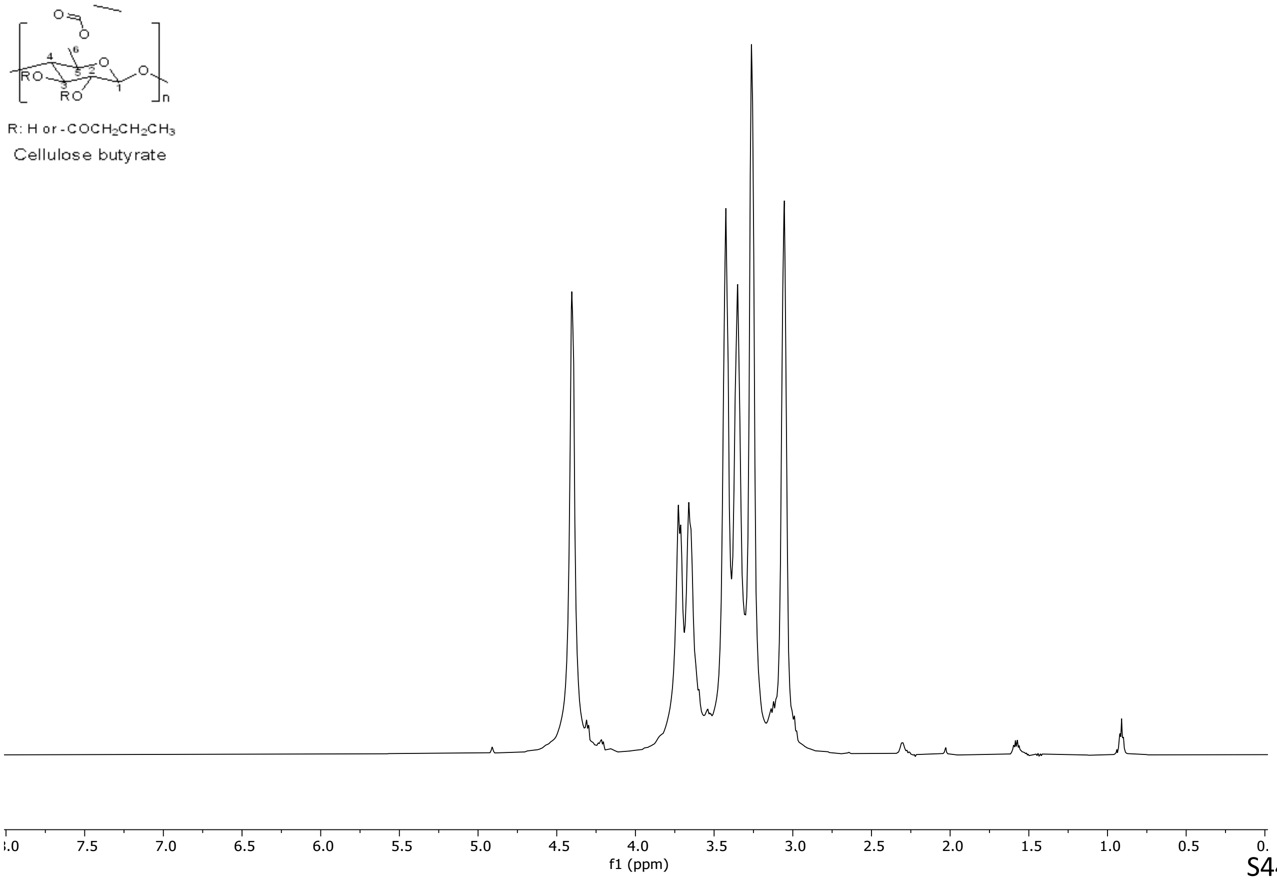

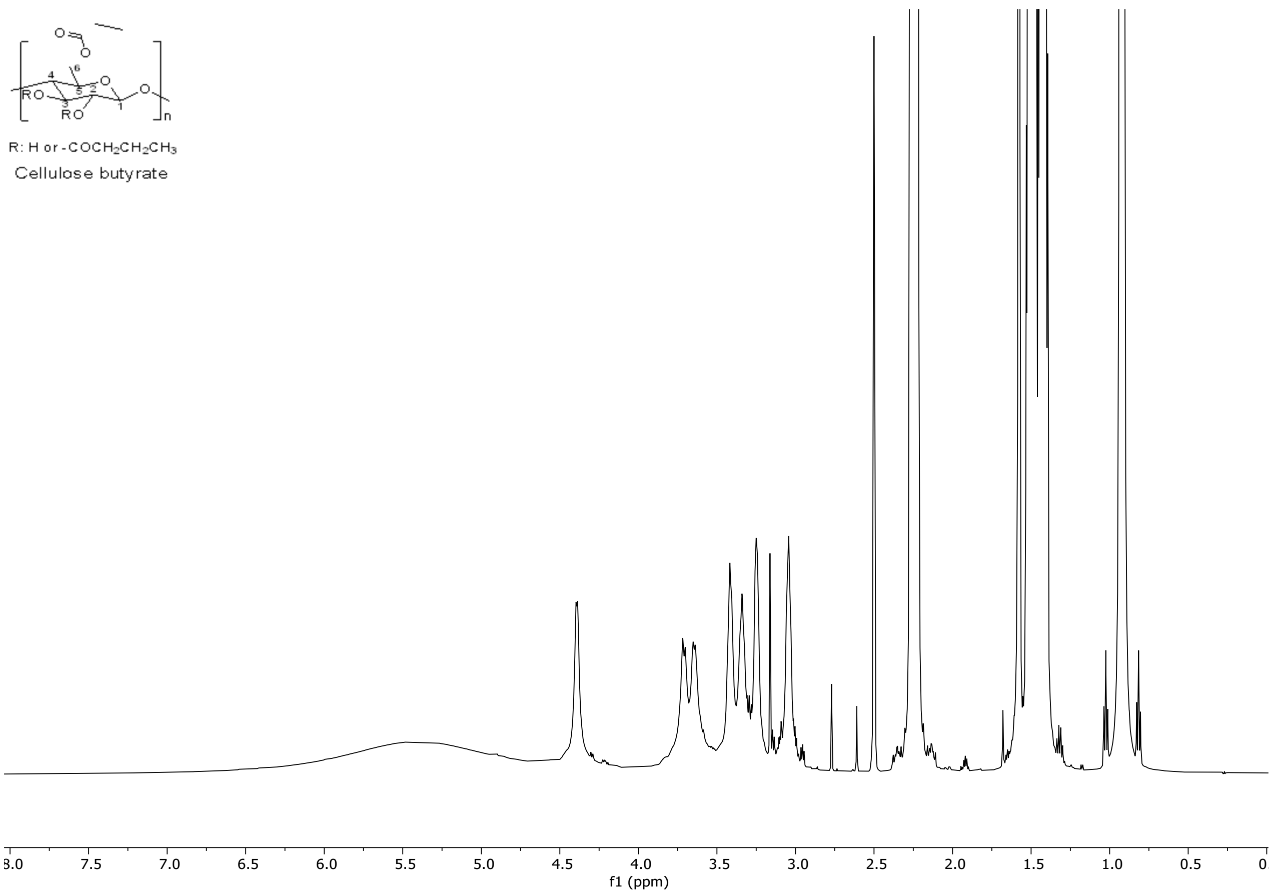

^{1}H and diffusion-edited ^{1}H spectra of **Cellulose butyrate DS 0.05** in 20 w.% tetrabutylphosphonium acetate DMSO-d_6 electrolyte solution.

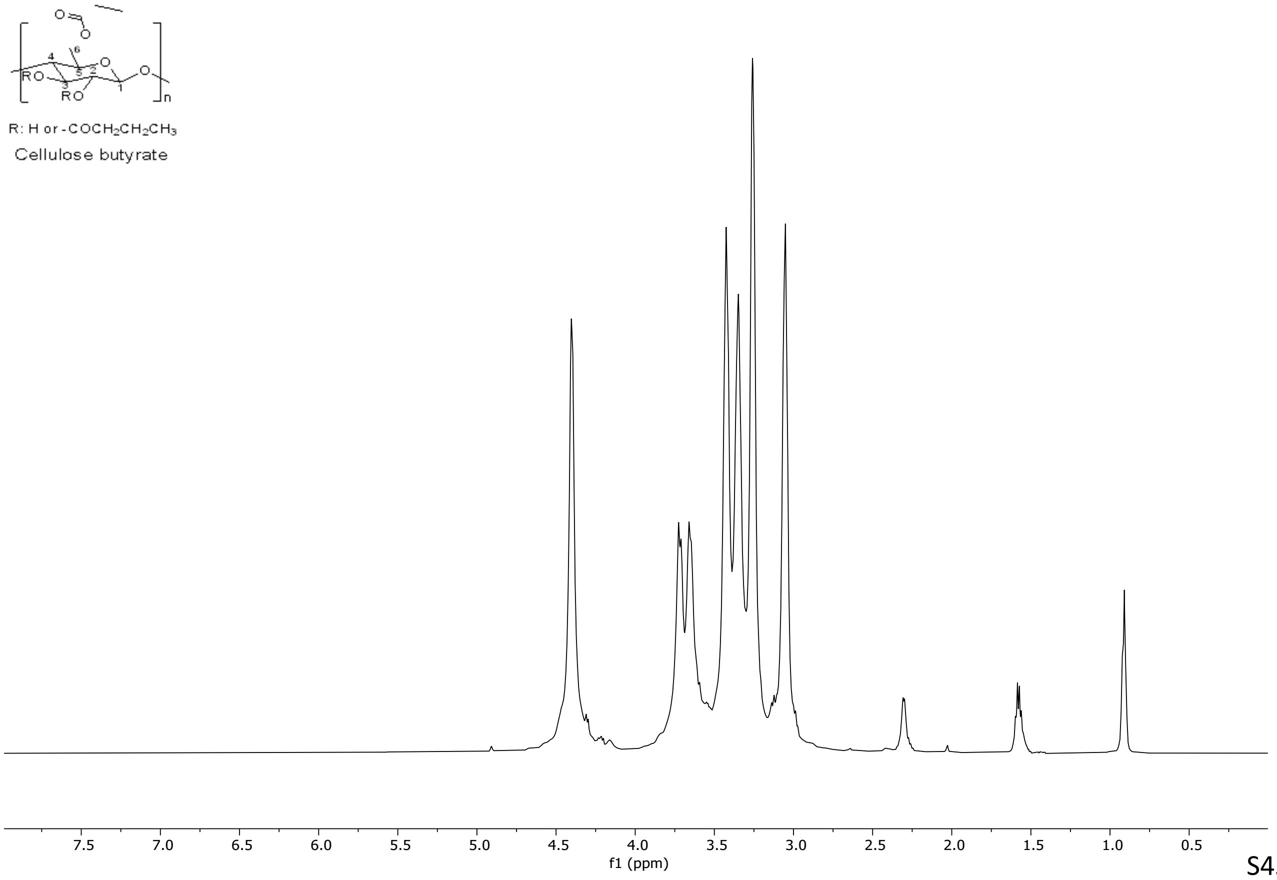

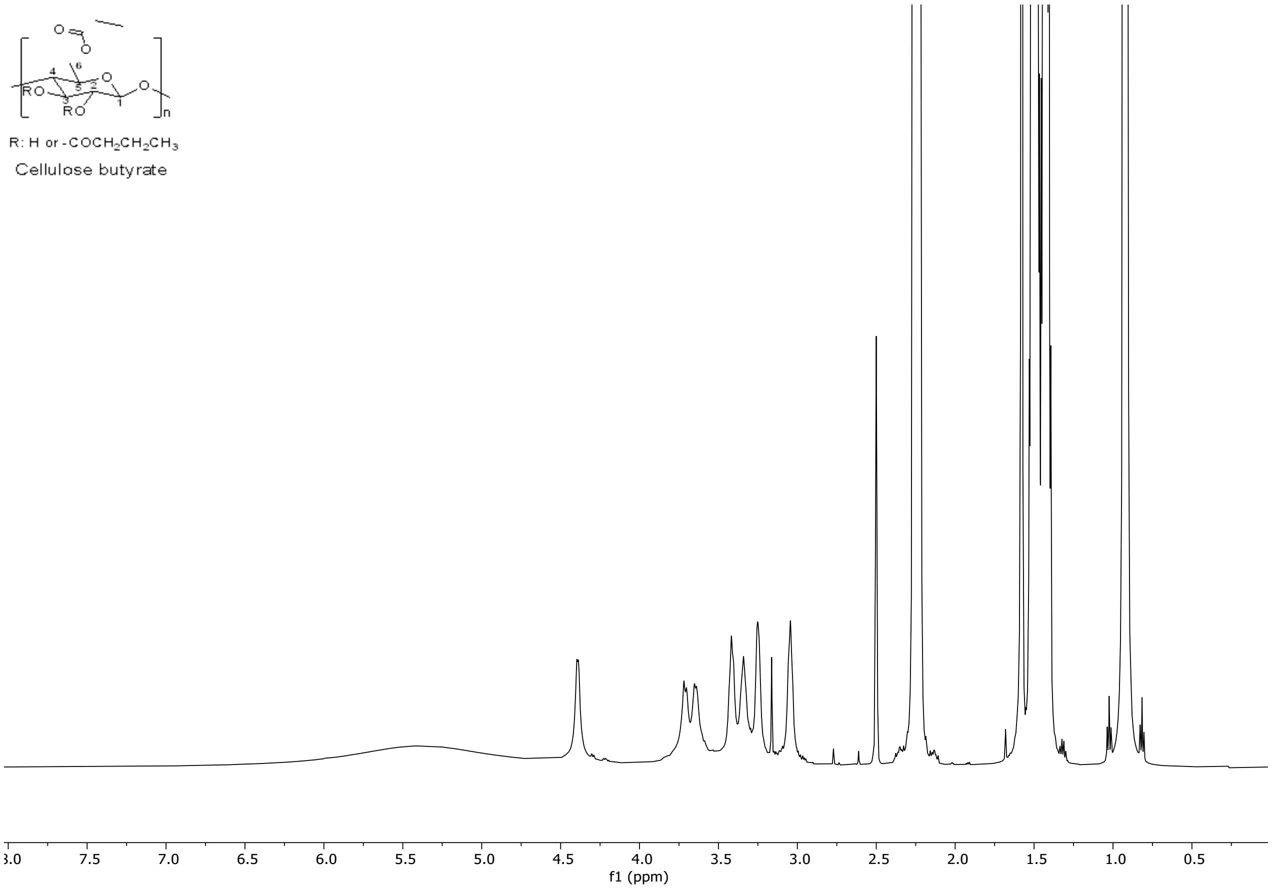

^{1}H and diffusion-edited ^{1}H spectra of **Cellulose butyrate DS 0.05** in 20 w.% tetrabutylphosphonium acetate DMSO-d_6 electrolyte solution.

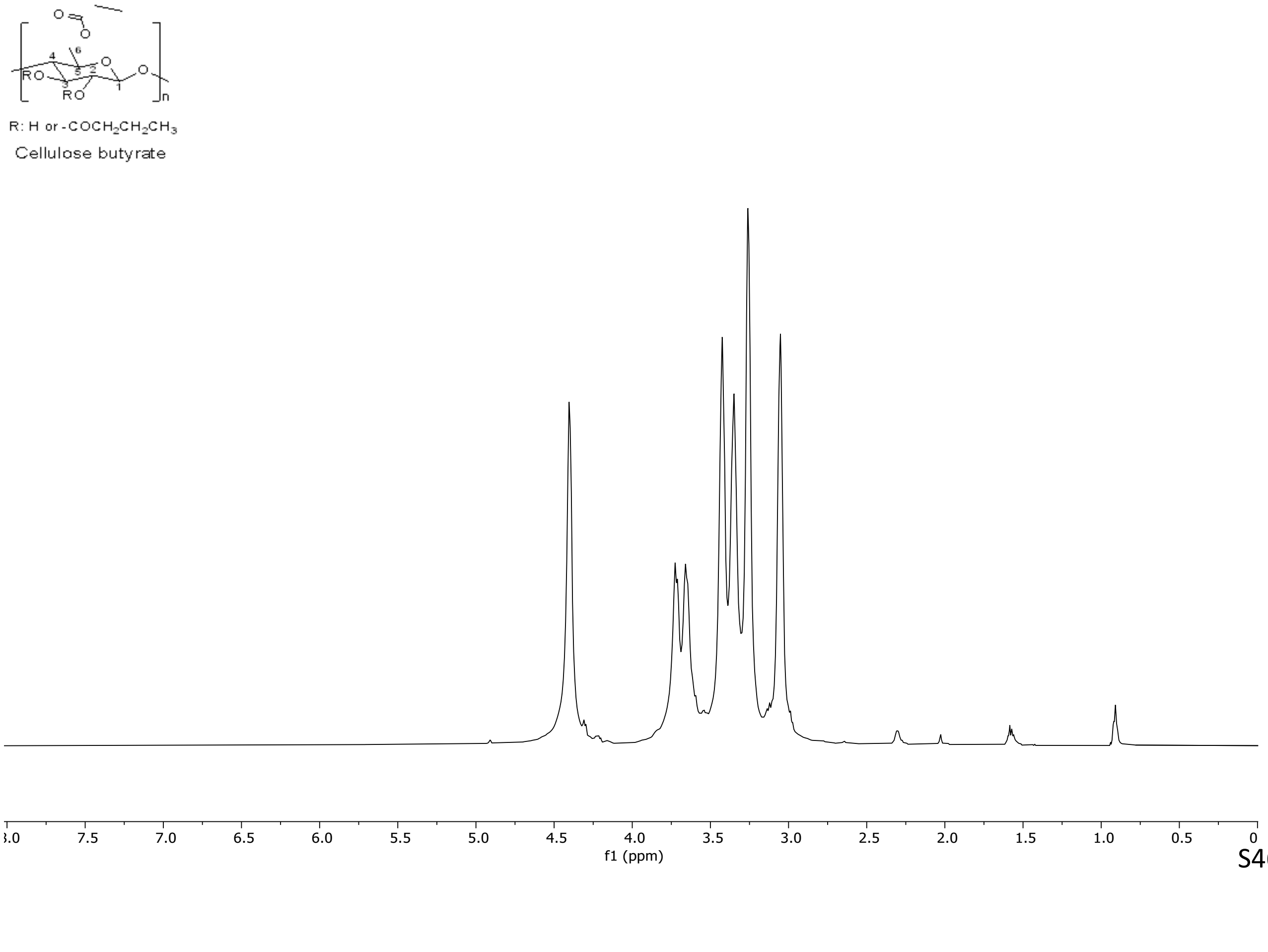

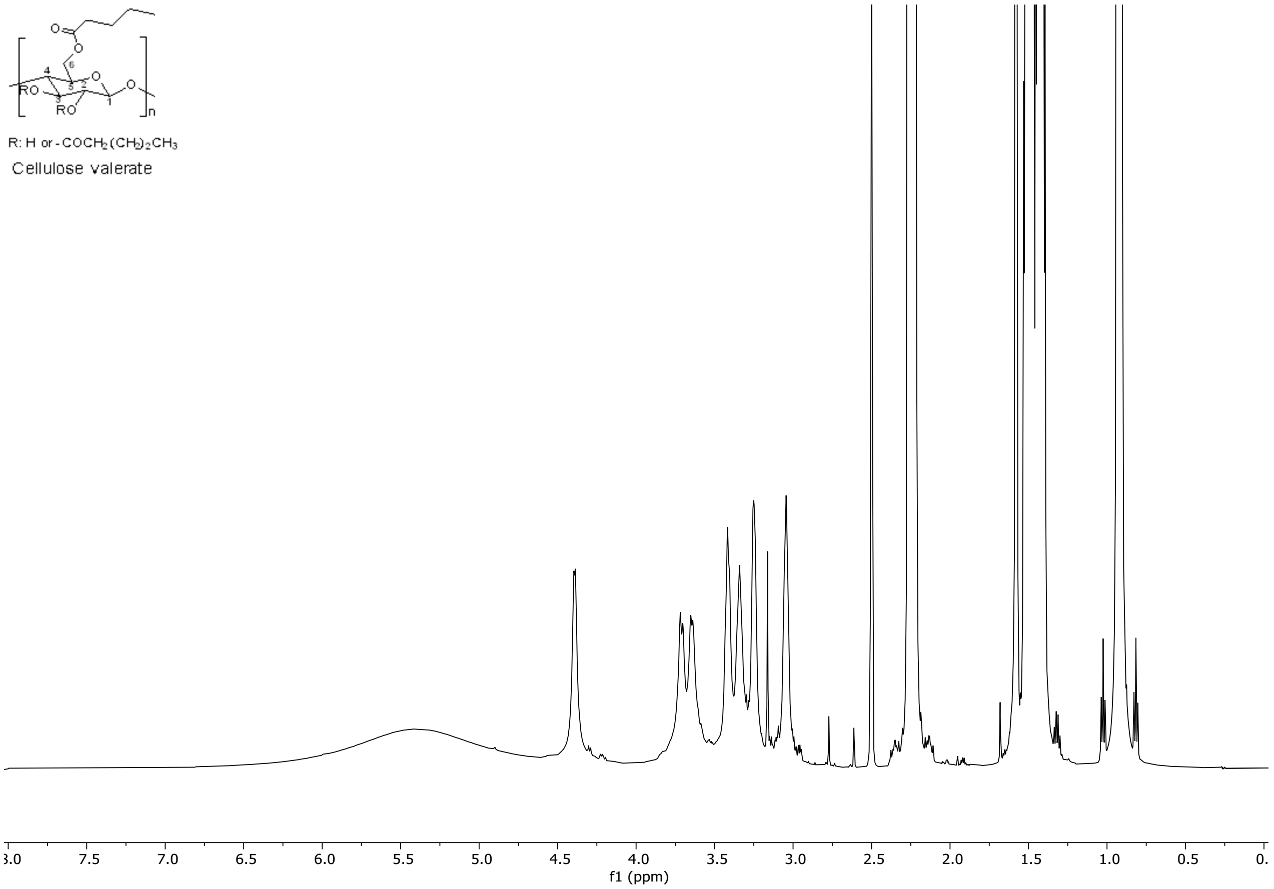

^{1}H and diffusion-edited ^{1}H spectra of **Cellulose valerate DS 0.04** in 20 w.% tetrabutylphosphonium acetate DMSO-d_6 electrolyte solution.

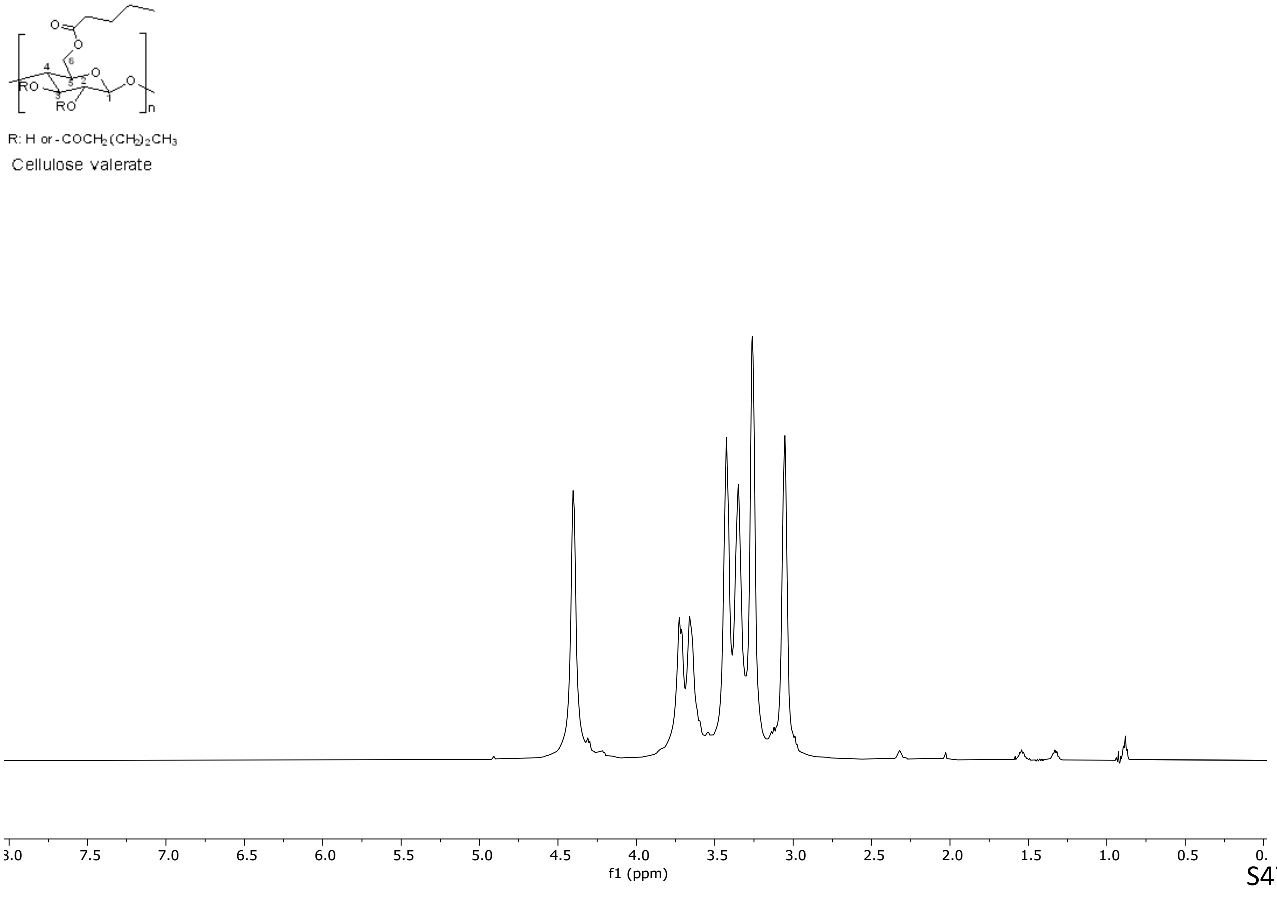

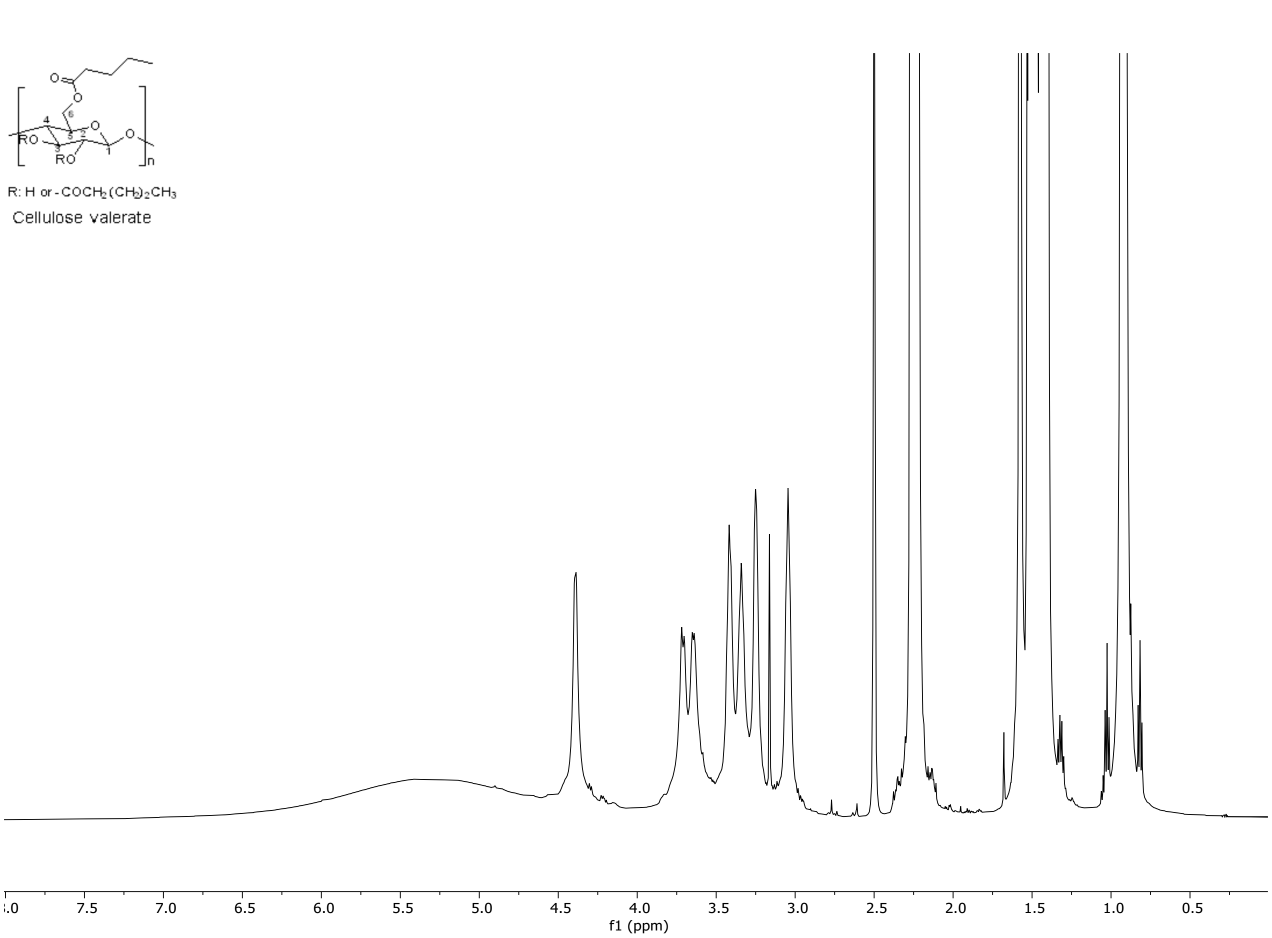

^{1}H and diffusion-edited ^{1}H spectra of **Cellulose valerate DS 0.05** in 20 w.% tetrabutylphosphonium acetate DMSO-d_6 electrolyte solution.

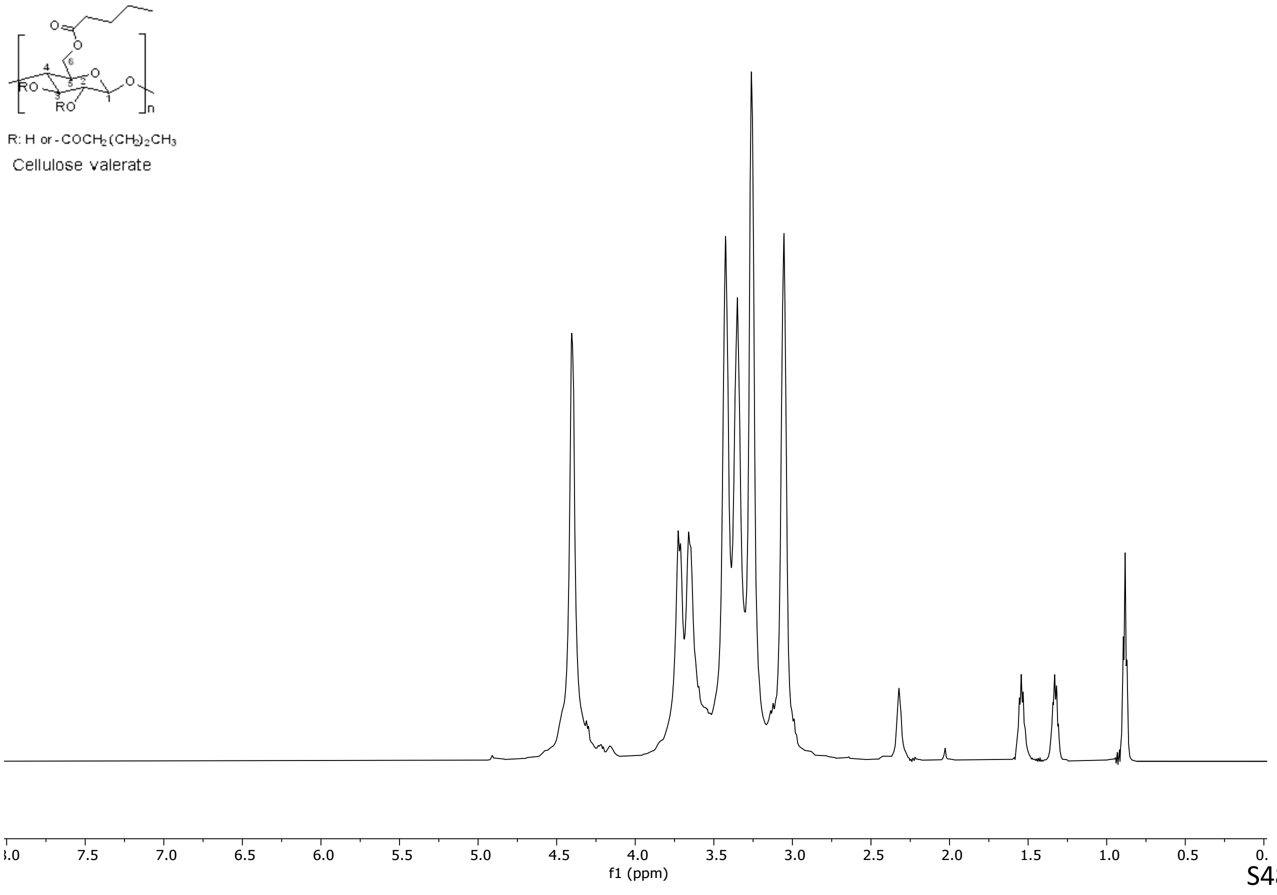

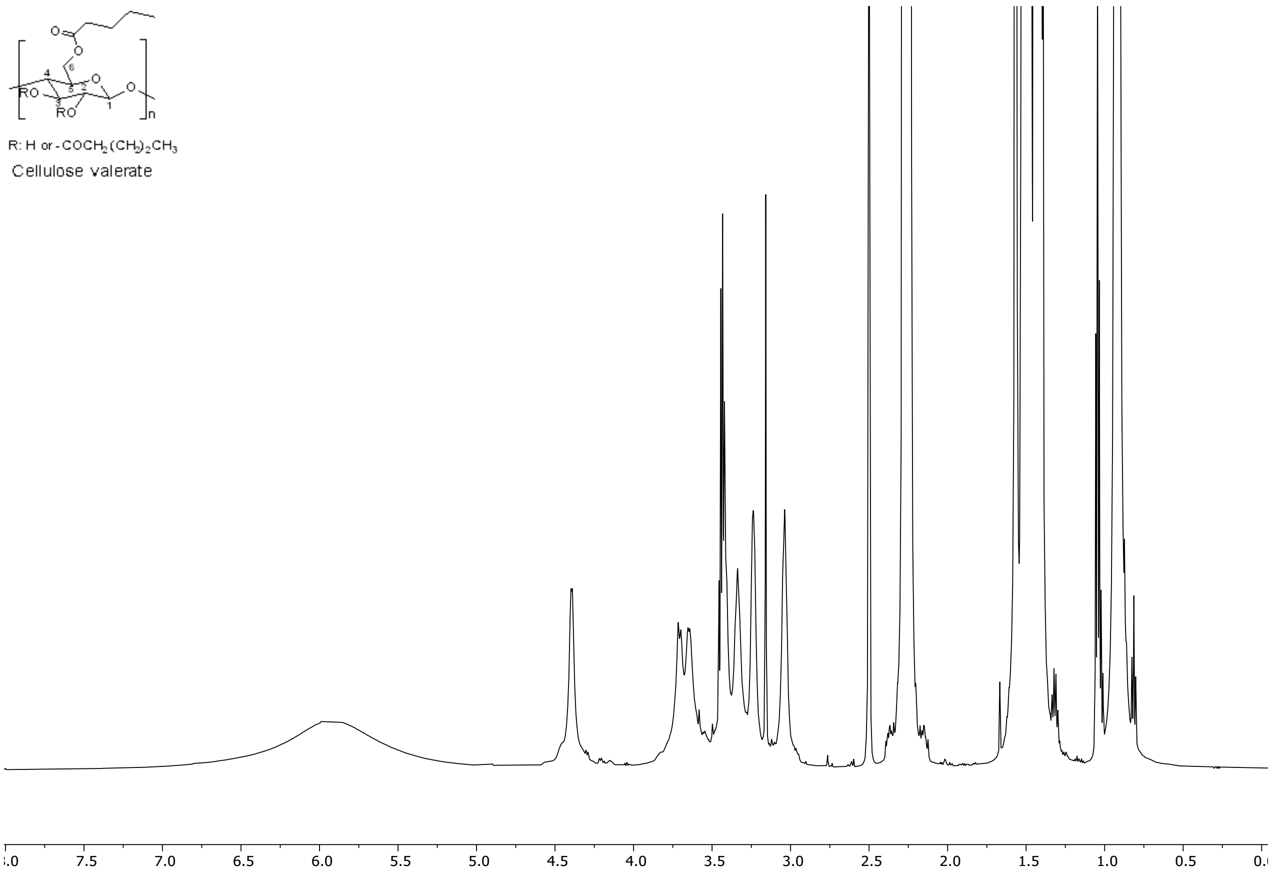

^{1}H and diffusion-edited ^{1}H spectra of **Cellulose valerate DS 0.06** in 20 w.% tetrabutylphosphonium acetate DMSO-d_6 electrolyte solution.

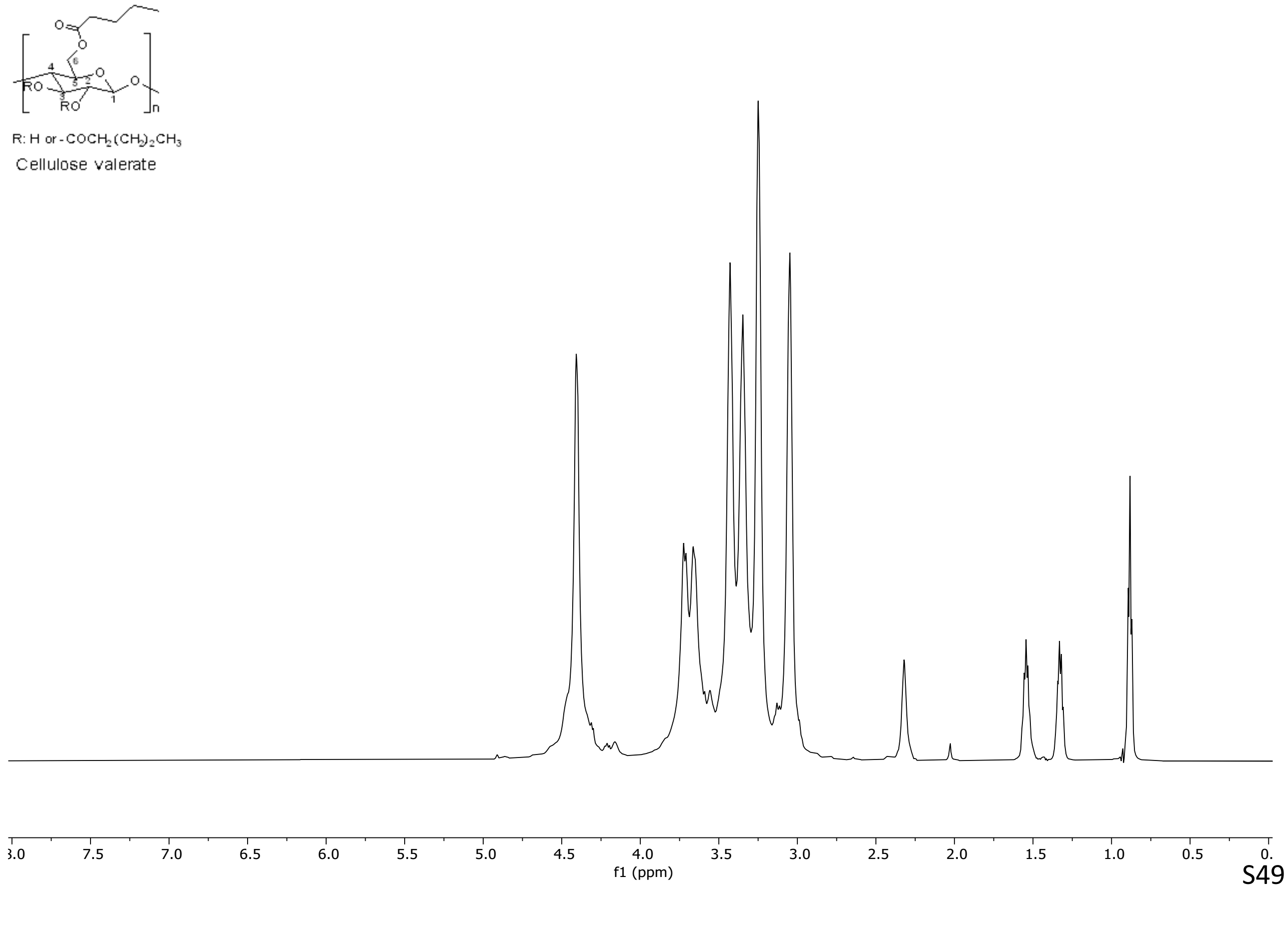

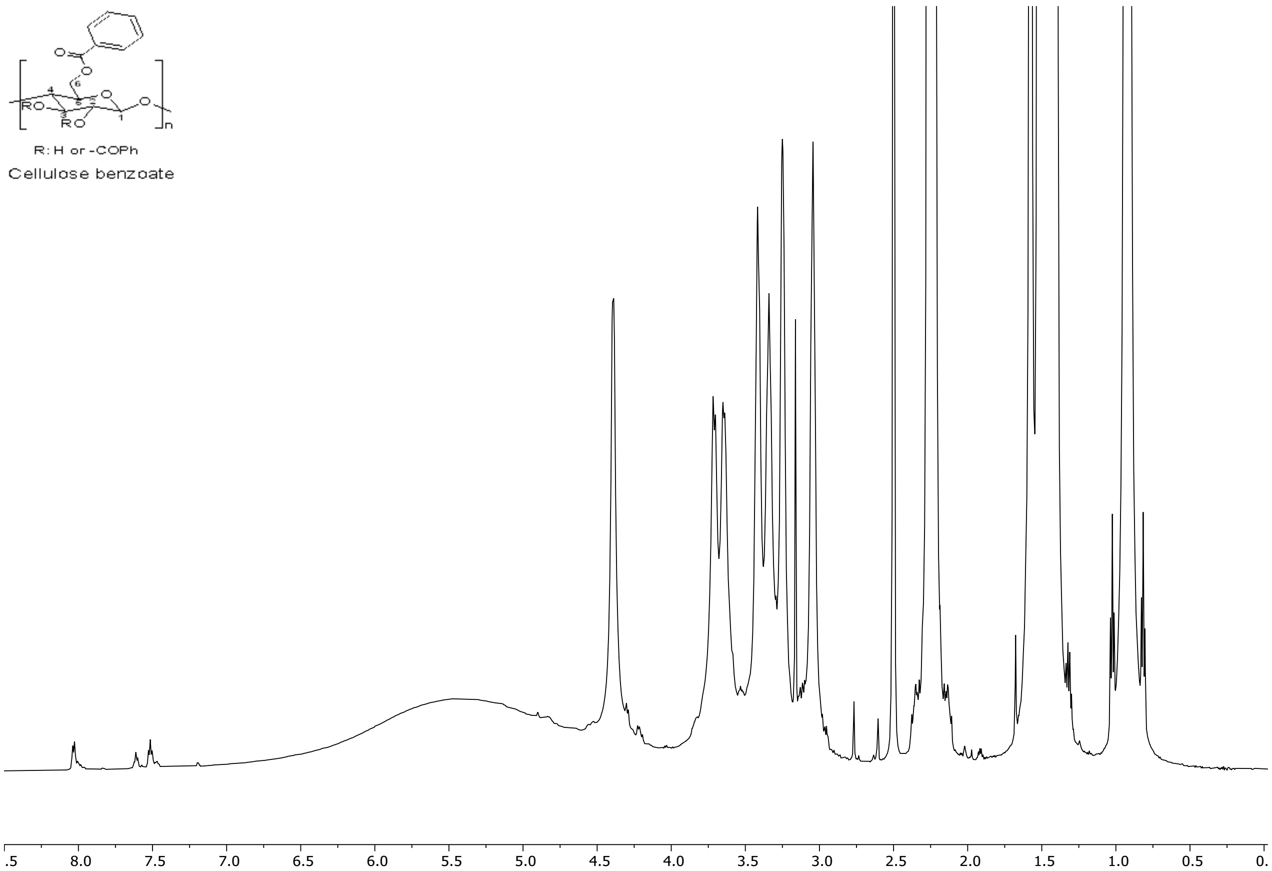

^{1}H and diffusion-edited ^{1}H spectra of **Cellulose benzoate DS 0.02** in 20 w.% tetrabutylphosphonium acetate DMSO-d_6 electrolyte solution.

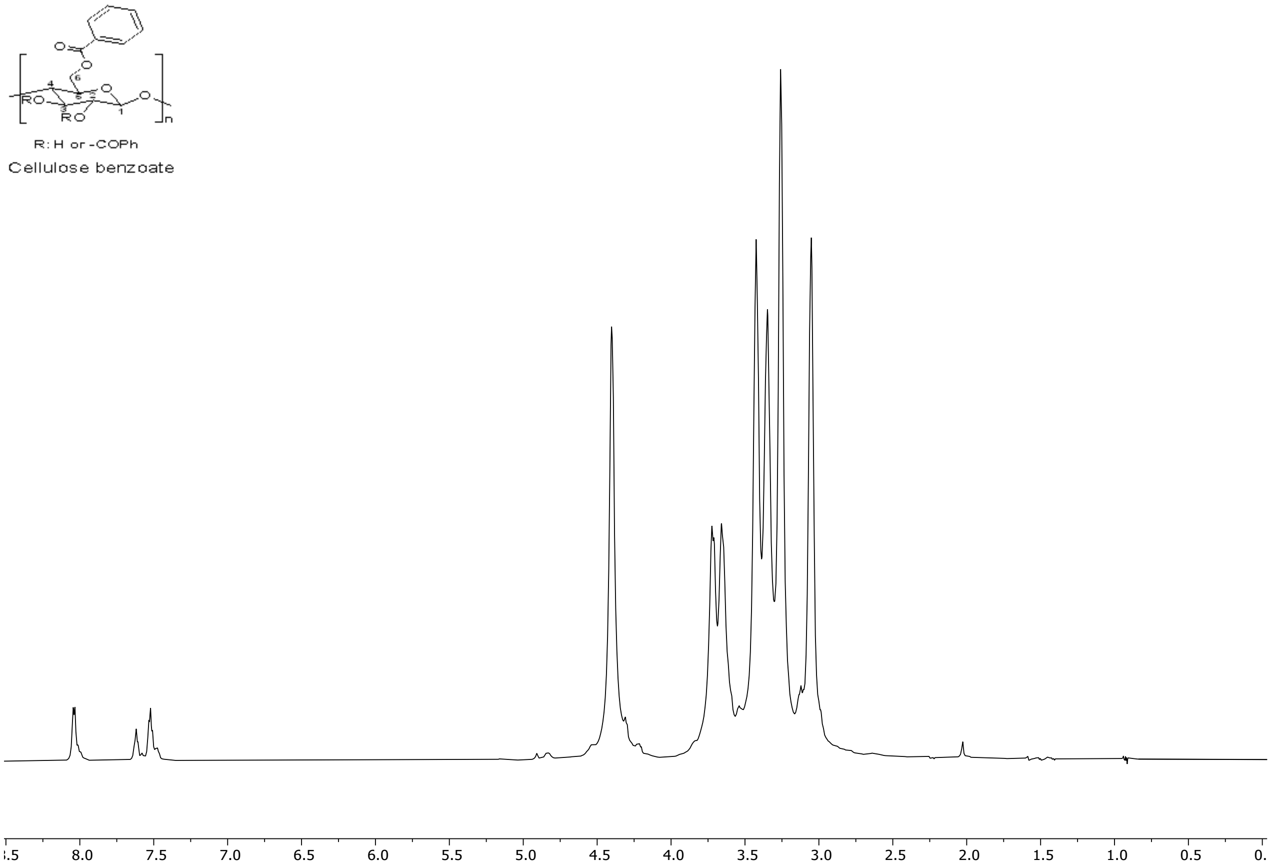

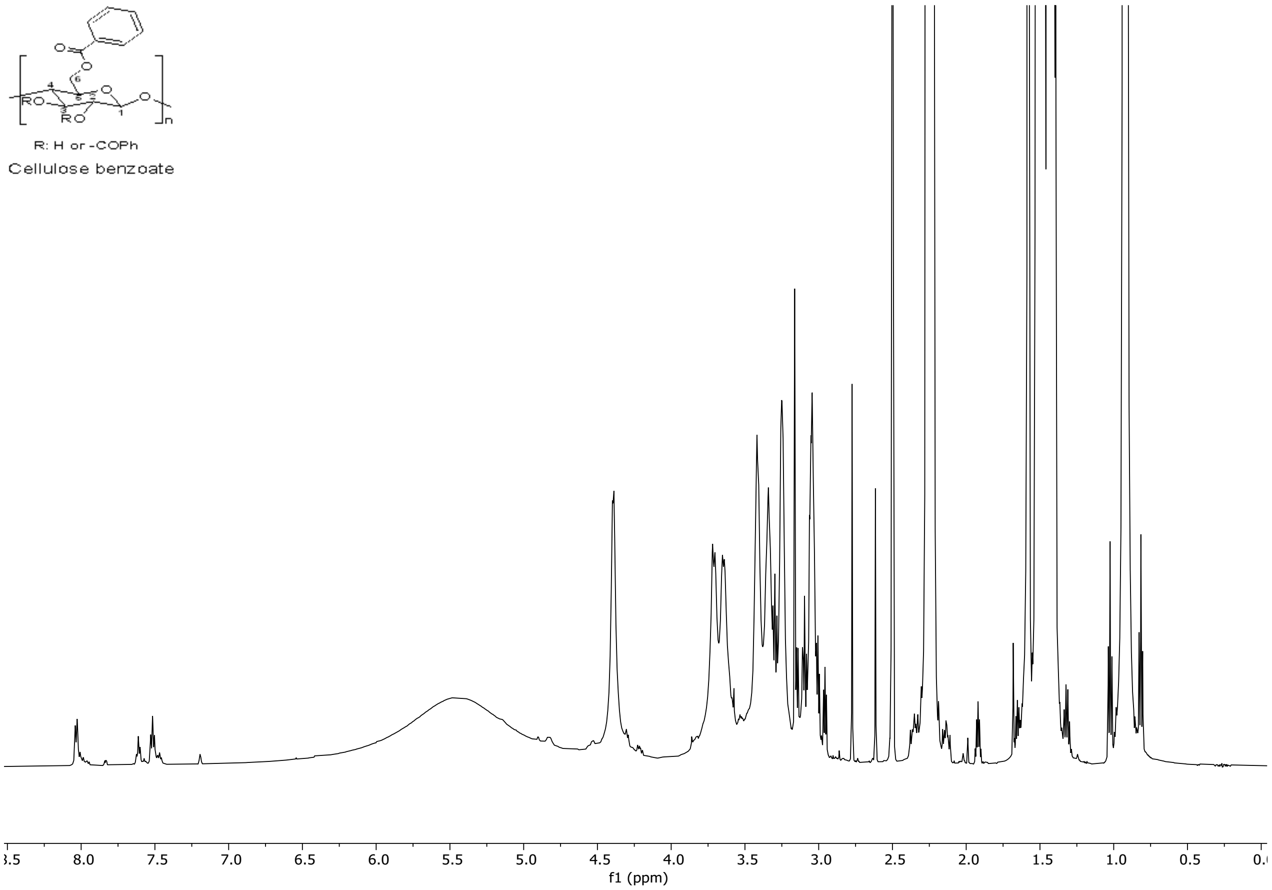

^{1}H and diffusion-edited ^{1}H spectra of **Cellulose benzoate DS 0.05** in 20 w.% tetrabutylphosphonium acetate DMSO-d_6 electrolyte solution.

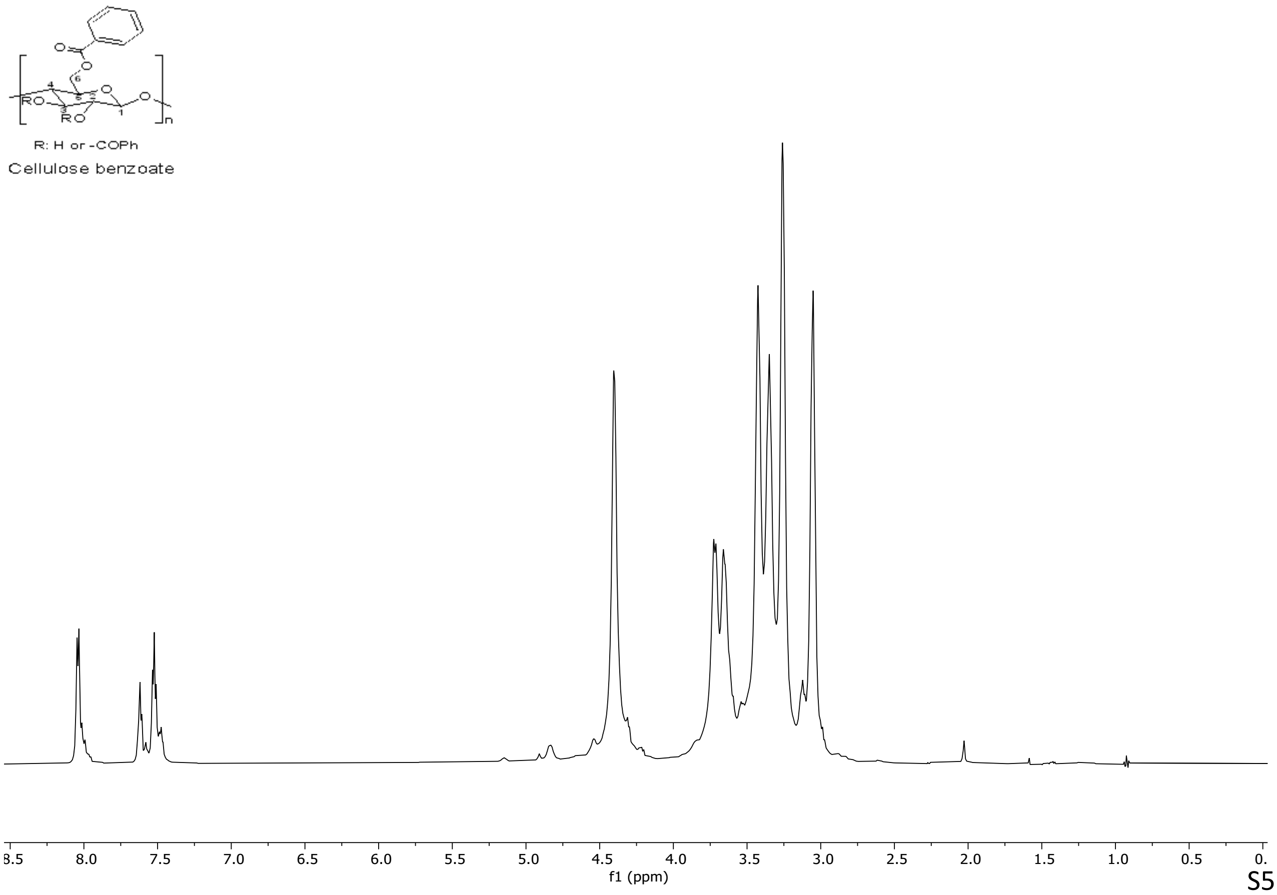

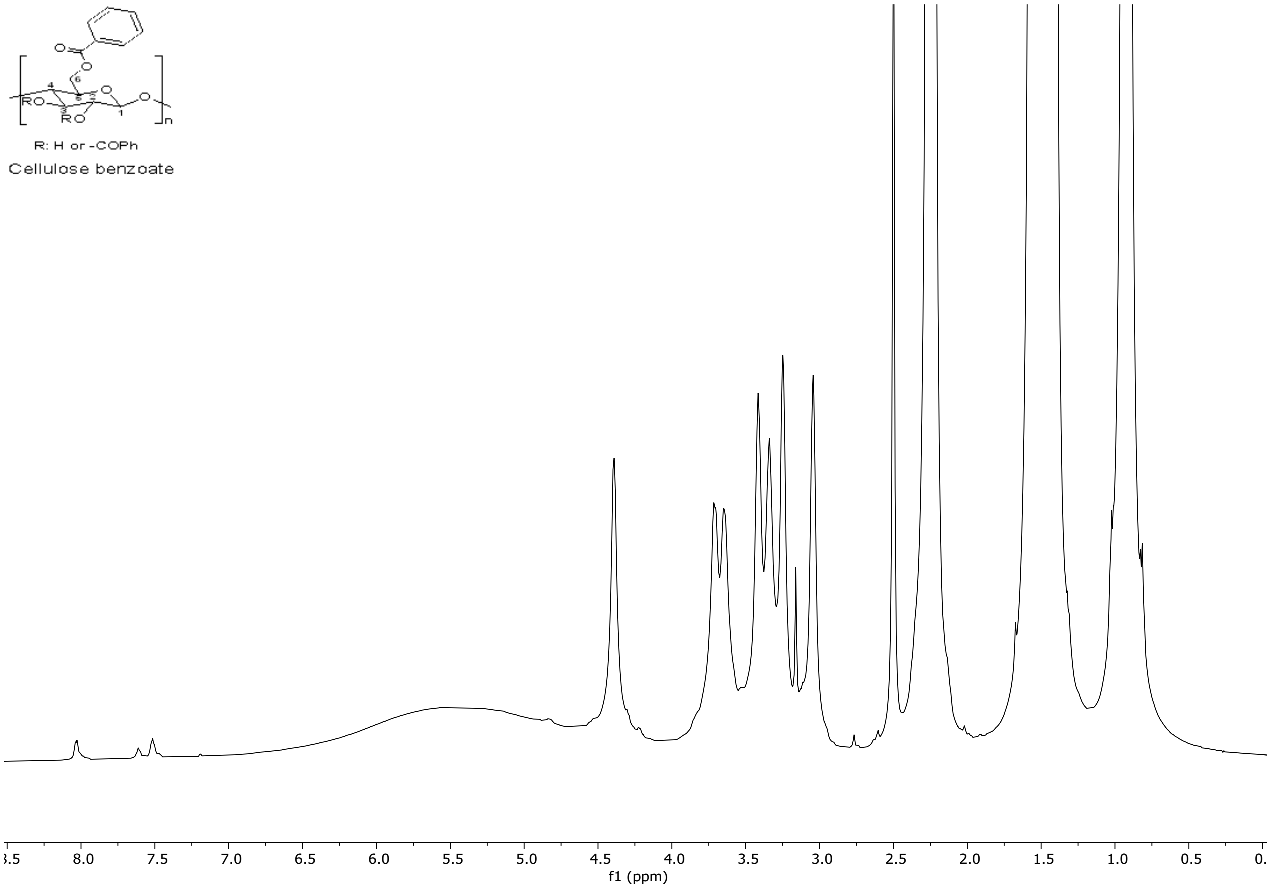

^{1}H and diffusion-edited ^{1}H spectra of **Cellulose benzoate DS 0.02** in 20 w.% tetrabutylphosphonium acetate DMSO-d_6 electrolyte solution.

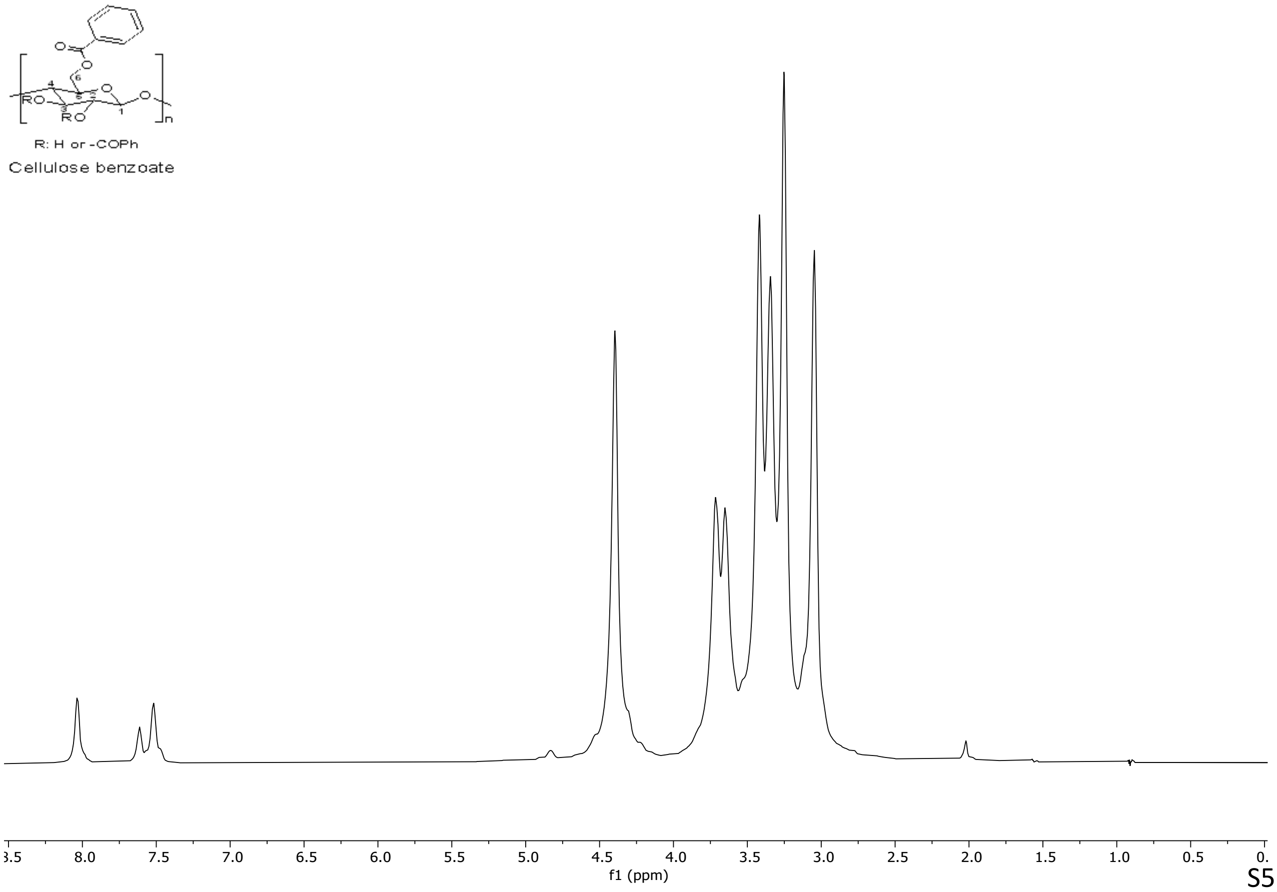

^{1}H and diffusion-edited ^{1}H spectra of **Cellulose 4-methoxybenzoate DS 0.005** in 20 w.% tetrabutylphosphonium acetate DMSO-d_6 electrolyte

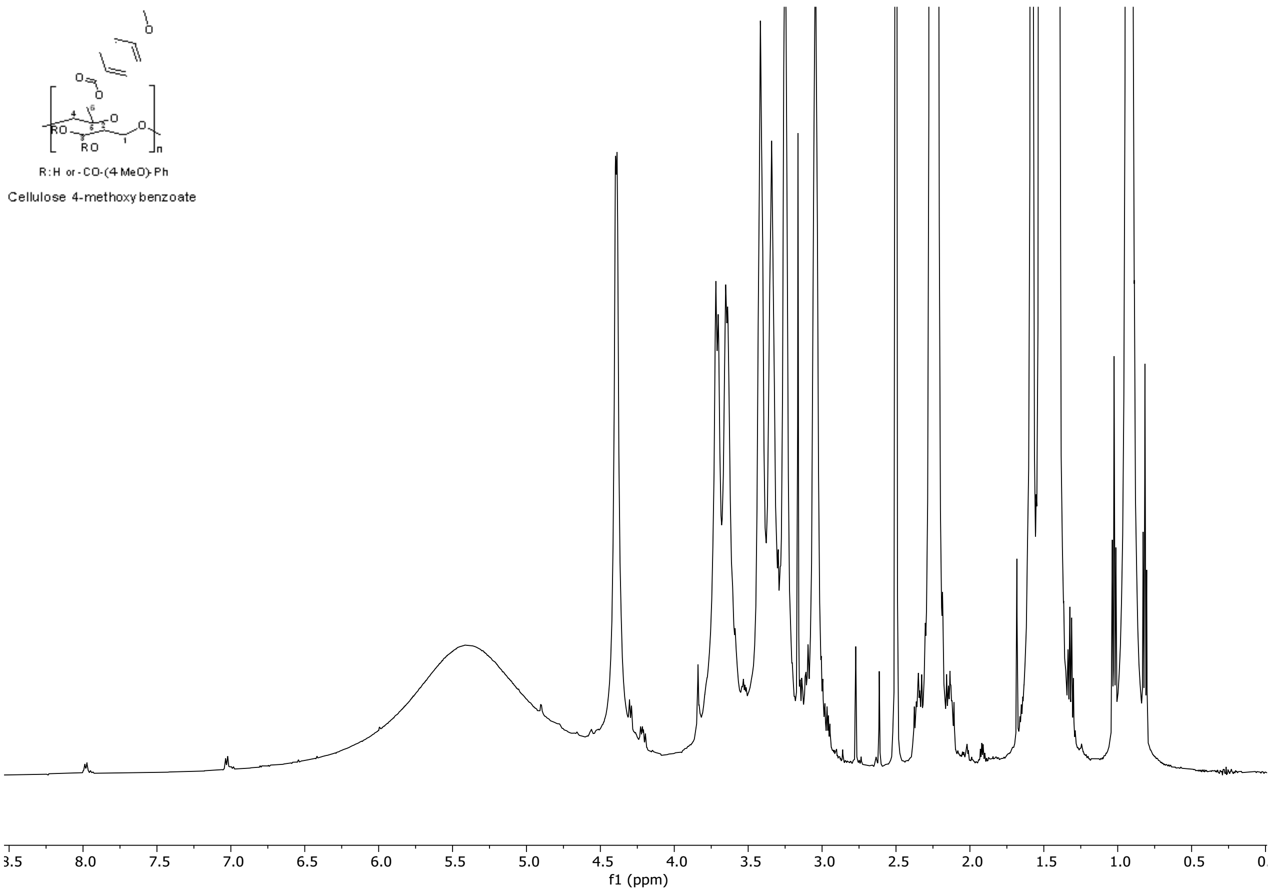

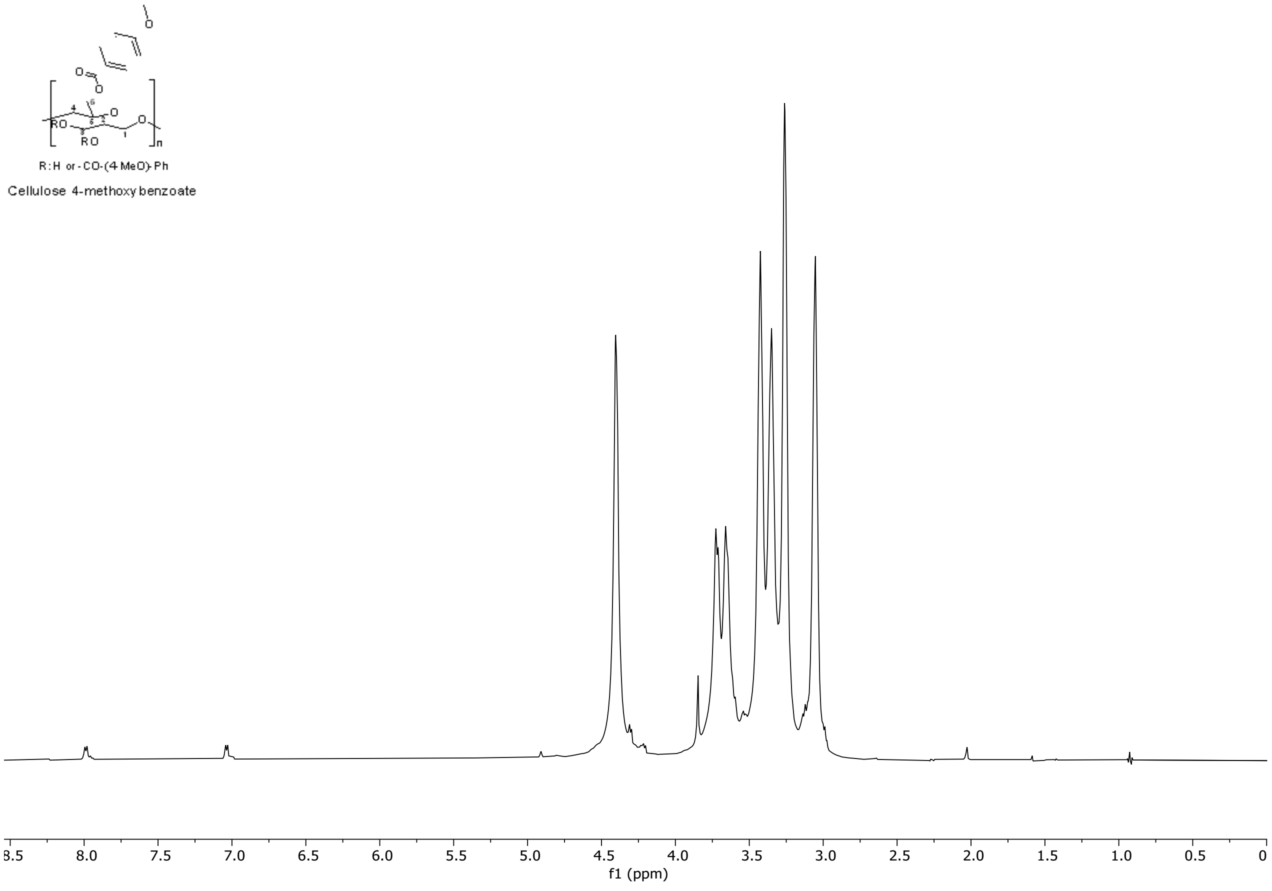

solution.

^{1}H and diffusion-edited ^{1}H spectra of **Cellulose 4-methoxybenzoate DS 0.01** in 20 w.% tetrabutylphosphonium acetate DMSO-d_6 electrolyte

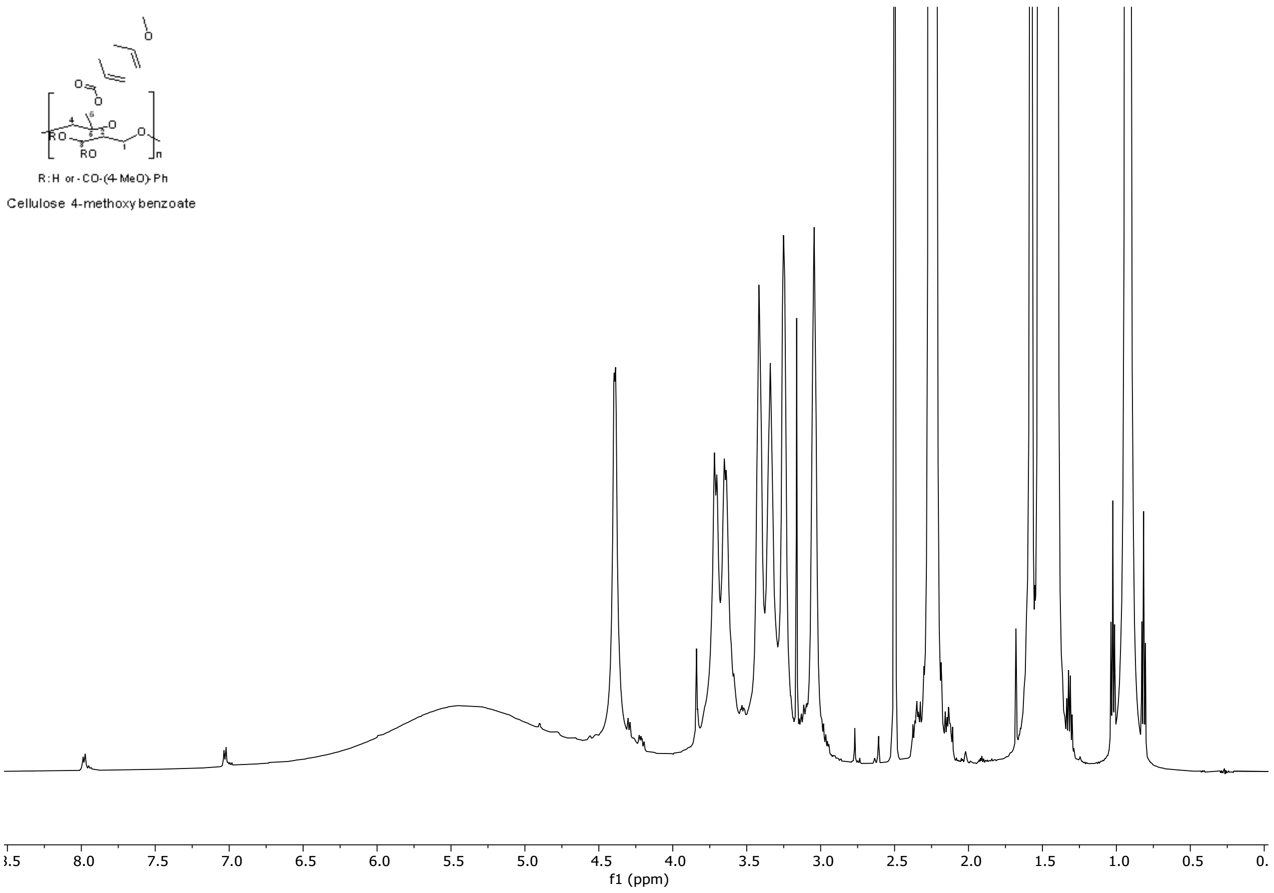

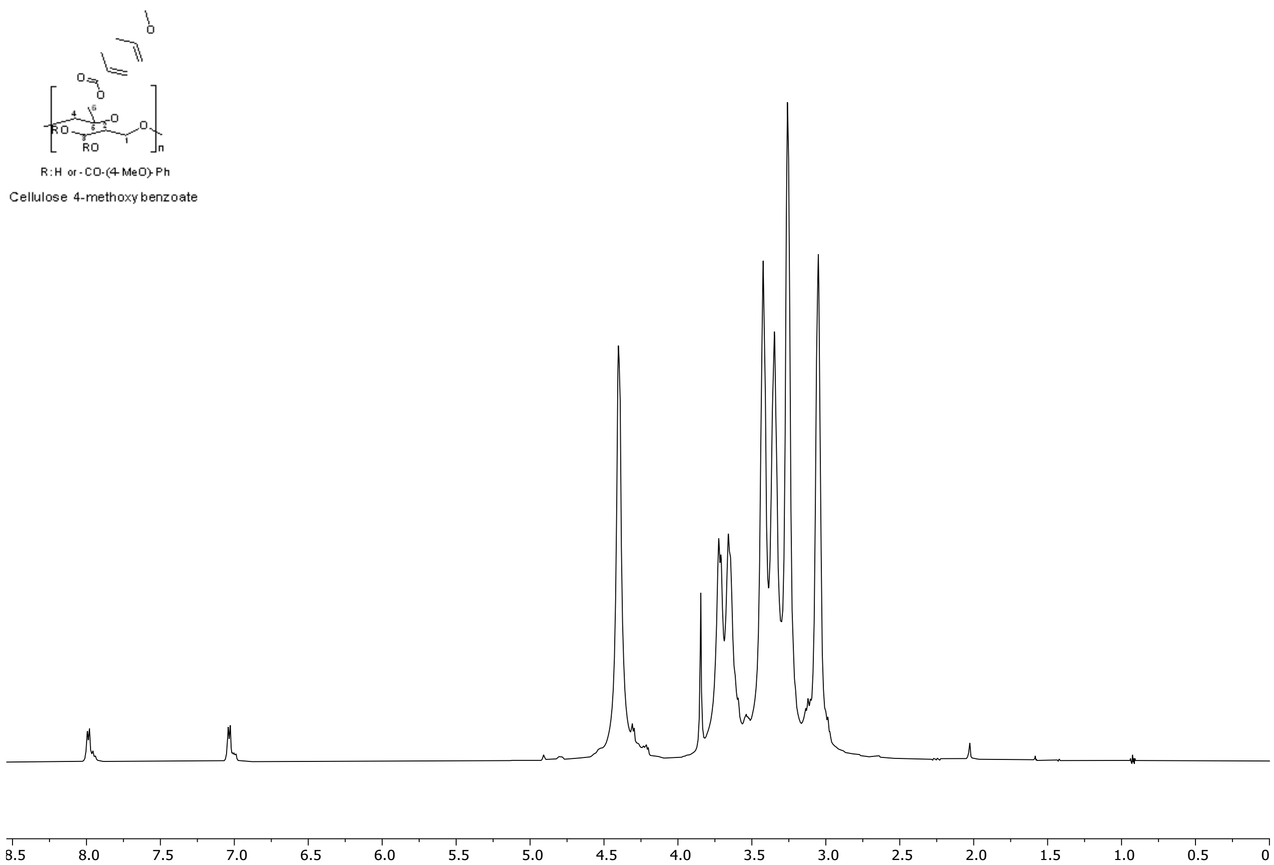

solution.

^{1}H and diffusion-edited ^{1}H spectra of **Cellulose 4-methoxybenzoate DS 0.01** in 20 w.% tetrabutylphosphonium acetate DMSO-d_6 electrolyte

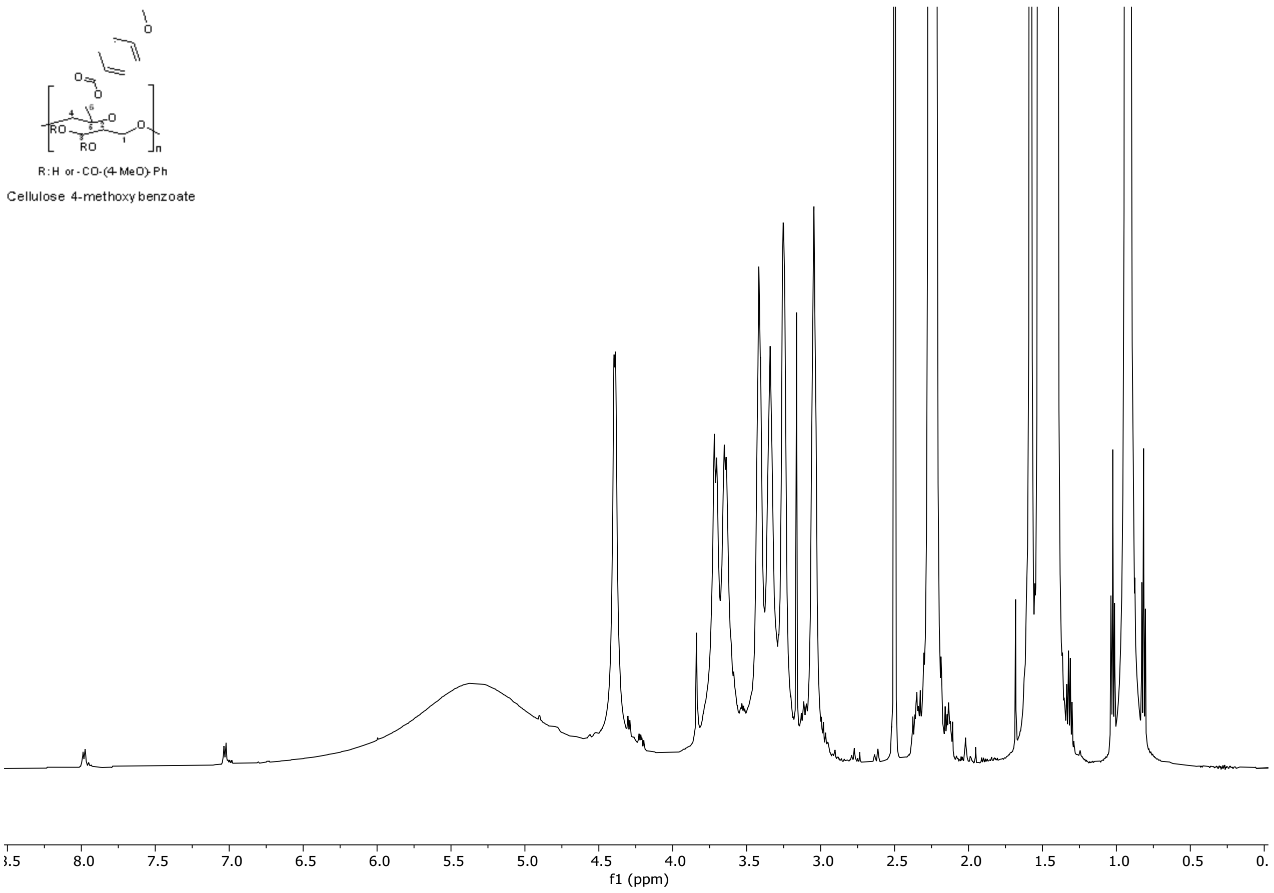

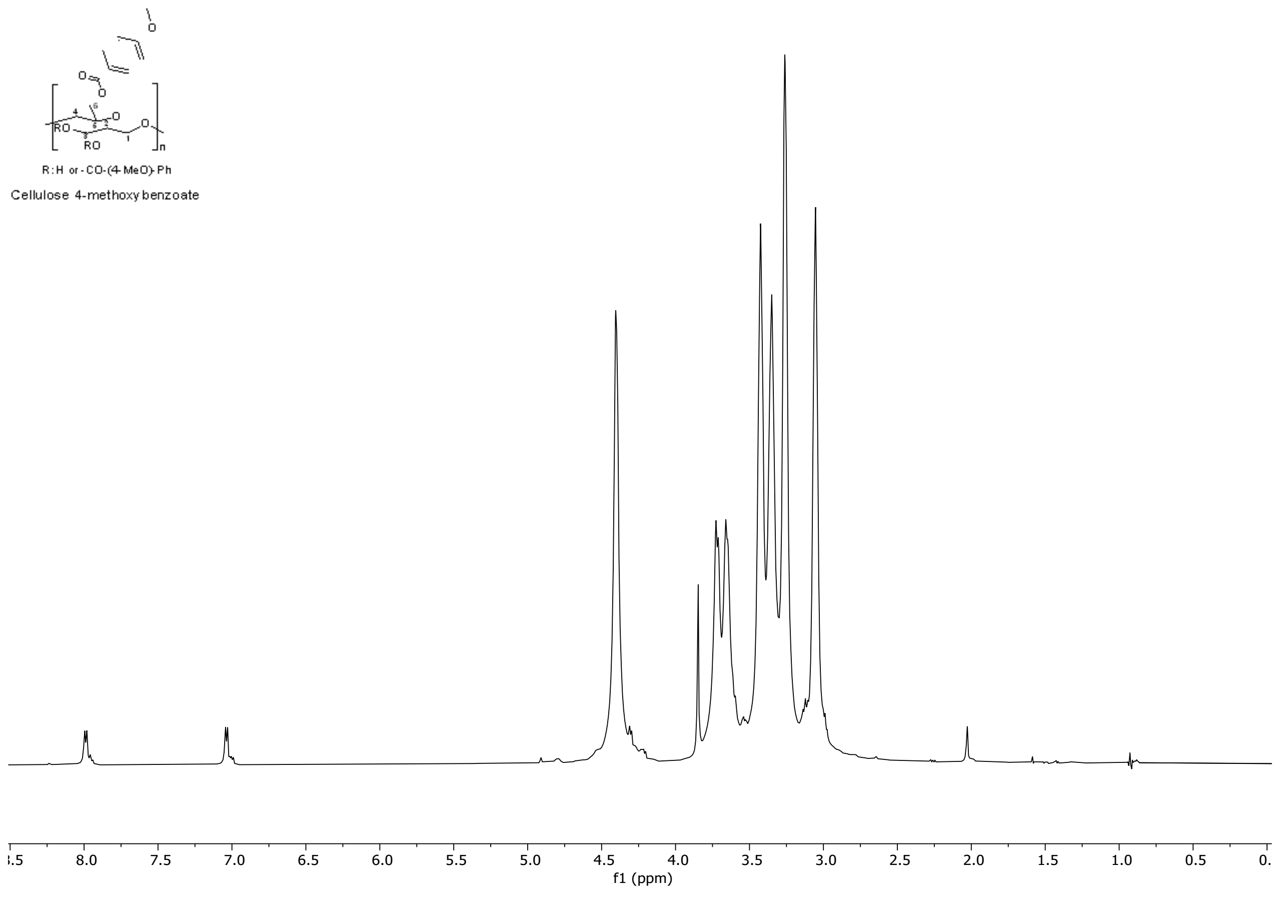

solution.

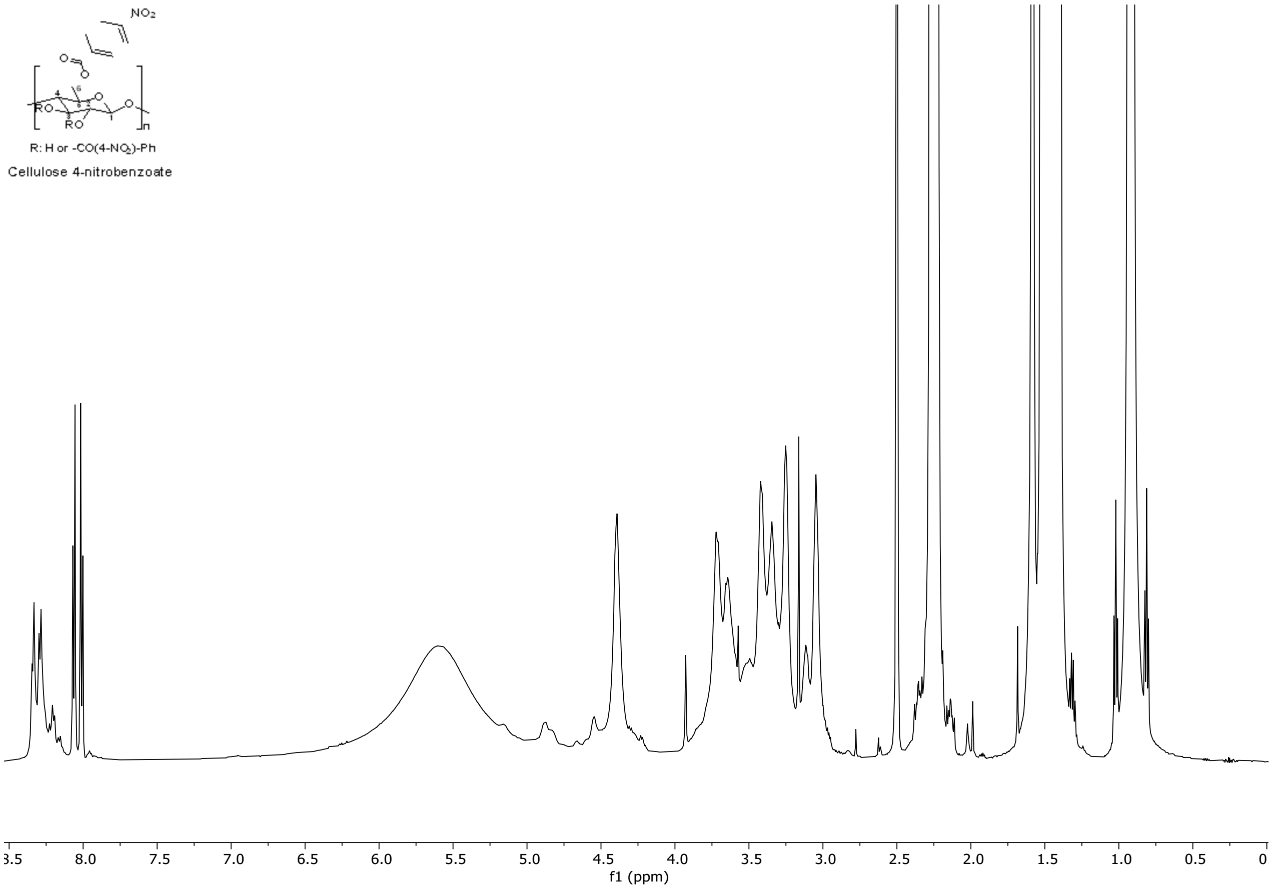

^{1}H and diffusion-edited ^{1}H spectra of **Cellulose 4-nitrobenzoate DS 0.25** in 20 w.% tetrabutylphosphonium acetate DMSO-d_6 electrolyte solution.

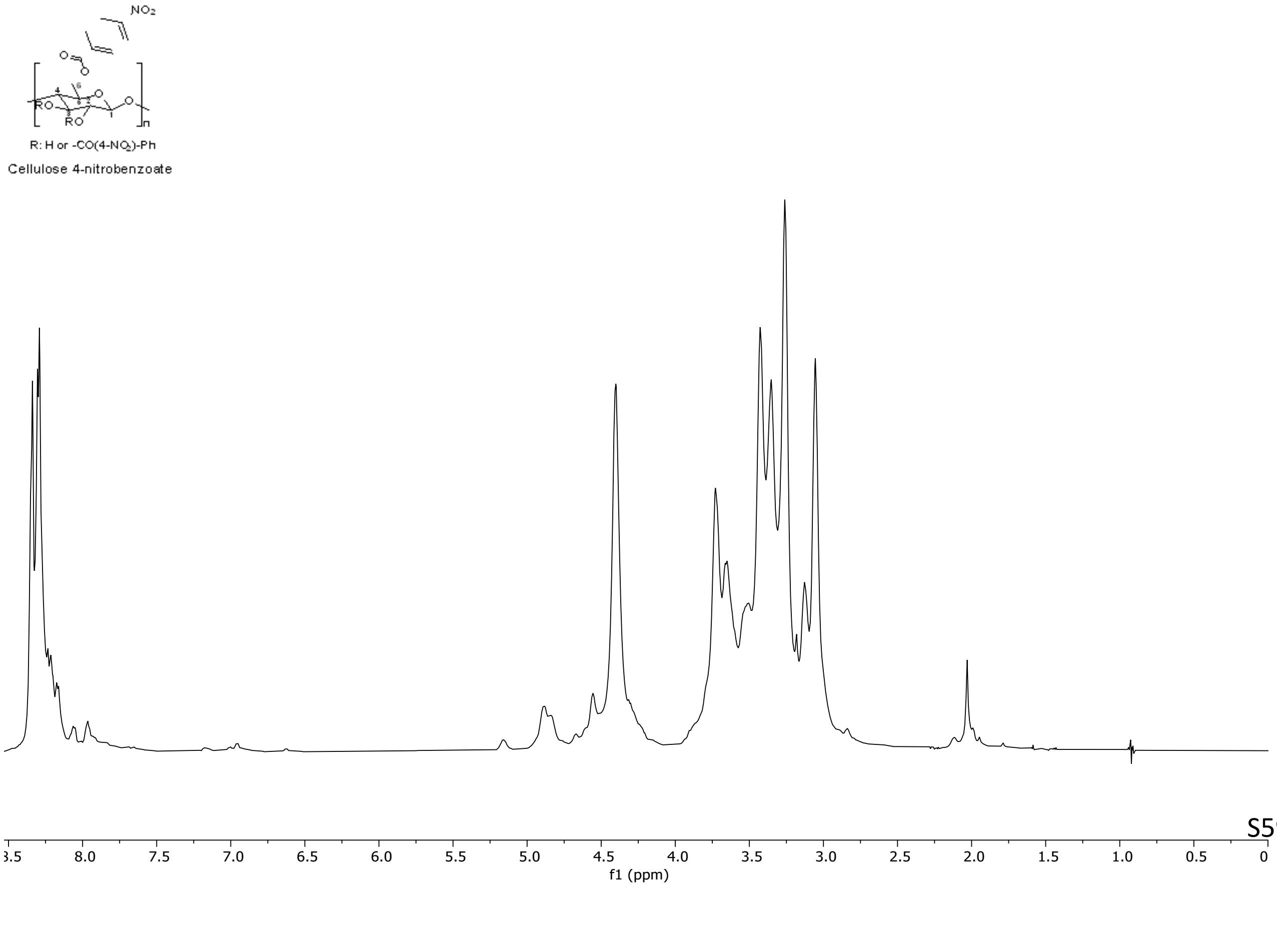

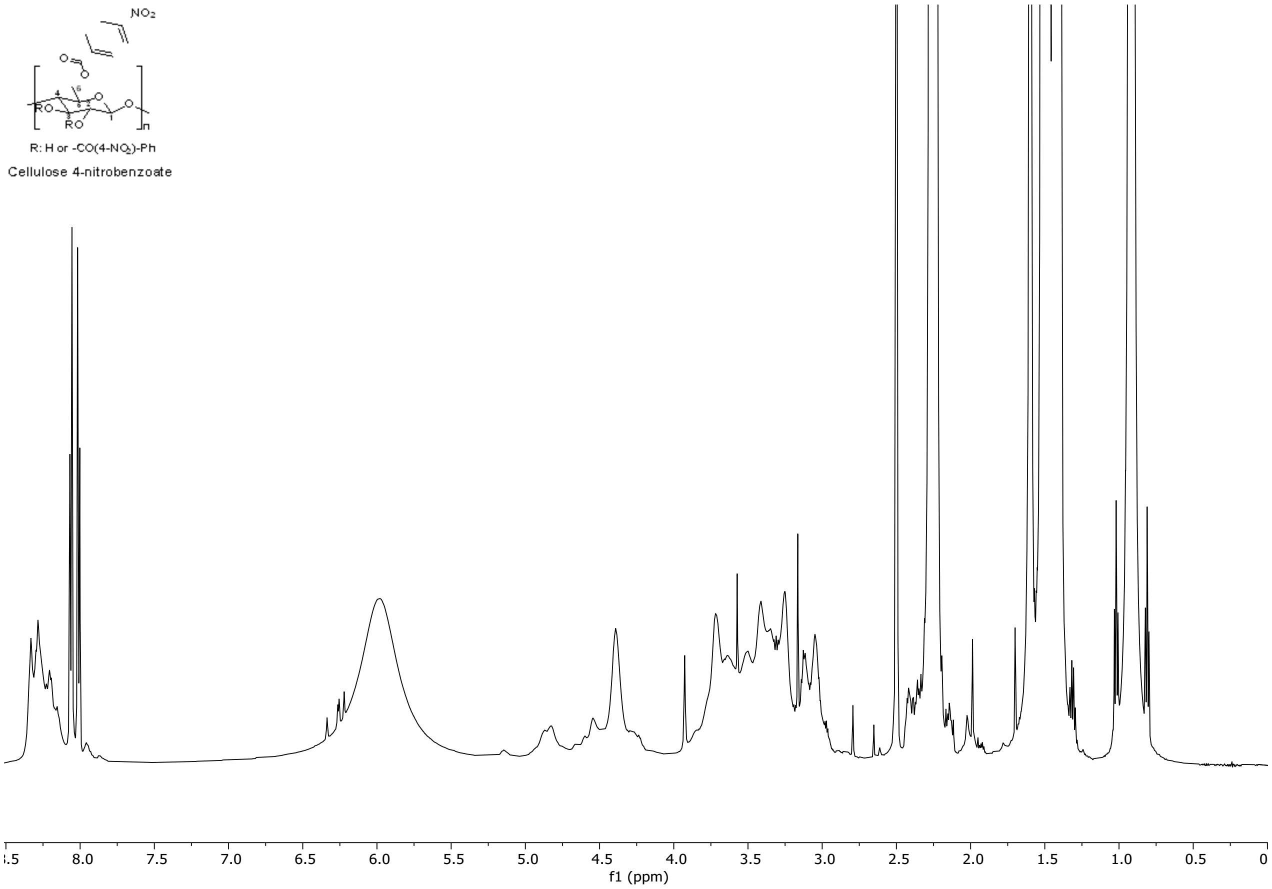

^{1}H and diffusion-edited ^{1}H spectra of **Cellulose 4-nitrobenzoate DS 0.75** in 20 w.% tetrabutylphosphonium acetate DMSO-d_6 electrolyte solution.

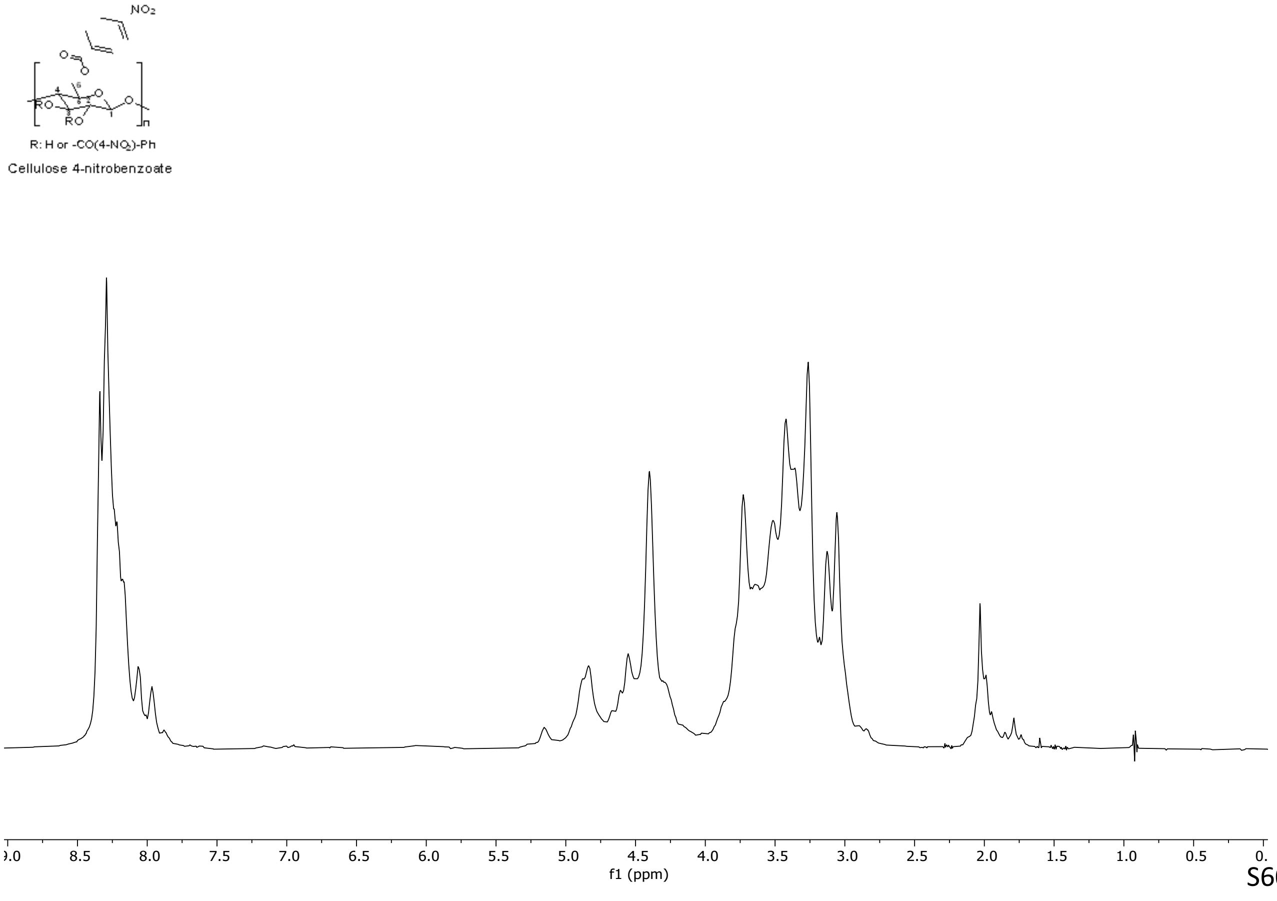

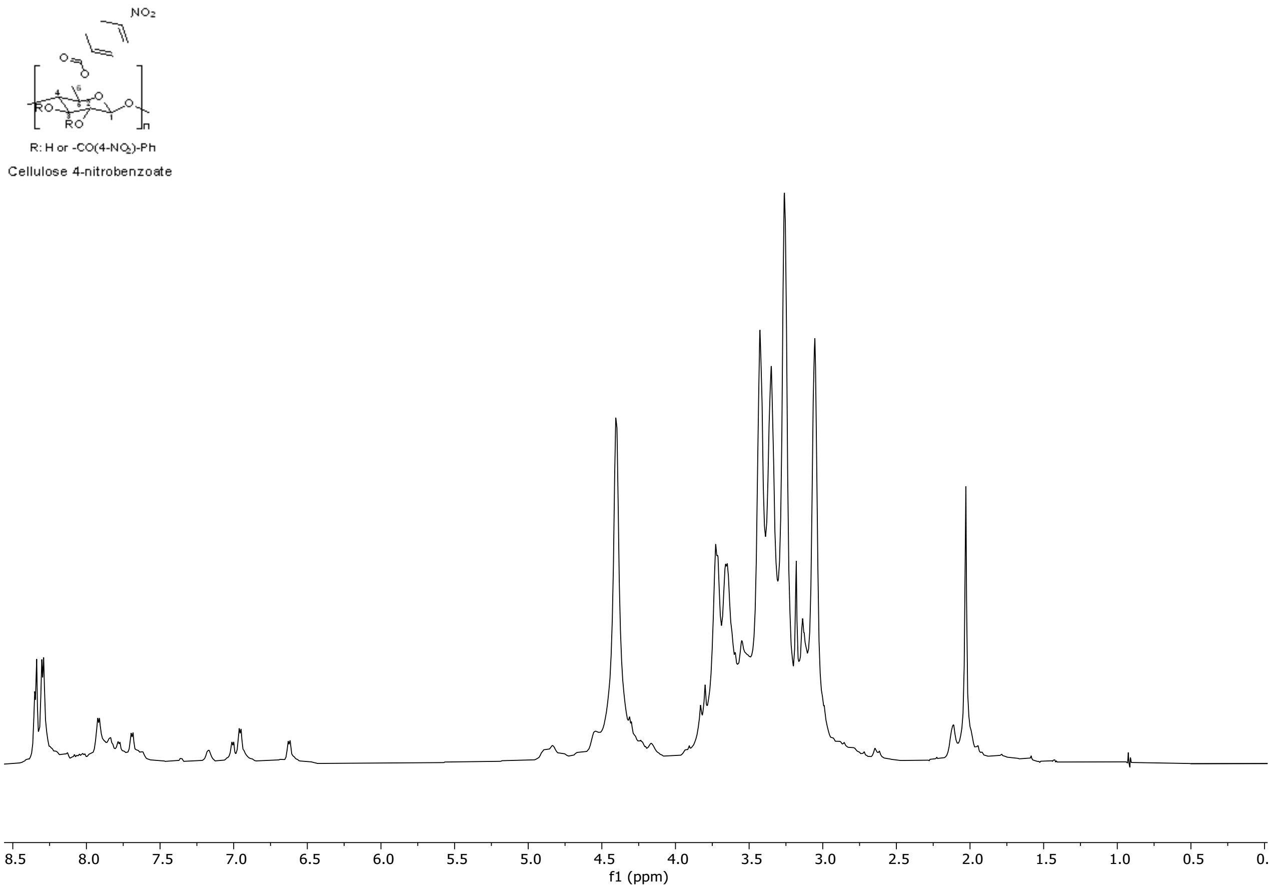

^{1}H and diffusion-edited ^{1}H spectra of **Cellulose 4-nitrobenzoate DS 0.03** in 20 w.% tetrabutylphosphonium acetate DMSO-d_6 electrolyte solution.

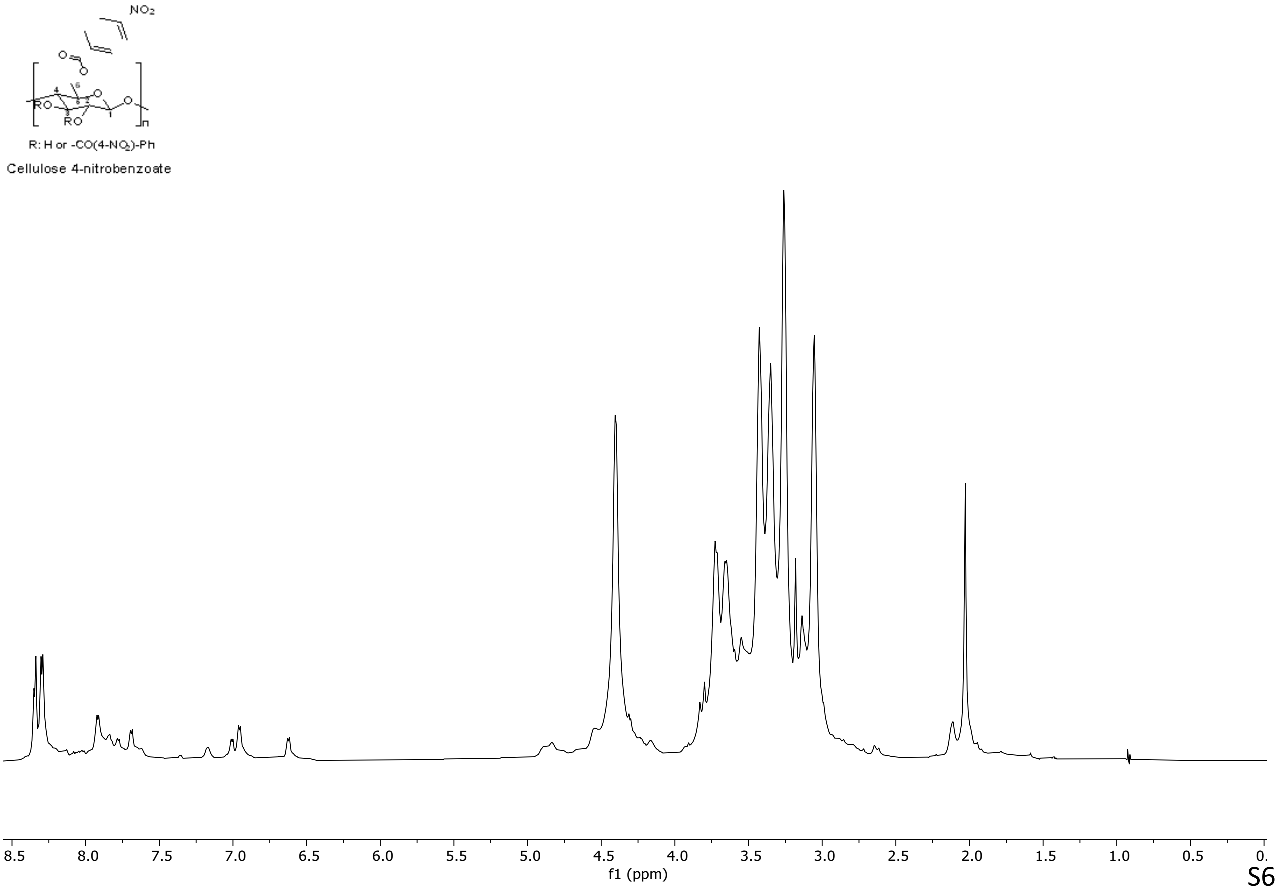

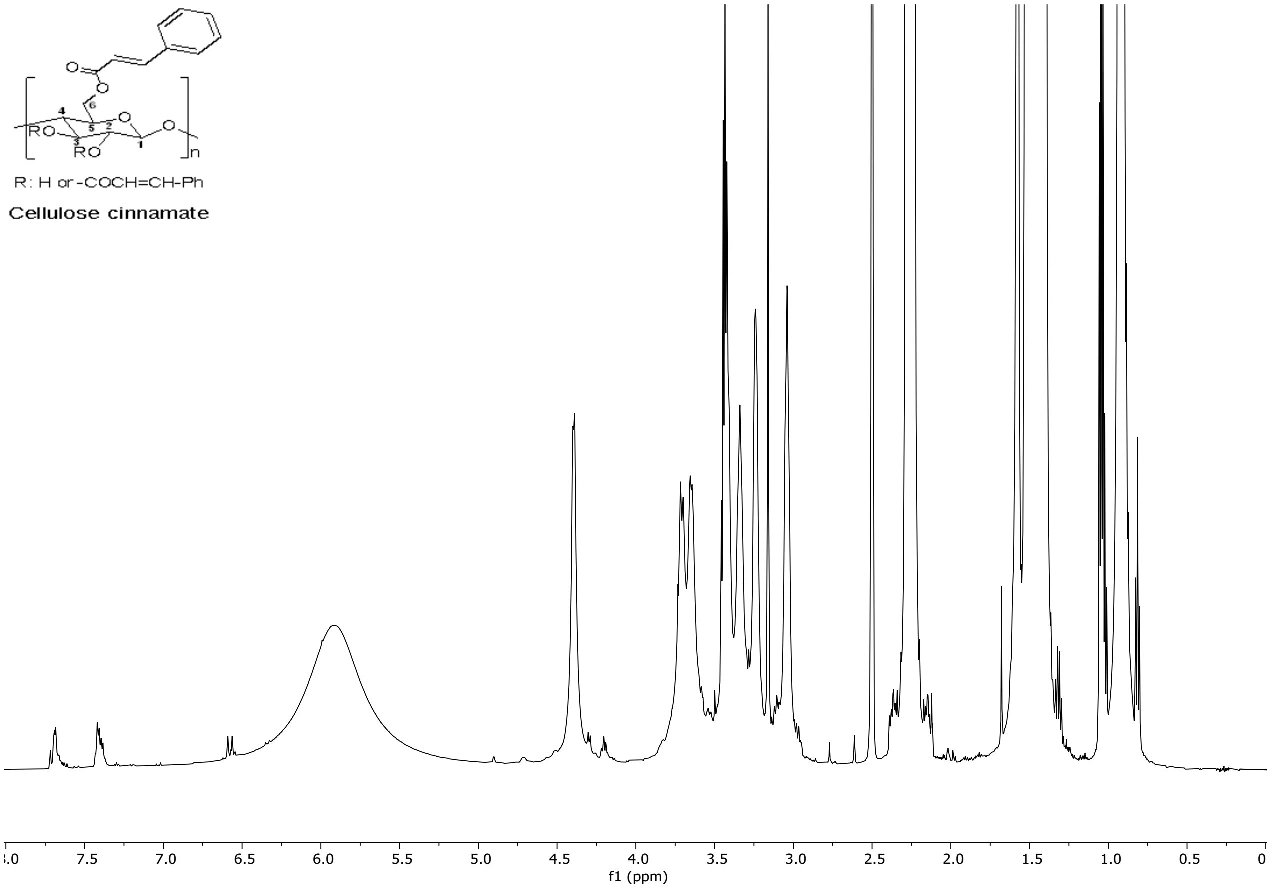

^{1}H and diffusion-edited ^{1}H spectra of **Cellulose cinnamate DS 0.03** in 20 w.% tetrabutylphosphonium acetate DMSO-d_6 electrolyte solution.

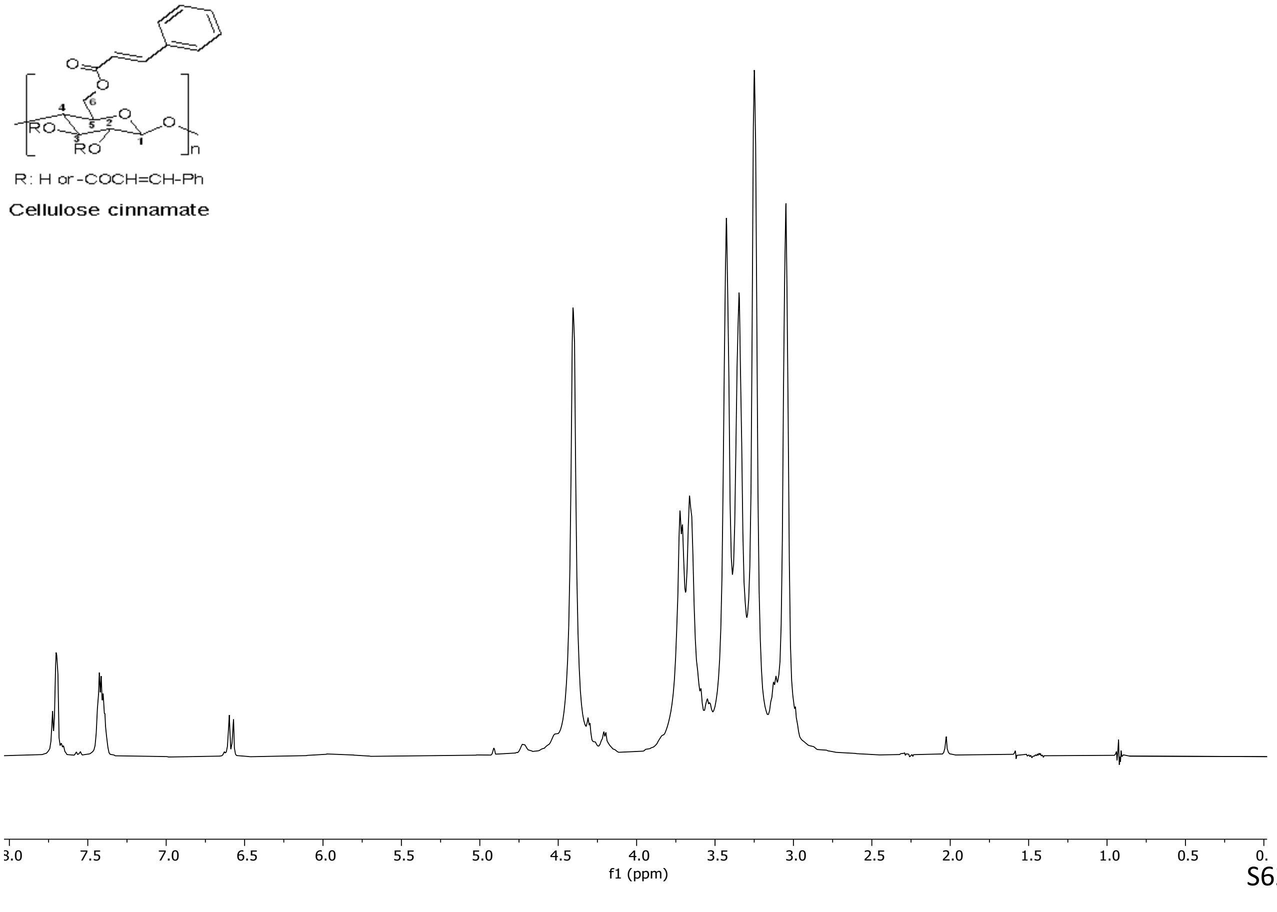

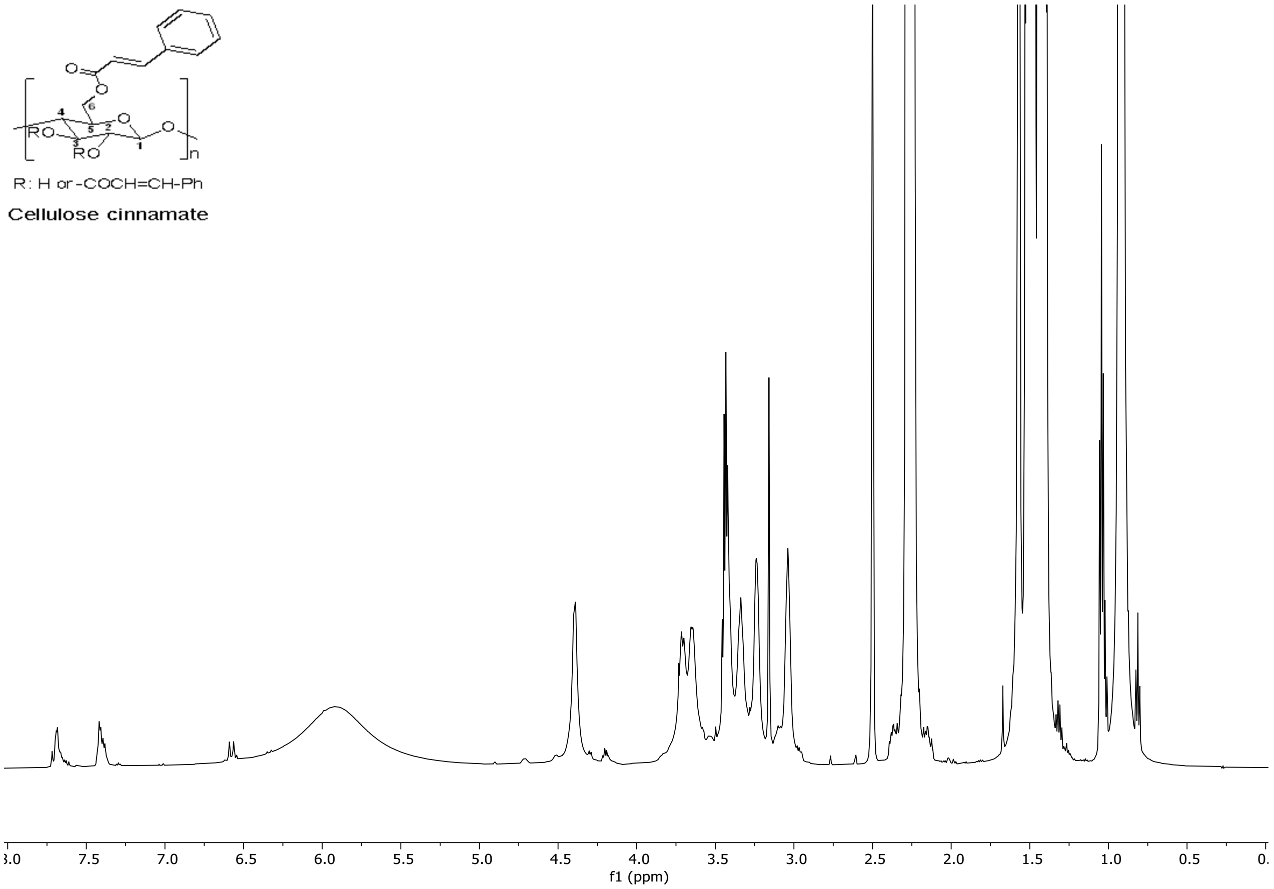

^{1}H and diffusion-edited ^{1}H spectra of **Cellulose cinnamate DS 0.07** in 20 w.% tetrabutylphosphonium acetate DMSO-d_6 electrolyte solution.

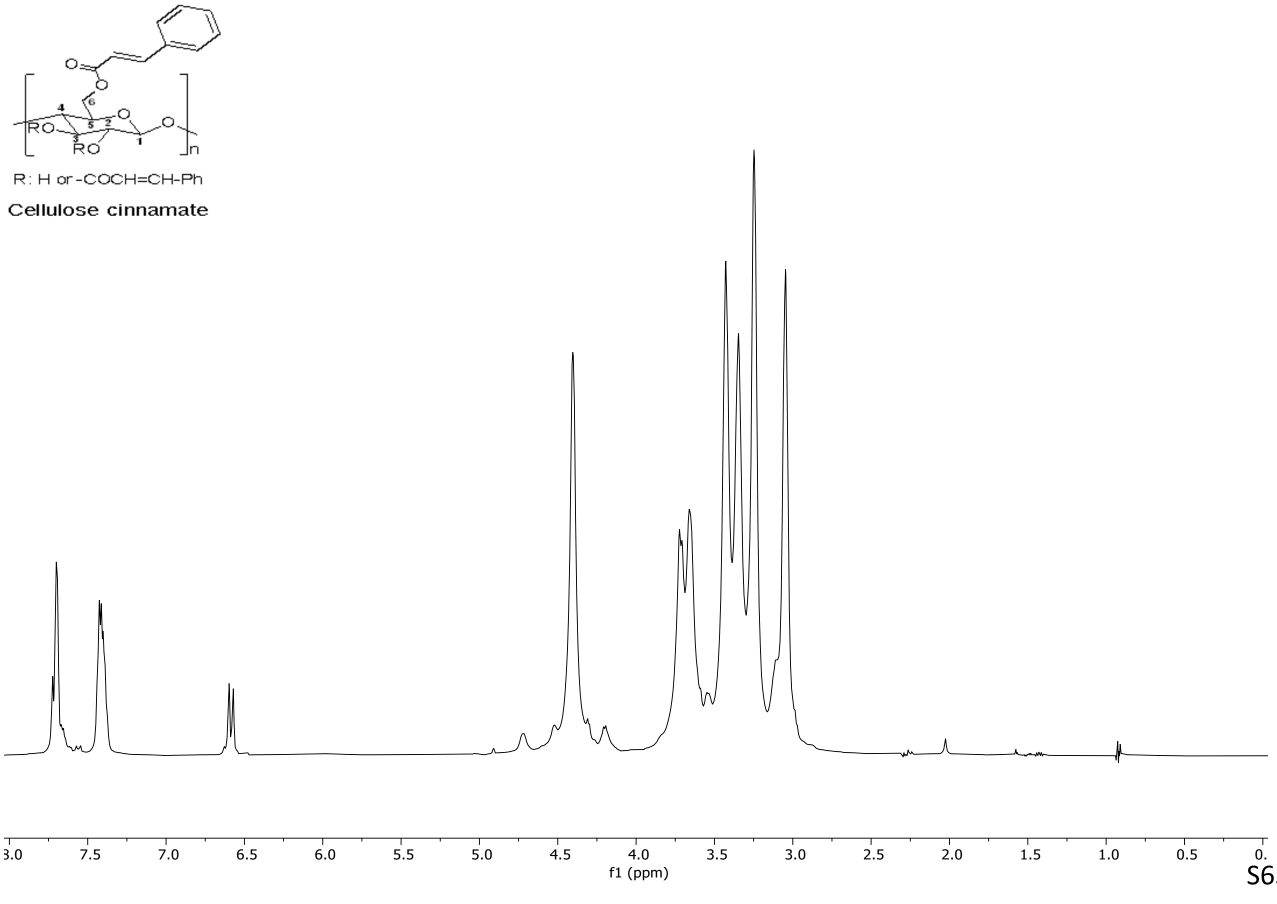

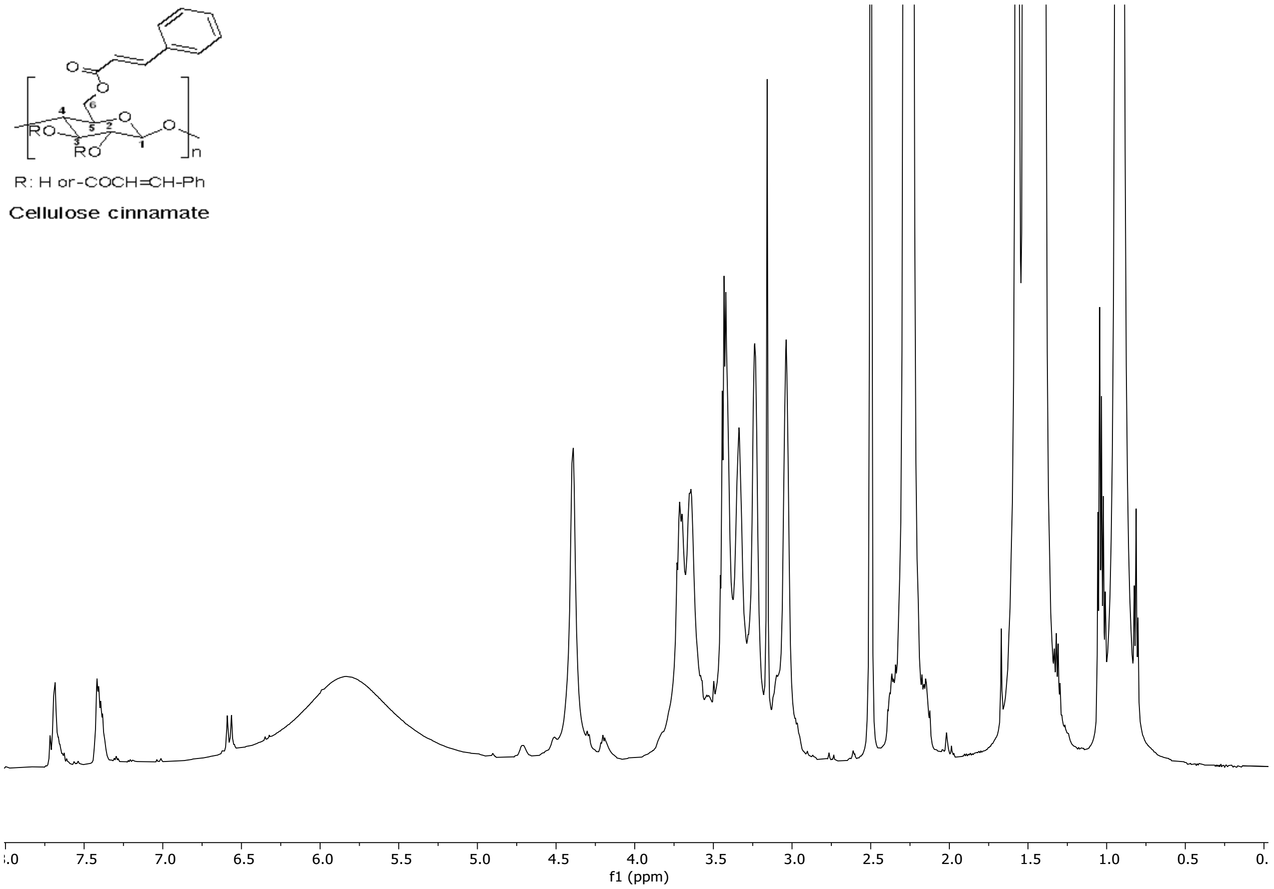

^{1}H and diffusion-edited ^{1}H spectra of **Cellulose cinnamate DS 0.07** in 20 w.% tetrabutylphosphonium acetate DMSO-d_6 electrolyte solution.

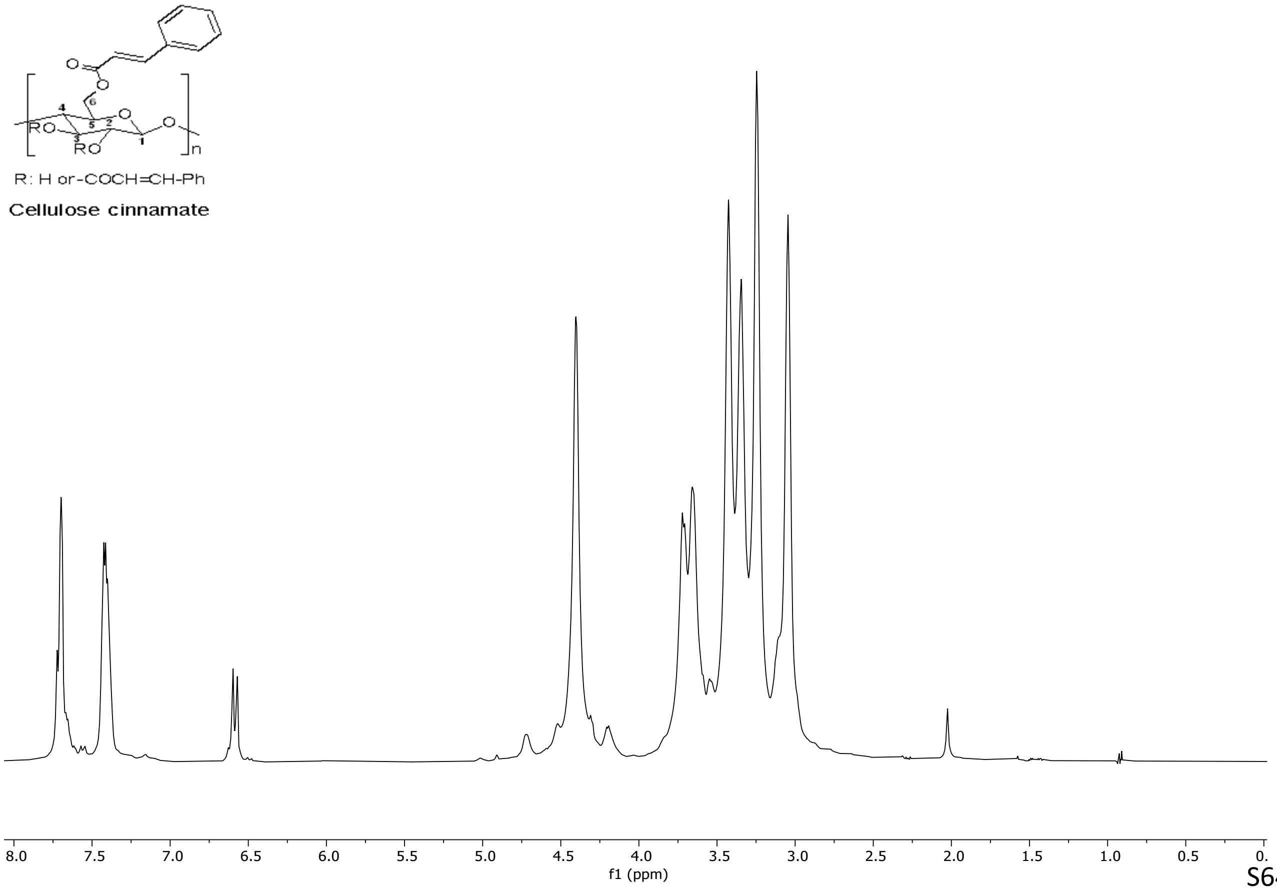

^{1}H, diffusion-edited ^{1}H, and q^{13}C{^{1}H} spectra of **Cellulose glycolate DS 0.05, DG 1.57** in 20 w.% tetrabutylphosphonium acetate DMSO-d_6 electrolyte

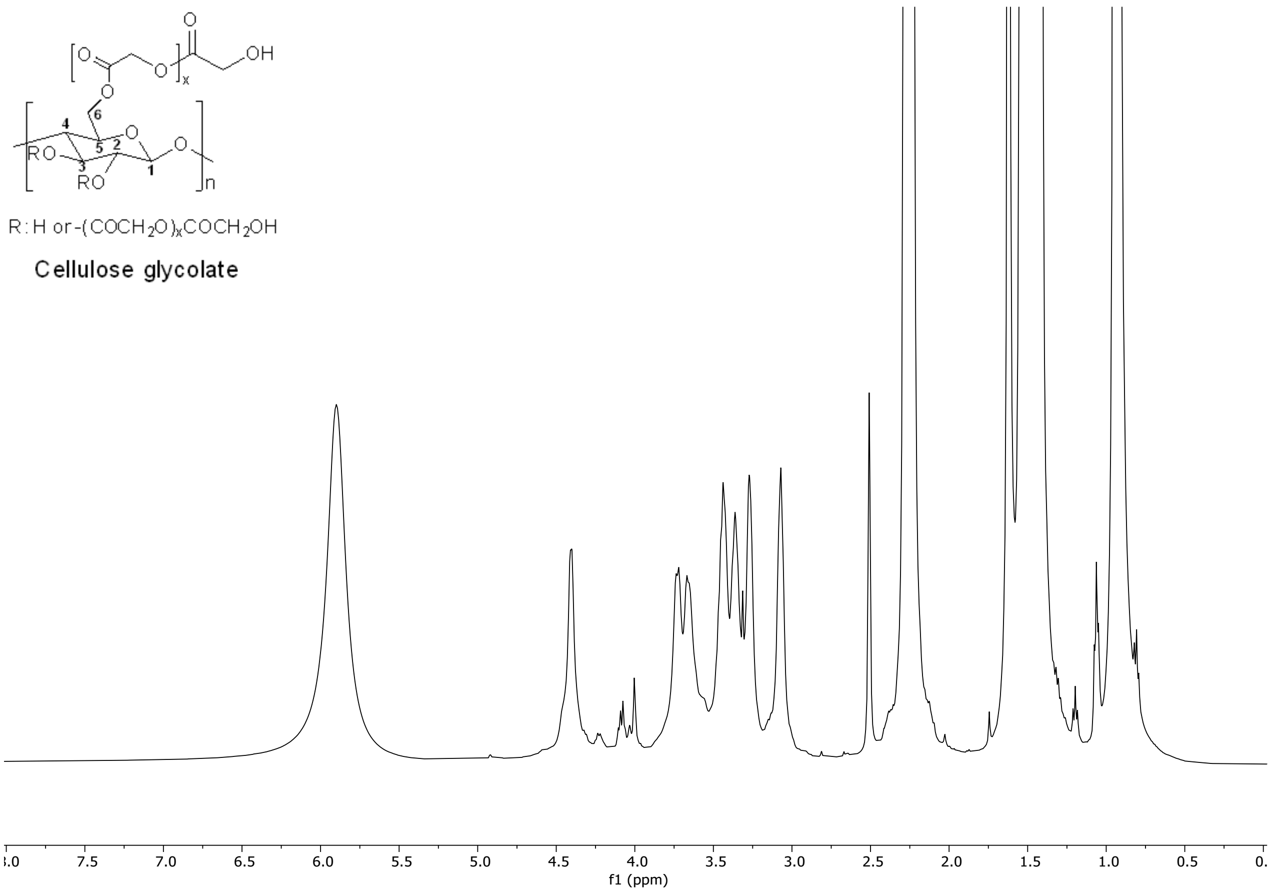

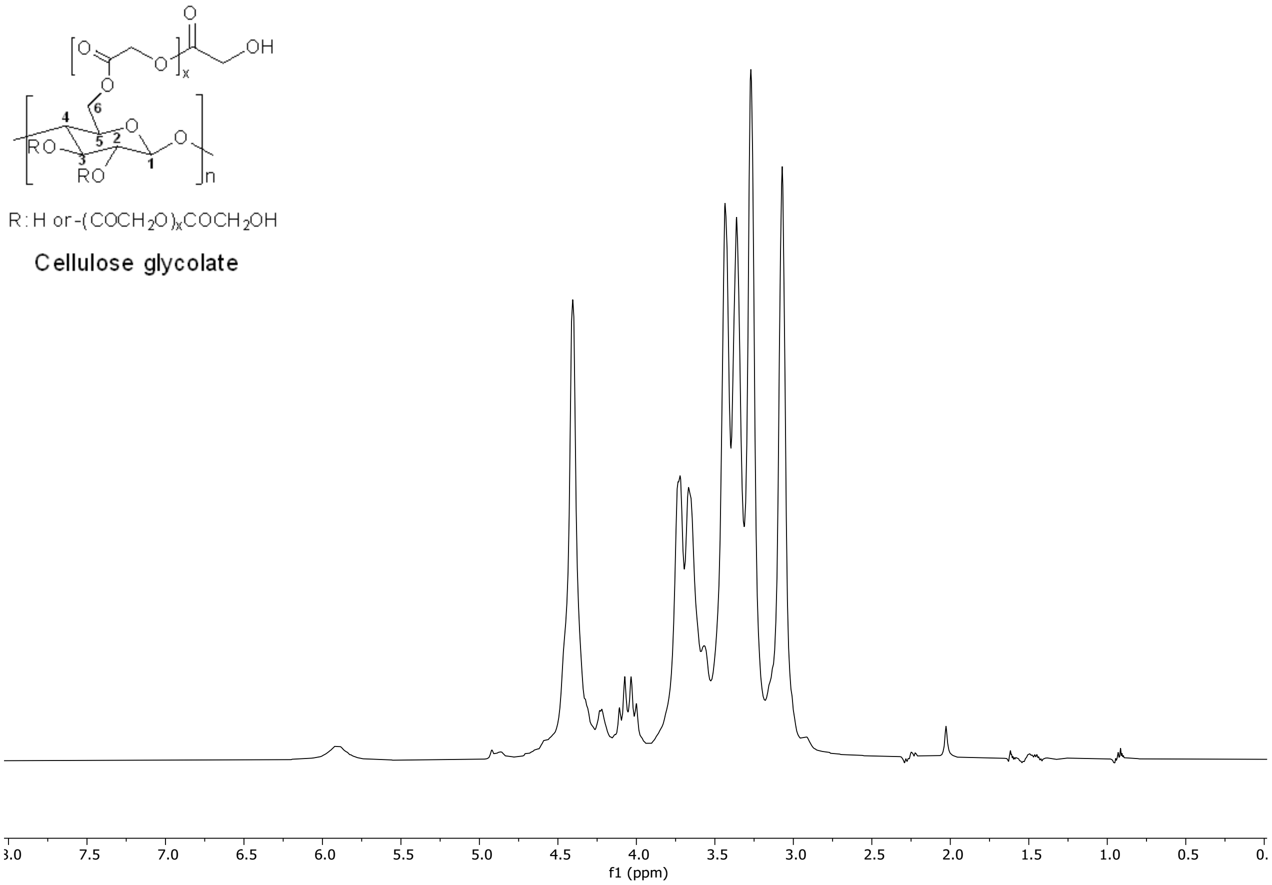

solution.

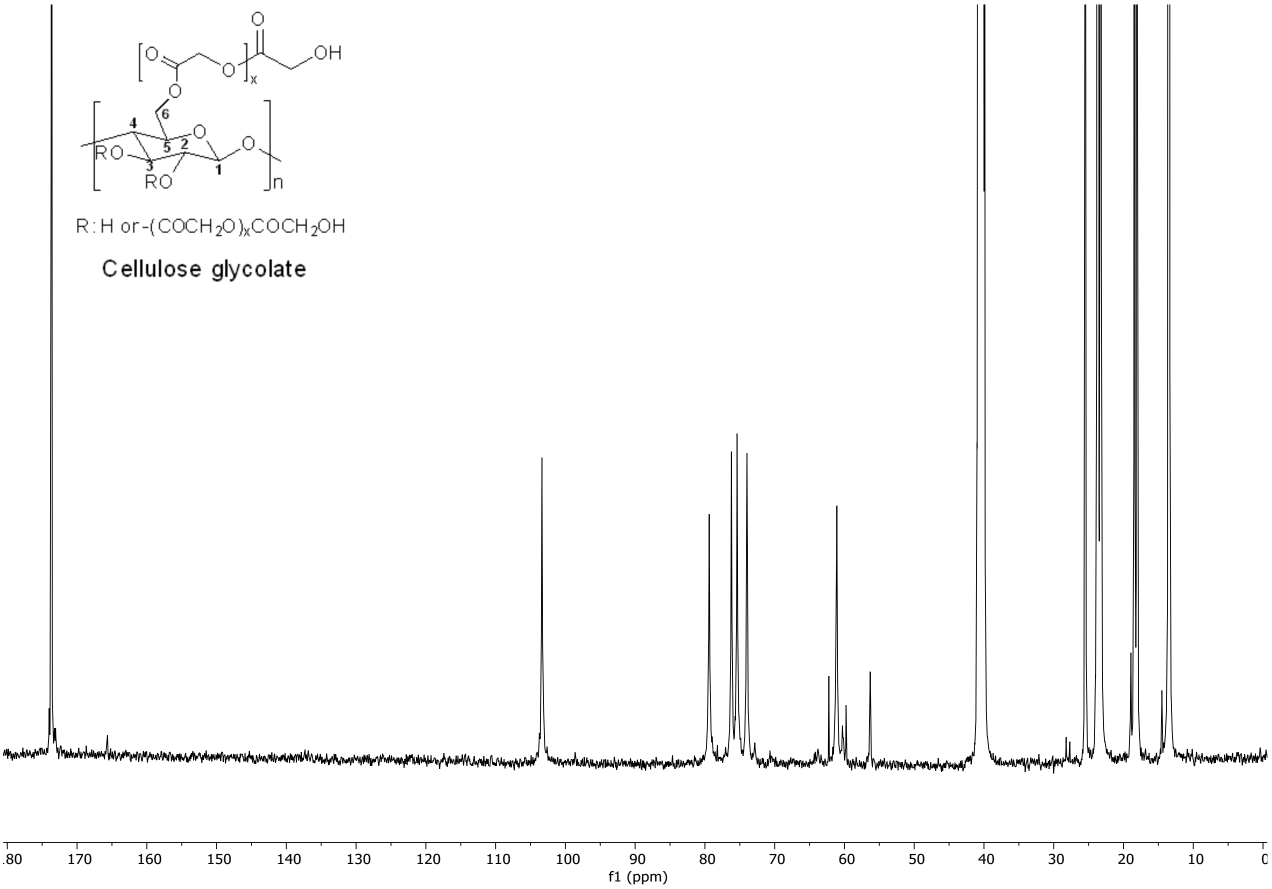

^{1}H, diffusion-edited ^{1}H, and q^{13}C{^{1}H} spectra of **Cellulose 4-oxopentanoate DS 0.01** in 20 w.% tetrabutylphosphonium acetate DMSO-d_6 electrolyte solution.

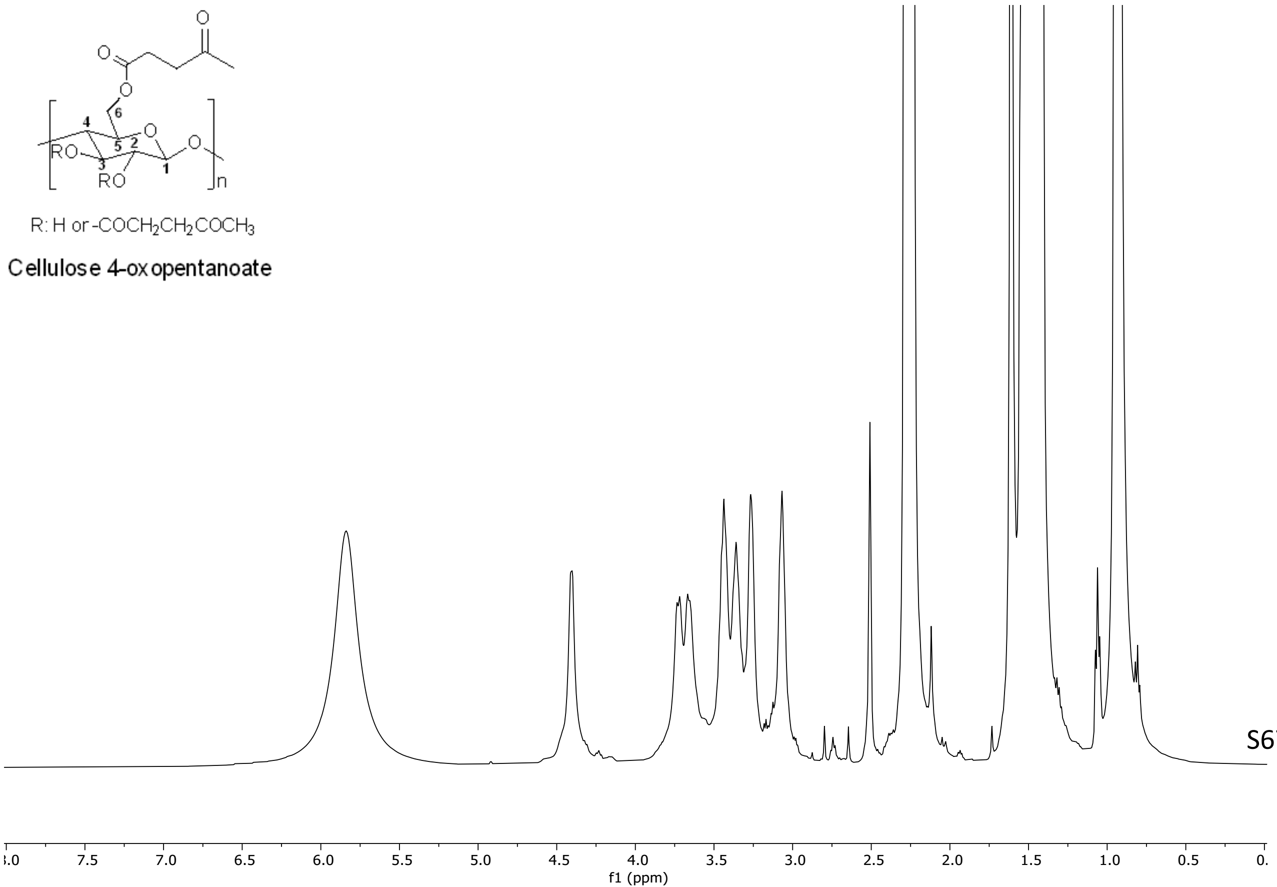

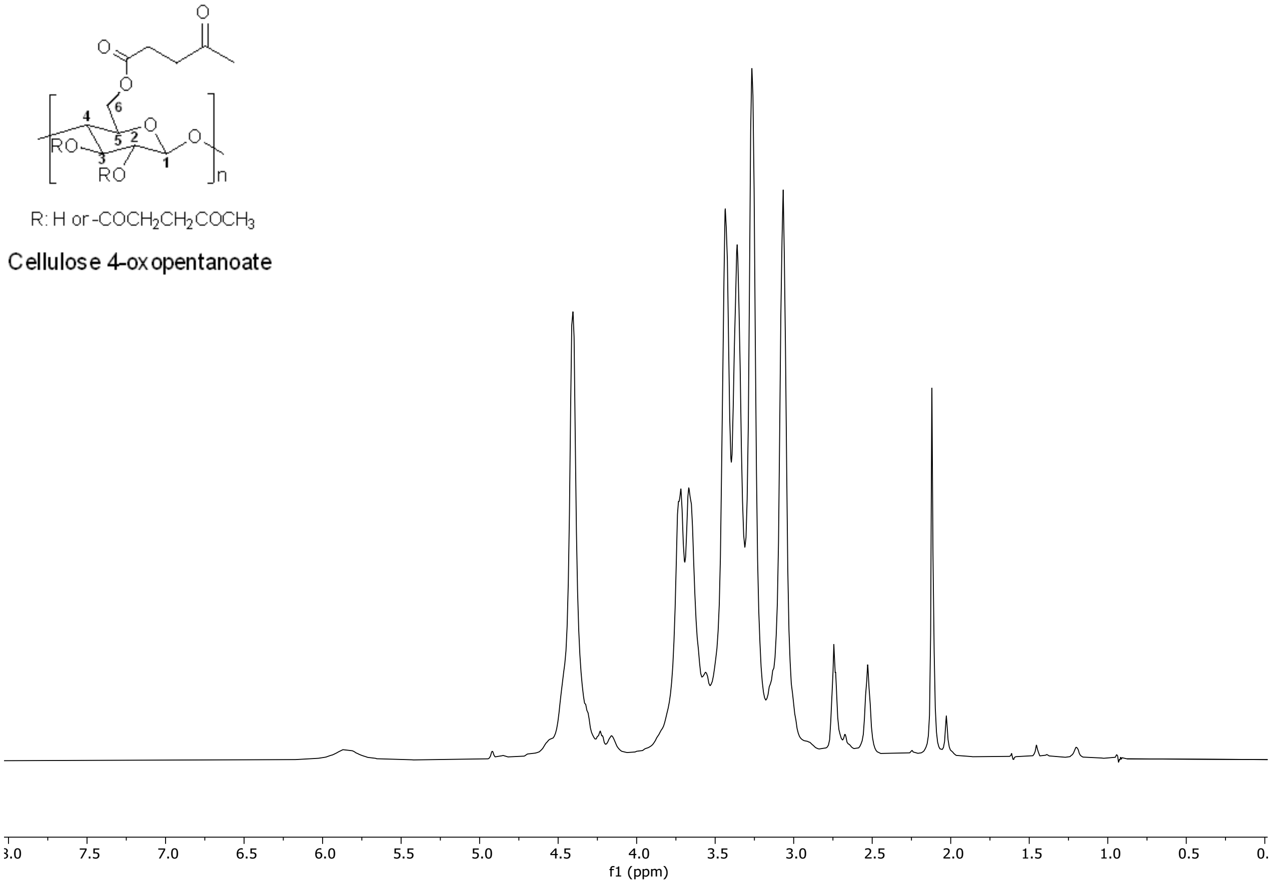
R: H or -COCH2CH2COCH3
Cellulose 4-oxopentanoate
f1 (ppm)

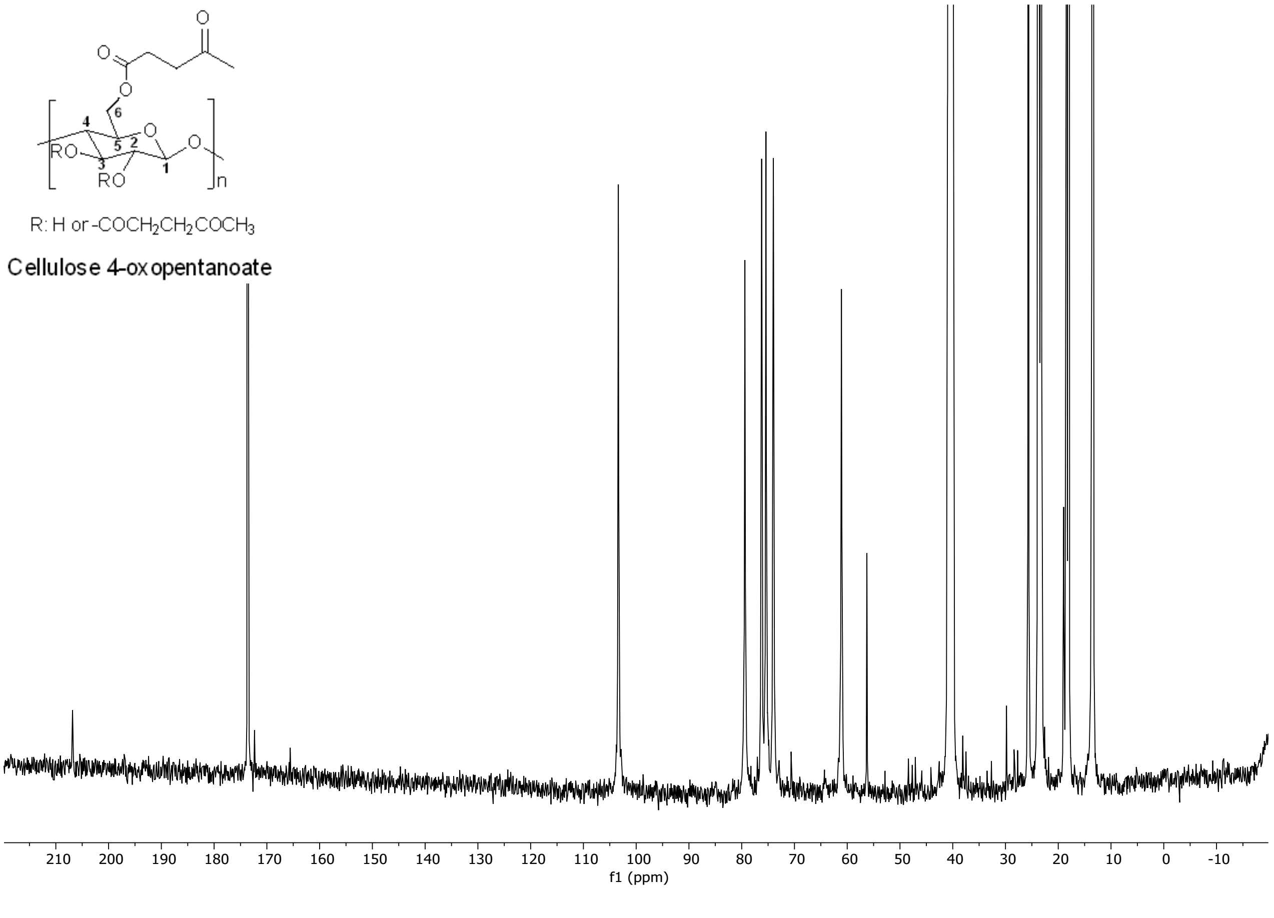
R: H or -COCH2CH2COCH3
Cellulose 4-oxopentanoate
f1 (ppm)

^{1}H, diffusion-edited ^{1}H, and $q^{13}C\{^{1}H\}$ spectra of **Cellulose 5-hydroxypentanoate DS 0.04, DG 2.01** in 20 w.% tetrabutylphosphonium acetate DMSO-

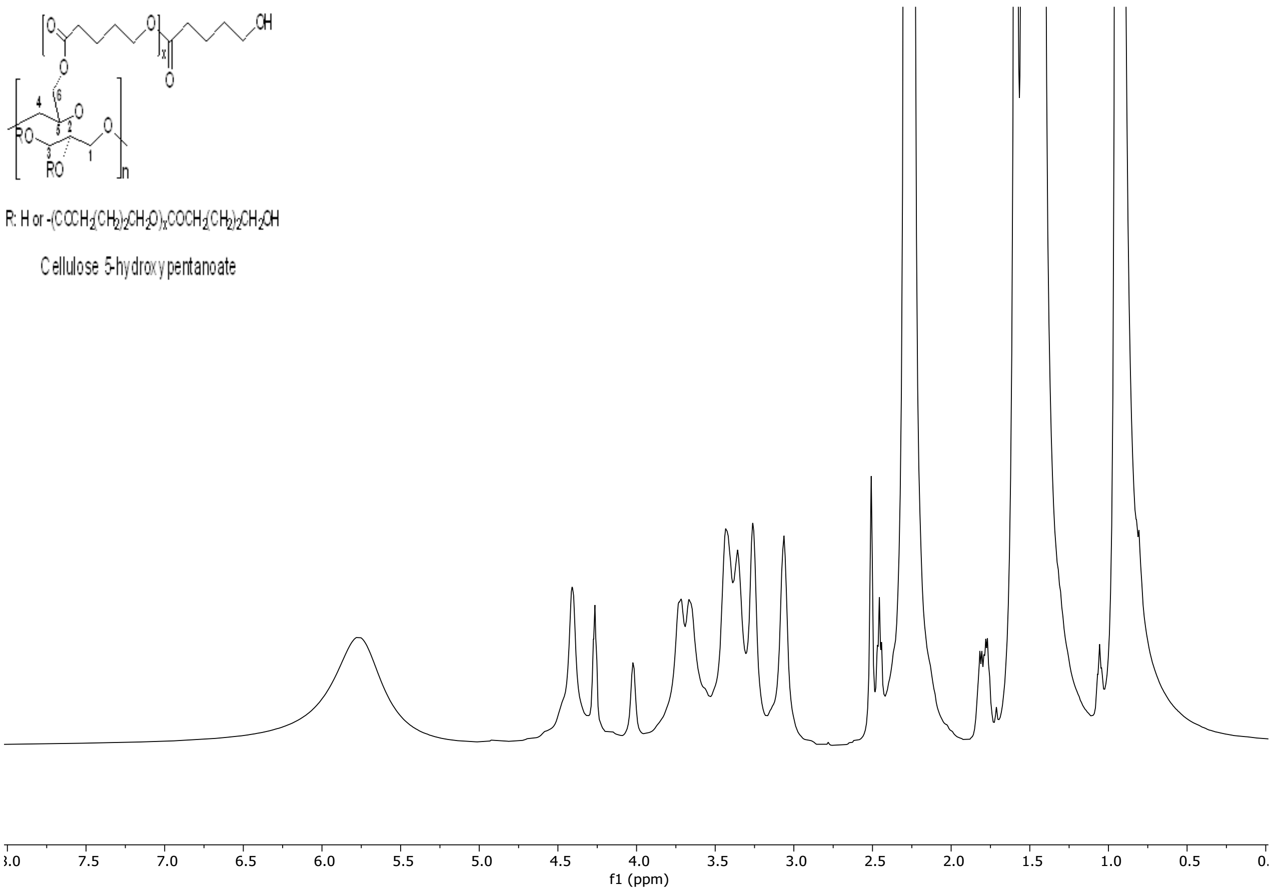

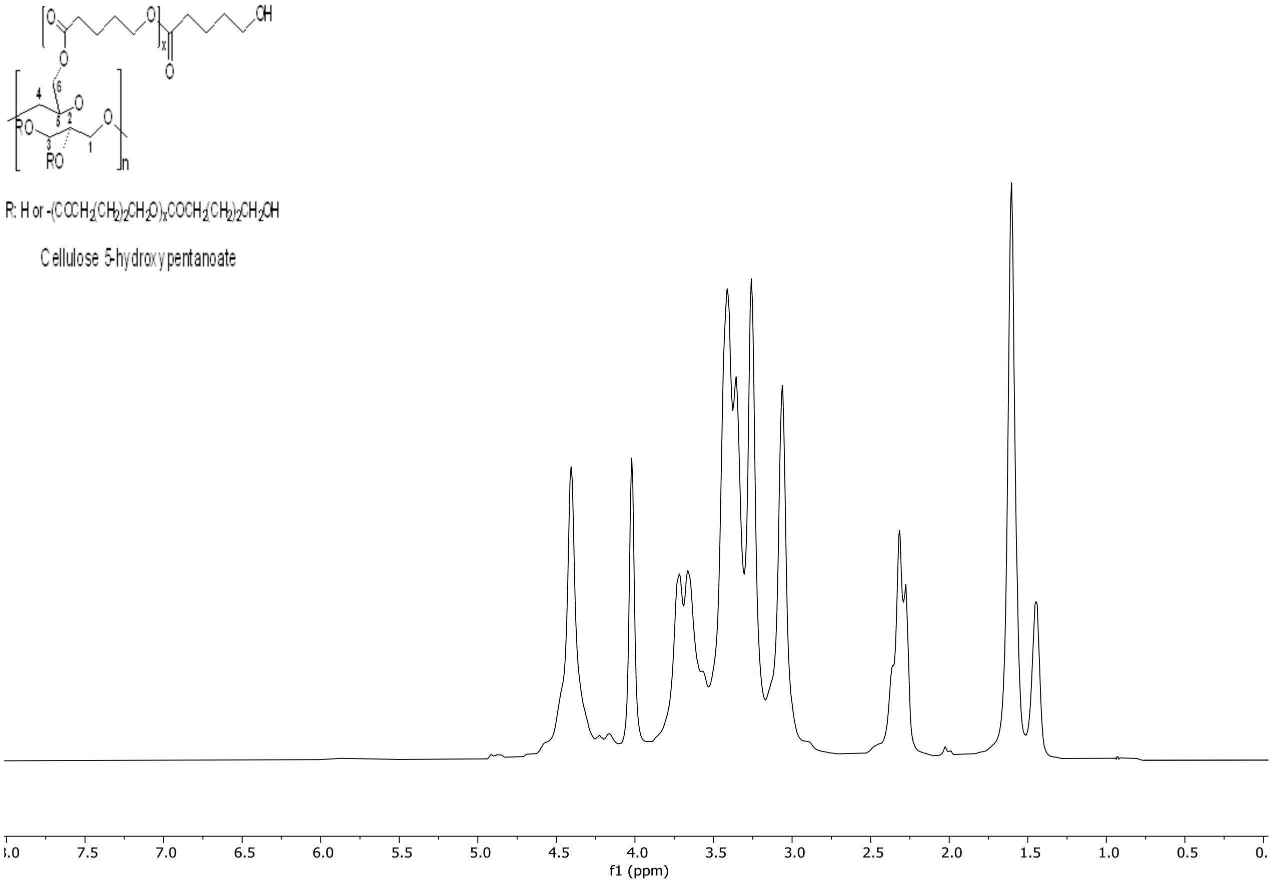

d_6 electrolyte solution.

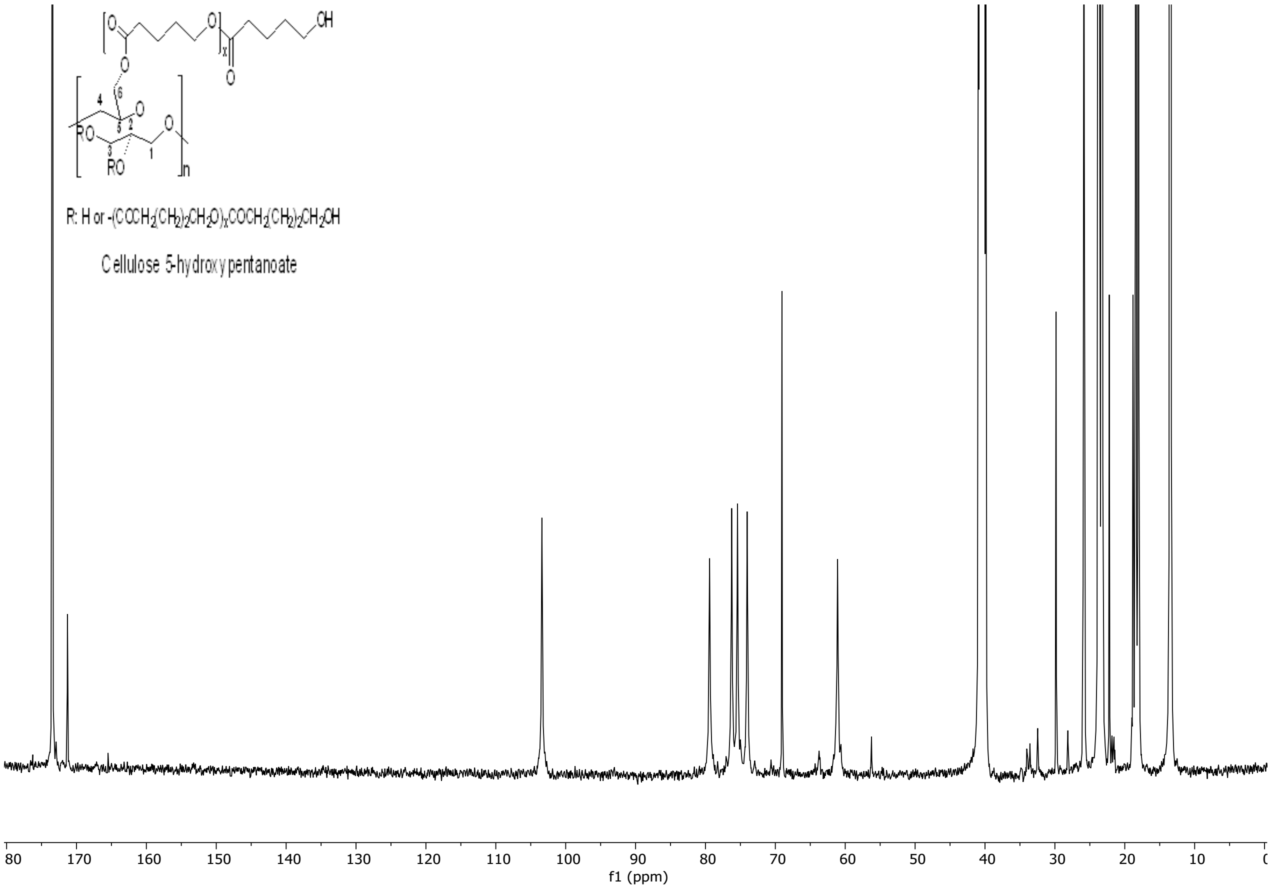

^{1}H, diffusion-edited ^{1}H, and $q^{13}C\{^{1}H\}$ spectra of **Cellulose 6-hydroxyhexanoate DS 0.24, DG 2.29** in 20 w.% tetrabutylphosphonium acetate DMSO-d_6

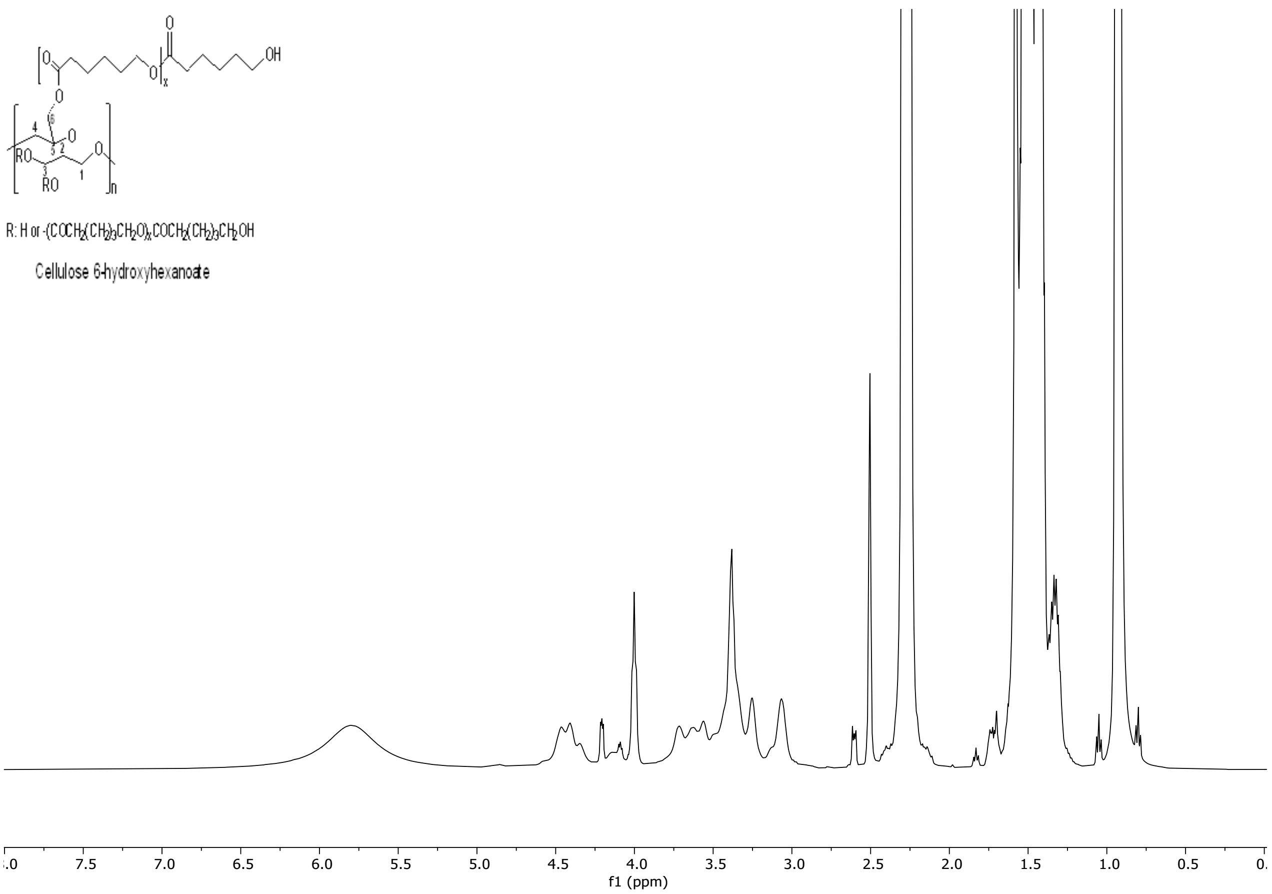

electrolyte solution.

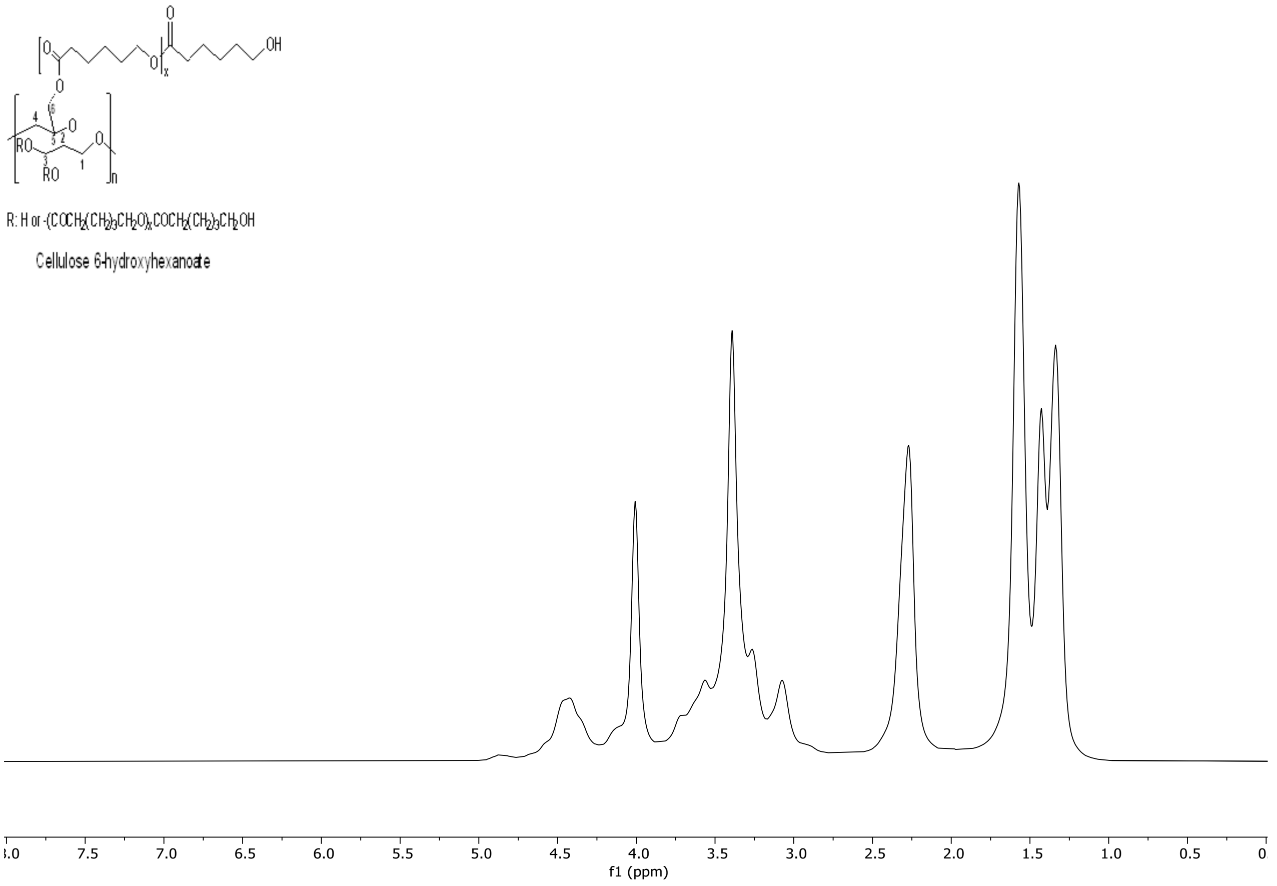
R: H or -(COCH2(CH2)3CH2O)xCOCH2(CH2)3CH2OH
Cellulose 6-hydroxyhexanoate
f1 (ppm)

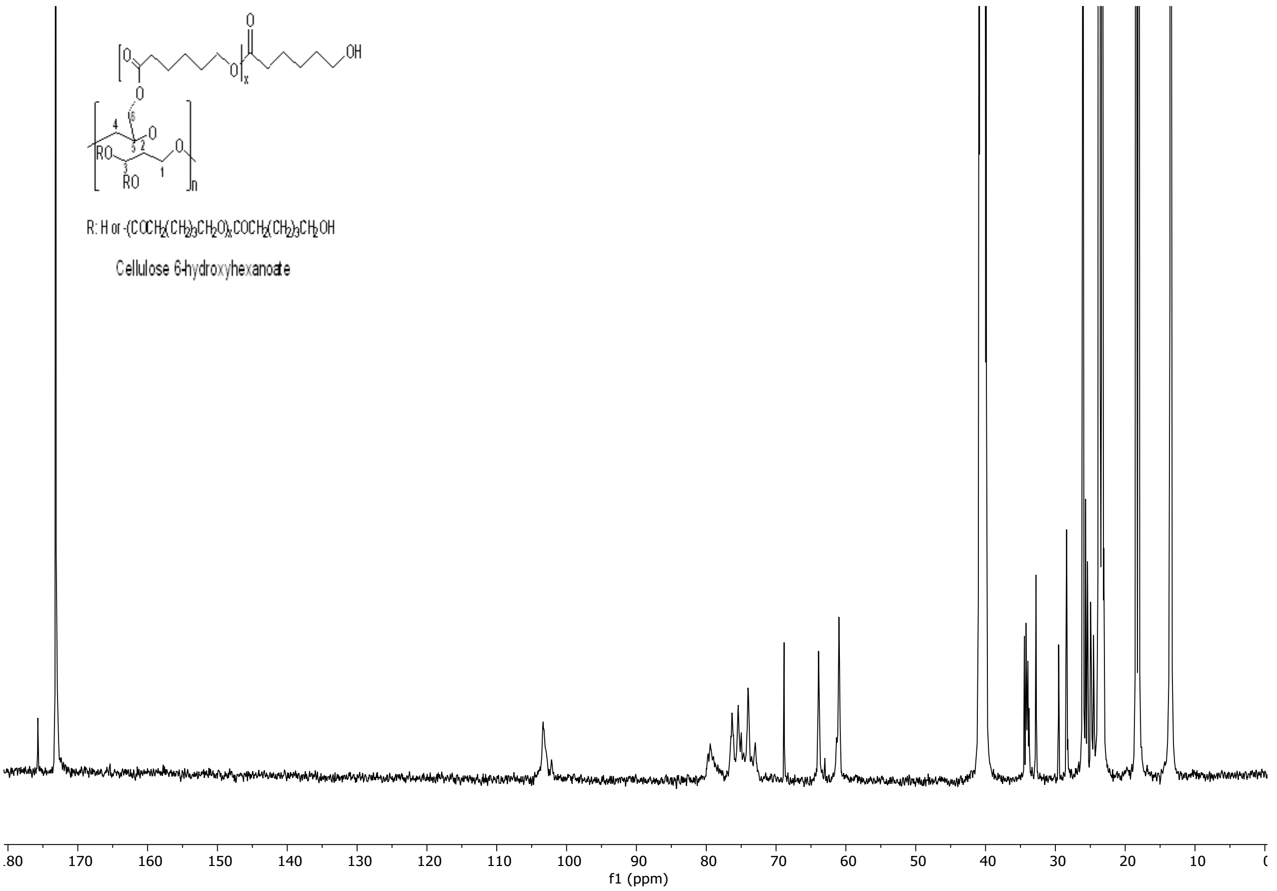
R: H or -(COCH2(CH2)3CH2O)xCOCH2(CH2)3CH2OH
Cellulose 6-hydroxyhexanoate
f1 (ppm)

^{1}H, diffusion-edited ^{1}H, and $q^{13}C\{^{1}H\}$ spectra of **Cellulose 6-hydroxyhexanoate DS 0.01, DG 1.84** in 20 w.% tetrabutylphosphonium acetate DMSO-d_6

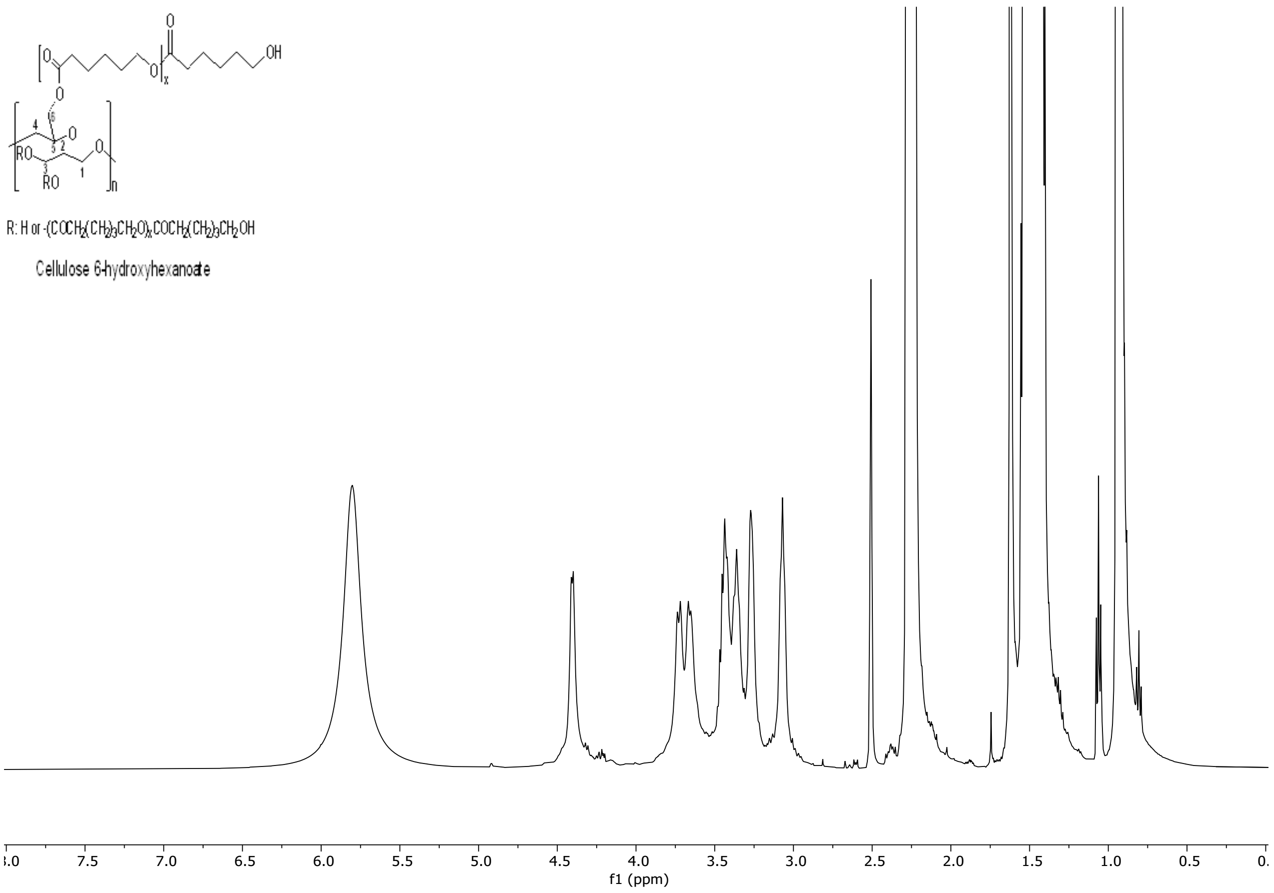

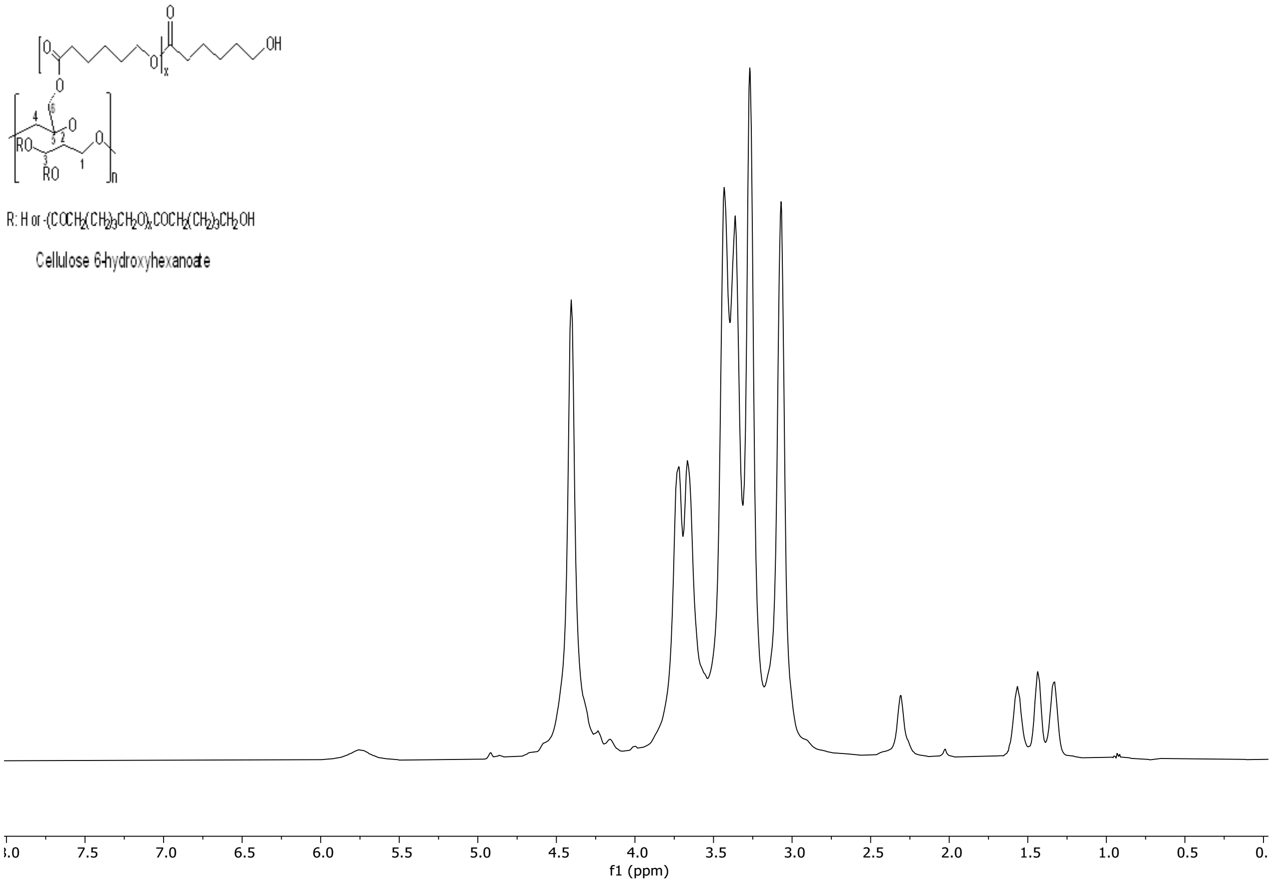

electrolyte solution.

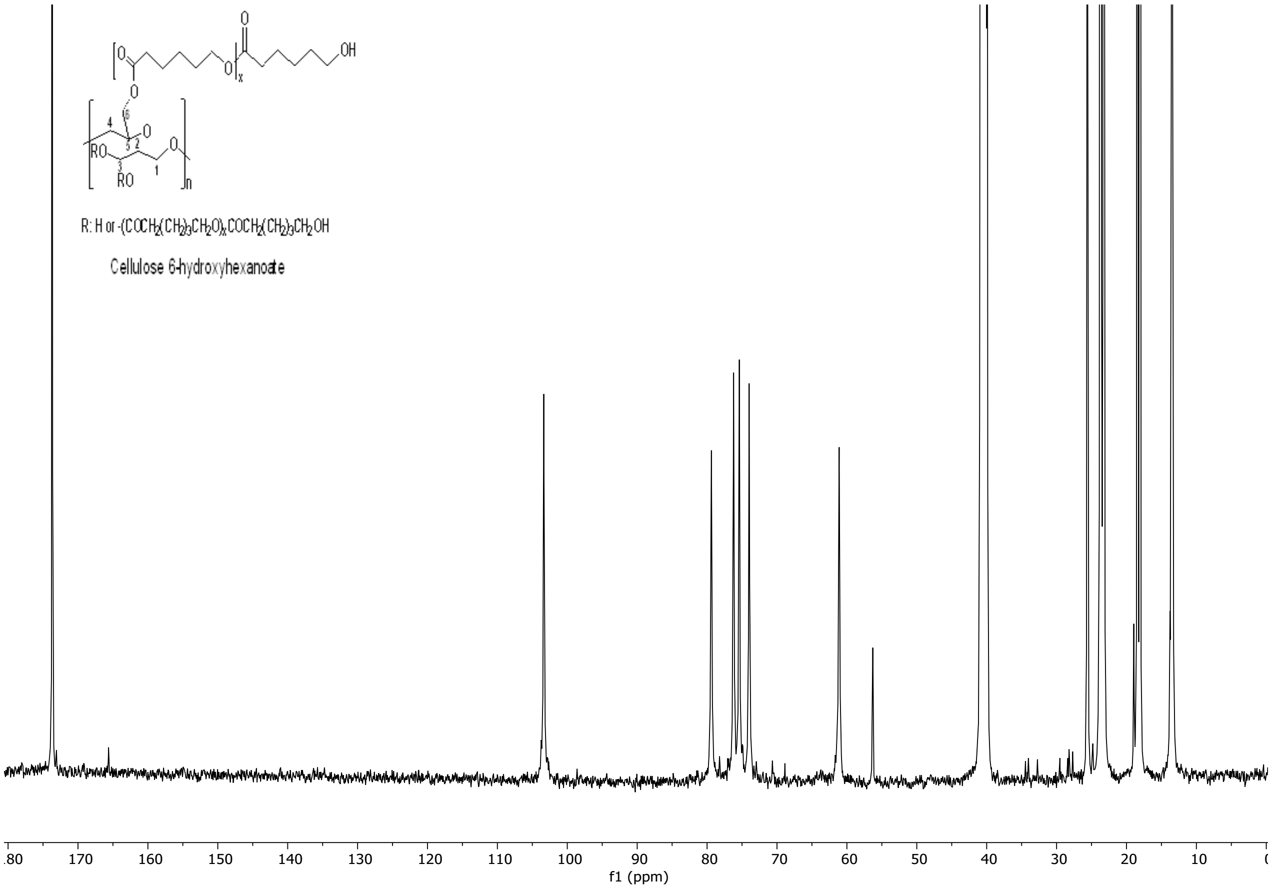

^{1}H, diffusion-edited ^{1}H, and $q^{13}C\{^{1}H\}$ spectra of **Cellulose 6-hydroxyhexanoate DS 0.03, DG 1.64** in 20 w.% tetrabutylphosphonium acetate DMSO-d_6

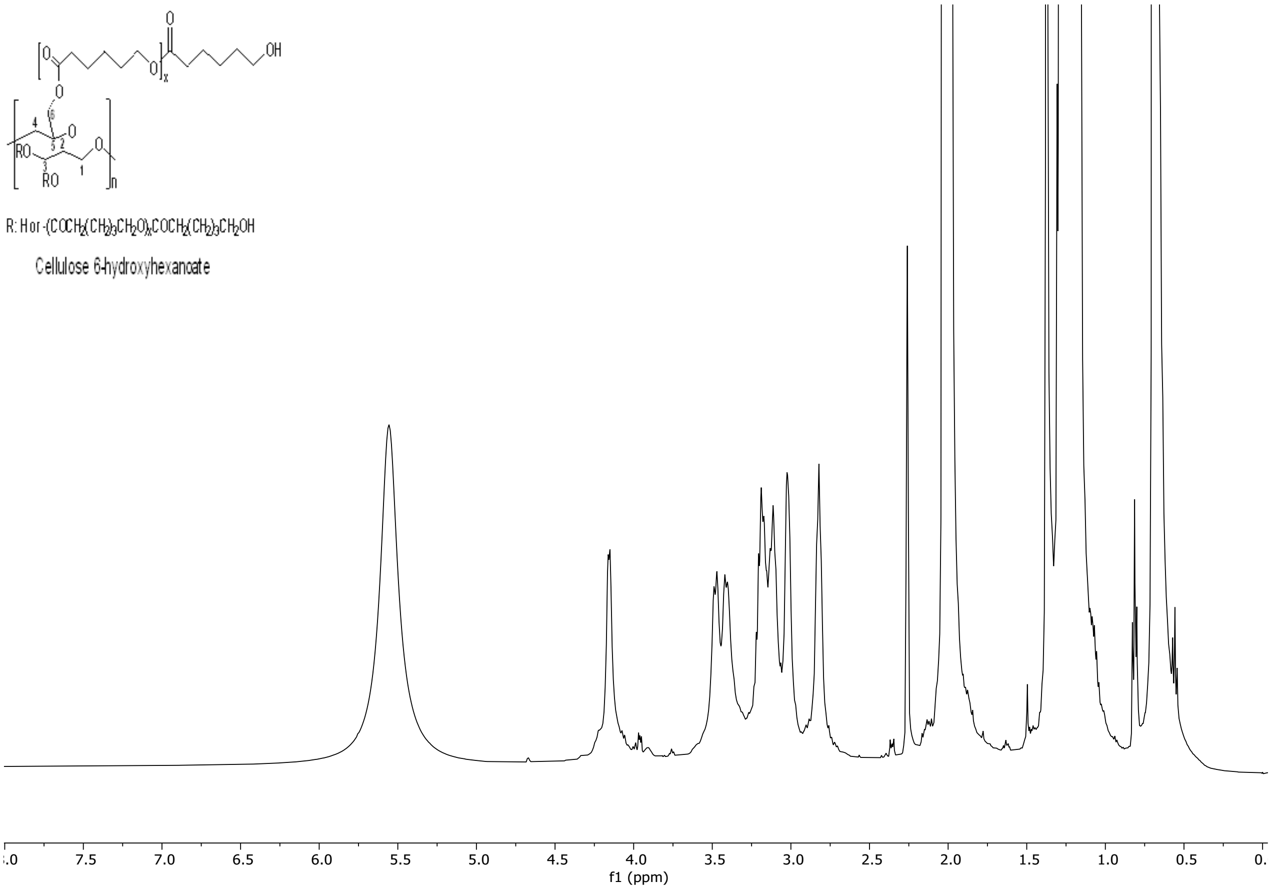

electrolyte solution.

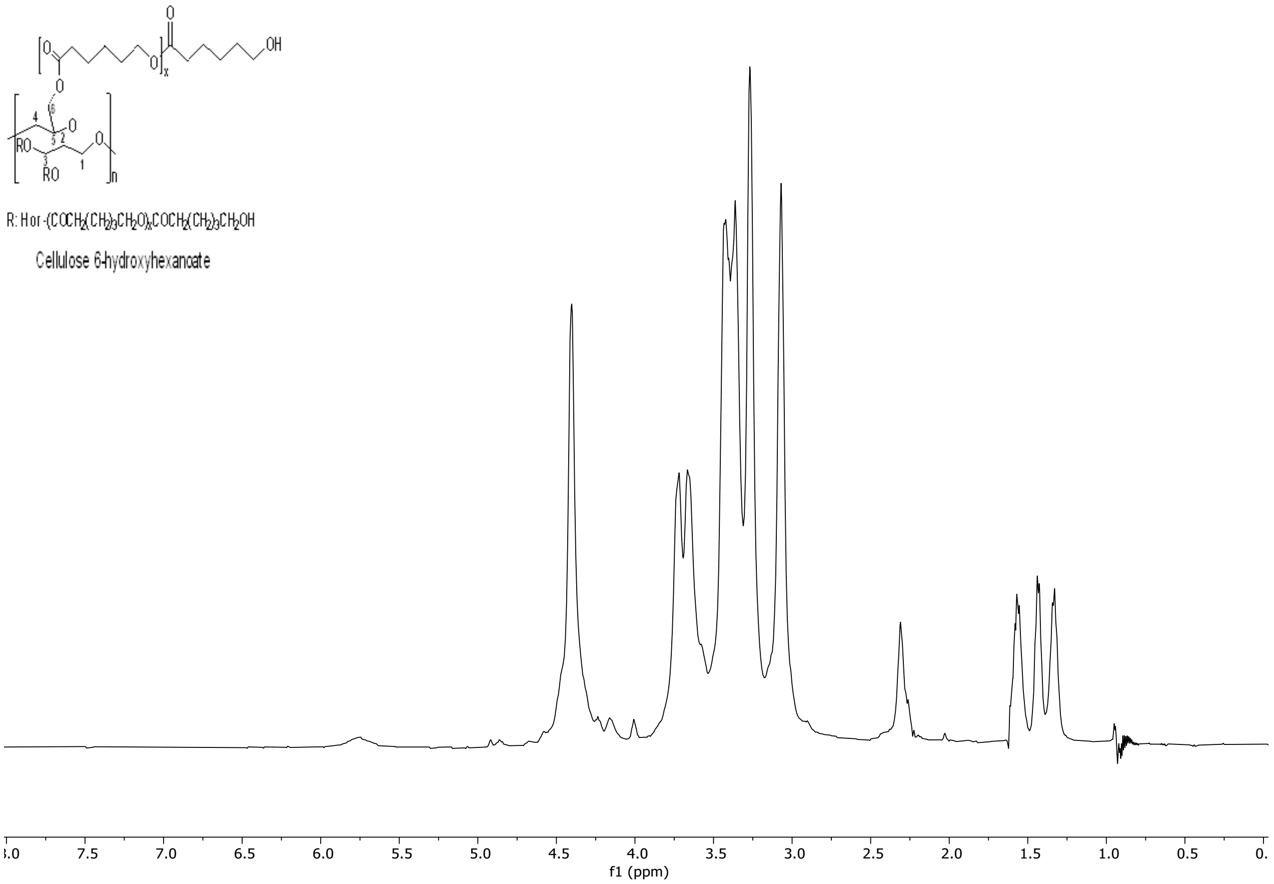
OH
R: H or -(COCH2(CH2)3CH2O)xCOCH2(CH2)3CH2OH
Cellulose 6-hydroxyhexanoate
f1 (ppm)

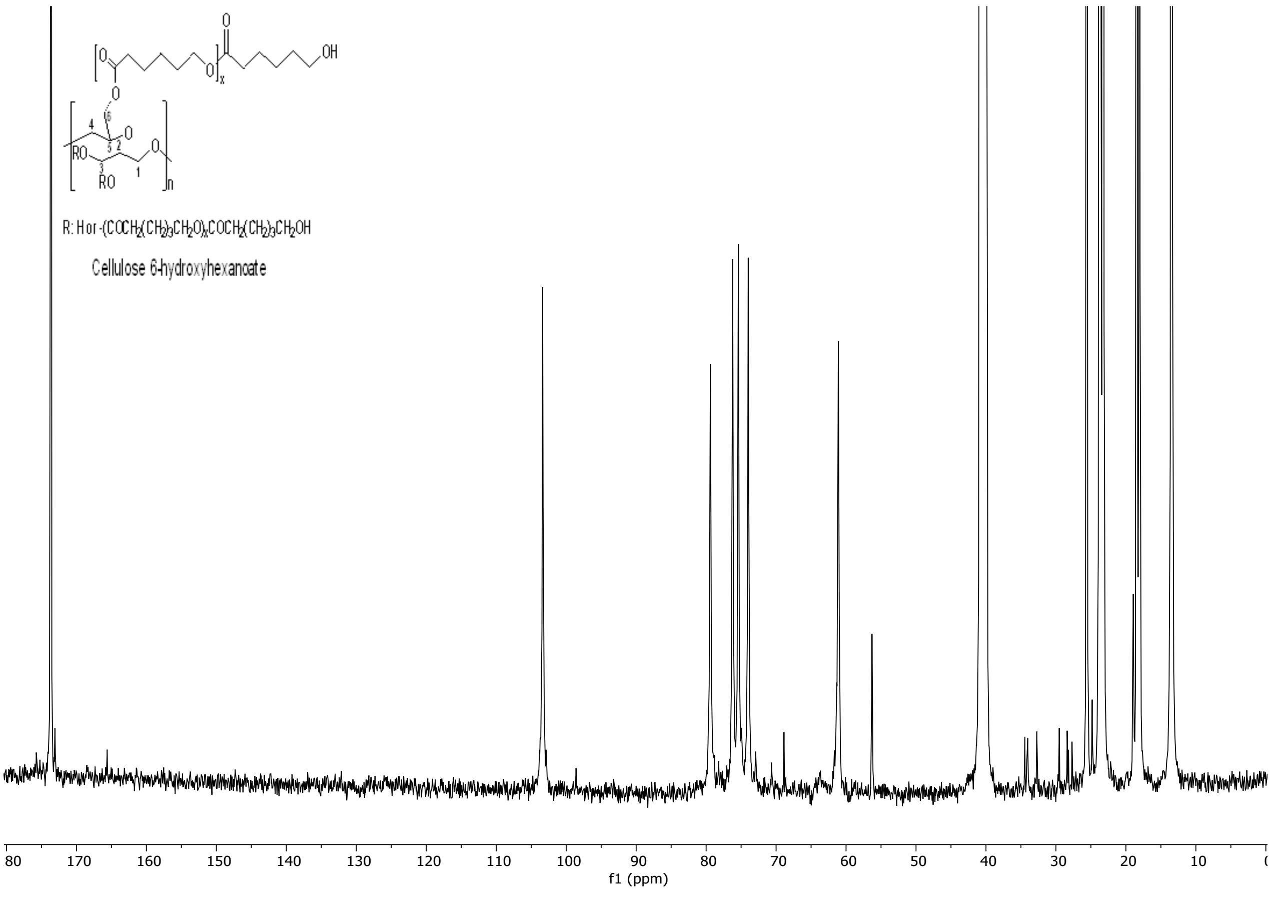
OH
R: H or -(COCH2(CH2)3CH2O)xCOCH2(CH2)3CH2OH
Cellulose 6-hydroxyhexanoate
f1 (ppm)

^{1}H, diffusion-edited ^{1}H, and q^{13}C{^{1}H} spectra of **Cellulose 6-hydroxyhexanoate DS 0.05, DG 1.56** in 20 w.% tetrabutylphosphonium acetate DMSO-d_6

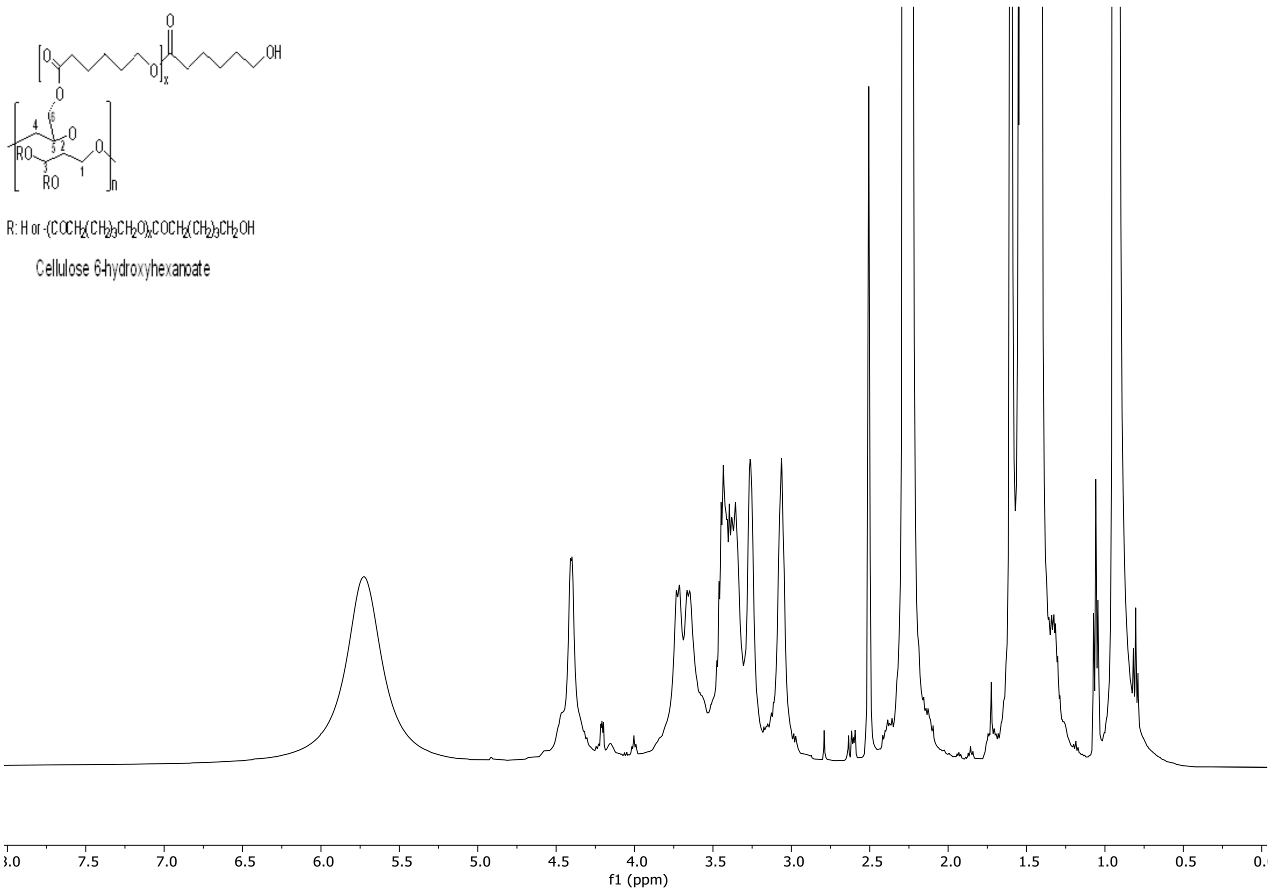

electrolyte solution.

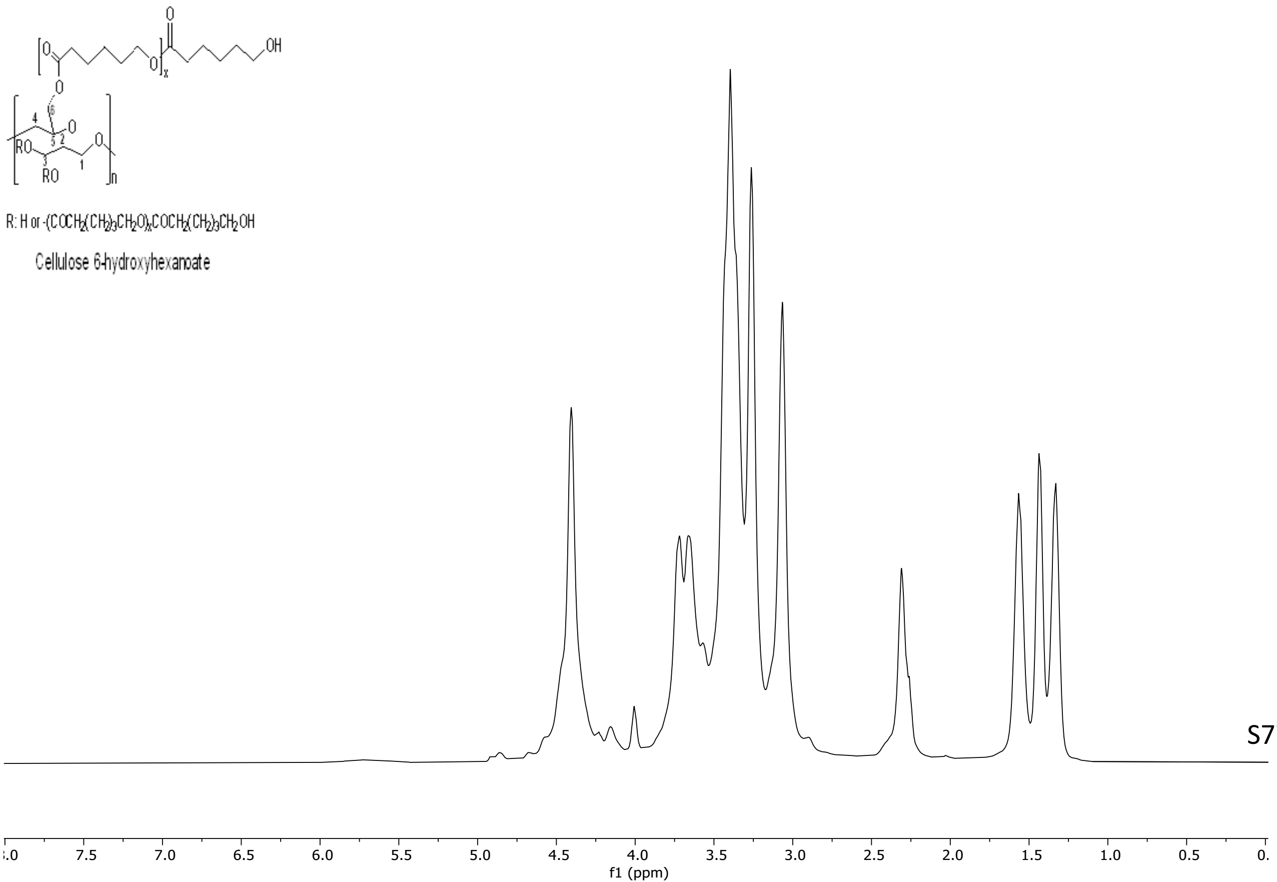

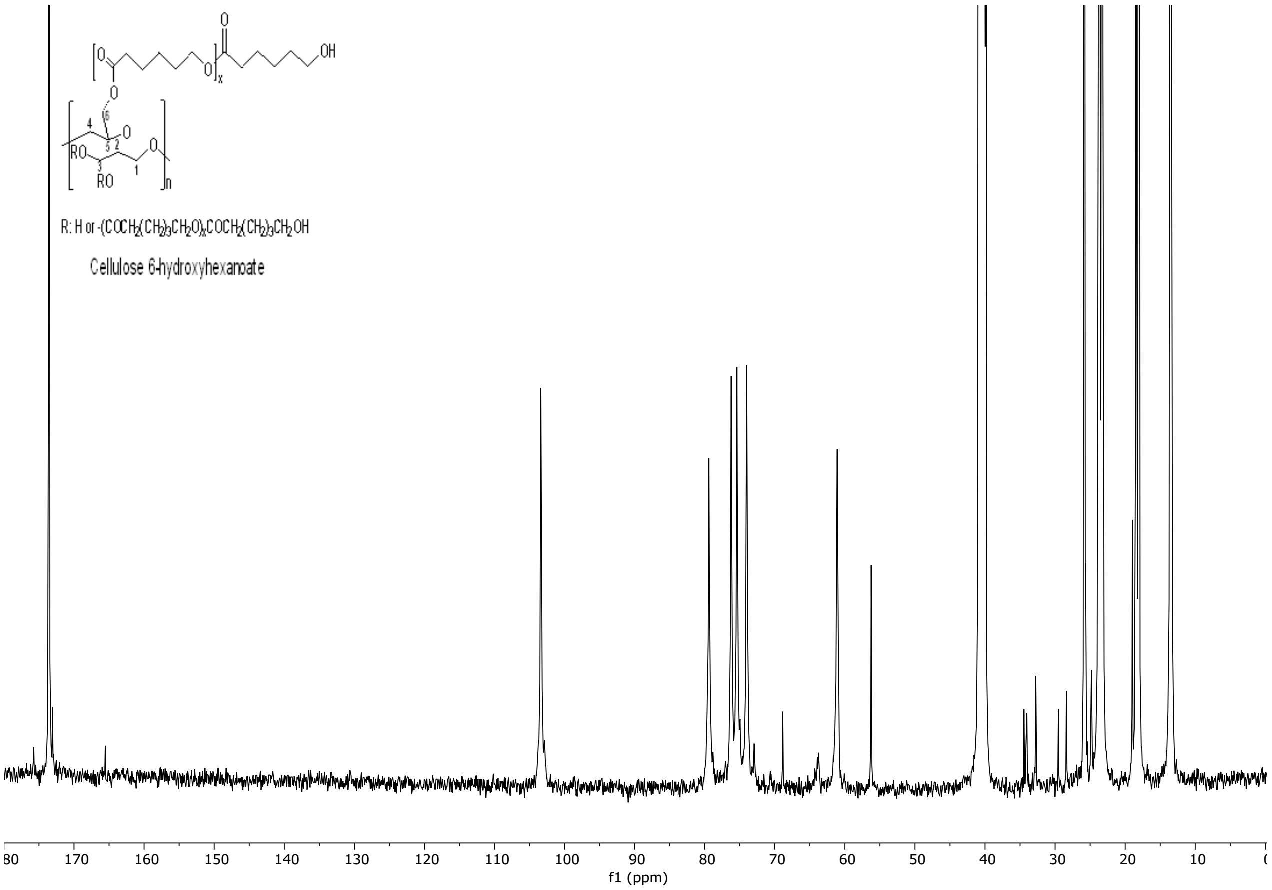

R: H or -(COCH2(CH2)3CH2O)xCOCH2(CH2)3CH2OH
Cellulose 6-hydroxyhexanoate
f1 (ppm)

^{1}H, diffusion-edited ^{1}H, and $q^{13}C\{^{1}H\}$ spectra of **Cellulose 6-hydroxyhexanoate DS 0.07, DG 1.53** in 20 w.% tetrabutylphosphonium acetate DMSO-d_6

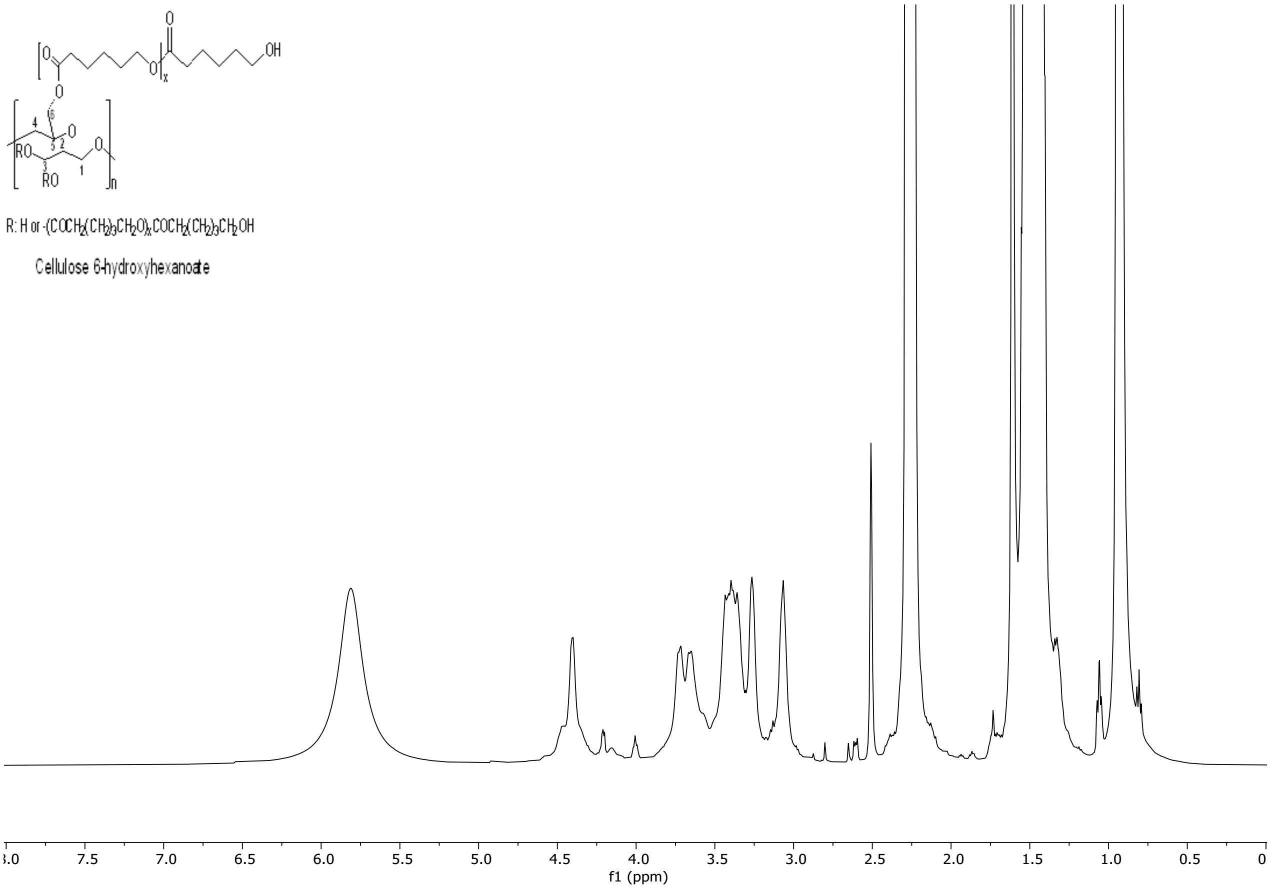

electrolyte solution.

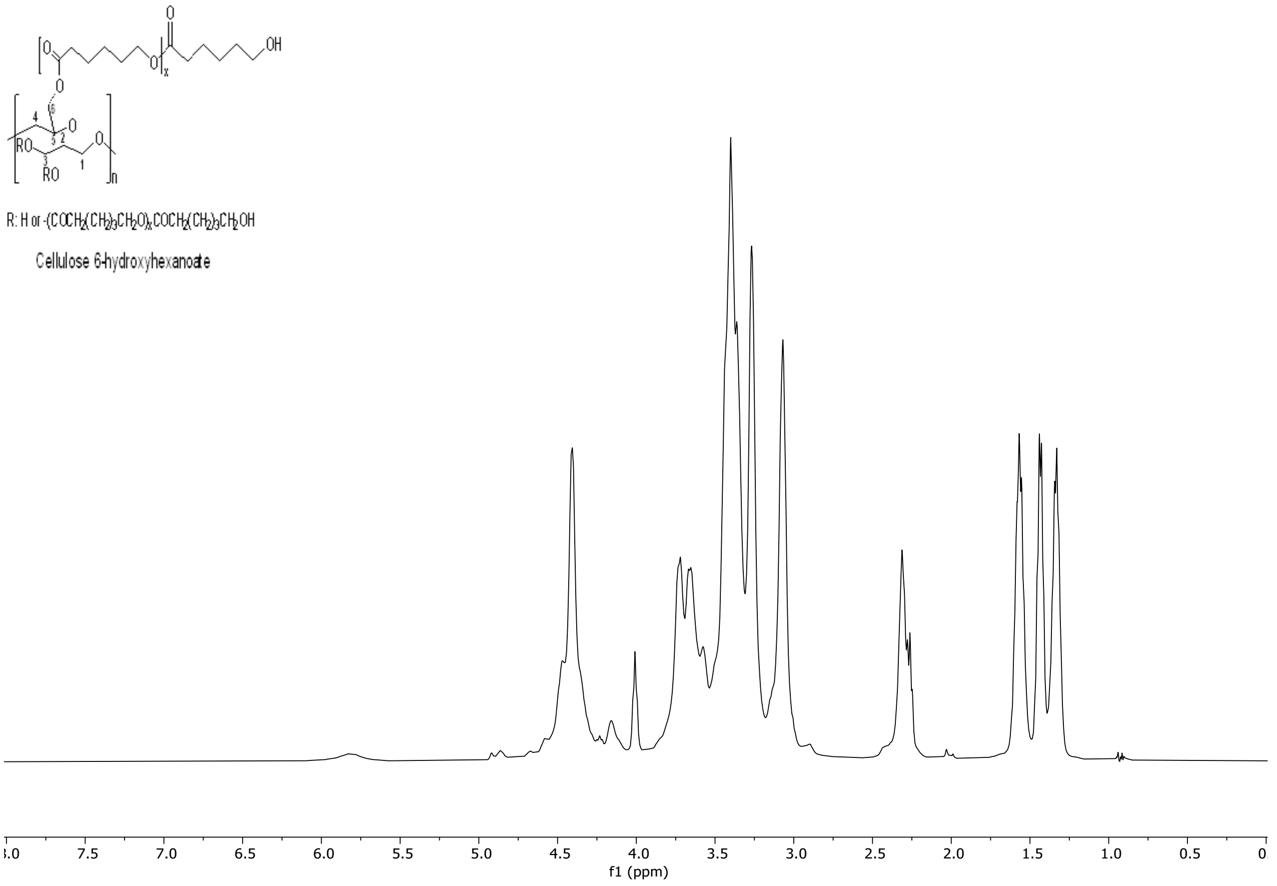
O
O
O
OH
x
O
6
4
O
5 2
RO
O
3
1
RO
n
R: H or -(COCH2(CH2)3CH2O)xCOCH2(CH2)3CH2OH
Cellulose 6-hydroxyhexanoate
f1 (ppm)

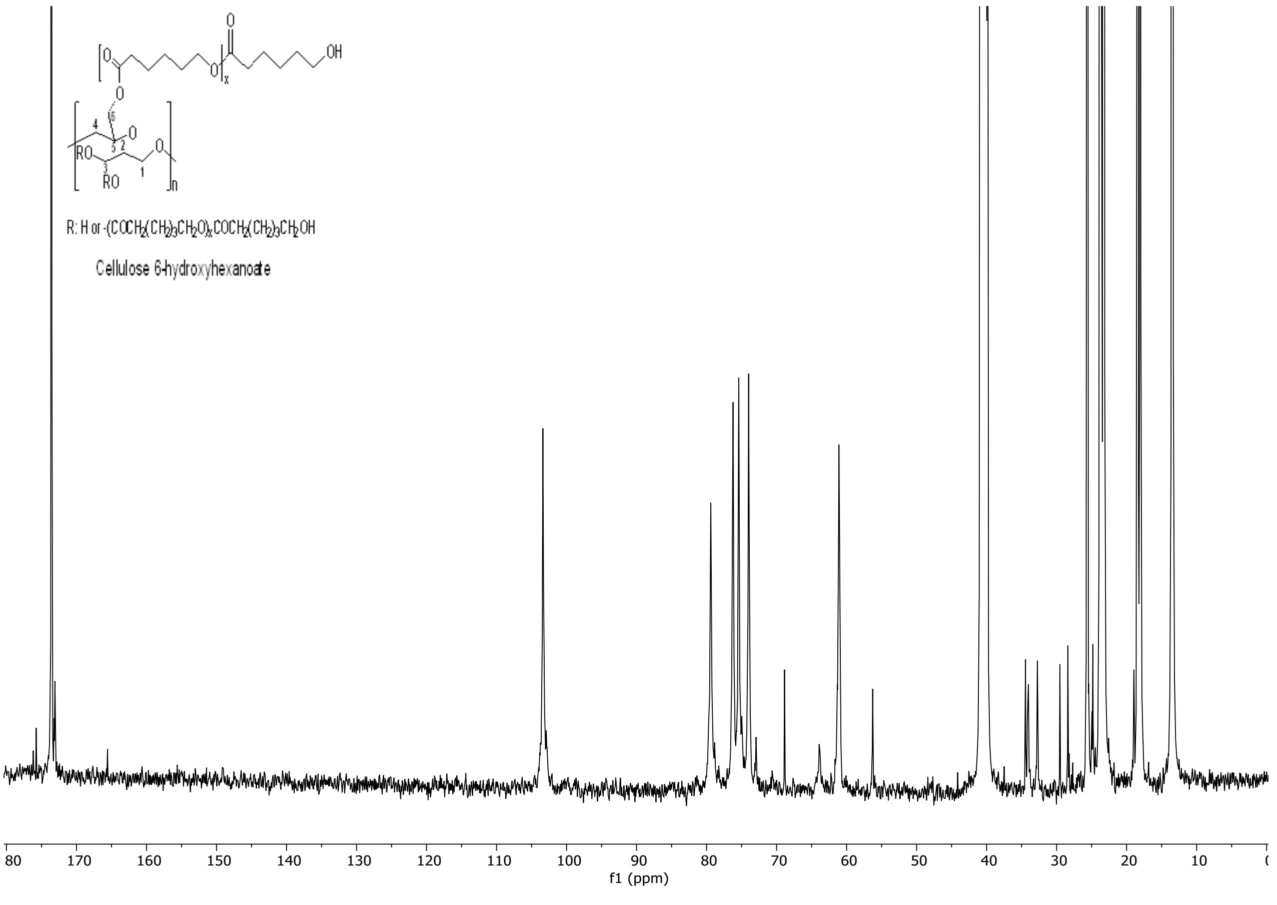
R: H or -(COCH2(CH2)3CH2O)xCOCH2(CH2)3CH2OH
Cellulose 6-hydroxyhexanoate
f1 (ppm)

^{1}H, diffusion-edited ^{1}H, and $q^{13}C\{^{1}H\}$ spectra of **Cellulose 6-hydroxyhexanoate DS 0.07, DG 1.58** in 20 w.% tetrabutylphosphonium acetate DMSO-d_6

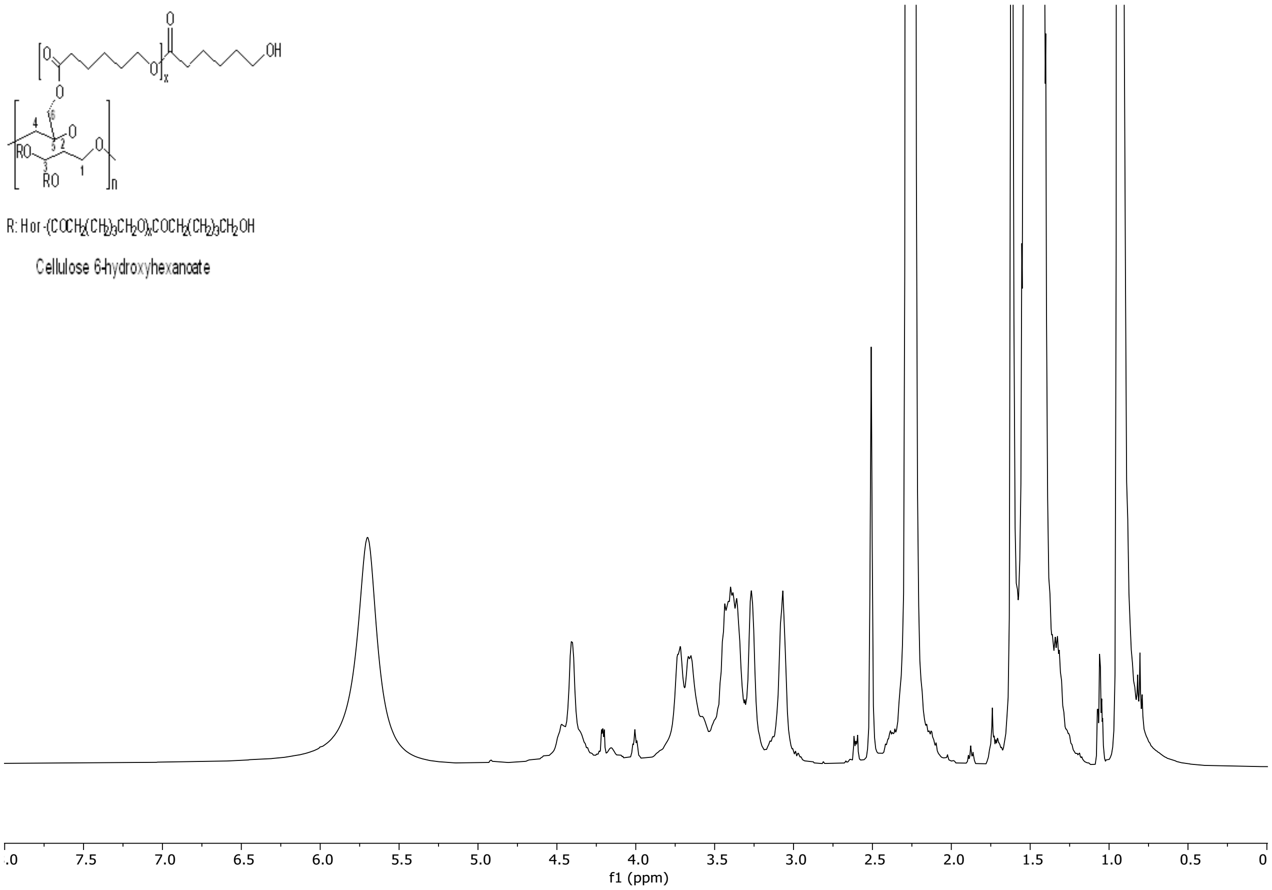

electrolyte solution.

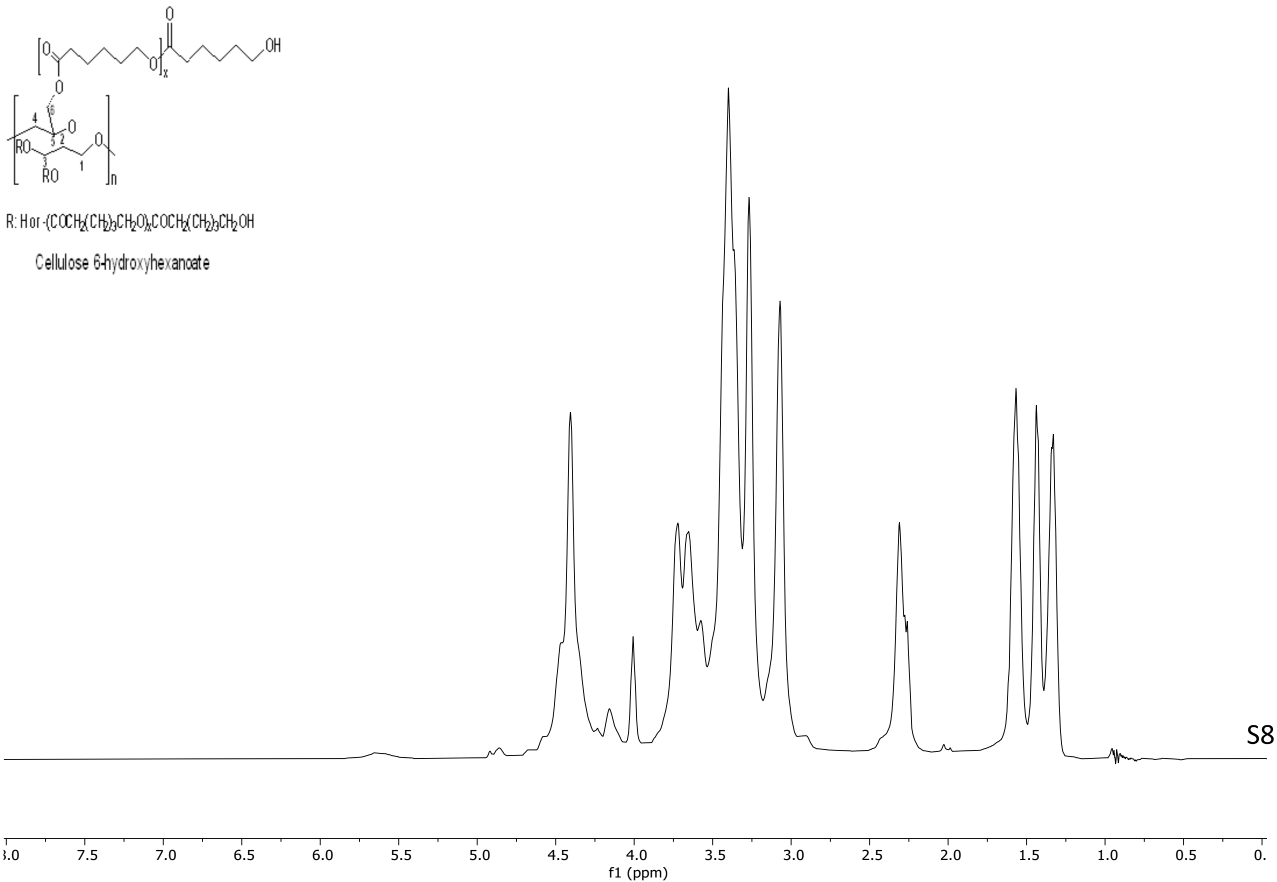

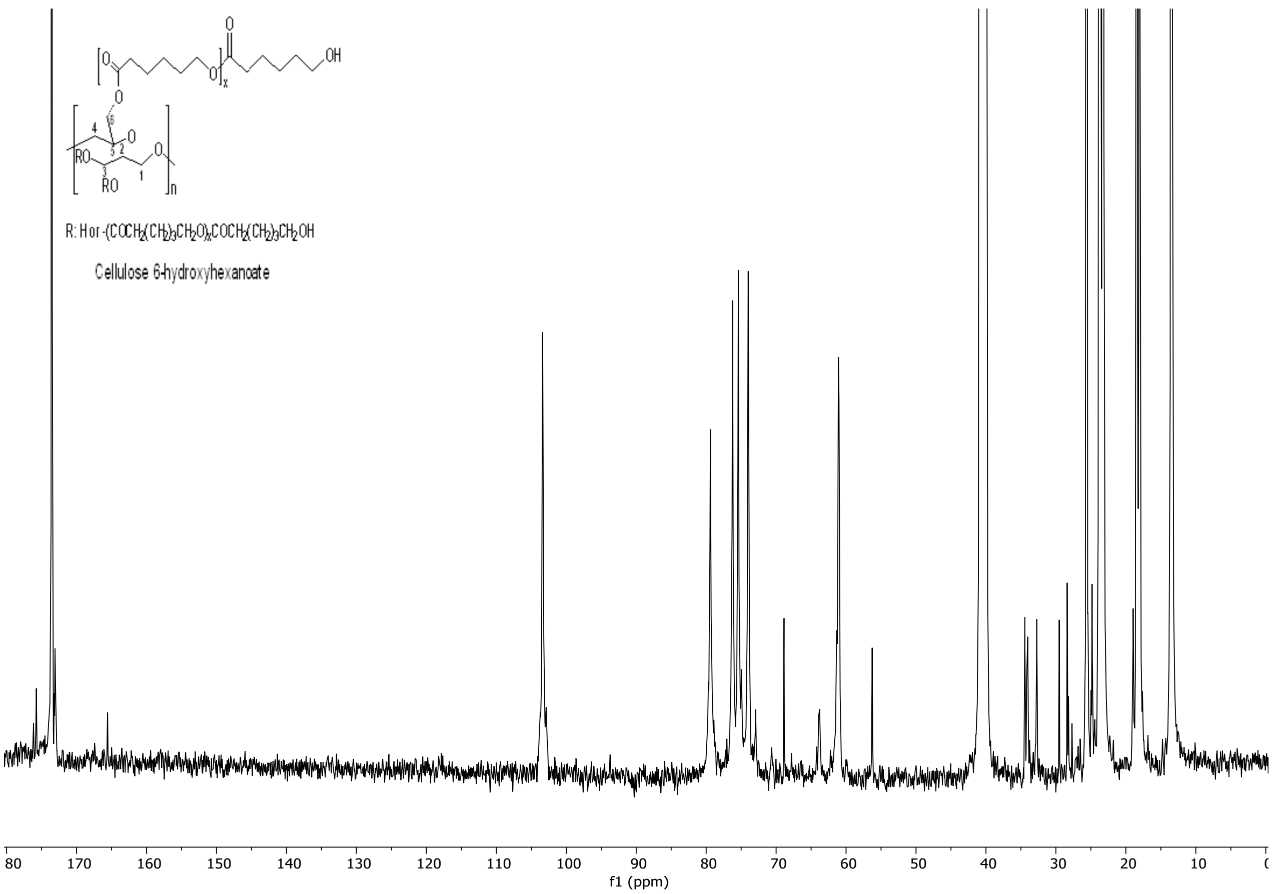
R: H or -(COCH2(CH2)3CH2O)xCOCH2(CH2)3CH2OH
Cellulose 6-hydroxyhexanoate
180 170 160 150 140 130 120 110 100 90 80 70 60 50 40 30 20 10
f1 (ppm)

^{1}H, diffusion-edited ^{1}H, and $q^{13}C\{^{1}H\}$ spectra of **Cellulose 6-hydroxyhexanoate DS 0.10, DG 1.76** in 20 w.% tetrabutylphosphonium acetate DMSO-d_6

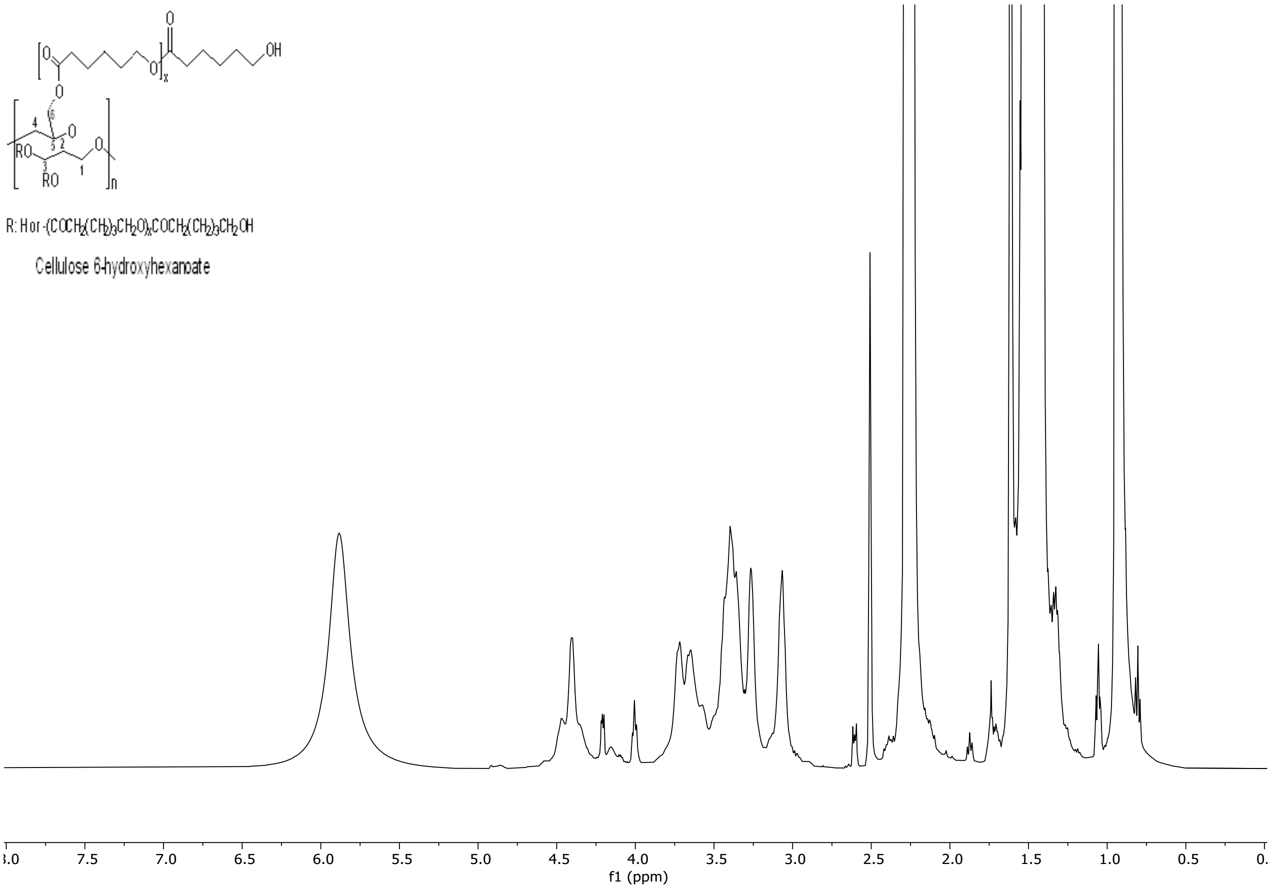

electrolyte solution.

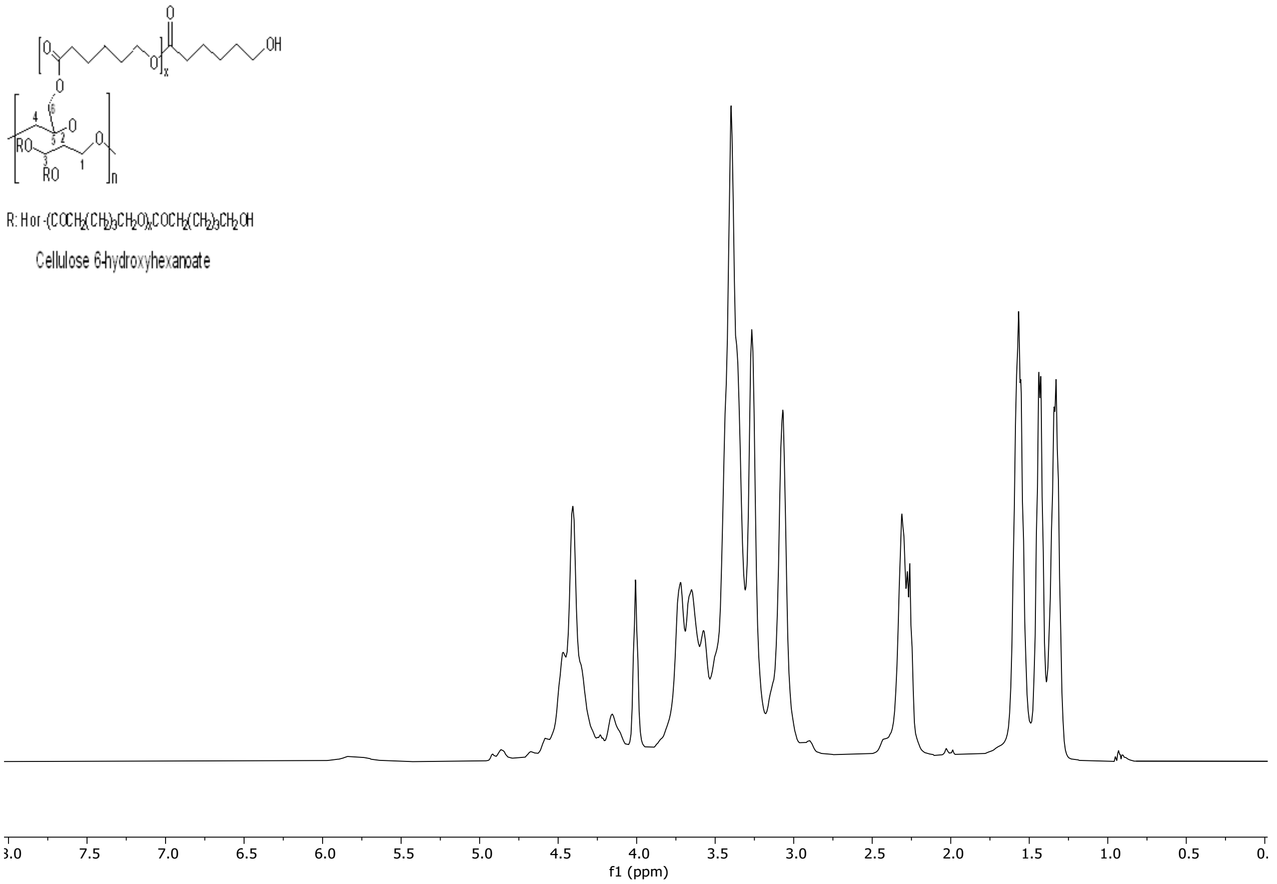
OH
R: H or -(COCH2(CH2)3CH2O)xCOCH2(CH2)3CH2OH
Cellulose 6-hydroxyhexanoate
f1 (ppm)

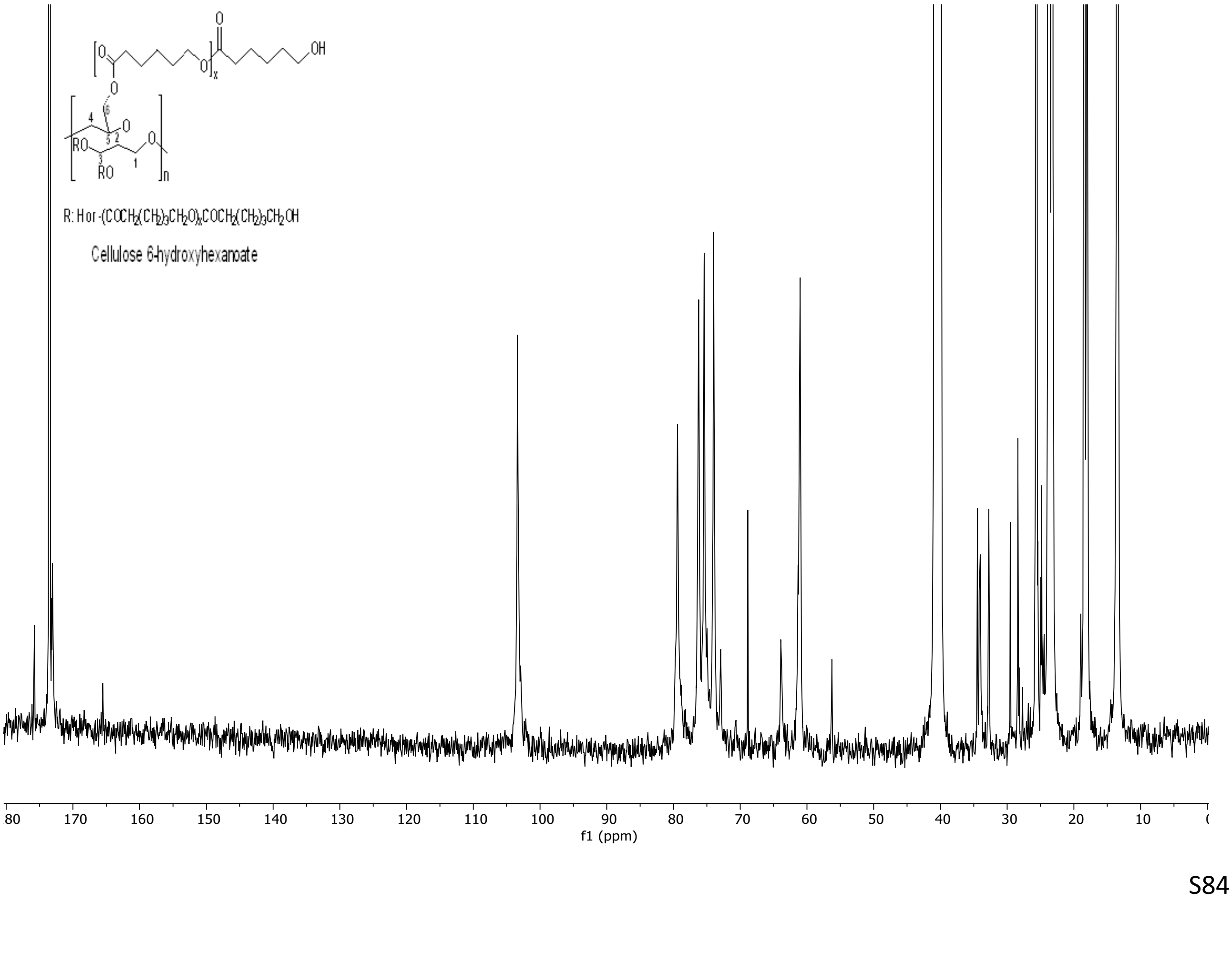
OH
R: H or -(COCH2(CH2)3CH2O)xCOCH2(CH2)3CH2OH
Cellulose 6-hydroxyhexanoate
f1 (ppm)
